# Engineering a Green Fluorescent Protein-Core-Inspired NIR-Photocage: Exploring *meso*-GFP-PRPG toward Alzheimer’s Disease Therapeutics

**DOI:** 10.1021/acscentsci.5c00027

**Published:** 2025-03-20

**Authors:** Saugat Mondal, Jusung An, Tapas Bera, Moumita Banerjee, Snehasish Debnath, Debasish Mandal, Antara Sikder, Samit Guha, Jong Seung Kim, N. D. Pradeep Singh

**Affiliations:** † Department of Chemistry, 30133 Indian Institute of Technology Kharagpur, Kharagpur 721302, India; ‡ Department of Chemistry, 34973 Korea University, Seoul 02841, Korea; § Department of Chemistry, Organic Chemistry Section, 30167 Jadavpur University, Kolkata 700032, India

## Abstract

NIR light-activated photocage with inherent protein tagging ability is unprecedented in contemporary photochemistry. Herein, we introduce a series of protein-taggable NIR-photocages derived from green fluorescent protein (GFP) chromophore analogs with spatiotemporal control for releasing the caged bioactive molecules. Through molecular engineering of the GFP chromophoric scaffold, a series of meso-substituted oxazolone-photocages (*meso*-GFP-PRPG) were judiciously designed and synthesized. These photocages, anchored with electron-donating groups (EDG) and electron-withdrawing groups (EWG), accommodate diverse payloads, including aliphatic carboxylic acids, expanding the possibilities for tailoring their properties and applications. Notably, under anaerobic conditions, irradiation of *meso*-GFP-PRPG leads to fast and efficient release of caged molecules. Insightful experimental and theoretical investigations revealed that photorelease is predominantly driven by the triplet state photochemistry in anaerobic conditions. The concept’s theranostic potential was demonstrated by the conditional release of valproic acid, a neuroprotective agent for Alzheimer’s disease (AD) treatment. *meso*-GFP-PRPG (**15E**) showed enhanced NIR emission with Aβ oligomers and fibrils (30–37 fold vs ThT) and effectively degraded amyloid fibrils under 640 nm light, offering a promising targeted treatment approach for neurodegenerative disorders.

## Graphical abstract

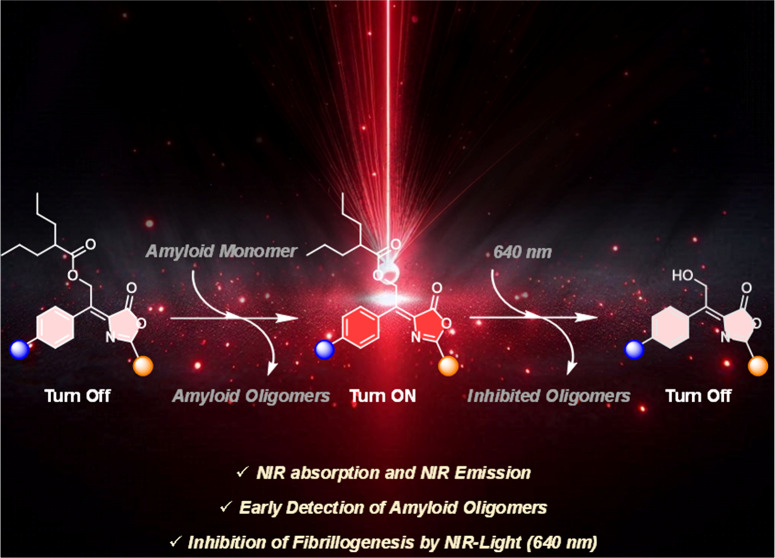

## Introduction

The advent of photoactivatable protecting groups (PRPGs) facilitates spatiotemporal control over the release of bioactive molecules in biological systems and has transformed the landscape of molecular biology and biomedical research. In recent years, an impressive collection of photoactivated linkers, including those based on *o*-nitrobenzyl,^1^ aryl methyl,^2^ and coumarin-4-ylmethyl,^3^ have been utilized to explore various biological processes.^4–6^ These photocages have proven effective in releasing molecules such as cell cycle regulators,^7^ nucleotides,^8^ neurotransmitters,^9^ ions,^10^ small gaseous molecules,^11–13^ and various bioactive molecules.^14^ Regretfully, most photocages are absorbed in the cell-damaging UV region, restricting their further biological applications. To circumvent this limitation, Winter et al. and Stacko et al. reported visible near-infrared (NIR) photocages, including *meso*-methyl BODIPY^15^ and meso-substituted cyanine dyes (Figure 1A).^16,17^ However, none of these chromophores offer inherent protein tagging ability.

**Figure 1:**
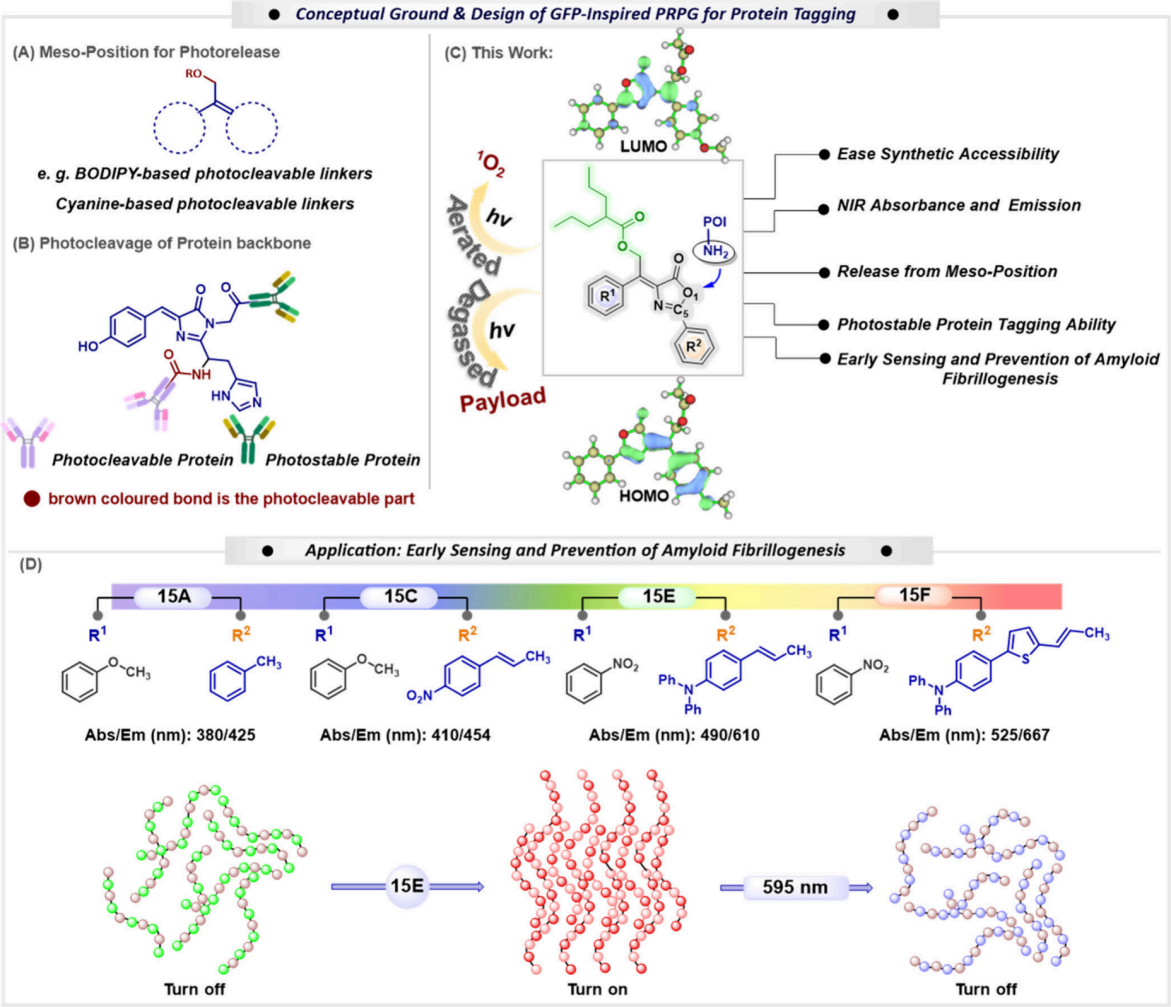
(A) Examples of *meso*-methyl positions for photorelease; (B) photocleavage of coral fluorescent protein: a prior idea for photocleavable and photostable protein; (C) rational design of GFP-core for photocleavage and protein tagging; (D) donor–acceptor complex to achieve red-shifted photocages for early sensing and prevention of amyloid fibrillogenesis.

In this work, oxazolone, a basic structural unit of green fluorescent protein (GFP) chromophore, serves as the primary skeletal structure for PRPGs.^18,19^ The modification of the primary oxazolone motif allows the PRPG to function as a “cage” for a bioactive molecule, while concurrently enabling the tagging of a specific protein of interest. The O_1_ and C_5_ positions of the basic oxazolone core are structurally accessible, making them suitable for protein tagging. Miyawaki group showed that the photocleavage of coral fluorescent protein (green),^20^ undergoes a structural change by β-elimination, which imposes the challenge of protein labeling in living organisms at the C_5_ position of the oxazolone moiety (Figure 1B). Inspired by their pioneering work, the oxazolone ring’s cyclic ester (O_1_) has been substituted with the amide group to tag a photostable protein. In addition, substituting the O_1_ position will help to prevent robust reactivity and retain the structural integrity of these oxazolone moieties toward any other primary amine functionality. Hence, we envisage repurposing the basic GFP chromophore scaffold into the protein-tagged photoactivatable system as *meso*-GFP-PRPG for releasing bioactive molecules (Figure 1C).

To demonstrate the theranostic applicability of *meso*-GFP-PRPG, we focused on a critical area of protein-responsive disease: Alzheimer’s disease (AD). Over the past two decades, myriads of fluorescent probes have been developed to detect amyloid-β (Aβ), the primary protein found in AD pathology.^21^ However, recent research implicates the involvement of Aβ oligomers in AD pathology, due to their potent synaptotoxicity.^22^ While antibody-based enzyme-linked immunosorbent assay (ELISA) offers success in the early diagnosis of AD,^23–25^ problems such as high cost and intricate procedure pose significant obstacles. In recent times, the concept of small molecule design integrating diagnostic and therapeutic functions represents a significant advancement in modern medicine. Although our recent contribution has enabled the successful detection of Aβ oligomers in AD patients,^22a–22c^ efforts to prevent fibrillogenesis remain unexplored, leaving a critical gap in understanding and potentially treating the progression of AD.

Herein, by introducing amphiphilic modifications and π-conjugation to the *meso*-GFP-PRPG, we successfully engineered NIR-light-responsive photocages capable of precisely releasing valproic acid, a well-known neuroprotective drug. **15D-15F** can detect solubilized oligomeric Aβ units, due to the restricted intramolecular rotation of the exocyclic double bond. Upon red light irradiation, the release of valproic acid effectively degrades amyloid fibrils. We believe this work demonstrates a targeted and potentially more effective treatment strategy for AD, manifesting a significant advancement in treating neurological conditions.

## Results and Discussion

A series of meso-substituted GFP chromophore-based photocages have been synthesized to explore different functions: (i) incorporating different substituents, ranging from electron-withdrawing groups (EWGs) to electron-donating groups (EDGs) (Scheme 1a) to monitor the substituent effect on the photochemistry, (ii) exemplified to showcase protein tagging ability either via direct protein tagging or via linker-based protein tagging, and (iii) donor–acceptor conjugate to achieve red-shifted absorptivity and alter the amphiphilicity.

**Scheme 1:**
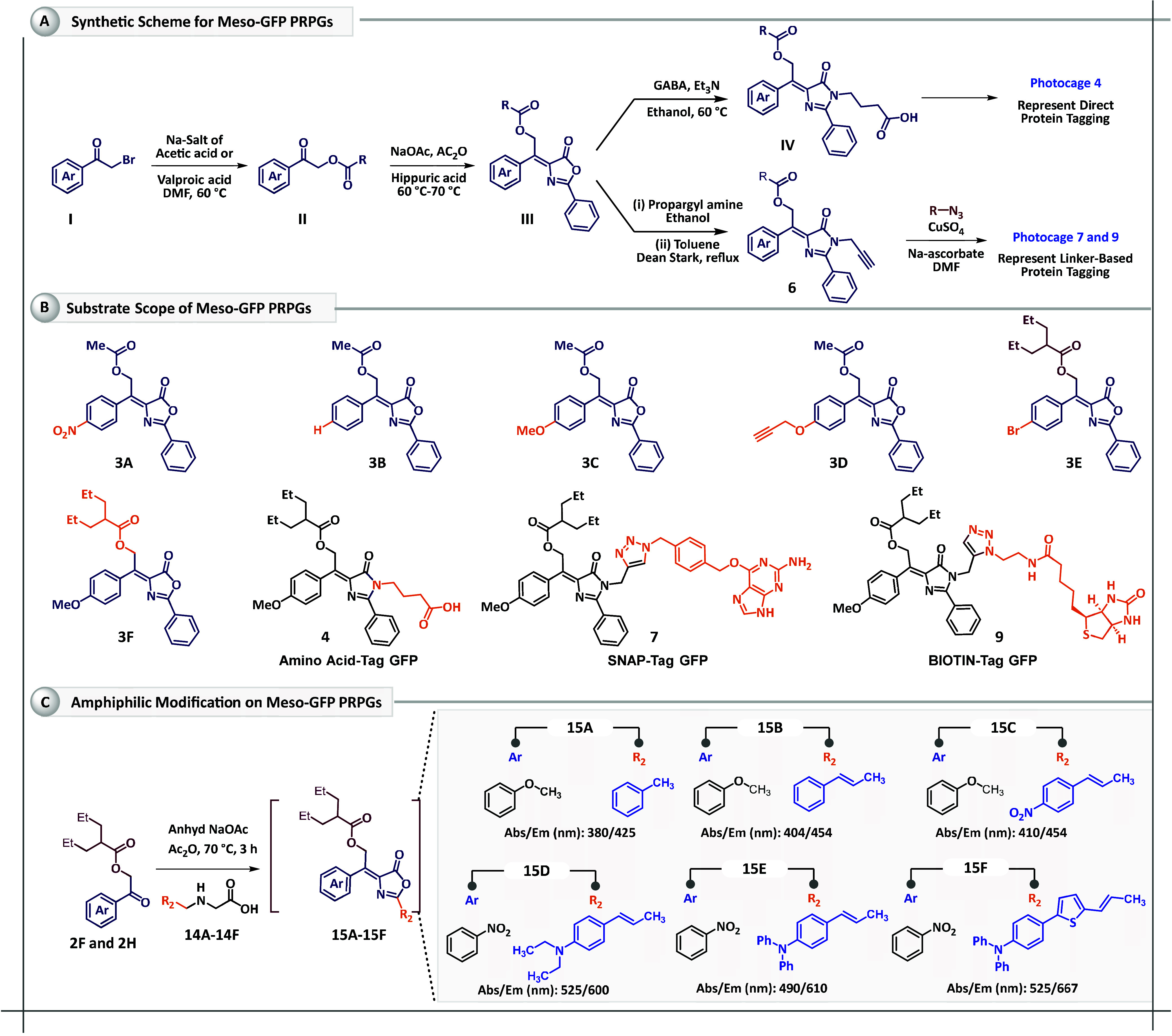
(A) General Synthesis of Meso-Substituted GFP-Photocages, (B) Examples of Meso-Substituted GFP-Photocages, and (C) Synthetic Procedure for Amphiphilic Modification on GFP-Photocages and Examples

The synthesis of *meso*-GFP-based photocages proved to be both straightforward and challenging, as Erlenmeyer condensation, typically works well for aldehydes, but fails for keto functionalities. An ester functionality was introduced at the α-carbon position to enhance the electrophilicity of the keto-carbonyl group. Para-substituted α-ester derivatives (II) were synthesized from their respective α-bromoacetophenone derivatives (I) with yields of 85%. The desired GFP-photocages (III) **3A**–**3F** were obtained by subjecting compound II to an Erlenmeyer condensation reaction, resulting in a mixture of *E*-*Z* isomers, 8–10% yield (Scheme 1b).

To demonstrate the direct protein tagging capability of *meso*-GFP-PRPG, we synthesized GFP-photocage **4**, by incorporating the nonproteinogenic amino acid GABA into the *meso*-GFP scaffold following the reported procedure (Schemes 1a and S4). Additionally, we selected two established linkers for linker-mediated protein tagging: (i) SNAP-linker^26,27^ and (ii) Biotin-linker,^28^ for compatibility with SNAP-tagging and avidin-targeted drug delivery protocols, respectively. The linker-modified GFP-photocages (7 and 9) were synthesized via click reaction between photocage 6 and their respective azide compounds (6f and 8, Scheme S2).

For the synthesis of NIR-responsive *meso*-GFP-PRPGs, we adapted our previously established synthetic protocol using OMe-α-ketoester (**1**) and hippuric acid derivative (compound 2). We attempted to extend the π-conjugation from compound **15A** using anhydrous ZnCl_2_.^29^ However, we observed hydrolysis of ester functionality instead. Consequently, the hippuric acids were modified to their π-extended versions (**14A**–**14F**), and the final NIR-responsive *meso*-GFP-PRPGs (**15A**–**15F**) were synthesized through Erlynmeyer condensation using NaOAc and acetic anhydride (Scheme 1c). The products were characterized thoroughly by NMR and HRMS as shown in Figures S2–S58.

The photophysical characterizations of the GFP-photocages were performed in different solvent systems. Depending on the substituents at the C4 position, GFP-photocages showed a red-shifted absorption from EWG to EDG. GFP-photocage 3A exhibited a maximum absorption at 330 nm, while **3B** and **3C**–**3F** had maxima at 344 nm, and up to 380 nm, respectively, corresponding to their n-π* transitions (Figure 2a and Figures S61-S63). In contrast, photocages **15E** and **15F** showed broad UV–vis spectra with absorption maxima (λ_max_) at 490 and 525 nm, extending up to 580–630 nm, respectively, demonstrating their applicability for photouncaging within the NIR window (Figure 2a). To mimic the acidic conditions of various diseases, including Alzheimer’s disease (pH of 5.3–7), we monitored the UV–vis spectra of all photocages at different pH conditions. Notably, a red-shifted absorption maximum was observed at low pH due to the protonation of the ‘N’ atom of the oxazolone. Indeed, photocage 15F showed λ_max_ at 563 nm in pH 3 (Figure S67).

**Figure 2:**
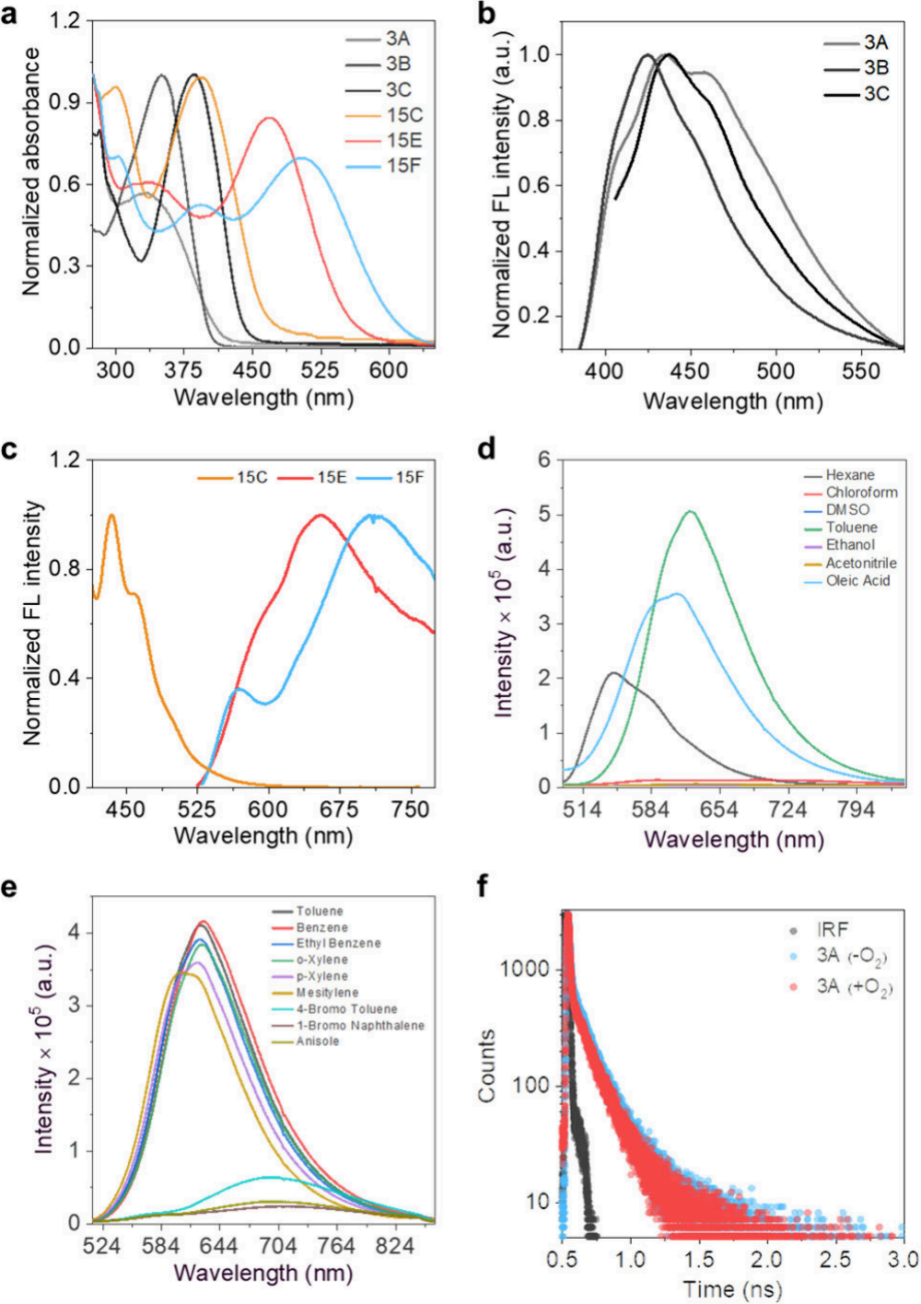
(a) Normalized UV–vis of photocages **3A**, **3B**, **3C**, **15C**, **15E** and **15F** in chloroform (1 × 10^–5^ M); normalized emission spectra of (b) **3A**, **3B**, and **3C** (c) **15C**, **15E**, and **15F** in chloroform (1 × 10^–5^ M); (d) solvent-dependent emission spectra of photocage **15E** (1 × 10^–5^ M); (e) change in the emission spectra of photocage **15E** in different aromatic solvents; (f) change in the singlet state lifetime of photocage **3A** in the presence and absence of oxygen.

Fluorescence emission studies revealed that GFP-photocage 3B displayed the most blue-shifted emission maximum at 425 nm, whereas others showed an emission ranging from 439 to 450 nm (Figure 2b). The photocages’ fluorescence quantum yield (Φ^F^) varies from 0.004 to 0.16. Although photocages **6** and **3D** had significantly longer excited-state lifetimes (approximately 2 and 1.6 ns, respectively) compared to **3C** (0.024 ns), the fluorescence quantum yield of **3C** was two times higher. This could be attributed to the frequent bond rotation of the propargyl moiety, leading to rapid nonradiative decay.

Solvent-dependent spectrophotometric analyses revealed that photocages **15E** and **15F** exhibited red-shifted emission maxima at 624 and 667 nm, respectively, with large Stokes shifts of 134 and 142 nm (Figure 2c). In viscous and nonpolar aromatic solvents, a substantial increase in fluorescence intensity has been noticed, specifically 45-fold for **15E** and 20-fold for **15F**, suggesting a strong correlation between solvent viscosity and observed emission. (Figure 2d). Interestingly, the emission intensity was significantly reduced in the case of aromatic solvents with bulky substituents, suggesting π-stacking (Figure 2e). Enhanced emission intensities were corroborated by singlet-state lifetime measurements in polar and nonpolar solvents (Figure 2f).

Next, photorelease properties of GFP-photocage were investigated using a 125 W medium-pressure Hg-lamp (≥410 nm, with a 1 M NaNO_2_ cutoff filter). Photolysis was monitored under both aerated and argon-purged conditions using reverse-phase HPLC (RP-HPLC) (Figure 3a). Under aerated conditions, 30% photodissociation of photocage 3C was achieved within 6 h. In contrast, degassed conditions led to 85% release within 1.5 h. RP-HPLC showed a decrease in the intensities of *E* and *Z* isomer peaks (**3C** and **3C′**) at 5.8 and 5.2 min, respectively, and the formation of new photoproducts at 3.9 min (Figure 3b). We also examined the photorelease of acetic acid in ACN-water systems (7:3 and 1:1 v/v), showing a faster release rate at higher water percentages, likely due to restricted intramolecular motion (Table 1).

**Figure 3:**
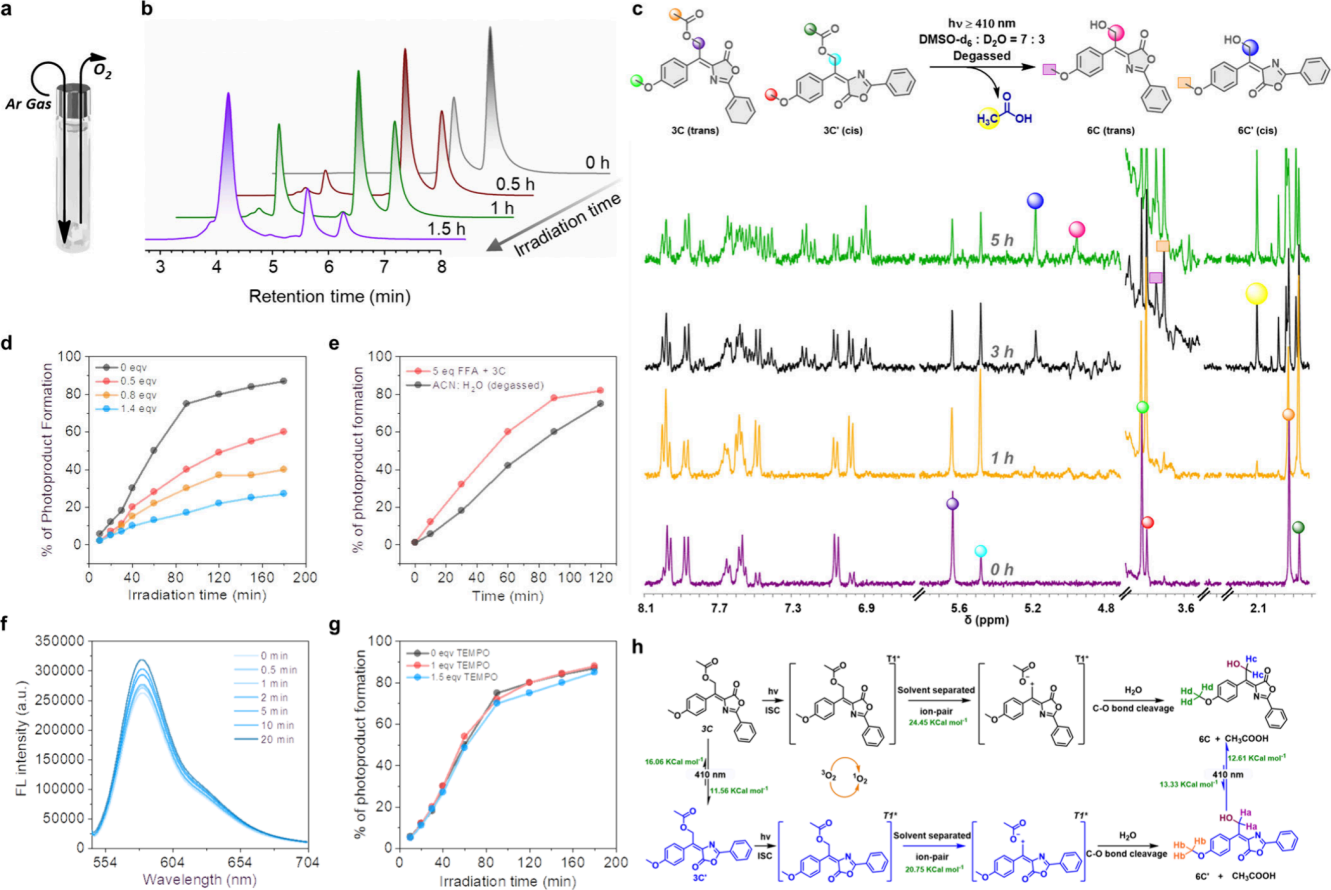
(a) Pictorial diagram of the experimental setup in degassed condition; (b) RP-HPLC diagram of the course of the photolysis of photocage **3C**; (c) ^1^H NMR study for monitoring the course of the photolysis [photocage **3C** (1 mg) in 0.4 mL DMSO-*d*_6_ + 0.1 mL D_2_O, degassed]; (d) photorelease of photocage **3C** with dose-dependent triplet state quencher, potassium sorbate (1 mg of photocage **3C** in 7:3 ACN: H_2_O, degassed, ≥410 nm); (e) photorelease of photocage **3C** in the presence of singlet oxygen scavenger, furfuryl alcohol (**3C** + furfuryl alcohol 1:10, aerated ethanol 10 mL); (f) change in emission spectra of SM-94 after sensing of singlet oxygen (**3C** + fluorescence reporter SM-94 1:1); (g) photolysis of photocage **3C** with dose-dependent radical scavenger, TEMPO (1 mg of photocage **3C** in 7:3 ACN: H_2_O, degassed, ≥410 nm); (h) probable photorelease mechanism of GFP-photocage **3C** (*cis*-*trans*).

**Table 1:**
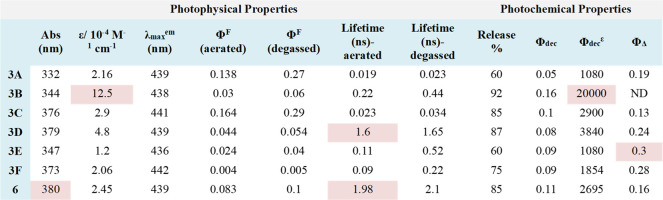
Photophysical and Photochemical Properties of GFP-Photocages^a^

a Abs: represents absorption maxima; ε: represents molar extension coefficient; λ_max_^em^ represents emission maxima; Φ^F^ represents fluorescence quantum yield; Φ_dec_ represents dissociation quantum yield; Φ_Δ_ represents the singlet-oxygen generation quantum yield. All the errors limits are within ± 0.003.

Photolysis was further monitored using ^1^H NMR (Figure 3c). Key observations included three noticeable changes in the NMR spectra. (i) Release of caged acid: There is a gradual increase in a new resonance peak at 2.1 ppm corresponding to the released acetic acid. (ii) Photoproduct formation: Unlike the HPLC profile (single peak), we found a concomitant increase in two sets of new peaks at 3.7–3.75 ppm and 5.0–5.2 ppm, which corresponds to the OMe-group and CH_2_–OH of the newly formed *Z*-*E* photoproducts (**6C** and **6C′**), respectively. (iii) Isomerization of the starting material: Along with the photoirradiation time, there is an interconversion of resonance peaks between 5.4 and 5.6 ppm (corresponds to the CH_2_ of the ester functionality) and 3.8 and 3.85 ppm (corresponds to the OMe-group) of the photocages (3C and 3C′), indicating *Z*-*E* isomerization under photoirradiation condition. In addition to the above findings, it was well pointed out that the formation of the trans-photoproduct was slightly higher than the *Z*-photoproduct. The newly generated alcohol-based photoproducts (**6C** or **6C′**) were confirmed by HRMS (Figure S78). Similar photorelease behavior was observed for photocages **3A** and **3B**, as indicated by time-dependent RP-HPLC (Figures S73a and S75a), NMR (Figures S73c and S75c), and HRMS (Figures S74 and S76).

To investigate the role of the attached substituents in the photorelease process, we conducted independent photorelease studies of GFP-photocages. Photocage 3B exhibited the highest photorelease with a quantum efficiency (Φ_dec_^ε^) of 20,000, followed by OMe-GFP (3C), and NO_2_-GFP (3A). The trend in the Φ_dec^ε^_ aligned well with the molar absorptivity coefficient (*ε*) of the corresponding photocages. For photocages **3D** and **6**, the photochemical quantum yields (Φ_dec_) were comparable to those of **3C**, as summarized in Table 1.

After successfully demonstrating the photouncaging of GFP-photocages, we turned our attention to illuminating the precise photorelease mechanism. The oxygen-dependent photorelease of GFP-photocage **3C** led us to believe that the triplet state chemistry might be responsible for the release mechanism. To validate this, we performed the photolysis of GFP-photocage **3C** (1 mg in 10 mL 7:3 ACN/water mixture) in the presence of a triplet state quencher, potassium sorbate (0–1.4 equiv). We observed that as the concentration of the quencher increased, the rate of release decreased concurrently with each half-hour increment (Figure 3d). This result indicated that energy transfer from the triplet state of **3C** to potassium sorbate competes with the photorelease process. Additionally, the photoreaction of **3C** proceeded with a similar photorelease rate in the presence of a singlet oxygen (^1^O_2_) scavenger, furfuryl alcohol (Figure 3e).

To evaluate the efficiency of GFP-photocages in producing singlet oxygen under aerobic conditions, we used a rhodamine-anthracene (SM-94) conjugate-based fluorescence reporter rather than the regular DPBF, as the rapid degradation of DPBF encounters errors in a very short time frame. The synthesized fluorescence reporter was previously used only to detect chemically generated.^30^ Therefore, this reporter was successfully employed to determine photochemically generated by GFP-photocages, yielding a promising result (Figures 3f and S81) of up to 40 min of photoirradiation. The quantum yields (Φ^Δ^) for the generation of of GFP-photocages were calculated with respect to phenalenone (which provides a separate absorption and fluorescence property than the sensor),^31^ and results are summarized in Table S1. GFP-photocage 3E showed the highest generation ability (Φ^Δ^) of 0.3 as compared to the other GFP-photocages due to the presence of heavy-atom (resulting in high spin–orbit crossover) on the oxazolone moiety.

It is well-known that the photorelease mechanism can be driven either by a radical pair or by a solvent-separated ion pair.^4^ To figure out the excited state chemistry, we carried out the photolysis of photocage 3C (degassed, ACN:H_2_O = 7:3) in the presence of a dose-dependent radical scavenger, TEMPO (2,2,6,6-Tetramethyl-1-piperidinyloxy), and monitored the photodegradation phenomena using HRMS analysis. No trace of TEMPO-adducted photoproduct was observed in the HRMS. Also, no significant change in the rate of the photodissociation process was noticed, in the presence and absence of TEMPO (Figure 3g).

The photodissociation of photocage 3C was further carried out in dry ethanol (degassed, ≥410 nm, 3 mg in 10 mL ethanol) medium. From the photorelease profile, it is evident that the kinetics of the foundation of a new photoproduct take place at a faster rate in a nucleophilic solvent as compared to ACN:H_2_O, suggesting an S_N_1-type process via the formation of a *meso*-methyl carbocation intermediate like BODIPY-based PRPG.^32^ Furthermore, analysis of the photolyzed mixture via HRMS affirmed that the newly formed peak at 338.1383 (*m*/*z*) corresponds to the ethoxy ether-based photoproduct (Figure S79). Therefore, the observed formation of the ethoxy-ether-based photoproduct and the absence of the TEMPO-adduct photoproduct, strongly indicate the occurrence of solvent-separated ion-pair formation, ultimately resulting in the formation of solvent-captured photoproduct.

Theoretical investigations using TD-DFT calculations in ORCA 5.03,^33^ showed a notable disparity in the isomerization barriers between **3C** and **3C′**. Specifically, the transition from **3C** to **3C′** exhibited a significantly lower barrier, approximately 5 kcal/mol, compared to the reverse isomerization process from **3C′** to **3C**. This disparity, graphically depicted in Figure S83 through the potential energy surface, substantiates the rapid isomerization observed from 3C to 3C′ reactant. Moreover, our findings indicated comparable barriers for the isomerization between **6C** and **6C′**, as well as **6C′** and **6C**, suggesting the coexistence of both products at equilibrium.

The validation of the triplet-state-driven photochemistry is further supported by the discernment of spin density distributions and nonzero spin–orbit coupling (SOC) values. The presence of substantial spin density, along with SOC values, underscores the key role of these factors in facilitating favorable ISC. This compelling evidence supports the photodissociation process via the triplet state. Our investigations suggest an ISC deactivation pathway through the S1-T2 channel, supported by a minimal energy gap of 0.1 eV (Figure S84 and Scheme S4).

Prior to utilizing the NIR-GFP-photocage for fluorescence marking of Aβ oligomers, two key factors were carefully examined: (i) systematic characterization of amyloid fibril formation from Aβ_40_ using different techniques such as TEM, FT-IR, and circular dichroism (CD) (Figure S90), and (ii) assessment of the emission properties of photocages **15A**–**15F** in aqueous media. Following the successful characterization of Aβ oligomers, each of the photocages **15A**–**15F** was incubated with the fibril solution. Photocages **15D**–**15F** exhibited a positive response (Figure S91 (v)-(vii)), whereas photocages **15A**–**15C** did not show significant changes in their emission profiles (Figure S91 (i)-(iv)), although they did show a positive fluorescence response in more viscous media.

Further investigation was conducted into the sensing capabilities of **15E** for both soluble Aβ oligomers and insoluble Aβ fibrils. Notably, photocage **15E** exhibited remarkable fluorescence enhancement, demonstrating a 37-fold increase in the presence of Aβ fibrils and a 30-fold increase in the presence of Aβ oligomers (Figure 4a), significantly outperforming the conventional fluorescence marker ThT, which only showed only a 5-fold enhancement (Figure S91 (ix)). In addition, photocage **15E** exhibited high binding affinity (*K*_D_ = 478 ± 0.75 nM) and high sensitivity toward Aβ_40_ oligomers (LOD = 0.23 ± 0.79 μM) and Aβ-40 fibrils (LOD = 1.2 ± 0.79 pM) during the process of amyloid fibrillogenesis. The impressive signal-to-noise ratio of photocage **15E** encouraged us to investigate its potential for monitoring the intermediate multimeric forms between the Aβ monomer and oligomer. Time-dependent fluorescence analysis employing **15E** illuminated a gradual augmentation in emission intensity, as depicted in Figure 4b. The fluorescence increment was well corroborated by the singlet state lifetime, as measured by time-correlated single photon counting (TCSPC). The presence of Aβ species, including monomers, oligomers, and fibrils, led to an increase in the singlet state lifetime of photocage **15E** from 1.58 to 3.49 ns (Figure S92­(ii)). To ensure the accuracy of the fluorometric assays, which can potentially distort the structure of amyloid fibrils, the structural integrity of Aβ40 fibrils was assessed by monitoring their secondary structure using CD spectrometry in the presence of different concentrations of photocage **15E** (Figure S92­(iii)). Despite the increased emission intensities of **15E**, the fibril structure remains unchanged, confirming the preservation of the helical content. Additionally, the comparison between **15E** and the gold standard ThT distinctively reflects that **15E** shows a 5-fold increase in the emission intensity, helping to detect the aggregated Aβ40 in the early incubation period as compared to ThT (Figure 4c). The selectivity toward Aβ40 was further assessed in comparison to other analytes, and the emission response depicts complete selectivity of **15E** toward Aβ40 (Figure 4d). Collectively, these results suggest the rapid responsiveness, high definition, and super selectivity of photocage **15E** toward Aβ40 as compared to ThT.

**Figure 4:**
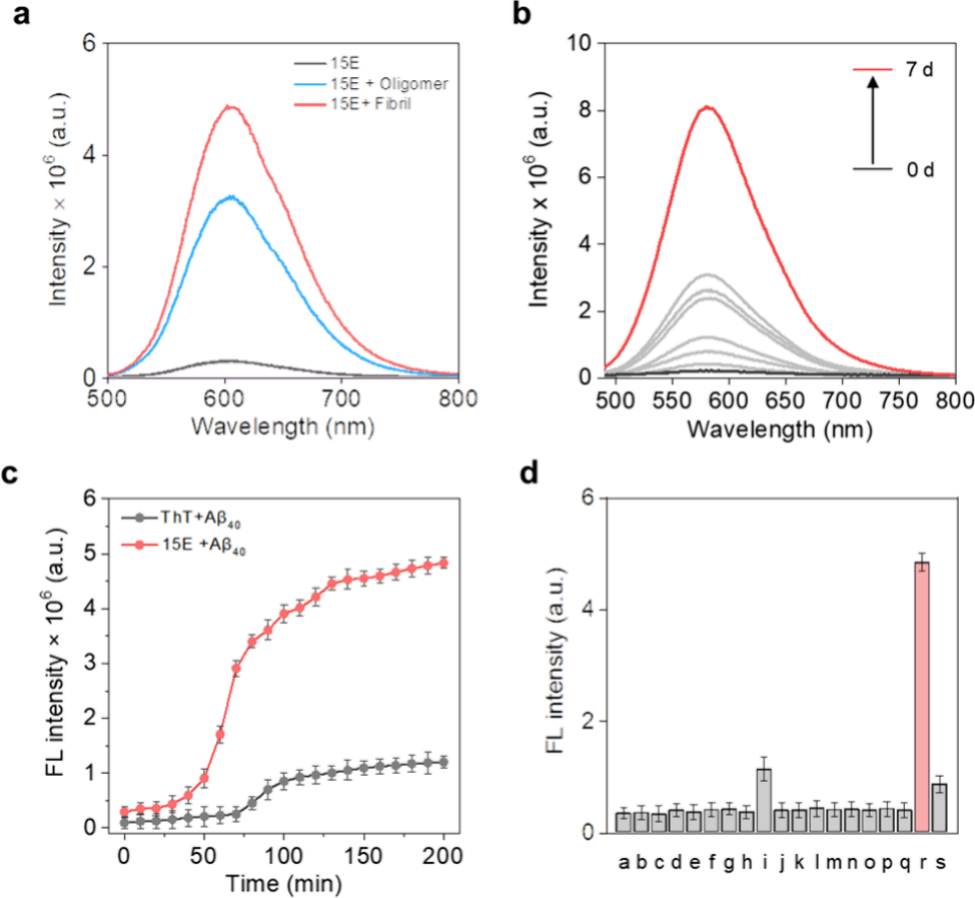
(a) Change in the emission spectra of photocage **15E** in the presence of Aβ_40_ oligomer and fibril; (b) day to day emission spectra of **15E** along with fibrillogenesis; (c) monitoring of Aβ_40_ protein aggregation kinetics with **15E** and thioflavin-T; (d) emission response of photocage **15E** in the presence of different analytes; a: Gly; b: Arg; c: Lys; d: Leu; e: Gln; f: Thr; g: Cys; h: Tyr; i: insulin; j: Na^+^; k: K^+^; l: Ca^2+^; m: NO^+^; n: H_2_O_2_; o: ClO^–^; p: TBHP; q: PBS; r: Aβ40; s: BSA.

To evaluate the efficacy of photocage **15E** in preventing fibrillogenesis, photolysis of photocage **15E** was conducted in a deoxygenated solution of ACN-water mixture (7:3, degassed, 1 × 10^–5^ M) under irradiation at various wavelengths. Under 500 ± 10 nm green light, 80% of the caged valproic acid was uncaged, similar to the results when irradiated under 525 ± 10 nm green light (Figure 5a). A similar trend was observed when photocage **15E** was irradiated in solutions with varying pH values. Notably, photocage **15E** was able to release up to 70% of caged valproic acid at pH 7.5 (525 ± 10 nm) and 60% at pH 3 when irradiated with 640 ± 10 nm red light, both within 90 min of photoirradiation (Figure 5b). These results indicate that the efficiency of photoconversion may not have a direct relationship with the absorptivity of the compound, consistent with observations from previously reported systems.^34^

**Figure 5:**
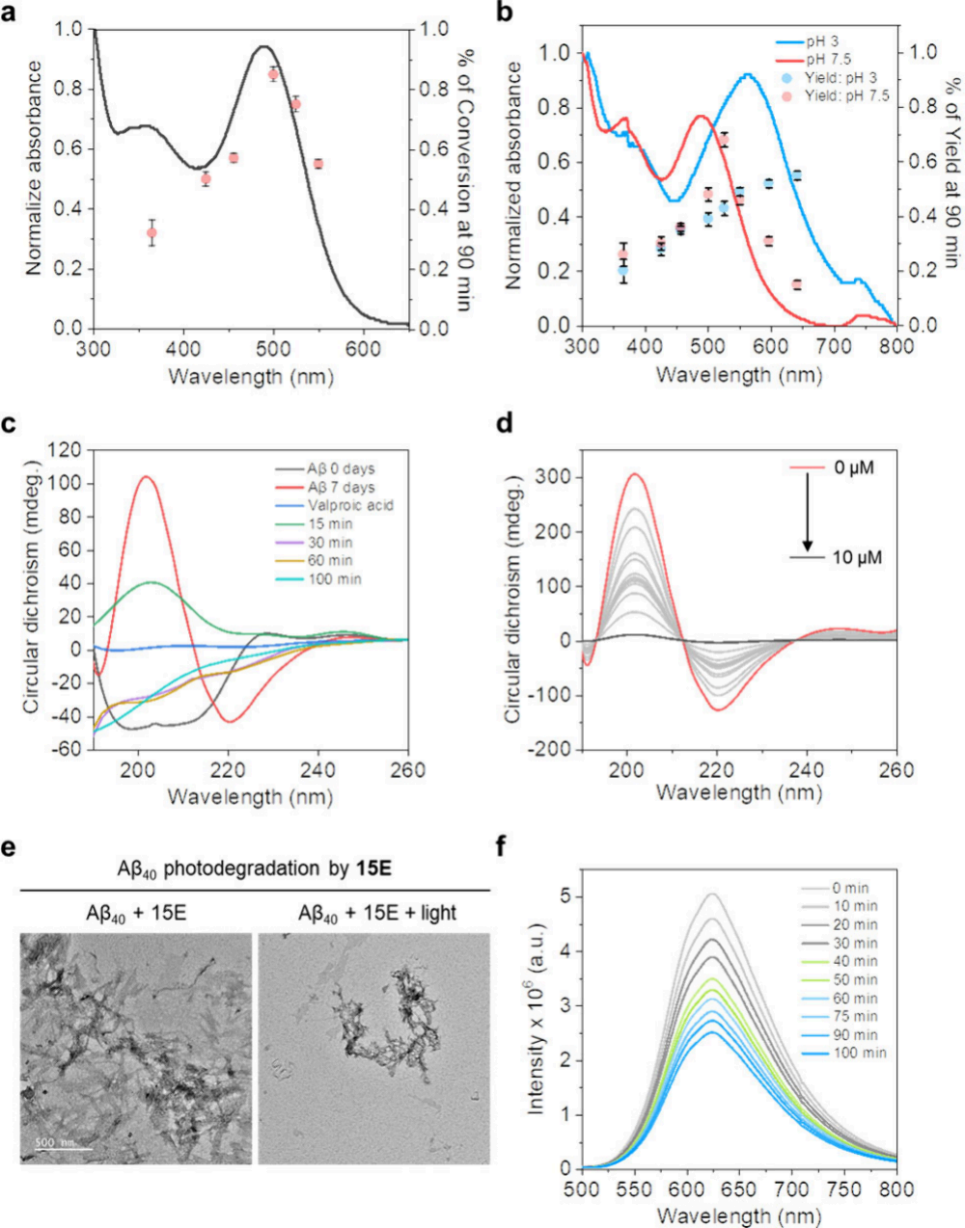
(a) Wavelength-specific photorelease of **15E** and percentage of photoconversions (7:3 ACN: H_2_O, degassed, 1× 10^–5^ M); (b) wavelength-specific photorelease of **15E** and percentage of photoconversions in different pH solutions (1.5 mL ACN + 1.5 mL pH solutions, degassed, 1× 10^–5^ M); (c) change in the CD spectra of Aβ_40_ fibril after photoirradiation with green light (525 ± 10 nm); (d) change in the CD spectra of Aβ_40_ fibril in different concentrations of valproic acid; (e) TEM image of Aβ_40_ fibril before and after the photoirradiation (525 ± 10 nm); (f) photolysis of **15E** (10 μM) by 595 ± 10 nm light after sensing the amyloid oligomers.

To further examine the ability of photocage **15E** in inhibiting fibrillogenesis, we incubated Aβ_40_ with **15E** in four separate sets, and carried out photolysis (595 ± 10 nm) at different time intervals of 15, 30, 60, and 120 min, followed by the incubation of the sample for 7 days. CD spectrometry results revealed that increasing the irradiation time led to a gradual inhibition of Aβ_40_ fibril formation, ultimately resulting in a random coil structure after being irradiated for 120 min. This observation was consistent with valproic acid-treated fibrils (Figure 5c,d). TEM analysis showed a broader amorphous structure with fewer fibrils after photolysis with green light (Figure 5e). These findings were further validated by ThT assays, which demonstrated a gradual decrease in the ThT emission intensity with increased irradiation time (Figure 5f). Taken together, the studies suggest that photocage **15E** possesses a remarkable dual functionality, not only senses Aβ oligomers but effectively prevents fibril formation upon exposure to red light.

Given the efficient theranostic performance of **15E**, we further investigated its practical application by conducting a cell viability assay on human neuroblastoma SH-SY5Y cells under both dark and photoirradiated conditions (Figure S93a). The half-maximal inhibitory concentration (IC_50_) of photocage **15E** was 1.7 μM under dark conditions, which significantly decreased to 0.75 μM upon photoirradiation. To evaluate Aβ-induced neurotoxicity and fibrillogenesis, we examined changes in cell morphology, damage, and dysfunction in SH-SY5Y cells. After 48 h of incubation with Aβ40, the cells exhibited pronounced neurotoxicity, membrane rupture, and cell death, along with the accumulation of aggregated Aβ40 fibrils in the extracellular region. Staining the cells with both photocage **15E** and ThT revealed colocalization of the two markers with a Pearson’s coefficient of 0.92, highlighting the potential of photocage **15E** as an effective tool for detecting amyloid fibrils (Figure S93b-f).

## Conclusions

In conclusion, we reported a series of meso-substituted oxazolone-photocages (*meso*-GFP-PRPG) with tunable photophysical and photochemical properties, enabling simultaneous protein tagging and controlled bioactive molecule release. The direct protein tagging capability of *meso*-GFP-PRPG has been successfully showcased through the integration of nonproteinogenic amino acid GABA, SNAP-linker, and Biotin-linker into the *meso*-GFP scaffold. Spectrophotometric analyses have firmly established that the *meso*-GFP-PRPGs undergo a faster photouncaging process under anaerobic conditions, paving the way for spatial and temporal control over the release of caged compounds. Theoretical calculations confirmed that the oxygen-dependent photorelease of GFP-photocage is governed by triplet state chemistry. In combination with the facile synthesis and modularity of the GFP scaffold, our best candidate, i.e., **15E**, exhibited remarkable NIR emission upon interaction with Aβ oligomers and fibrils with an impressive LOD of 1.2 pM. In addition, the efficient release of valproic acid from photocage **15E** under 640 nm light irradiation manifests its potential for diagnostic as well as therapeutic capabilities in the NIR region. The photorelease process with *meso*-GFP-PRPG led to the effective degradation of amyloid fibrils, suggesting a promising avenue for light-controlled therapeutic interventions in neurodegenerative disorders. We believe this study illuminates a promising direction for advancing the field of photochemistry through the development of novel PRPGs, while simultaneously unlocking new avenues for the diagnosis and treatment of Alzheimer’s Disease.

## Supplementary Material



## Notes

Experimental procedures, NMR spectroscopy and mass spectrometry characterization data for new compounds, and details of the photolysis and the experimental evidence (PDF)
