# Supplementary material for: Engineering a Green Fluorescent Protein-Core-Inspired NIR-Photocage: Exploring meso-GFP-PRPG toward Alzheimer’s Disease Therapeutics
Source: ACS Cent Sci. 2025 Mar 20;11(7):1062–70. doi: 10.1021/acscentsci.5c00027 (PMC12291143; doi:10.1021/acscentsci.5c00027)
Supplement: Supplementary file 1 [file oc5c00027_si_001.pdf]

Engineering a GFP-Core-Inspired NIR-Photocage: Exploring meso-GFP-PRPG Towards Alzheimer's Disease Therapeutics

*Saugat Mondal[a‡], Jusung An[b‡], Tapas Bera[c], Moumita Banerjee[a], Snehasish Debnath[b], Debasish Mandal[a], Antara Sikder[a], Samit Guha[*c], Jong Seung Kim[*b], and N. D. Pradeep Singh[*a]*

a Department of Chemistry, Indian Institute of Technology Kharagpur, Kharagpur 721302

b Department of Chemistry, Korea University, Seoul 02841, Korea

c Department of Chemistry, Organic Chemistry Section, Jadavpur University, Kolkata 700032, India

Supporting Information

Sl No	Contents	Page No
**1.**	General Experimental Details	S3
**2.**	Failure Reactions	S3
**3.**	Synthesis of GFP-photocages, phenolenone, fluorescence reporter SM-94	S3-S14
**4.**	Characterization of GFP-Photocages (^{1}H, ^{13}C, HRMS)	S15-S59
**5.**	Photophysical Characterizations of the GFP-Photocages	S59-S65
**6.**	Determination of Photon Flux (I_0) and Specification of Light Sources	S66-S67
**7.**	Photochemical Characterizations of the GFP-Photocages	S68-S70
**8.**	Quantification of Singlet Oxygen	S71
**9.**	TD-DFT Calculations	S72-S75
**10.**	Fluorescence Response in Presence of Bio-molecules	S76
**11.**	Experimental Details of Cell Experiments	S77-S80
**12.**	Co-ordinates Used in DFT and TDDFT Calculations	S81-S98
**12a.**	References	S98-S99

1. General experimental details:

Glassware used in reactions was thoroughly oven-dried. All commercial-grade reagents were used without further purification and solvents were dried prior to use following standard protocol. Reactions were monitored by thin layer chromatography (TLC) using Merck silica gel 60 F254 pre-coated plates (0.25 mm), and the spots were visualized by exposure to UV light and/or by dipping into $KMnO_4$ solution. Silica gel of particle size 230–400 mesh and petroleum ether/ethyl acetate as eluent was used for column chromatographic purification. ^{1}H and ^{13}C NMR spectra for all the compounds were recorded at 400/600 and 100/150 MHz (Bruker UltrashieldTM 400, AscendTM 600), respectively. The spectra were recorded in deuterochloroform ($CDCl_3$), deuterated acetonitrile (CD_3CN), deuterated dimethyl sulfoxide (DMSO-d_6), and deuterated water (D_2O) as solvent at room temperature. Chemical shifts are reported in ppm from tetramethylsilane with the solvent resonance as internal standard ($CDCl_3$: $\delta_H = 7.26$, $\delta_C = 77.16$ ppm and CD_3CN: $\delta_H = 1.94$, $\delta_C = 1.32$, 118.26 ppm). Data for ^{1}H NMR are reported as follows: chemical shift (δ ppm), multiplicity (s = singlet, d = doublet, t = triplet, m = multiplet, dd = doublet of doublet q: quartet, dt: doublet of triplets, br: broad.), coupling constant (Hz), integration. Data for ^{13}C NMR are reported as chemical shifts. UV/vis absorption spectra were recorded on the Shimadzu UV-2405 UV/vis spectrophotometer and Shimadzu UV-2600 UV/vis spectrophotometer. Fluorescence emission spectra were recorded on the Hitachi F-7000 fluorescence spectrophotometer and Shimadzu RF-6000 spectrofluorophotometer. HRMS spectra were recorded on a JEOL-AccuTOF JMS-T100L mass spectrometer. Photolysis was carried out using a 125 W medium-pressure mercury lamp supplied by SAIC (India).

2. Failure Reactions:

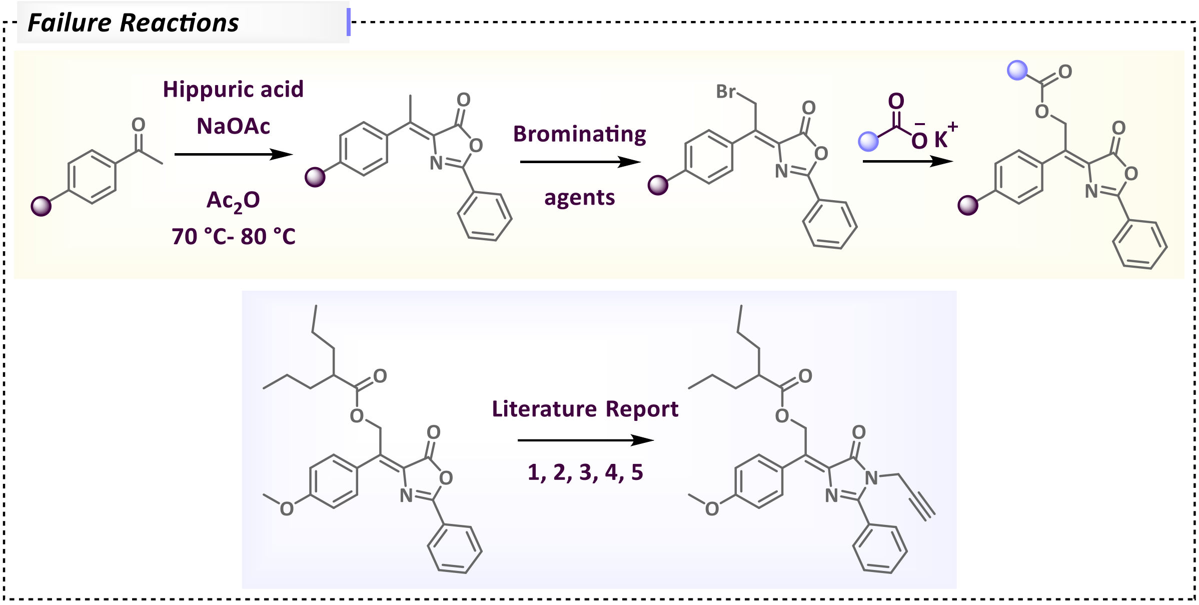

Scheme S1: Synthetic scheme of the failure reactions.

3. Synthesis of GFP-Photocages:

3. 1. General Synthesis of Compound I:

A solution of appropriately substituted acetophenone (12 mmol) in 80 ml of $CHCl_3$ was added in one portion to a vigorously stirred, refluxing suspension of (25 mmol) of $CuBr_2$ in 65 ml of Ethyl acetate. The reaction was practically completed after 3 h, with the indication of a white precipitate of CuBr. The reaction mixture was filtered with 75 ml of ethyl acetate, and the workup was done with saturated $NaHCO_3$, dried over Na_2SO_4, and the mixture was purified by 100-200 mesh silica gel with 10% EtOAc: PET ether (V: V) to obtain the pure mono-bromo compounds **1A**, and **1C**.

1A: (*p*-nitrophenacyl bromide) yield 48%

^{1}H NMR (400 MHz, $CDCl_3$): δ 8.35 (d, *J* = 8.2 Hz, 1H), 8.16 (d, *J* = 8.5 Hz, 1H), 4.45 (s, 1H).
^{13}C NMR (126 MHz, $CDCl_3$) δ 190.0, 150.8, 138.5, 130.2, 124.1, 77.4, 77.2, 76.9, 30.3.

1B: Was directly purchased from Spectrochem.

1C: (*p*-methoxyphenacyl bromide) yield 48%
^{1}H NMR (500 MHz, $CDCl_3$): δ 7.94 (d, *J* = 8.9 Hz, 2H), 6.93 (d, *J* = 8.9 Hz, 2H), 4.38 (s, 2H), 3.85 (s, 3H).
^{13}C NMR (126 MHz, $CDCl_3$): δ 190.0, 164.2, 131.4, 127.0, 114.1, 77.4, 77.2, 76.9, 55.6, 30.8.

1D: Was directly purchased from BLD-Pharm
1E: Was directly purchased from BLD-Pharm

3. 2. General Synthesis of Compound II:

In dry DMF solvent (5-10 ml) appropriate acid (RCOOH, 1.2 eq) was dissolved and stirred for 30 min at 0 °C, $NaHCO_3$ (1.2 eq) was added to the above mixture and also stirred for 1 h to generate the corresponding anion. Compound **1** (1 eq) was then added to it at room temperature. The reaction was monitored by checking TLC. After completion of the reaction, it was quenched with brine solution and worked up with saturated bicarbonate solution. A Flash column was done to get the pure compounds **2A**, **2B**, **2C**, **2D**, and **2E,** along with a yield of ~92%.

2A:
^{1}H NMR (500 MHz, $CDCl_3$) δ 8.31 (d, *J* = 8.8 Hz, 2H), 8.07 (s, 2H), 5.32 (d, *J* = 3.9 Hz, 2H), 2.21 (dd, *J* = 6.0, 4.6 Hz, 3H).
^{13}C NMR (126 MHz, $CDCl_3$) δ 191.24, 170.41, 150.78, 138.69, 129.02, 124.16, 77.43, 66.17, 20.51.

2B:
^{1}H NMR (400 MHz, $CDCl_3$) δ 7.89 (d, *J* = 8.1 Hz, 2H), 7.66 – 7.54 (m, 1H), 7.46 (t, *J* = 7.7 Hz, 2H), 5.32 (s, 2H), 2.28 – 2.15 (m, 3H).
^{13}C NMR (126 MHz, $CDCl_3$) δ 192.24, 170.41, 134.28, 133.88, 128.88, 127.77, 66.05, 20.53.

2C:
^{1}H NMR (400 MHz, $CDCl_3$) δ 7.89 (t, *J* = 7.2 Hz, 2H), 6.95 (t, *J* = 7.2 Hz, 2H), 5.29 (d, *J* = 5.8 Hz, 2H), 3.86 (s, 3H), 2.34 – 2.12 (m, 3H).
^{13}C NMR (101 MHz, $CDCl_3$) δ 190.73, 170.61, 164.16, 130.17, 127.33, 114.16, 65.87, 55.64, 20.72.

2D:
^{1}H NMR (400 MHz, $CDCl_3$) δ 7.90 (d, *J* = 9.0 Hz, 2H), 7.02 (d, *J* = 9.0 Hz, 2H), 5.28 (s, 2H), 4.75 (d, *J* = 2.4 Hz, 2H), 2.56 (d, *J* = 2.3 Hz, 1H), 2.21 (s, 3H).
^{13}C NMR (126 MHz, $CDCl_3$) δ 190.72, 170.46, 161.93, 130.05, 128.12, 115.05, 77.71, 76.42, 65.83, 56.00, 20.60.

2E:
^{1}H NMR (500 MHz, $CDCl_3$) δ 7.76 (d, *J* = 8.6 Hz, 2H), 7.61 (d, *J* = 8.6 Hz, 2H), 5.26 (s, 2H), 2.58 – 2.48 (m, 1H), 1.74 – 1.63 (m, 2H), 1.52 – 1.43 (m, 2H), 1.41 – 1.32 (m, 4H), 0.92 (t, *J* = 7.3 Hz, 6H).
^{13}C NMR (126 MHz, $CDCl_3$) δ 191.76, 176.02, 133.22, 132.28, 129.39, 129.08, 65.60, 45.11, 34.66, 34.48, 20.65, 14.11.

2F:
^{1}H NMR (500 MHz, $CDCl_3$) δ 7.87 (d, *J* = 8.8 Hz, 2H), 6.91 (d, *J* = 8.9 Hz, 2H), 5.26 (s, 2H), 3.83 (s, 3H), 2.59 – 2.45 (m, 1H), 1.77 – 1.61 (m, 2H), 1.62 – 1.14 (m, 6H), 0.89 (dt, *J* = 17.2, 6.6 Hz, 6H).
^{13}C NMR (126 MHz, $CDCl_3$) δ 190.49, 175.46, 163.71, 129.70, 128.11, 127.06, 113.73, 65.18, 55.05, 44.78, 34.37, 20.30, 13.79.

2G:

^{1}H NMR (500 MHz, $CDCl_3$) δ 8.56 (s, 1H), 8.05 (d, *J* = 13.1 Hz, 1H), 7.94 (d, *J* = 8.7 Hz, 2H), 6.95 (d, *J* = 8.7 Hz, 2H), 6.76 (d, *J* = 6.7 Hz, 1H), 5.52 (s, 2H), 4.23 (d, *J* = 7.1 Hz, 2H), 3.87 (s, 3H), 3.70 – 3.58 (m, 4H), 3.28 – 3.15 (m, 4H), 1.53 (t, *J* = 7.1 Hz, 3H), 1.49 (s, 8H).
^{13}C NMR (126 MHz, $CDCl_3$) δ 191.55, 191.15, 172.89, 164.98, 164.20, 154.76, 154.47, 148.69, 144.91, 136.16, 130.33, 127.54, 114.23, 113.97, 113.78, 109.61, 104.44, 80.35, 66.22, 55.66, 50.16, 49.39, 28.57, 14.54.

3. 3. General Synthesis of Compound III:

1 equivalent of compound 3 was taken in a 10 ml test tube with a stirrer bar. 1 equivalent of fused NaOAc, 1 equivalent of hippuric acid, and 3 equivalents of acetic anhydride was added to the above compound. The reaction was heated to 60 °C-80 °C for 6-8 h. The progress of the reaction was checked by TLC. The reaction was quenched by saturated bicarbonate solution, extracted with EtOAc, dried over Na_2SO_4, and purified by column chromatography.

3A: ($C_{19}H_{15}N_2O_6^{+}$) Calculated Mass: 367.0925 Observed Mass: 367.0938

^{1}H NMR (400 MHz, $CDCl_3$) δ 8.30 (d, *J* = 8.8 Hz, 2H), 8.22 (d, *J* = 8.8 Hz, 1H), 8.13 (d, *J* = 8.0 Hz, 2H), 7.75 – 7.51 (m, 5H), 5.55 (s, 2H), 1.96 (d, *J* = 13.0 Hz, 3H).
^{13}C NMR (101 MHz, $CDCl_3$) δ 134.11, 130.10, 129.23, 128.71, 124.83, 124.27, 123.64, 63.27, 20.64

3B: ($C_{19}H_{16}NO_4^{+}$) Exact Mass: 322.1074 Observed Mass: 322.1072

^{1}H NMR (400 MHz, $CDCl_3$) δ 8.16 – 8.01 (m, 3H), 7.86 – 7.75 (m, 2H), 7.59 (dt, *J* = 7.2, 6.6 Hz, 2H), 7.54 – 7.36 (m, 9H), 5.73 (s, 2H), 5.54 (s, 1H), 1.97 (s, 3H), 1.93 (s, 2H).
^{13}C NMR (101 MHz, $CDCl_3$) δ 170.67, 165.77, 146.56, 144.22, 135.25, 133.79, 133.50, 130.53, 130.32, 130.06, 129.04, 128.64, 128.26, 125.51, 63.65, 60.22, 20.77.

3C: ($C_{20}H_{18}NO_5^{+}$) Exact Mass: 352.1179 Observed Mass: 352.1188

^{1}H NMR (400 MHz, $CDCl_3$) δ 8.09 (dd, *J* = 5.7, 4.1 Hz, 12H), 7.92 (dd, *J* = 7.0, 1.9 Hz, 7H), 7.68 – 7.38 (m, 23H), 7.09 – 6.86 (m, 12H), 5.74 (s, 8H), 5.57 (s, 4H), 3.89 (s, 11H), 3.88 (d, *J* = 5.8 Hz, 6H), 2.01 (s, 11H), 1.96 (s, 6H).
^{13}C NMR (101 MHz, $CDCl_3$) δ 170.75, 161.50, 143.50, 133.82, 133.27, 132.80, 132.45, 131.21, 130.32, 129.04, 128.63, 128.30, 127.65, 125.66, 113.89, 63.39, 59.85, 55.52, 29.85, 20.88.

3D: ($C_{22}H_{18}NO_5^{+}$) Exact Mass: 376.1180 Observed Mass: 376.1201

^{1}H NMR (400 MHz, $CDCl_3$) δ 8.09 (d, *J* = 7.5 Hz, 2H), 7.94 (t, *J* = 8.7 Hz, 2H), 7.59 (t, *J* = 7.3 Hz, 1H), 7.49 (dd, *J* = 11.4, 4.1 Hz, 2H), 7.08 (d, *J* = 8.4 Hz, 2H), 5.64 (d, *J* = 70.2 Hz, 2H), 4.76 (dd, *J* = 10.7, 1.6 Hz, 2H), 2.58 (dd, *J* = 2.3, 1.5 Hz, 1H), 2.01 (d, *J* = 0.6 Hz, 3H).
^{13}C NMR (126 MHz, $CDCl_3$) δ 170.68, 165.88, 162.60, 159.43, 143.24, 133.34, 132.70, 131.09, 130.28, 129.03, 128.57, 128.33, 125.63, 114.83, 78.22, 76.09, 59.92, 56.03, 20.78.

3E: ($C_{25}H_{27}BrNO_4^{+}$) Exact Mass: 484.1118 Observed Mass: 484.1122

^{1}H NMR (400 MHz, $CDCl_3$) δ 8.11 (d, *J* = 7.3 Hz, 2H), 7.60 (d, *J* = 7.4 Hz, 1H), 7.58 – 7.45 (m, 4H), 7.33 (d, *J* = 8.3 Hz, 2H), 5.57 (s, 2H), 2.27 – 2.15 (m, 1H), 1.41 – 1.31 (m, 2H), 1.24 (d, *J* = 6.7 Hz, 2H), 1.05 (dt, *J* = 14.0, 7.1 Hz, 4H), 0.77 (t, *J* = 7.2 Hz, 6H).

^{13}C NMR (126 MHz, CDCl$_3$) δ 175.94, 163.93, 162.67, 145.86, 133.59, 133.41, 132.19, 131.64, 130.90, 129.11, 128.50, 125.52, 124.59, 62.68, 45.32, 34.58, 20.59, 14.07.

3F: ($C_{26}H_{30}NO_5^+$) Exact Mass: 436.2118 Observed Mass: 436.2113

^{1}H NMR (500 MHz, CDCl$_3$) δ 8.10 (t, *J* = 8.0 Hz, 4H), 7.88 (d, *J* = 8.9 Hz, 2H), 7.58 (dd, *J* = 7.9, 5.7 Hz, 2H), 7.52 – 7.47 (m, 6H), 6.96 (dd, *J* = 21.7, 8.8 Hz, 4H), 5.78 (s, 2H), 5.60 (s, 2H), 3.88 (s, 3H), 3.85 (s, 3H), 2.30 – 2.22 (m, 2H), 1.09 (ddd, *J* = 31.0, 15.2, 7.6 Hz, 9H), 0.91 – 0.82 (m, 7H), 0.77 (dt, *J* = 14.8, 7.3 Hz, 13H).
^{13}C NMR (126 MHz, CDCl$_3$) δ 176.01, 164.43, 161.52, 147.63, 133.14, 132.86, 132.28, 131.38, 131.11, 129.96, 129.23, 129.08, 129.02, 128.34, 128.26, 127.33, 127.03, 125.85, 124.92, 113.89, 113.77, 62.76, 55.45, 45.35, 34.63, 29.84, 20.57, 14.08.

3. 4. Synthetic procedure of photocage 4:

In a 5 ml round-bottomed flask, photocage **3F** was dissolved in 0.4 ml ethanol. In a separate glass vial, 13 mg of GABA was dissolved in 16 µL of distilled water, and 16 µL of trimethylamine was added to it and stirred for 5 min. The resulting solution was added to the first solution. The reaction was further stirred for 2 h, and the progress of the reaction was checked by TLC. The reaction was quenched with excess water and extracted with ethyl acetate. The ethyl acetate was dried over Na_2SO_4 and purified by column chromatography with a 50 % ethyl acetate: hexane mixture.
Yield: 80%

3. 5. Synthesis of SNAP-Tag and Biotin-Tag GFP-Photocages:

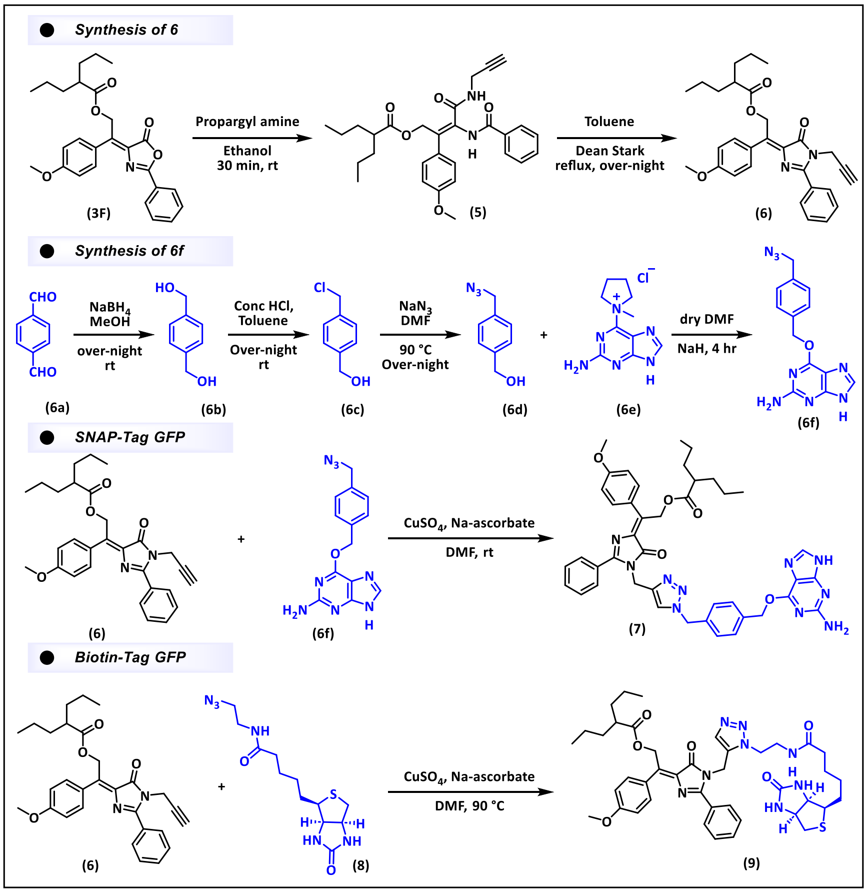

Scheme S2: Synthetic procedure of SNAP-Tag and Biotin-Tag GFP-photocages.

Synthetic procedure for SNAP-Tag GFP:

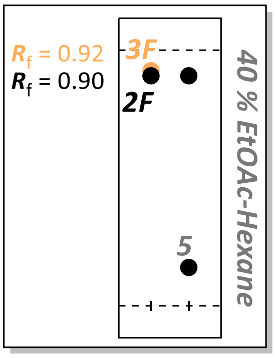

Compound 5: To synthesize photocage **5**, we separate photocage **2F** and photocage **3F** as a mixture (we did not isolate them separately, as the polarity of these two compounds is very close). The photocage **3F** (+ compound **2F**) was dissolved in 30 ml pure EtOH, then 1 ml of propargylamine was added to the crude mixture (for 5 gm) and stirred for 30 min. The solvent was evaporated under reduced pressure and purified by column chromatography to extract photocage **5**, and photocage **2F** as well. (20 %- 40 % Ethyl acetate: hexane)

Yield: 10 % (after two steps)

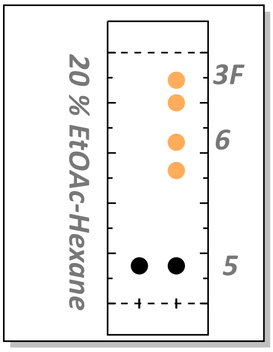

Photocage 6: 2 gm of photocage **5a** was dissolved in 10 ml dry toluene and refluxed overnight with a dean-stark apparatus. The solvent was evaporated and purified by column chromatography to afford 200 mg of photocage **6**. (10 %- 20 % Ethyl acetate: hexane)

Yield: 20 %

^{1}H NMR (400 MHz, $CDCl_3$) δ 7.92 (t, *J* = 8.8 Hz, 2H), 7.83 (d, *J* = 8.9 Hz, 1H), 7.59 – 7.48 (m, 4H), 6.94 (t, *J* = 8.7 Hz, 2H), 5.80 (d, *J* = 121.3 Hz, 2H), 4.47 (d, *J* = 2.1 Hz, 2H), 4.38 (d, *J* = 2.2 Hz, 2H), 3.84 (d, *J* = 6.3 Hz, 3H), 2.34 (s, 1H), 2.30 – 2.20 (m, 1H), 1.09 (dd, *J* = 15.1, 7.5 Hz, 2H), 0.96 – 0.82 (m, 6H), 0.75 (dd, *J* = 13.4, 6.2 Hz, 6H).

^{13}C NMR (101 MHz, $CDCl_3$) δ 176.21, 169.30, 161.02, 143.63, 136.55, 132.92, 132.43, 131.66, 131.50, 128.94, 128.65, 127.79, 113.69, 99.93, 78.19, 72.83, 59.40, 55.46, 45.39, 41.09, 34.65, 31.69, 29.84, 20.56, 14.13.

Photocage 6f:

The synthesis of photocage **6f** started with a bulk amount of terepthalaldehyde, and yields are calculated consecutively.

Photocage 6b:

12 gm of terepthalaldehyde was dissolved in 120 ml dry MeOH and chilled to 0 °C. 4 eq of $NaBH_4$ was added to the above solution in portions over 1 h. The reaction mixture turned turbid after the completion of the addition. The reaction was stirred overnight at room temperature. The solvent was evaporated under reduced pressure and quenched with 6 N HCl. The organic photocage was extracted with diethyl ether (yield was less) or ethyl acetate. The organic solvent was dried over Na_2SO_4 and concentrated under a rotary evaporator to afford a pure white solid of photocage **6b** (11.7 gm).

^{1}H NMR (400 MHz, DMSO-d_6) δ 7.25 (d, *J* = 10.9 Hz, 1H), 4.46 (d, *J* = 10.8 Hz, 1H).

Photocage 6c:

3 gm of photocage **6b** was suspended in 70 ml of toluene and 5.6 ml of Conc. HCl was slowly added to the mixture. The reaction was stirred overnight at room temperature. The reaction was quenched with saturated $NaHCO_3$, and the organic compound was extracted with diethyl ether. The organic part was dried over Na_2SO_4 and concentrated under reduced pressure to afford the crude compound **6c**. The crude mixture was washed with hexane (30 ml) to remove the minor dichloro compound.

Yield: 2.2 gm (off-white solid)

^{1}H NMR (400 MHz, $CDCl_3$) δ 7.37 (q, *J* = 8.1 Hz, 4H), 4.69 (s, 2H), 4.59 (s, 2H).
^{13}C NMR (126 MHz, $CDCl_3$) δ 141.31, 137.02, 128.95, 127.40, 65.02, 46.10.

Compound 6d:
3 gm of compound **6c** was dissolved in DMF, and 1.5 eq of NaN_3 was added to it. The reaction mixture was stirred for 24 h at 90 °C. The reaction was quenched with water and extracted with ethyl acetate to afford the pure compound **6d** (pale yellow liquid).

^{1}H NMR (400 MHz, $CDCl_3$) δ 7.37 (d, *J* = 8.0 Hz, 2H), 7.29 (d, *J* = 8.0 Hz, 2H), 4.68 (s, 2H), 4.31 (s, 2H).
^{13}C NMR (126 MHz, $CDCl_3$) δ 141.39, 134.60, 128.71, 128.48, 127.42, 64.77, 54.61.

Compound 6f:
2 gm of 6-chloropurin was dissolved in dry DMF (30 ml, 60 °C, once dissolved the reaction was allowed to stir at room temperature), then 1.5 eq of 1-methyl pyrrolidine was added to the reaction mixture. The reaction mixture was stirred for 24 h. 8 ml of acetone was added to the reaction mixture to complete the precipitation. The precipitate was filtered off and washed with diethyl ether to afford the pyrolidium salt of purine **6e**.
Compound **6d** was dissolved in dry DMF and cooled to 0 °C, then 1.2 eq of NaH (60 % in mineral oil) was added to the reaction mixture in portions. The reaction mixture was stirred for 30 min at 0 °C, and 1.2 eq of compound **6e** was added to the reaction. The reaction was allowed to room temperature and stirred for 4- 5 h. The reaction mixture was quenched with water and extracted with ethyl acetate. The product was purified by column chromatography (DCM: MeOH= 5 %). The yield was pretty much less than its reported value.

^{1}H NMR (500 MHz, DMSO) δ 12.42 (s, 1H), 7.81 (s, 1H), 7.53 (d, *J* = 7.8 Hz, 2H), 7.40 (d, *J* = 7.8 Hz, 2H), 6.28 (s, 2H), 5.50 (s, 2H), 4.46 (s, 2H).
^{13}C NMR (126 MHz, DMSO) δ 159.81, 159.61, 155.21, 137.79, 136.77, 135.31, 128.65, 128.43, 113.52, 66.31, 53.33.

Compound 7:
Compound **6** (1 eq) and compound **6f** were dissolved in DMF and to the reaction mixture, 10 mol % of CuI was added. The reaction was stirred overnight at room temperature, and the yellow precipitate was filtered off to afford the pure compound with a yield of 67%. We have tried the reaction with $CuSO_4$ and Na-ascorbate in THF: H_2O; however, the yield was less.

Due to the insolubility of the product in DMSO-d_6 and MeOH-d_4, we did not get the NMR. The compound was characterized by HRMS (Figure S51, Calculated Mass **[$C_{42}H_{45}N_{10}O_5^+$ = 769.3569, found= 769.3562]**), and purity was checked by RP-HPLC.

Compound 9:
To synthesize compound **9**, first, we prepared biotin azide (**8**) after following our lab procedure and characterized it by HRMS **(Chemical Formula: $C_{12}H_{21}N_6O_2S^+$; Calculated Mass: 313.1441; Observed Mass: 313.1434)**.

To a THF (5.0 mL) solution of compound **6** (43 mg, 1 eq) were added sodium ascorbate (10 mol %) and biotin azide (25 mg, 0.9 eq). The reaction mixture was degassed for 15 min by purging argon gas. Then, 2.0 mg (0.003 mmol) of $CuSO_4$ in 0.5 mL water was added to the reaction mixture. The reaction was stirred at 40 °C for overnight. The completion of the reaction was monitored by TLC. The THF was removed, and the workup was done with ethyl acetate and brine solution. The final product was directly characterized by HRMS analysis.

3. 6. Synthesis of Phenalenone:

To synthesize the singlet oxygen generator, phenalenone, $AlCl_3$ (3.00 g), and naphthalene (1.00 g) were added to 20 mL CH_2Cl_2, and the mixture was cooled to 0 °C. Cinnamoyl chloride (1.4 g) was added in succession under 0 °C, and the mixture was taken to reflux for 3 h. Then, the reaction mixture was quenched with iced hydrochloric acid and filtered. The mixture was workup with 50 ml × 3 CH_2Cl_2 and washed with brine. All the organic extracts

were combined, dried over anhydrous Na_2SO_4, and concentrated in a rotary evaporator to give a yellow solid. The crude product was further purified by column chromatography (10-20 % ethyl acetate: hexane).

Yield: 42 %

^{1}H NMR (400 MHz, CDCl$_3$) δ 8.74 – 8.58 (m, 1H), 8.21 (d, J = 8.1 Hz, 1H), 8.03 (d, J = 8.2 Hz, 1H), 7.90 – 7.71 (m, 3H), 7.60 (dd, J = 8.1, 7.2 Hz, 1H), 6.74 (d, J = 9.8 Hz, 1H).

^{13}C NMR (126 MHz, CDCl$_3$) δ 185.78, 141.86, 135.00, 132.37, 132.04, 131.44, 130.51, 129.70, 129.45, 128.06, 127.77, 127.28, 126.77.

3. 7. Synthesis of Rhodamin-anthracene-based fluorescence reporter (SM-94):

Rhodamine B (2 mmol) was dissolved in 20 mL of anhydrous dichloromethane, and 4-dimethylamiopryidine (DMAP, 0.2 mmol) was added as the catalyst agent. Then, equimolar 9-anthracenemethanol (synthesized from anthracene-9-carbaldehyde by $NaBH_4$ reduction) and the dicyclohexyl carbodiimide (DCC, 2 mmol) were added. The mixture was stirred under an Argon atmosphere for 24 h. The reaction was directly worked with dichloromethane and water to dissolve the unreacted rhodamine B. All the organic extracts were dried over anhydrous Na_2SO_4 and concentrated under a rotary evaporator. The crude product was further reprecipitated with DCM: hexane (3 ml: 20 ml) several times to afford the compound **SM-94**. Finally, the product was characterized by HRMS analysis.

Chemical Formula: $C_{43}H_{42}N_2O_3^+$ Calculated Mass: 634.3190 Observed mass: 634.3176

3. 8. Synthetic procedure of thiophene derivative:

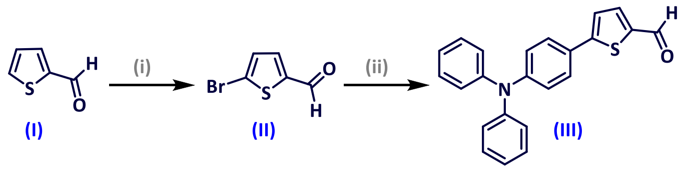

Reagent and Condition: (i) NBS, Chloroform, rt; (ii) 4-(Diphenylamino)phenylboronic acid, $Pd(PPh_3)_4$, Na_2CO_3, Dioxane-Water, 80 °C.

Scheme S3: Synthetic scheme of thiophene derivative.

In an oven-dried 250 ml round-bottomed flask, 5.0 g of 2-thiophene carboxaldehyde (I) was dissolved in 100 ml of dry $CHCl_3$ and stirred. In the reaction mixture, 8.7 g (49.04 mmol) N-bromosuccinamide was slowly added. The reaction was stirred for 14 h at room temperature and monitored by TLC. The organic compound was extracted with $CHCl_3$ and dried under Na_2SO_4. The organic layer was evaporated under reduced pressure to get crude compound II.

The crude compound II (502 mg, 2.6 mmol) was taken in an oven-dried round-bottomed flask. To that, 1 g (3.45 mmol) of 4-(Diphenylamino)phenylboronic acid and 40 mg (0.03 mmol) freshly prepared $Pd(PPh_3)_4$ were added and purged with argon. To the above mixture, 20 ml dioxane was added, and the mixture was bubbled with argon for 30 min. A water solution (10 ml) of Na_2CO_3 (1 g, 9.4 mmol) was added to the mixture and again bubbled for 15 min. The reaction was set to reflux at 80 °C for overnight, followed by a TLC check. The reaction was quenched with water, and the organic compound was extracted with ethyl acetate. The organic layer was dried over Na_2SO_4 and evaporated under reduced pressure. The crude compound was finally purified by column chromatography using 10% ethyl acetate: hexane as an eluent and characterized by NMR spectroscopy.

Bright yellow solid compound (Isolated Yield: 52%)

^{1}H NMR (400 MHz, CDCl$_3$) δ 9.85 (s, 1H), 7.71 (d, J = 3.9 Hz, 1H), 7.52 (d, J = 8.4 Hz, 2H), 7.30 (t, J = 7.7 Hz, 5H), 7.17 – 7.10 (m, 5H), 7.07 (t, J = 7.9 Hz, 3H).

^{13}C NMR (126 MHz, CDCl$_3$) δ 182.67, 154.72, 149.31, 147.13, 141.50, 137.78, 129.63, 127.39, 126.31, 125.33, 124.03, 123.00, 122.52.

2. 2. Synthetic procedure of hippuric acid derivatives:

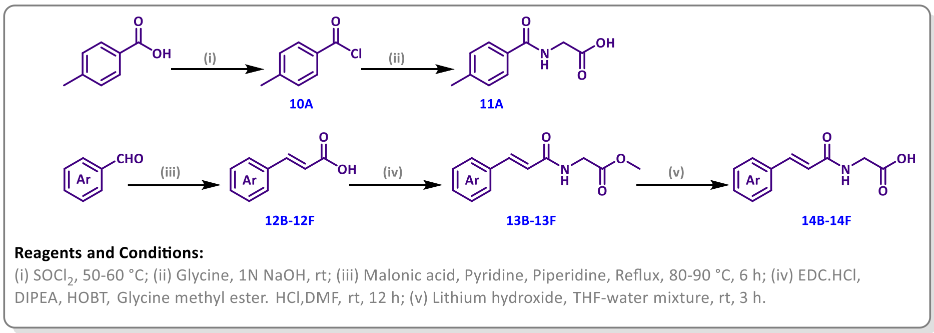

Scheme S4: General synthetic scheme of the corresponding hippuric acids (11A and 14B-14F).

Synthetic procedure of 11A:

In an oven-dried round-bottomed flask, 5 ml of thionyl chloride (69 mmol) was added to 4.5 gm of *p*-toluic acid (33 mmol). The mixture was heated to 50-60 °C for 4 h to get the acid chloride 10A. The excess thionyl chloride was removed under vacuum, and the crude acid chloride was directly used for the next step.

In a 250 ml round-bottomed flask, 1 gm of glycine was dissolved into 20 ml of 10 % NaOH at 0 °C. To the mixture, the crude acid chloride was added dropwise for 10 min. The mixture was left to stir for 2 h at room temperature. The reaction was finally quenched with 6N HCl. The formed precipitate was collected by vacuum filtration and recrystallized from hot water. Isolated Yield: 56%

Synthetic Procedure of 12B-12F:

To synthesize the cinnamic acid derivatives (12B-12F), we followed the classical Knoevenagel condensation reaction. In an oven-dried one-neck round-bottomed flask, the corresponding aldehyde (50 mmol), malonic acid (125 mmol), pyridine (50 ml), and piperidine (1 ml) were taken together. The reaction was heated at 80-90 °C for 6h. The mixture was directly poured into ice-cold water (100 ml) and quenched with 6N HCl, resulting in the formation of a precipitate of the corresponding cinnamic acid. The precipitate was collected by vacuum filtration, dried overnight under the oven, and was directly used for the next step.

12B: Yield: 59%

^{1}H NMR (400 MHz, DMSO-D6) δ 12.72 (brs, 1H), 8.43 – 8.12 (m, 2H), 8.12 – 7.86 (m, 2H), 7.68 (dd, J = 16.1, 3.9 Hz, 1H), 6.92 – 6.60 (m, 1H).

^{13}C NMR (126 MHz, DMSO) δ 166.99, 147.93, 141.28, 140.73, 129.26, 123.89, 123.60.

12C: Yield: 56%

^{1}H NMR (500 MHz, DMSO) δ 8.38 (d, J = 15.7 Hz, 1H), 8.18 (d, J = 8.3 Hz, 1H), 8.04 – 7.95 (m, 2H), 7.93 (d, J = 7.0 Hz, 1H), 7.66 – 7.51 (m, 3H), 6.60 (d, J = 15.7 Hz, 1H).

^{13}C NMR (126 MHz, DMSO) δ 167.48, 140.26, 133.35, 131.03, 130.80, 130.43, 128.78, 127.21, 126.36, 125.79, 125.28, 123.01, 121.95.

12D: Yield: 56%

^{1}H NMR (500 MHz, DMSO) δ 7.52 – 7.35 (m, 3H), 6.63 (d, J = 8.8 Hz, 2H), 6.14 (d, J = 15.8 Hz, 1H), 3.35 (dd, J = 13.9, 6.9 Hz, 4H), 1.07 (t, J = 6.9 Hz, 6H).

^{13}C NMR (126 MHz, DMSO) δ 168.65, 149.31, 145.09, 130.34, 120.81, 112.27, 111.39, 44.00, 12.63.

12E: Yield: 62%

^{1}H NMR (500 MHz, DMSO) δ 7.52 (dd, J = 17.0, 12.4 Hz, 3H), 7.34 (t, J = 7.5 Hz, 4H), 7.12 (t, J = 7.0 Hz, 2H), 7.07 (d, J = 7.6 Hz, 4H), 6.87 (d, J = 8.5 Hz, 2H), 6.34 (d, J = 15.9 Hz, 1H).

^{13}C NMR (126 MHz, DMSO) δ 167.78, 149.12, 146.38, 143.49, 129.72, 129.51, 127.28, 125.08, 124.13, 121.01, 116.45.

Synthetic Procedure of 13B-13F:

To synthesize the amide derivatives 13B-13F, we followed the Steglich esterification, using EDC as a coupling agent. The corresponding cinnamic acid derivatives (12B-12F) were dissolved in dry DMF, followed by the addition of HOBT and diisopropylethylamine (DIPEA). The mixture was stirred for 20 min at room temperature. To the reaction mixture, EDC.HCl was added in one portion and stirred for 30 min at room temperature to form the EDC-complex. Finally, Glycine methyl ester hydrochloride was added to the reaction mixture and stirred overnight. The progress of the reaction was monitored by TLC. The reaction was quenched using a brine solution, and the compounds were extracted from ethyl acetate in three fractions. The organic layer was dried and evaporated under reduced pressure. The crude amide was directly used for the next step.

Isolated Yield: 78%

^{1}H NMR (500 MHz, $CDCl_3$) δ 7.56 (d, J = 15.5 Hz, 1H), 7.37 (d, J = 8.8 Hz, 2H), 6.61 (d, J = 8.7 Hz, 2H), 6.23 (d, J = 15.5 Hz, 1H), 6.14 (s, 1H), 4.17 (d, J = 5.2 Hz, 2H), 3.77 (s, 3H), 3.37 (q, J = 7.1 Hz, 4H), 1.17 (t, J = 7.1 Hz, 6H).

^{13}C NMR (126 MHz, $CDCl_3$) δ 170.93, 167.11, 149.14, 142.37, 129.87, 121.71, 113.92, 111.37, 52.44, 44.52, 41.58, 12.70.

Isolated Yield: 62%

^{1}H NMR (500 MHz, $CDCl_3$) δ 7.55 (d, J = 15.6 Hz, 1H), 7.31 (d, J = 8.7 Hz, 2H), 7.24 (d, J = 7.1 Hz, 4H), 7.08 (d, J = 7.5 Hz, 4H), 7.04 (dd, J = 10.5, 4.2 Hz, 2H), 6.96 (d, J = 8.7 Hz, 2H), 6.29 (d, J = 15.6 Hz, 1H), 6.26 – 6.09 (t, 1H), 4.15 (d, J = 5.1 Hz, 2H), 3.75 (s, 3H).

^{13}C NMR (126 MHz, $CDCl_3$) δ 170.78, 166.44, 149.61, 147.19, 141.57, 129.57, 129.11, 127.96, 125.35, 123.93, 122.15, 117.30, 52.53, 41.60.

Isolated Yield: 64%

^{1}H NMR (500 MHz, $CDCl_3$) δ 7.69 (d, J = 15.2 Hz, 1H), 7.41 (d, J = 8.7 Hz, 2H), 7.26 – 7.21 (m, 4H), 7.13 (d, J = 3.8 Hz, 1H), 7.10 (dd, J = 6.5, 5.7 Hz, 5H), 7.03 (t, J = 7.8 Hz, 4H), 6.19 (d, J = 15.2 Hz, 1H), 6.14 – 6.04 (t, 1H), 4.15 (d, J = 5.1 Hz, 2H), 3.76 (s, 3H).

^{13}C NMR (126 MHz, $CDCl_3$) δ 170.62, 166.07, 148.22, 147.42, 146.89, 138.07, 135.03, 132.41, 129.54, 127.48, 126.88, 124.97, 123.60, 123.23, 123.04, 117.65, 52.61, 41.67.

Synthetic Procedure of 14B-14F:

The π-extended hippuric acids were synthesized from the corresponding 13B-13F. In a 100 ml round-bottomed flask, compound 13B-13F was dissolved in 20 ml THF. To the solution, a water solution of lithium hydroxide was added in one portion. The mixture was stirred for 2 h at room temperature, and the progress of the reaction was monitored by TLC. THF was removed under reduced pressure, and the reaction was quenched using 3N HCl. The compound was extracted by ethyl acetate and dried using Na_2SO_4. The crude hippuric acid derivatives were directly used for the next step.

Yield: White Crystalline (72%)

^{1}H NMR (500 MHz, DMSO) δ 8.75 (s, 1H), 7.77 (d, J = 6.9 Hz, 2H), 7.26 (d, J = 7.8 Hz, 2H), 3.93 (d, J = 5.6 Hz, 4H), 2.32 (s, 3H).

^{13}C NMR (126 MHz, DMSO) δ 171.71, 167.00, 141.77, 131.24, 129.62, 129.20, 127.52, 41.49, 21.19.

Yield: White Crystalline (72%)

^{1}H NMR (400 MHz, DMSO) δ 8.53 (s, 1H), 8.25 (d, J = 8.5 Hz, 2H), 7.85 (d, J = 7.0 Hz, 2H), 7.55 (d, J = 15.9 Hz, 1H), 6.94 (d, J = 15.9 Hz, 1H), 3.90 (d, J = 5.1 Hz, 2H).

^{13}C NMR (126 MHz, DMSO) δ 171.37, 165.10, 147.84, 141.54, 137.36, 128.96, 125.97, 124.32, 41.20.

2. 3. Synthetic Procedure of α-ketoesters:

The α-ketoester derivatives were synthesized from the corresponding phenacyl bromide derivatives. In a 100 ml round-bottomed flask, the corresponding phenacyl bromide (10 mmol) was dissolved in 10 ml dry DMF and stirred for 10 min. 15 mmol of Sodium 2-Propylvalerate (Na-Valproate) was added to the above reaction mixture in three portions. The resulting mixture was stirred for 4 h at room temperature, and the progress of the reaction was monitored by TLC. The reaction was quenched using a brine solution. The organic compound was extracted by ethyl acetate and washed vigorously with brine. The organic layer was dried over Na_2SO_4 and evaporated under reduced pressure. The compound was purified by column chromatography using 10% ethyl acetate: hexane.

Compound 2F: Yield 72%

^{1}H NMR (500 MHz, $CDCl_3$) δ 7.87 (d, J = 8.8 Hz, 2H), 6.91 (d, J = 8.9 Hz, 2H), 5.26 (s, 2H), 3.83 (s, 3H), 2.59 – 2.45 (m, 1H), 1.77 – 1.61 (m, 2H), 1.62 – 1.14 (m, 6H), 0.89 (dt, J = 17.2, 6.6 Hz, 6H).

^{13}C NMR (126 MHz, $CDCl_3$) δ 190.49, 175.46, 163.71, 129.70, 128.11, 127.06, 113.73, 65.18, 55.05, 44.78, 34.37, 20.30, 13.79.

Compound 2H: Yield 75%

^{1}H NMR (500 MHz, $CDCl_3$) δ 8.33 (d, J = 8.9 Hz, 2H), 8.07 (d, J = 8.8 Hz, 2H), 5.31 (s, 2H), 2.59 – 2.50 (m, 1H), 1.73 – 1.63 (m, 2H), 1.54 – 1.44 (m, 2H), 1.36 (dtd, J = 13.8, 7.0, 4.6 Hz, 4H), 0.92 (t, J = 7.3 Hz, 6H).

^{13}C NMR (126 MHz, $CDCl_3$) δ 191.57, 176.00, 150.83, 139.04, 129.08, 124.19, 65.87, 45.08, 34.66, 20.66, 14.13.

Synthetic Procedure of 15A-15F:

To synthesize the final oxazolone core, we followed a classical Erlynmayer condensation reaction between α-ketoesters and the derivatives of hippuric acids. 10 mmol of α-ketoesters was taken in a 10 ml test tube with a stirrer bar. 10 mmol of fused NaOAc, 10 mmol of hippuric acid, and 30 mmol of acetic anhydride were added to the α-ketoesters. The reaction was heated to 60 °C-80 °C for 3 h. The progress of the reaction was checked by TLC. The reaction was quenched by saturated bicarbonate solution, extracted with EtOAc, dried over Na2SO4, and purified by column chromatography (5%-10% ethyl acetate: hexane).

15A: Isolated Yield: 8%

^{1}H NMR (500 MHz, $CDCl_3$) δ 7.97 (d, J = 8.2 Hz, 2H), 7.88 (d, J = 8.9 Hz, 2H), 7.29 (d, J = 8.1 Hz, 2H), 6.98 (d, J = 8.9 Hz, 2H), 5.77 (s, 2H), 3.87 (s, 3H), 2.43 (s, 3H), 2.32 – 2.23 (m, 1H), 1.51 – 1.41 (m, 2H), 1.32 – 1.24 (m, 2H), 1.18 – 1.07 (m, 4H), 0.78 (t, J = 7.3 Hz, 6H).

^{13}C NMR (126 MHz, $CDCl_3$) δ 176.07, 166.12, 162.39, 161.44, 144.17, 143.68, 132.76, 132.26, 129.77, 128.34, 127.67, 122.92, 113.85, 59.28, 55.51, 45.36, 34.58, 21.95, 20.58, 14.07.

15B: Isolated Yield: 8%

^{1}H NMR (400 MHz, $CDCl_3$) δ 7.77 (d, J = 8.8 Hz, 2H), 7.63 (d, J = 16.5 Hz, 4H), 7.56 (d, J = 3.5 Hz, 5H), 7.47 (d, J = 8.8 Hz, 1H), 7.41 (dd, J = 7.0, 4.2 Hz, H), 6.95 (dd, J = 13.4, 8.8 Hz, 2H), 6.76 (d, J = 16.2 Hz, 1H), 5.75 (s, 1.3H), 5.53 (s, 0.6H), 3.85 (d, J = 5.4 Hz, 3H), 1.48 – 1.38 (m, 2H), 1.32 – 1.20 (m, 2.5H), 1.17 – 0.97 (m, 4H), 0.76 (dt, J = 11.1, 7.3 Hz, 6H).

^{13}C NMR (126 MHz, $CDCl_3$) δ 176.10, 165.62, 162.18, 161.49, 144.50, 143.36, 132.52, 131.45, 130.78, 129.24, 128.48, 128.23, 113.95, 113.72, 113.43, 62.59, 59.31, 55.54, 55.44, 45.36, 45.32, 34.64, 34.6, 20.58, 14.10.

15C: Isolated Yield: 10%

^{1}H NMR (400 MHz, $CDCl_3$) δ 8.27 (d, J = 5.7 Hz, 14H), 7.90 (d, J = 8.6 Hz, 3H), 7.77 (d, J = 8.7 Hz, 7H), 7.73 – 7.66 (m, 12H), 7.63 (d, J = 16.2 Hz, 7H), 7.49 (d, J = 8.8 Hz, 5H), 7.01 – 6.92 (m, 14H), 6.88 (d, J = 16.2 Hz, 6H), 5.76 (s, 7H), 5.54 (s, 4H), 5.28 (s, 3H), 3.86 (d, J = 5.9 Hz, 21H), 2.29 – 2.17 (m, 8H), 1.45 – 1.34 (m, 18H), 1.16 – 0.97 (m, 24H), 0.98 – 0.84 (m, 16H), 0.84 – 0.67 (m, 35H).

^{13}C NMR (126 MHz, $CDCl_3$) δ 191.09, 176.14, 176.02, 175.96, 165.11, 164.12, 163.65 161.92, 161.86, 161.20, 160.30, 149.32, 148.68, 146.71, 140.76, 139.87, 138.74, 132.77, 131.97, 131.62, 130.23, 128.65, 124.49, 117.71, 117.66, 114.16, 114.01, 113.80, 65.53, 62.52, 59.28, 55.65, 55.57, 55.47, 45.33, 45.28, 45.20, 34.72, 34.61, 34.57, 20.70, 20.56, 20.53, 14.18, 14.07.

15D: Isolated Yield: 12%

^{1}H NMR (400 MHz, $CDCl_3$) δ 8.27 (d, J = 8.8 Hz, 2H), 7.89 (d, J = 8.8 Hz, 2H), 7.62 (d, J = 15.8 Hz, 1H), 7.43 (d, J = 8.8 Hz, 2H), 6.65 (d, J = 8.8 Hz, 2H), 6.42 (d, J = 15.8 Hz, 1H), 5.75 (d, J = 9.2 Hz, 2H), 3.42 (dd, J = 13.9, 6.9 Hz, 4H), 2.26 (dt, J = 9.5, 4.8 Hz, 1H), 1.50 – 1.37 (m, 2H), 1.32 – 1.27 (m, 2H), 1.21 (t, J = 7.0 Hz, 6H), 1.17 – 1.02 (m, 4H), 0.75 (t, J = 7.2 Hz, 6H).

^{13}C NMR (126 MHz, $CDCl_3$) δ 175.97, 165.33, 165.12, 150.39, 147.75, 146.25, 142.32, 136.80, 136.31, 131.07, 123.31, 111.60, 105.45, 59.35, 45.28, 44.75, 34.58, 20.56, 13.99, 12.71.

15E: Isolated Yield: 16%

^{1}H NMR (400 MHz, $CDCl_3$) δ 8.31 (dd, J = 27.0, 8.8 Hz, 15H), 8.08 (d, J = 8.8 Hz, 5H), 7.87 (d, J = 8.9 Hz, 11H), 7.62 (d, J = 16.1 Hz, 6H), 7.39 (d, J = 8.8 Hz, 11H), 7.32 (t, J = 7.8 Hz, 24H), 7.23 – 7.09 (m, 35H), 6.99 (d, J = 8.7 Hz, 12H), 6.53 (d, J = 16.0 Hz, 5H), 5.75 (s, 10H), 5.32 (s, 4H), 2.39 (s, 3H), 2.30 – 2.19 (m, 6H), 1.56 – 1.17 (m, 64H), 1.06 (dd, J = 14.8, 7.4 Hz, 22H), 0.75 (t, J = 7.3 Hz, 34H).

^{13}C NMR (126 MHz, $CDCl_3$) δ 175.98, 165.03, 164.63, 151.03, 147.99, 146.57, 145.21, 141.88, 139.16, 135.82, 131.16. 129.95, 129.78, 129.09, 126.96, 126.00, 124.81, 124.21, 123.38, 121.01, 109.03, 65.88, 59.33, 45.28, 45.10, 44.87, 34.67, 34.58, 34.53, 29.84, 20.70, 20.68, 20.58, 14.16, 14.12, 14.01

15F: Isolated Yield: 12%

^{1}H NMR (500 MHz, $CDCl_3$) δ 8.28 (d, J = 8.8 Hz, 2H), 7.87 (d, J = 8.9 Hz, 2H), 7.75 (d, J = 15.6 Hz, 1H), 7.46 (d, J = 8.6 Hz, 2H), 7.29 (dt, J = 9.8, 4.9 Hz, 5H), 7.21 (s, 1H), 7.13 (d, J = 7.6 Hz, 4H), 7.08 (t, J = 7.3 Hz, 2H), 7.05 (d, J = 8.7 Hz, 2H), 6.42 (d, J = 15.6 Hz, 1H), 5.75 (s, 1.8H), 5.51 (s, 0.3H), 2.30 – 2.19 (m, 1H), 1.47 – 1.36 (m, 2H), 1.31 – 1.22 (m, 2H), 1.07 (dd, J = 15.1, 7.6 Hz, 4H), 0.76 (t, J = 7.4 Hz, 6H).

^{13}C NMR (126 MHz, $CDCl_3$) δ 175.93, 164.84, 164.02, 150.21, 148.86, 148.02, 147.18, 141.80, 139.53, 138.05, 137.75, 135.75, 134.27, 132.59, 131.20, 129.77, 129.61, 127.03,126.20, 126.18, 125.24, 123.94, 123.55, 123.34, 122.69, 109.84, 59.31, 45.26, 34.56, 20.57, 14.00.

3. 9. Characterization of GFP-photocages:

Figure S1: ^{1}H and ^{13}C NMR of compound 1A.

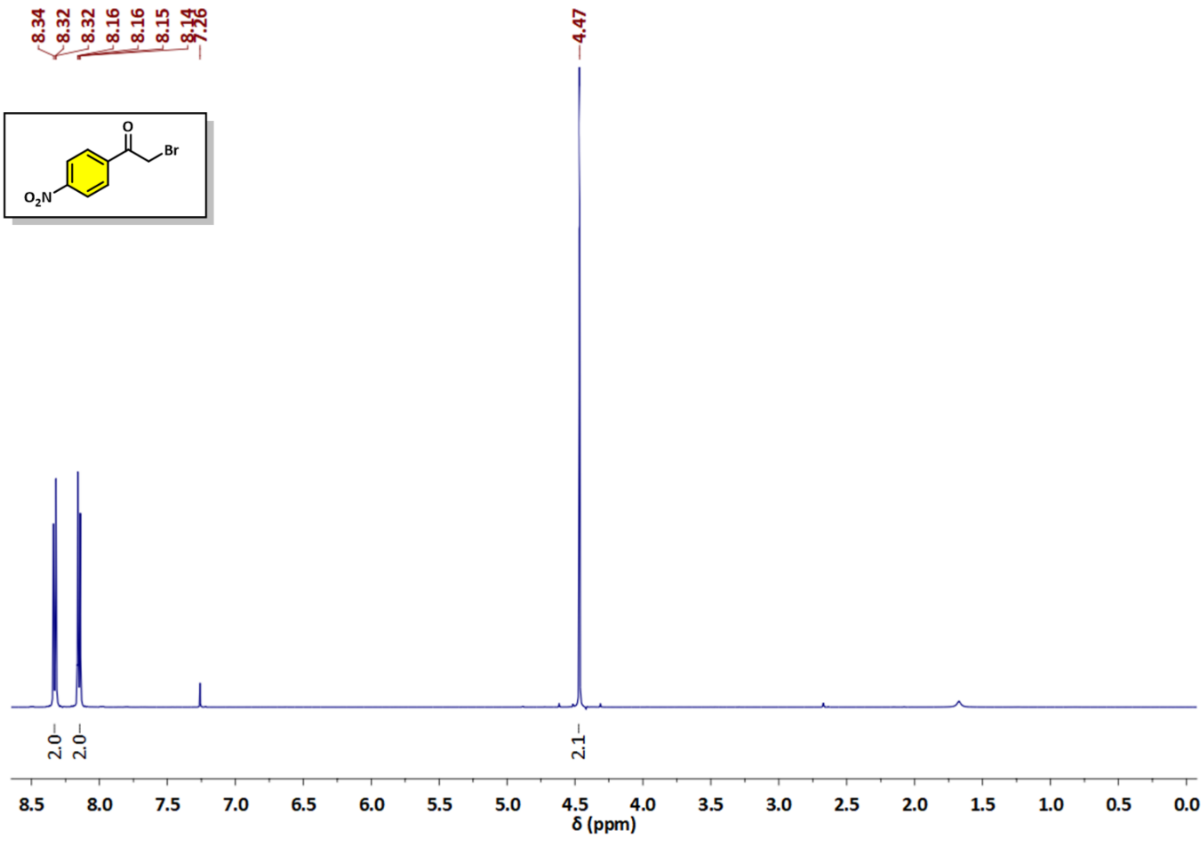

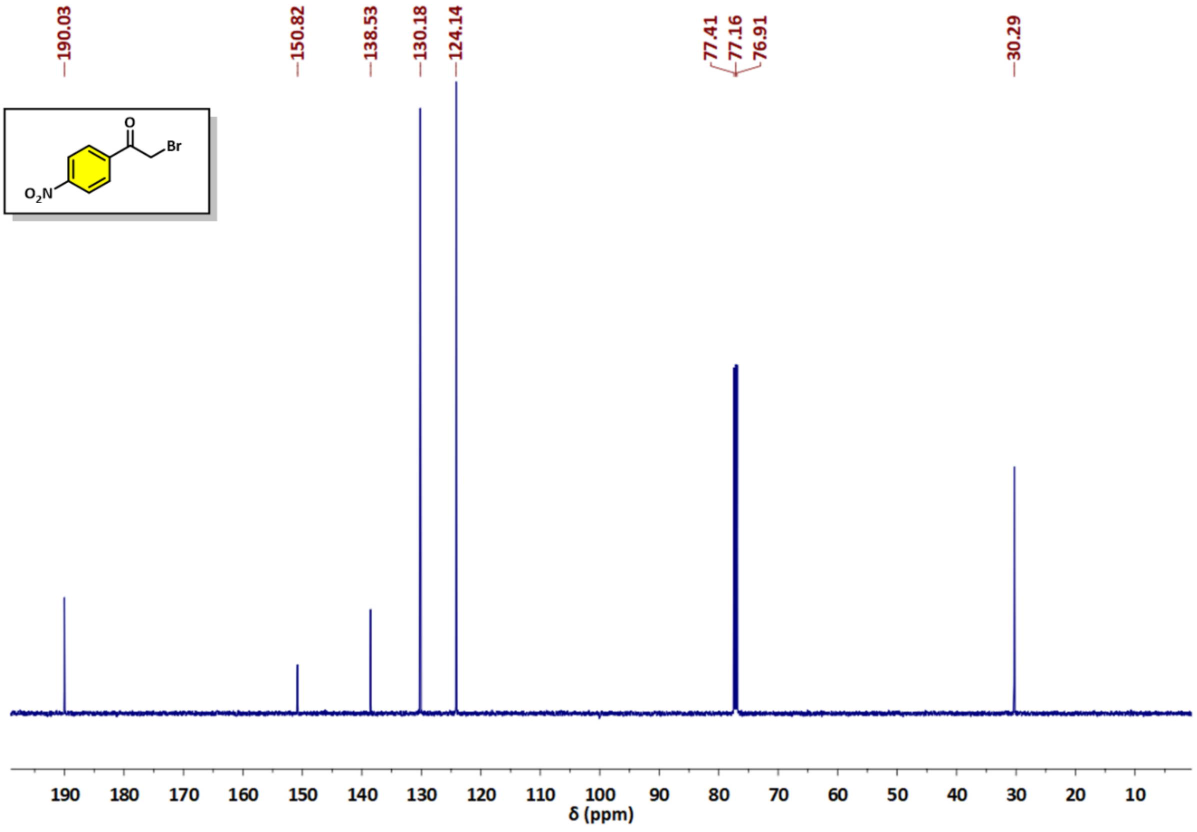

Figure S2: ^{1}H and ^{13}C NMR of compound 1C.

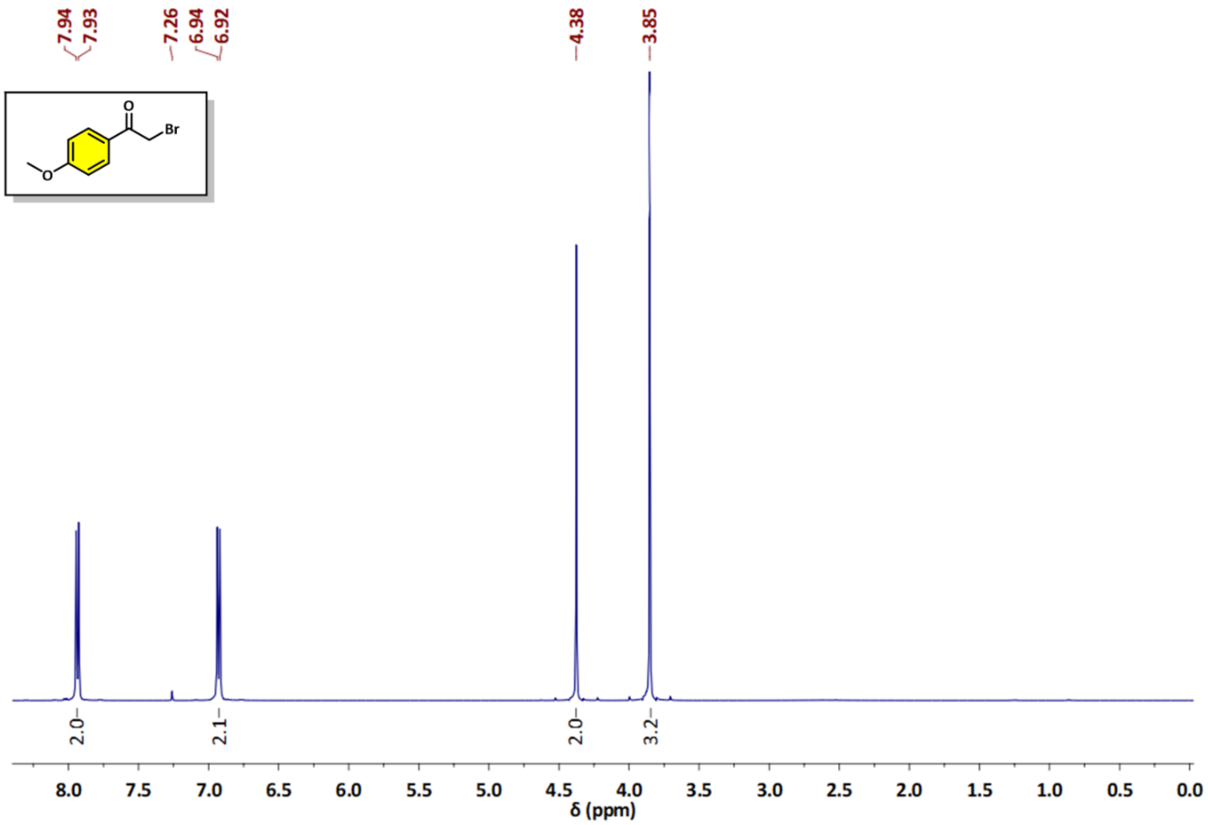

7.94
7.93
7.26
6.94
6.92
4.38
3.85
O
Br
O
2.0
2.1
2.0
3.2
8.0 7.5 7.0 6.5 6.0 5.5 5.0 4.5 4.0 3.5 3.0 2.5 2.0 1.5 1.0 0.5 0.0
δ (ppm)

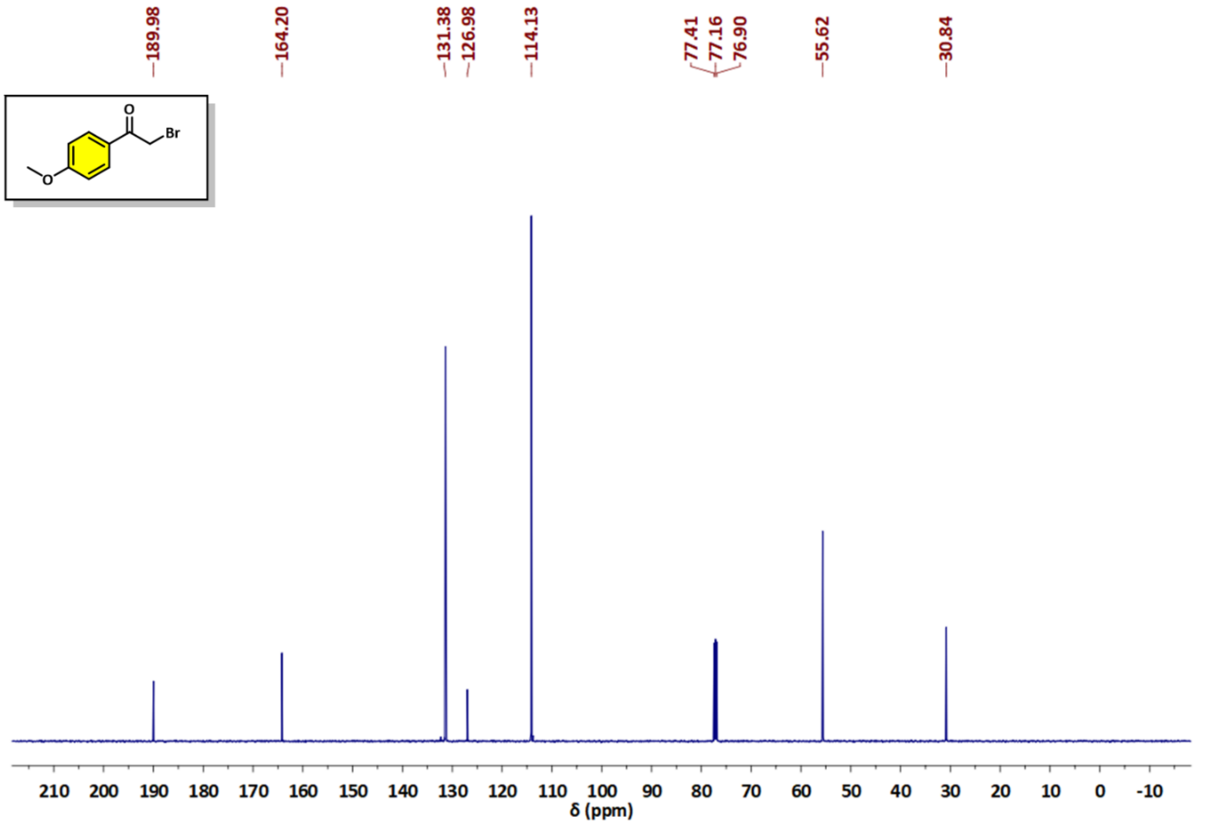

189.98
164.20
131.38
126.98
114.13
77.41
77.16
76.90
55.62
30.84
O
Br
O
210 200 190 180 170 160 150 140 130 120 110 100 90 80 70 60 50 40 30 20 10 0 -10
δ (ppm)

Figure S3: ^{1}H and ^{13}C NMR of compound 2A.

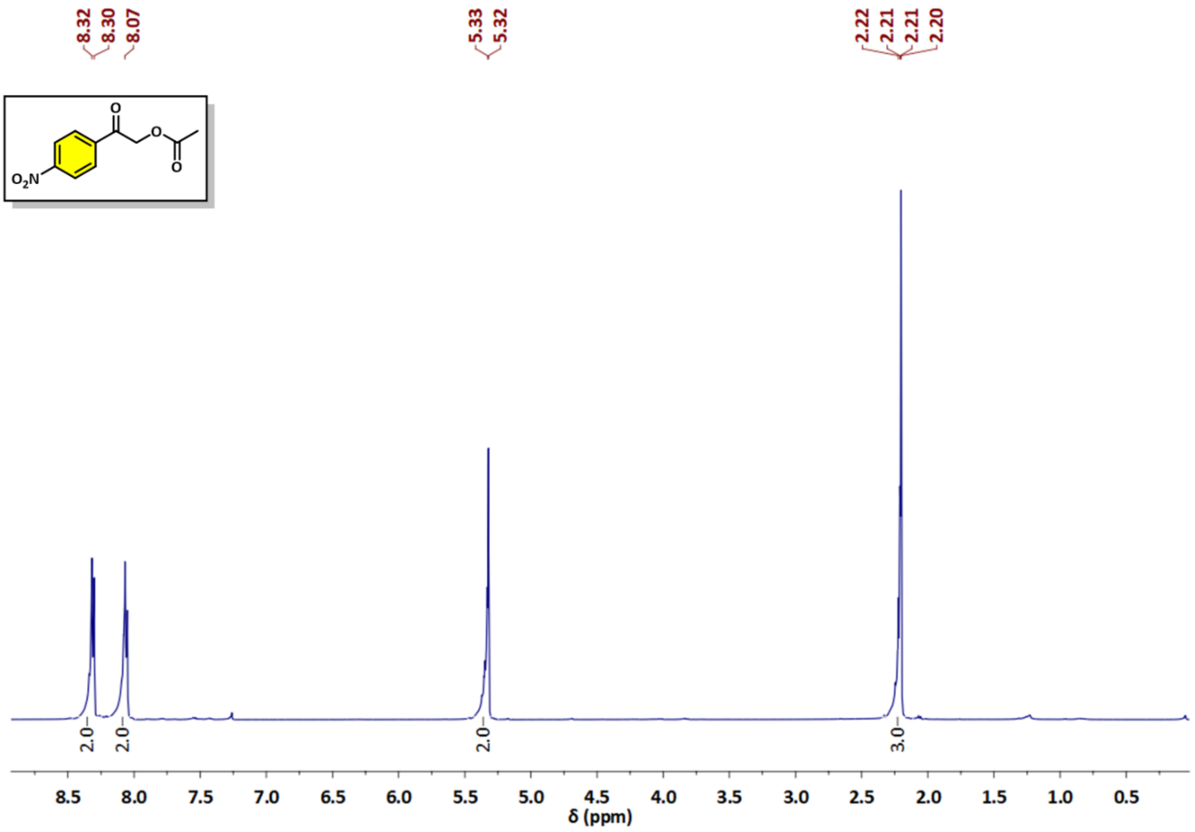

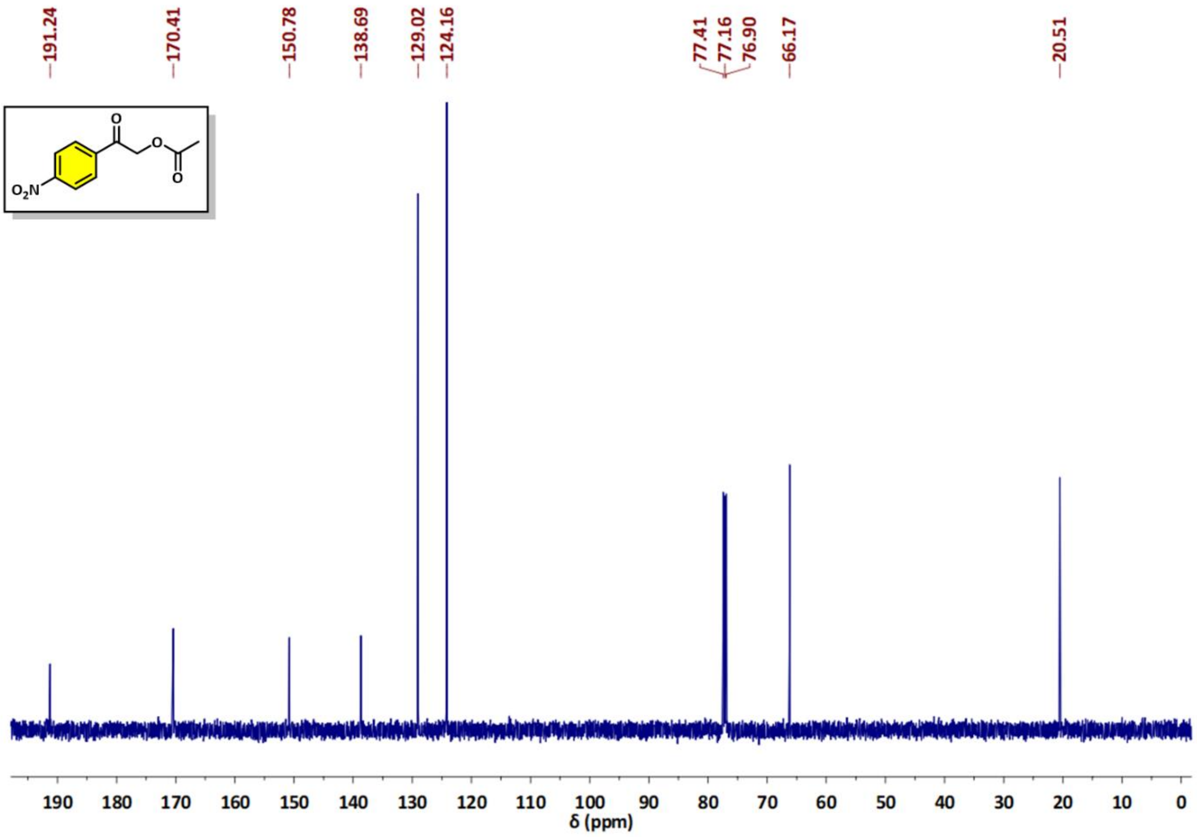

Figure S4: ^{1}H and ^{13}C NMR of compound 2B.

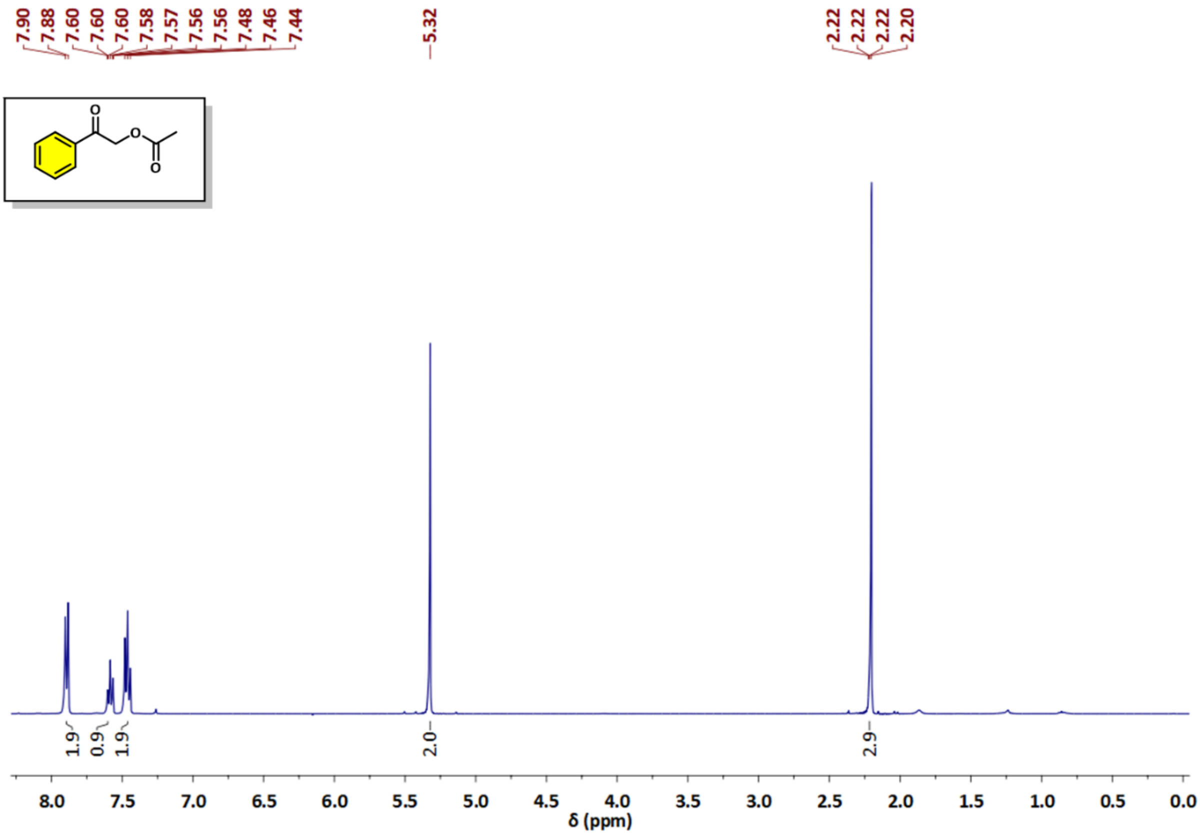

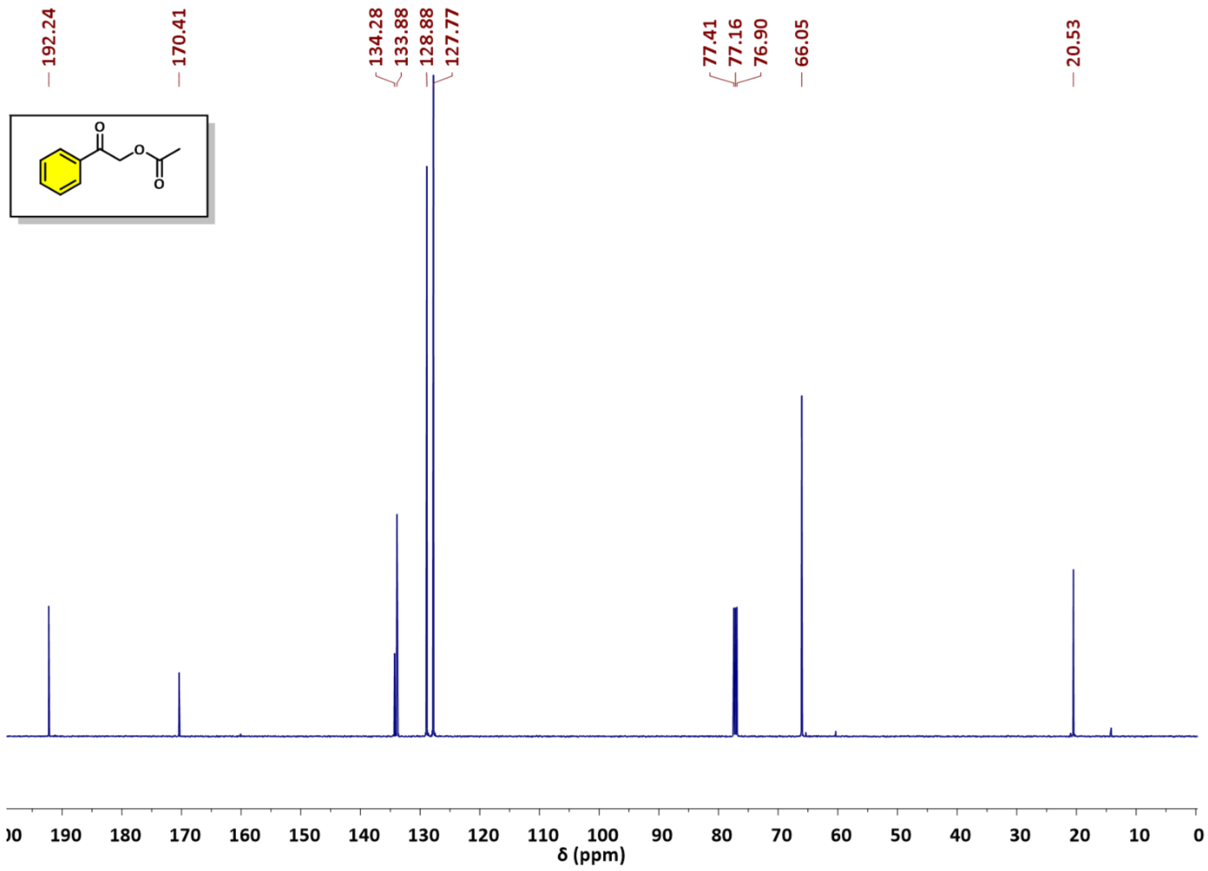

Figure S5: ^{1}H and ^{13}C NMR of compound 2C.

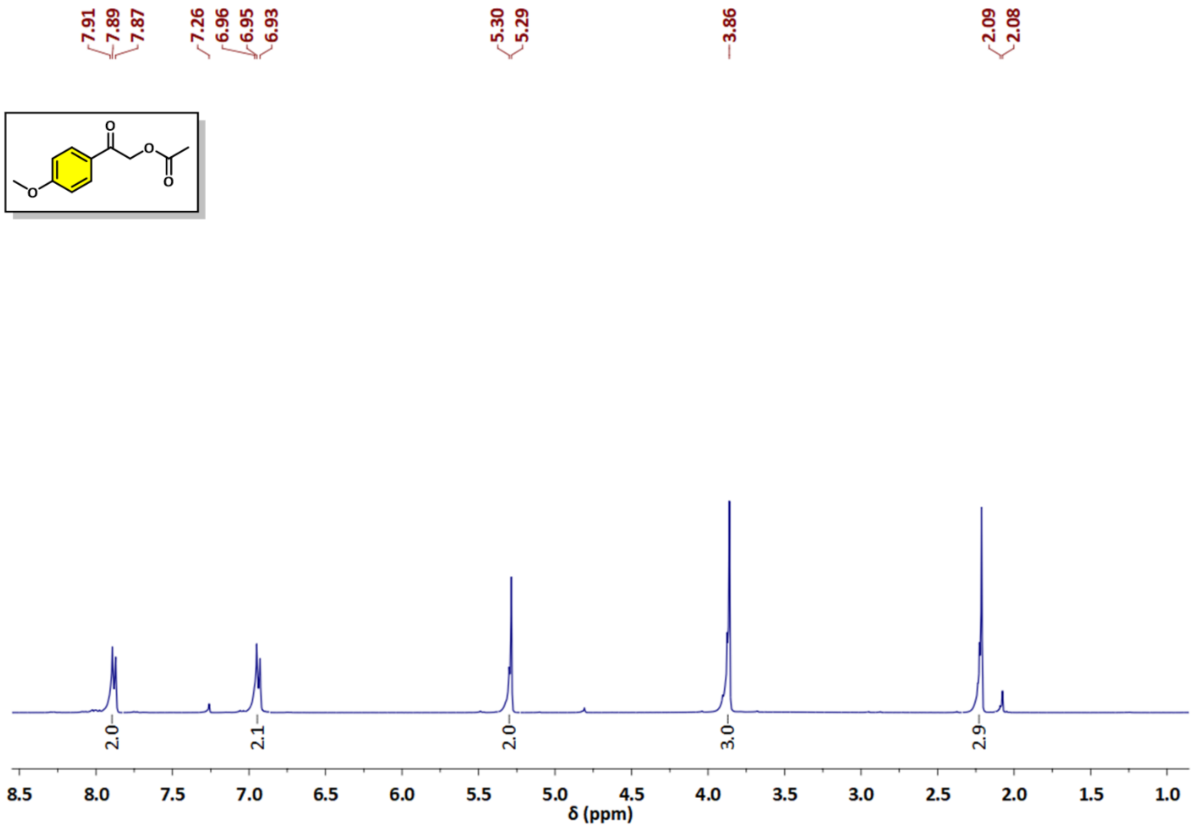
7.91
7.89
7.87
7.26
6.96
6.95
6.93
5.30
5.29
3.86
2.09
2.08
O
O
O
O
2.0
2.1
2.0
3.0
2.9
8.5 8.0 7.5 7.0 6.5 6.0 5.5 5.0 4.5 4.0 3.5 3.0 2.5 2.0 1.5 1.0
δ (ppm)

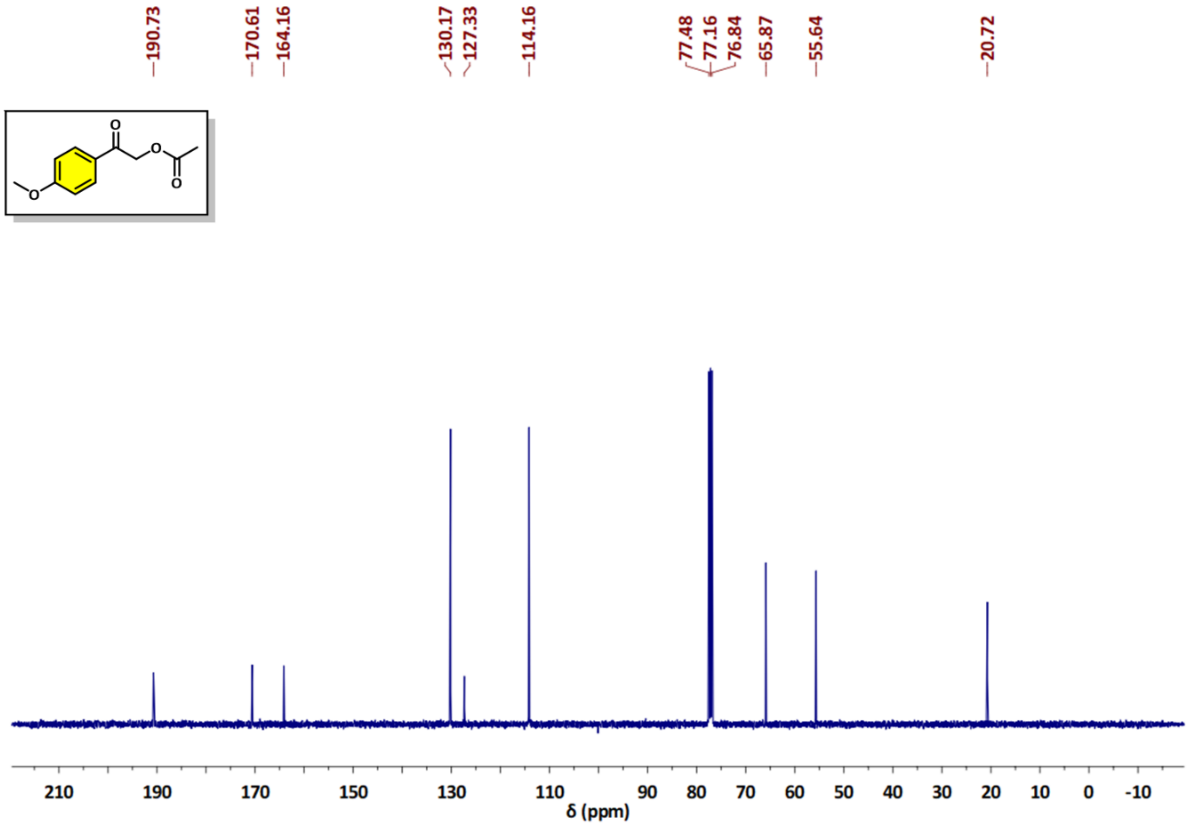
190.73
170.61
164.16
130.17
127.33
114.16
77.48
77.16
76.84
65.87
55.64
20.72
O
O
O
O
210 190 170 150 130 110 90 80 70 60 50 40 30 20 10 0 -10
δ (ppm)

Figure S6: ^{1}H and ^{13}C NMR of compound 2F.

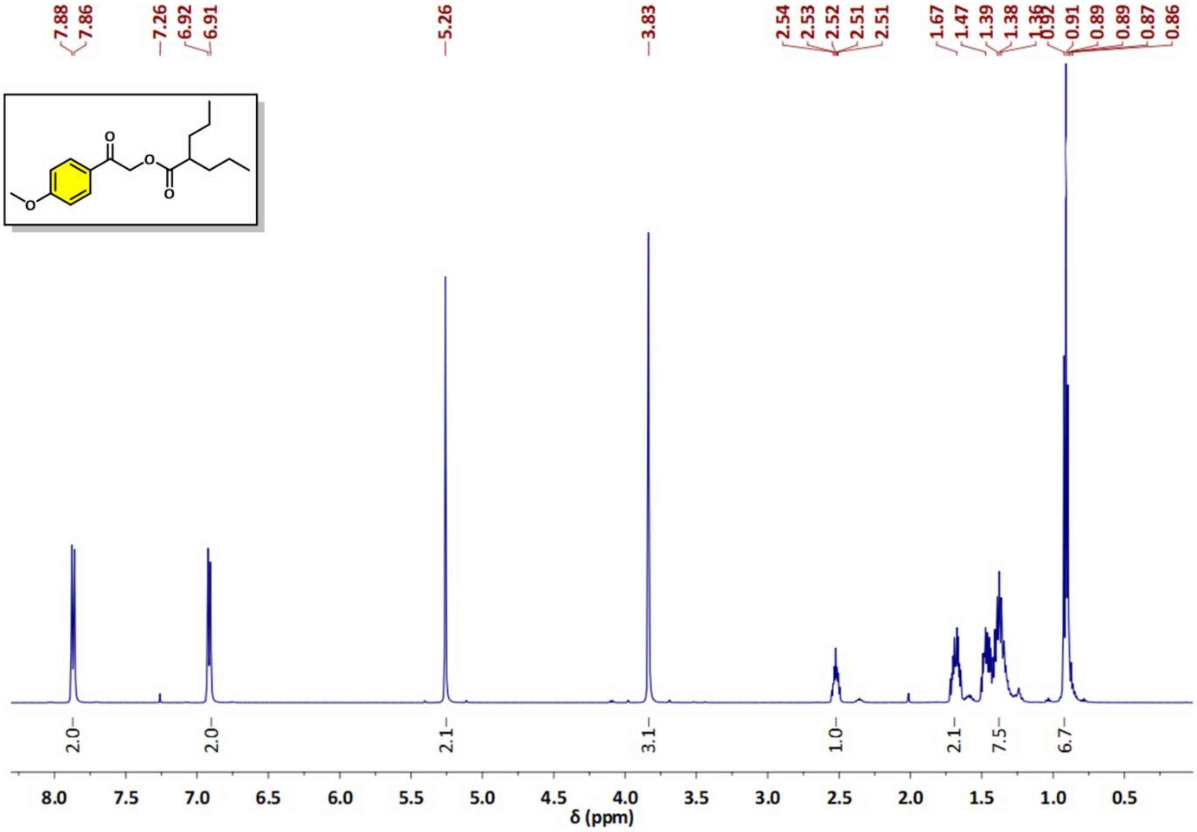

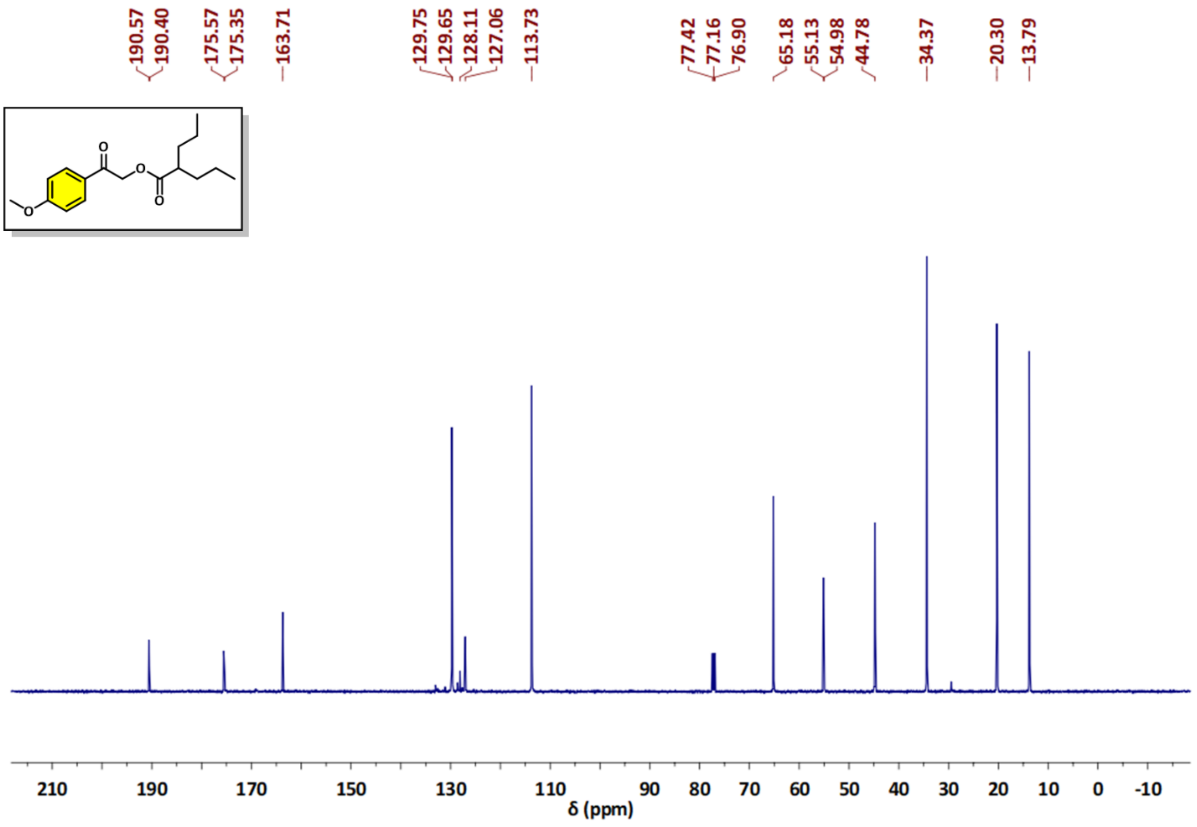

Figure S7: ^{1}H and ^{13}C NMR of compound 2D.

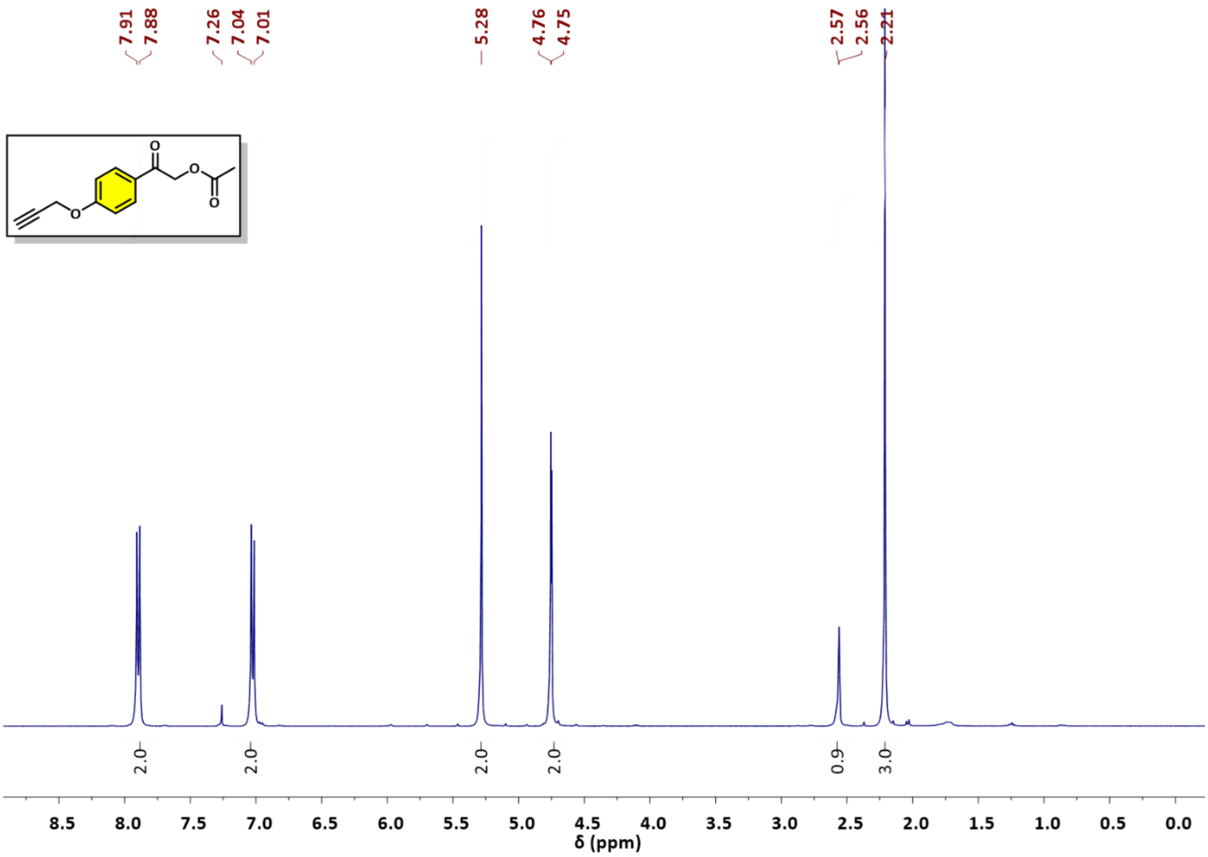

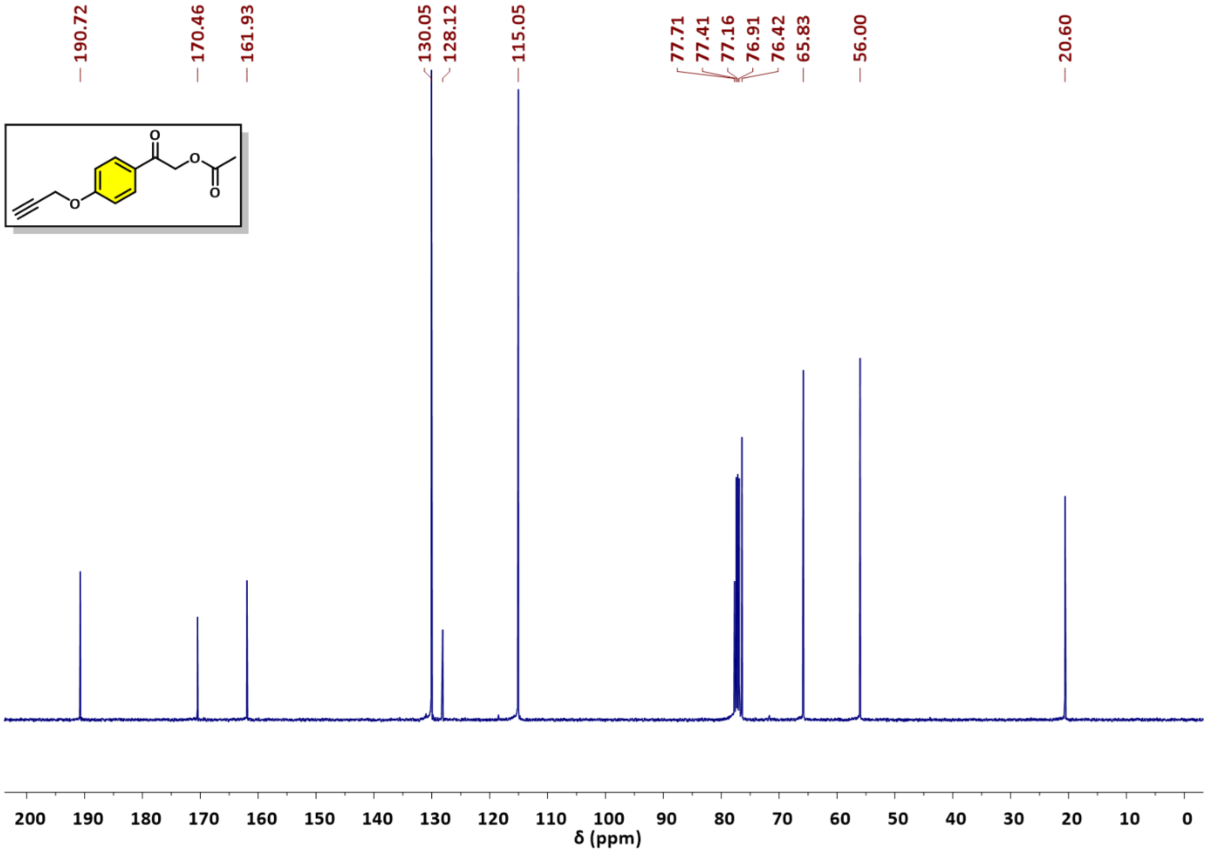

Figure S8: ^{1}H and ^{13}C NMR of compound 2E.

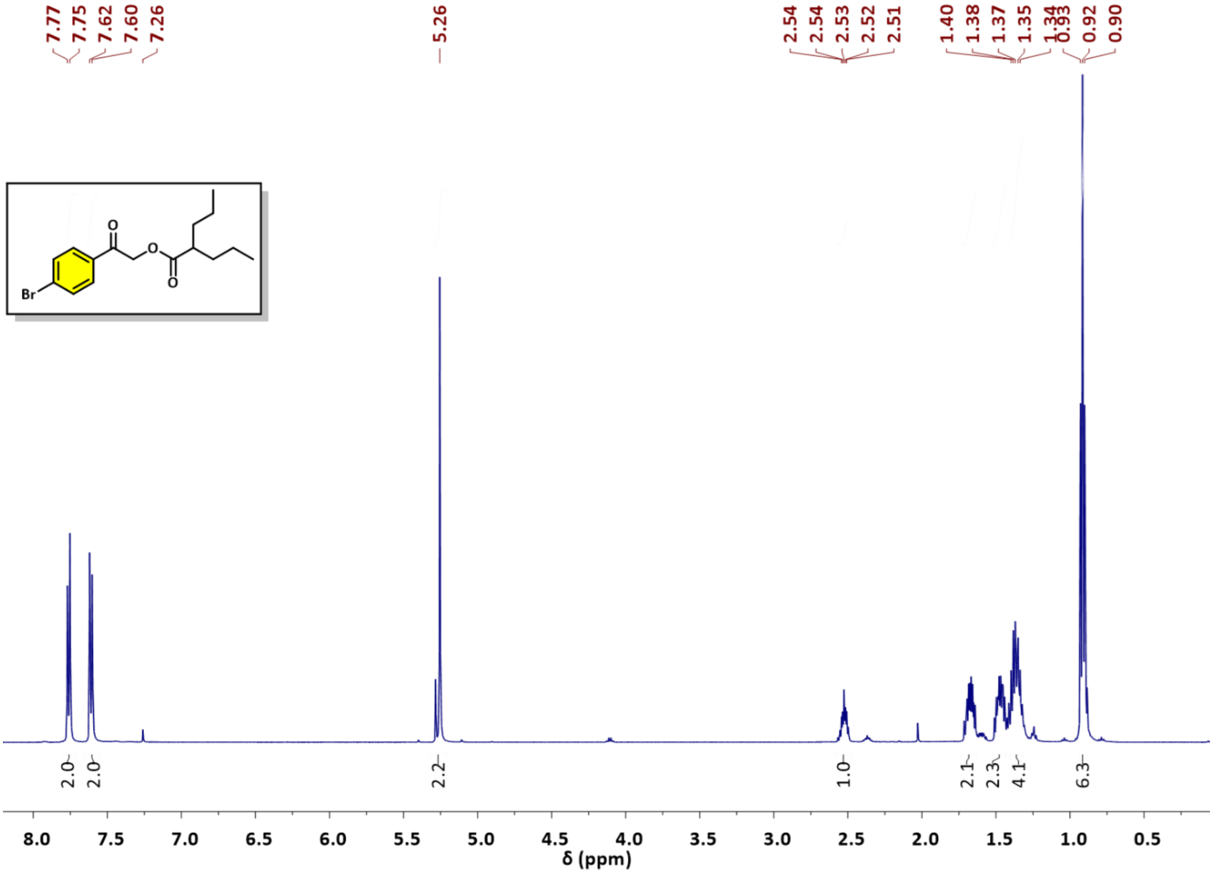

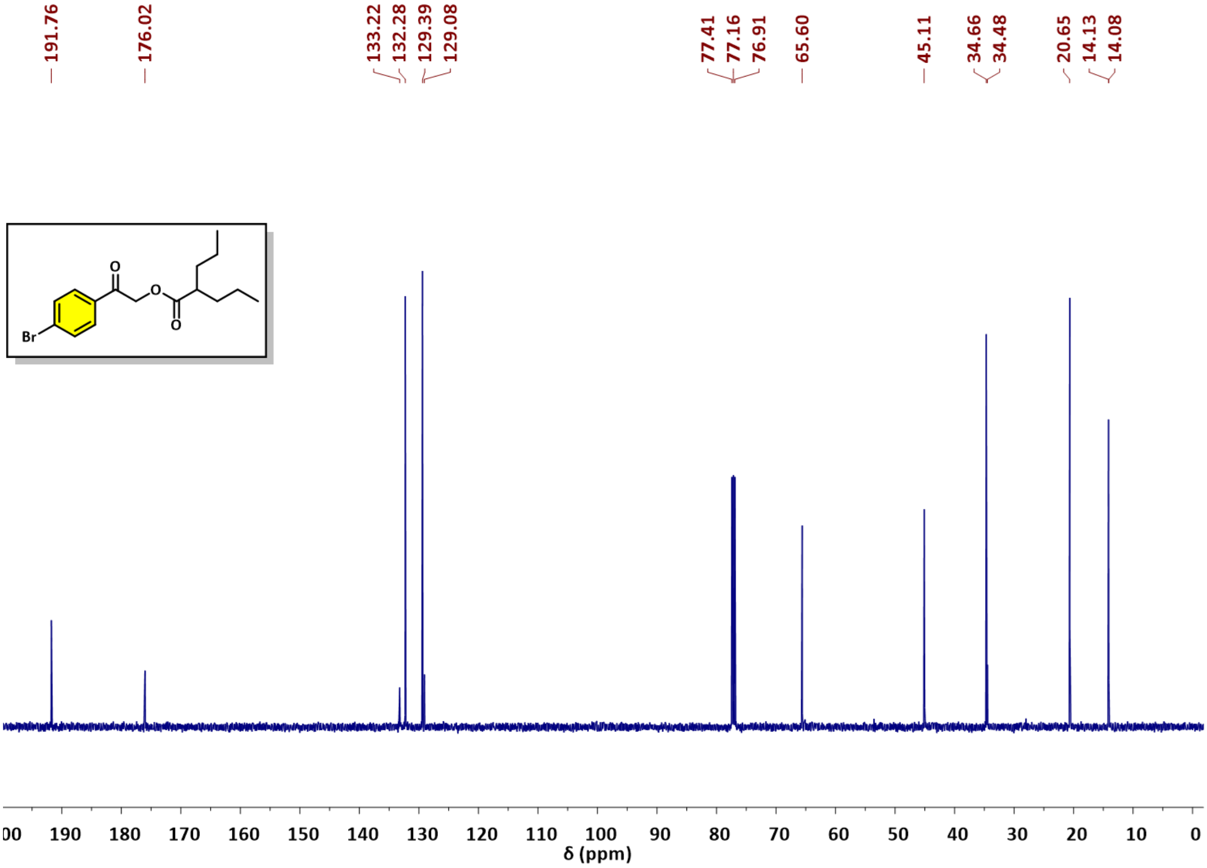

Figure S9: ^{1}H and ^{13}C NMR of compound 2G.

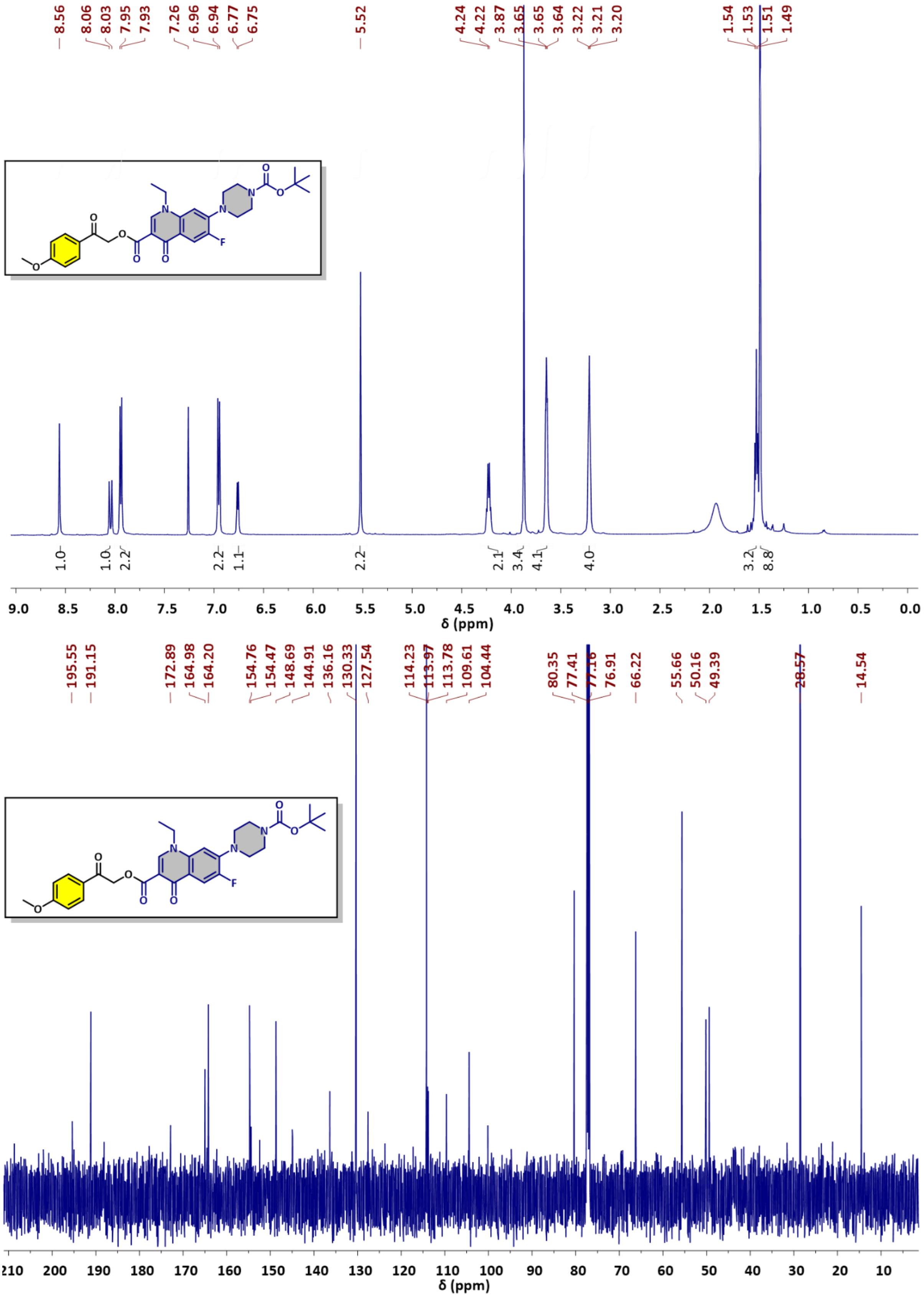

Figure S10: ^{1}H and ^{13}C NMR of photocage 3A.

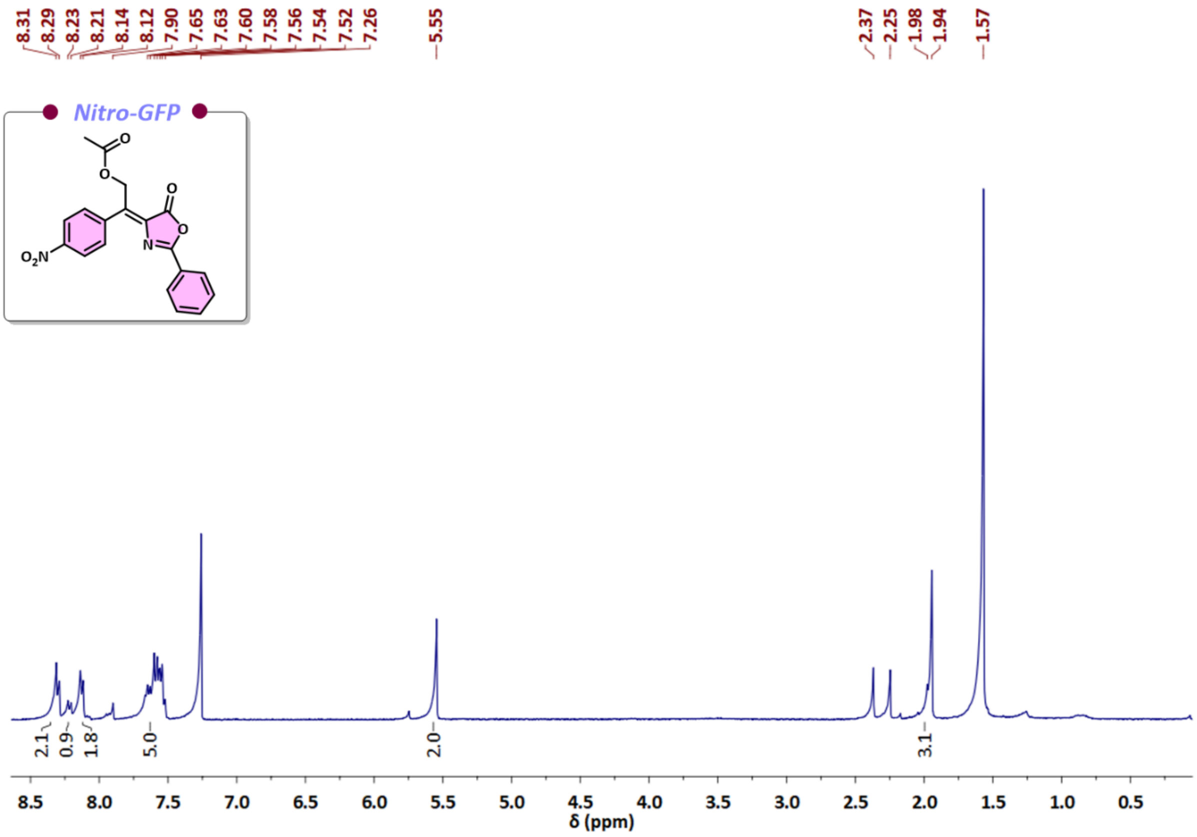

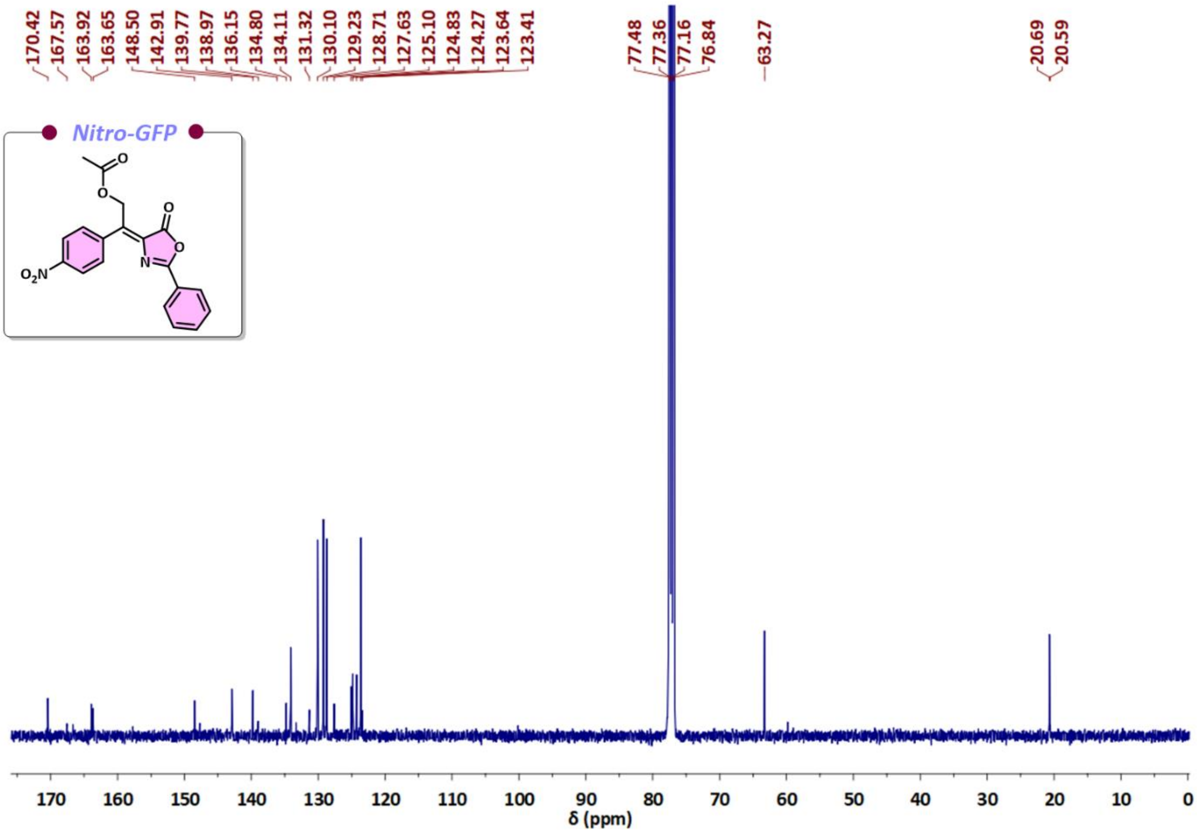

Figure S11: ^{1}H and ^{13}C NMR of photocage 3B.

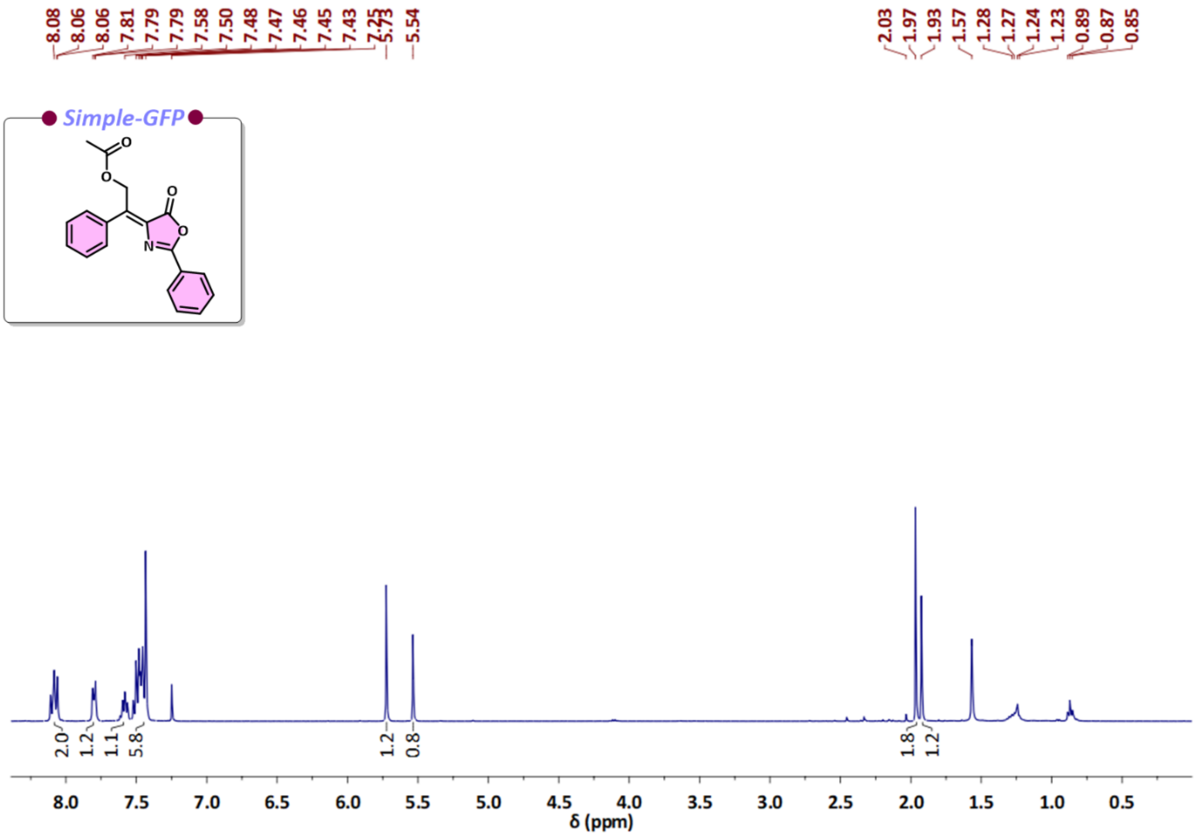

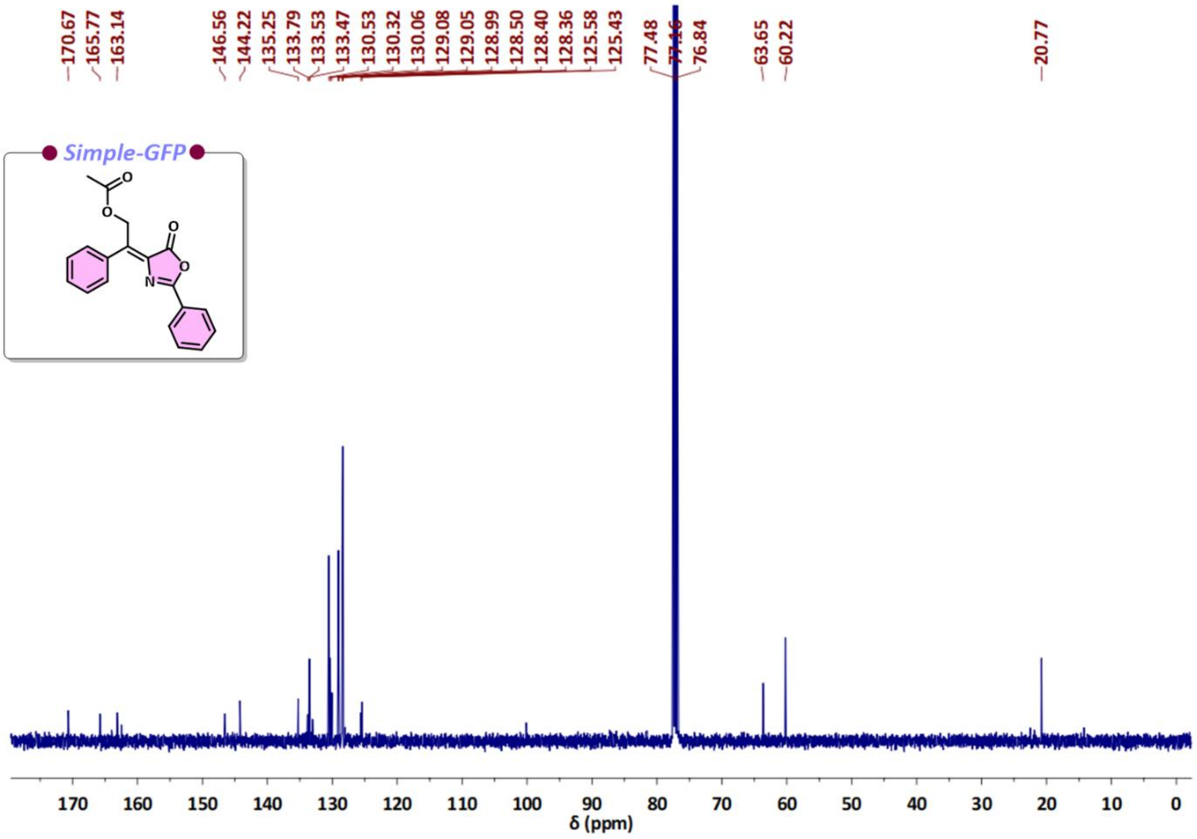

Figure S12: ^{1}H and ^{13}C NMR of photocage 3C.

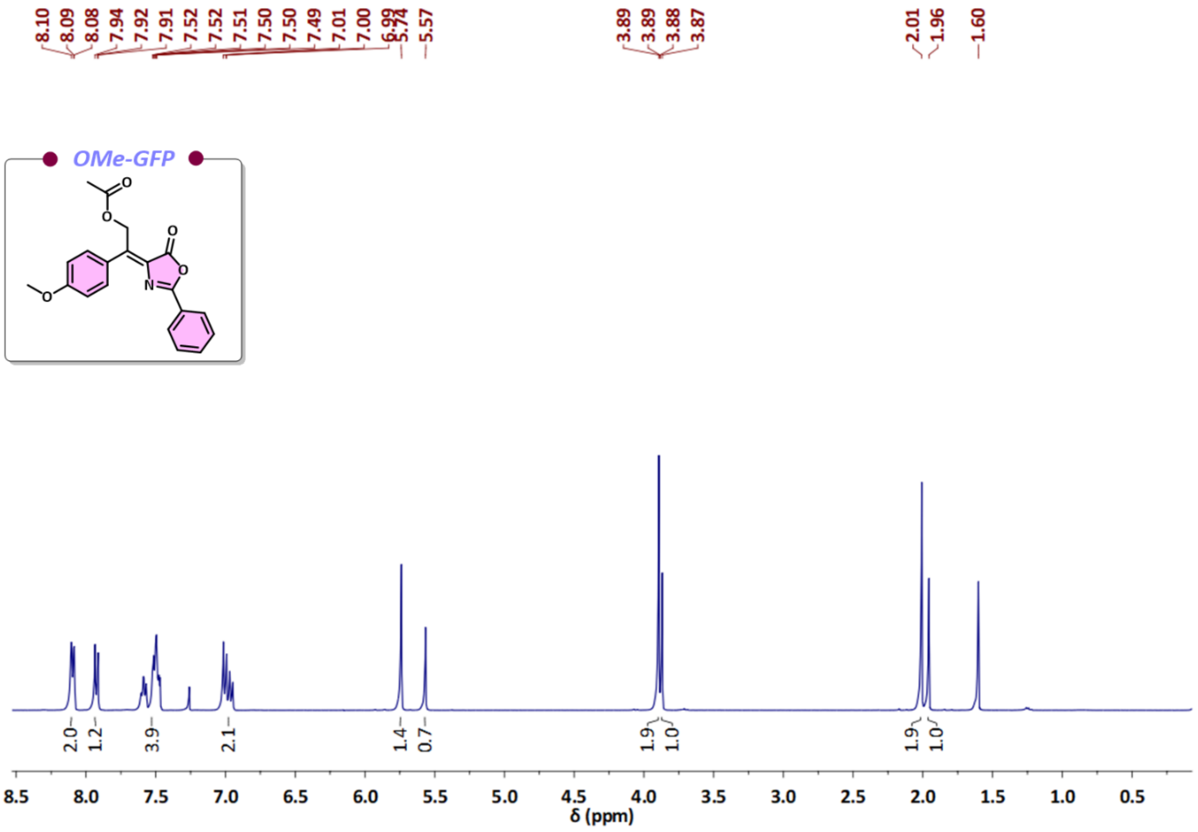

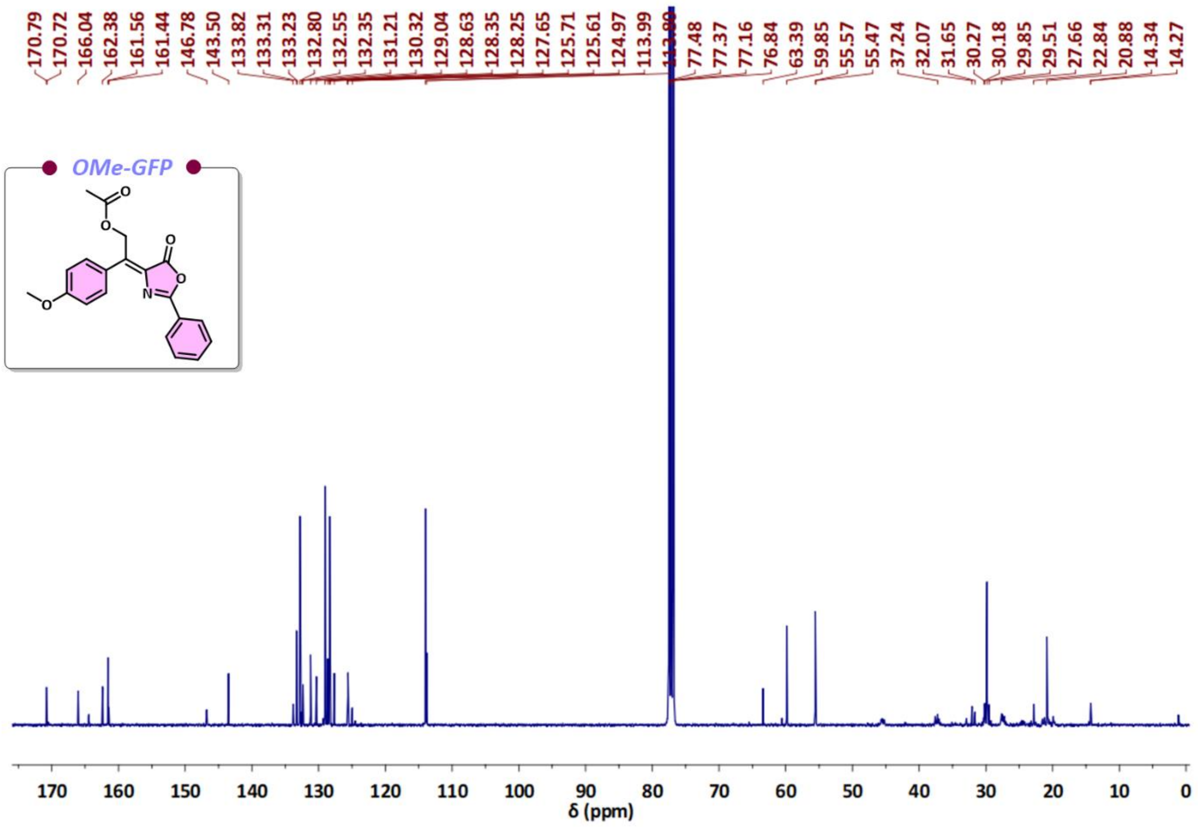

Figure S13: ^{1}H and ^{13}C NMR of photocage 3F.

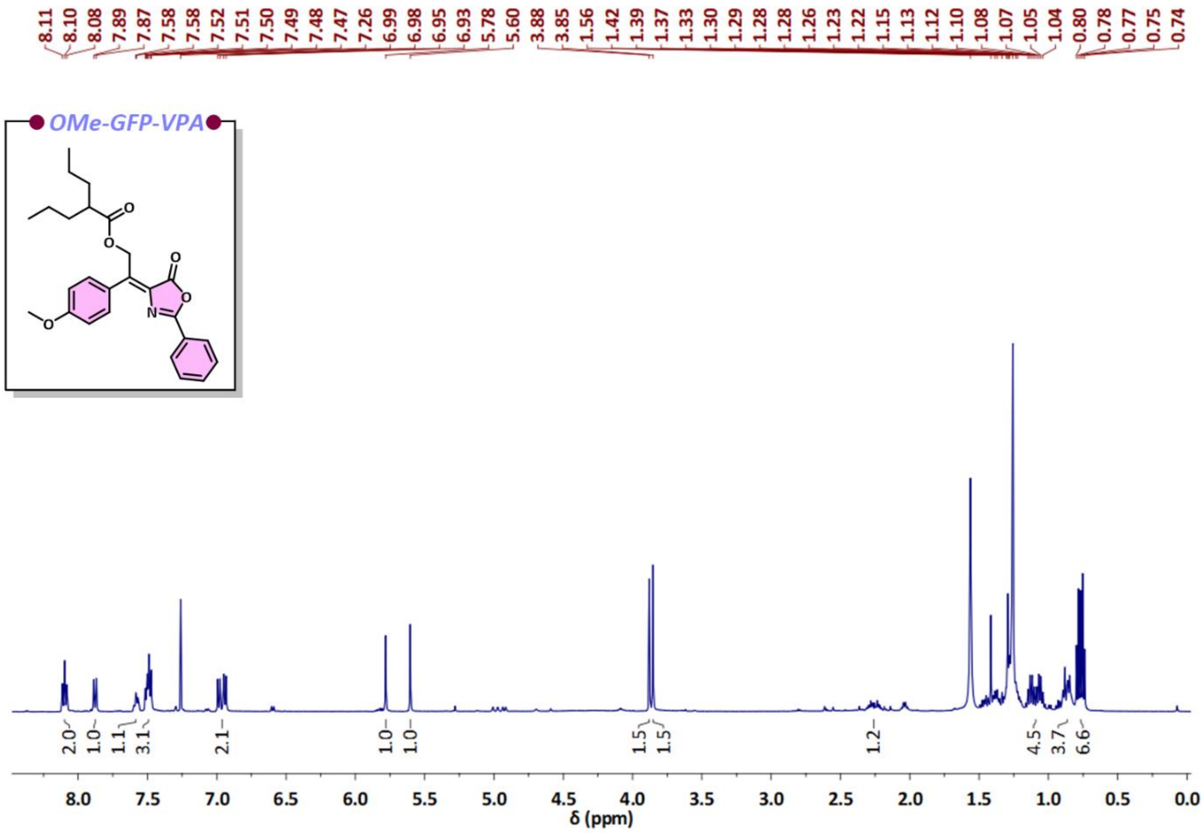

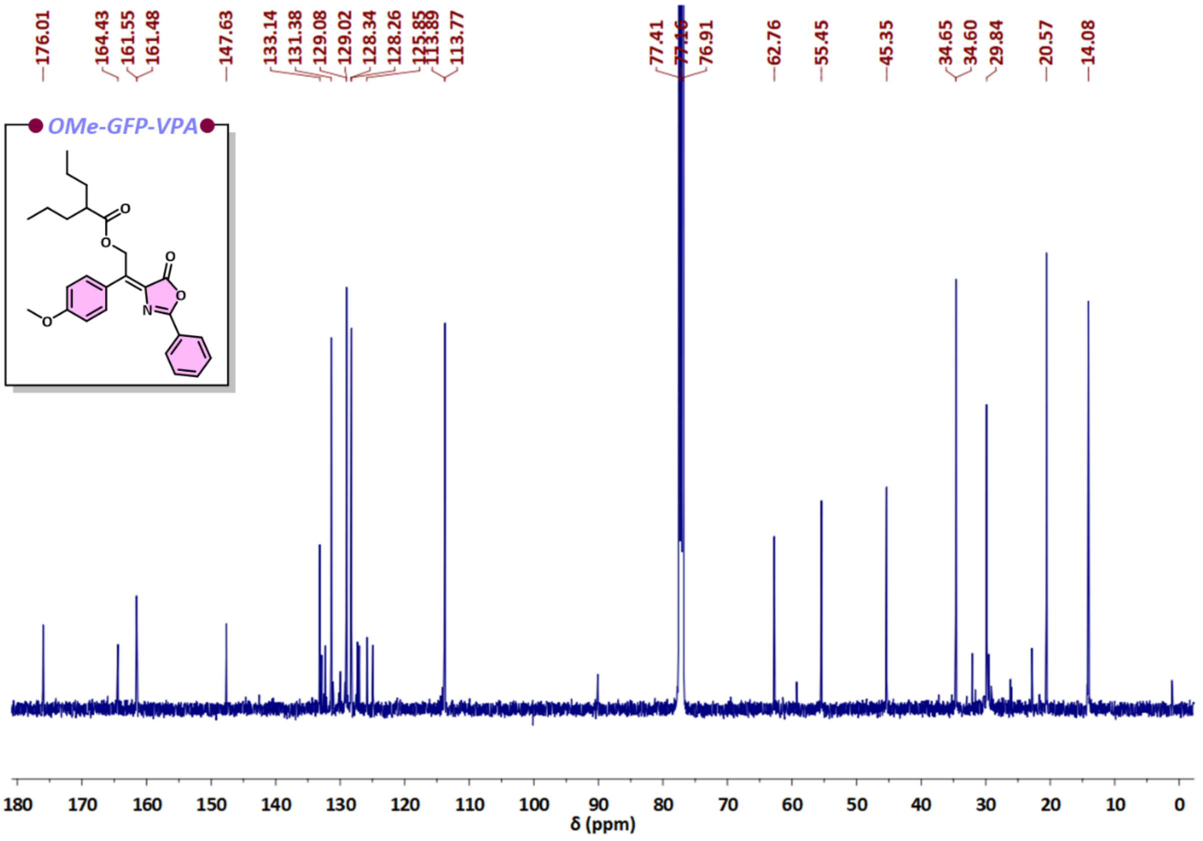

Figure S14: ^{1}H and ^{13}C NMR of photocage 3D.

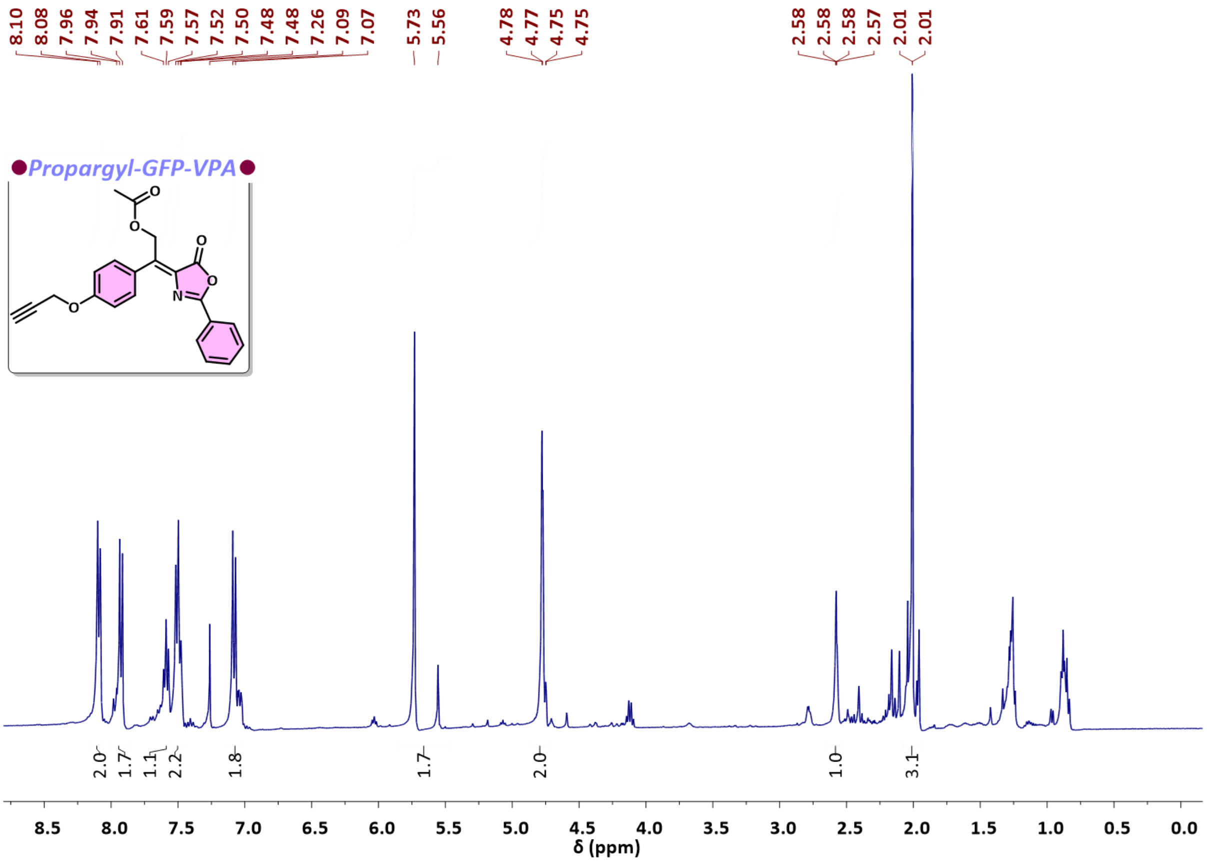

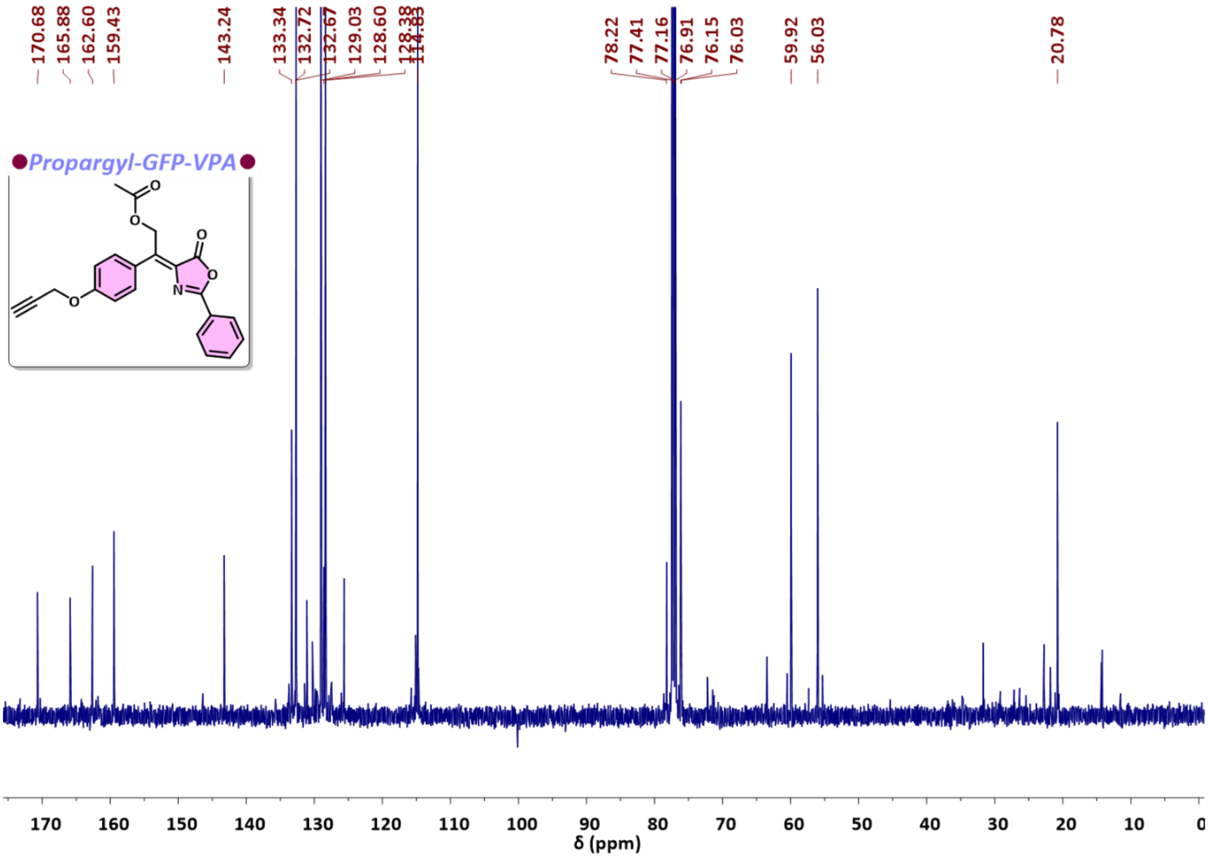

Figure S15: ^{1}H and ^{13}C NMR of photocage 3E.

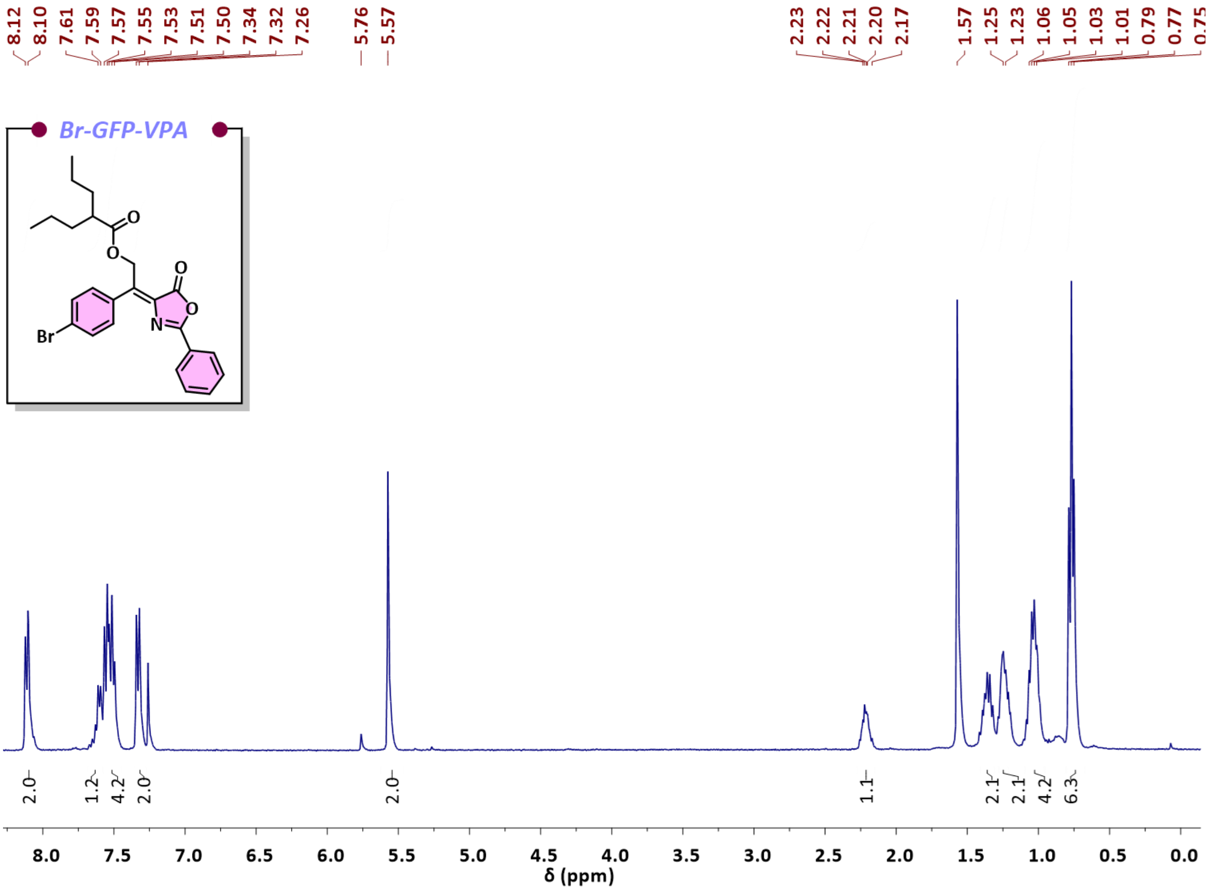

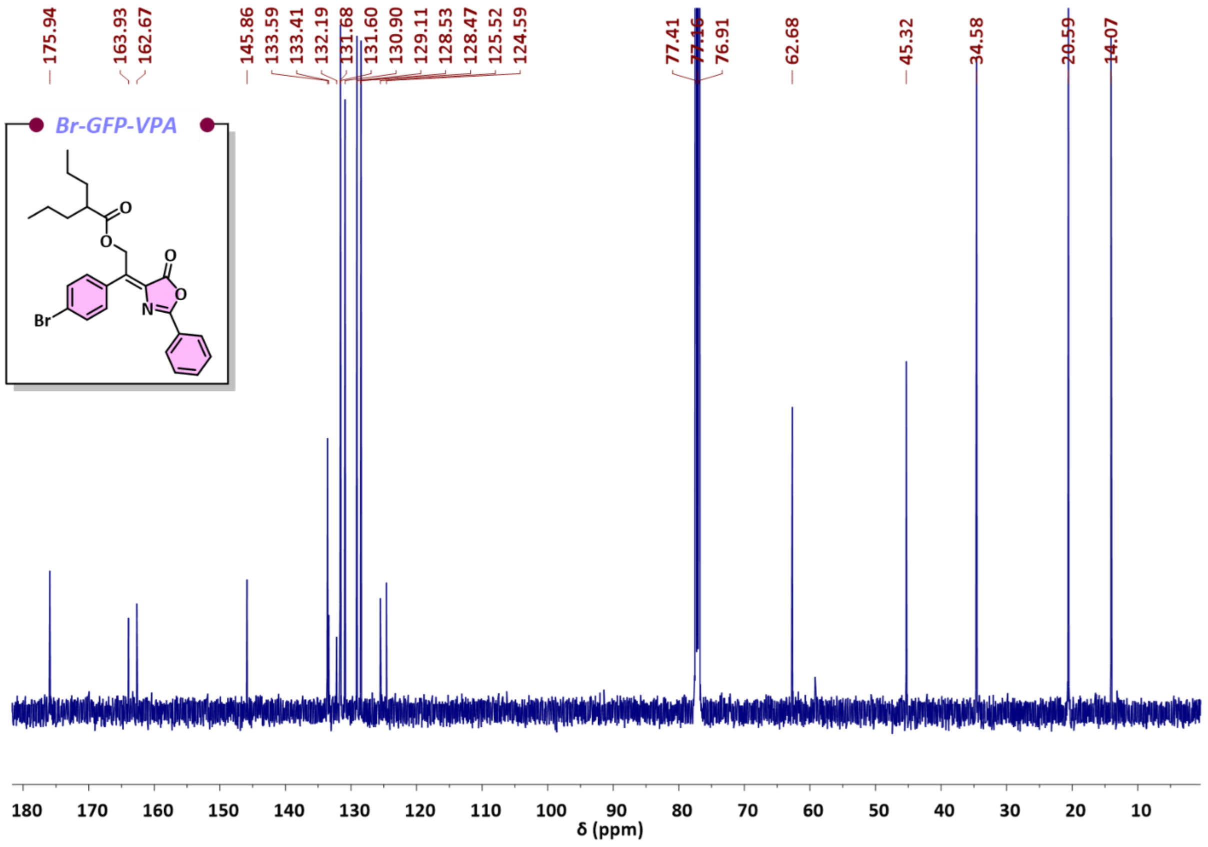

Figure S16: ^{1}H and ^{13}C NMR of photocage 6.

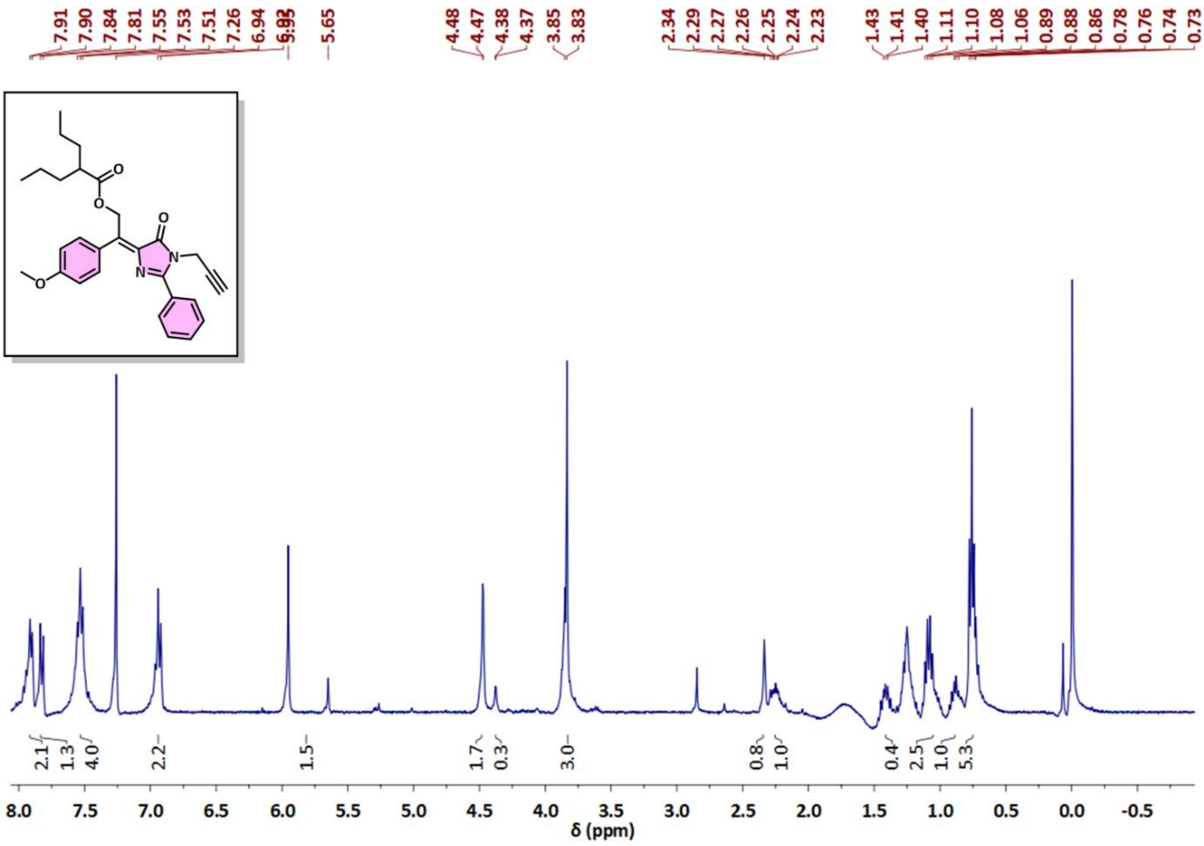

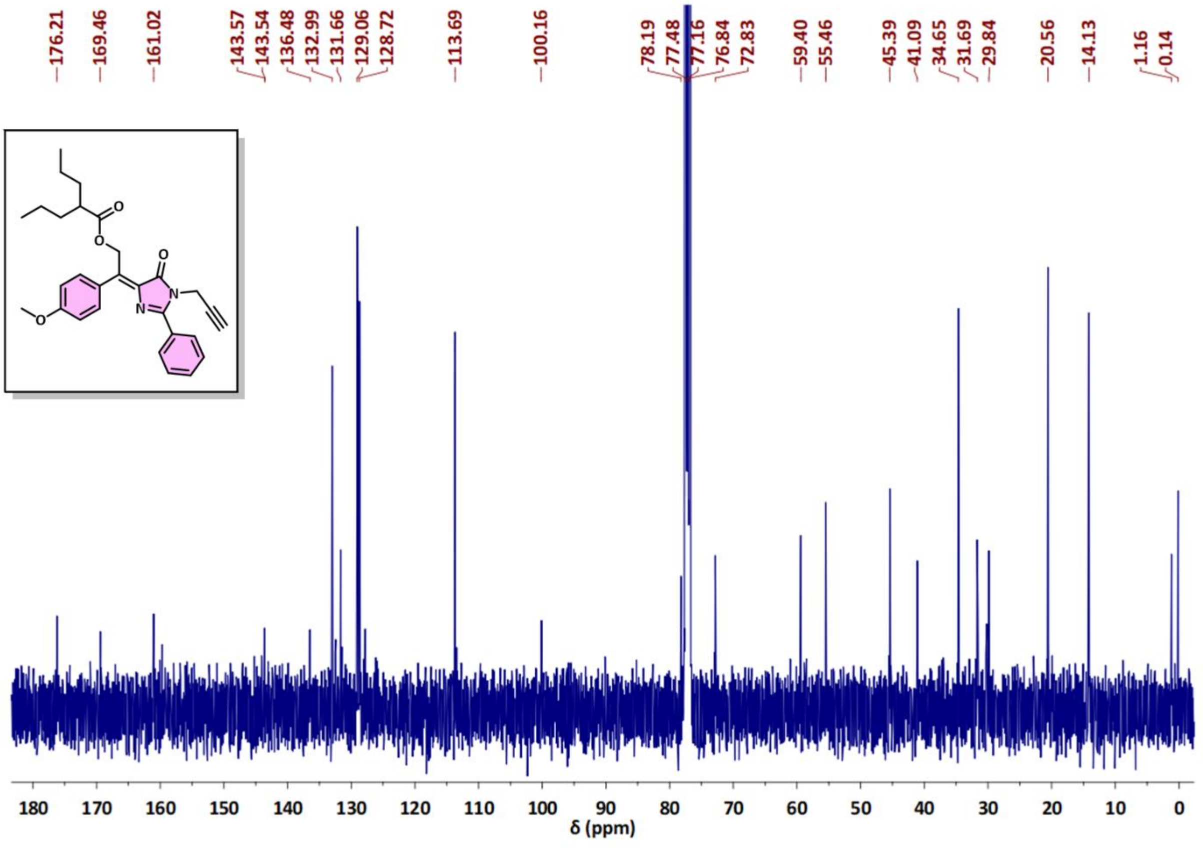

Figure S17: ¹H NMR of compound 6b.

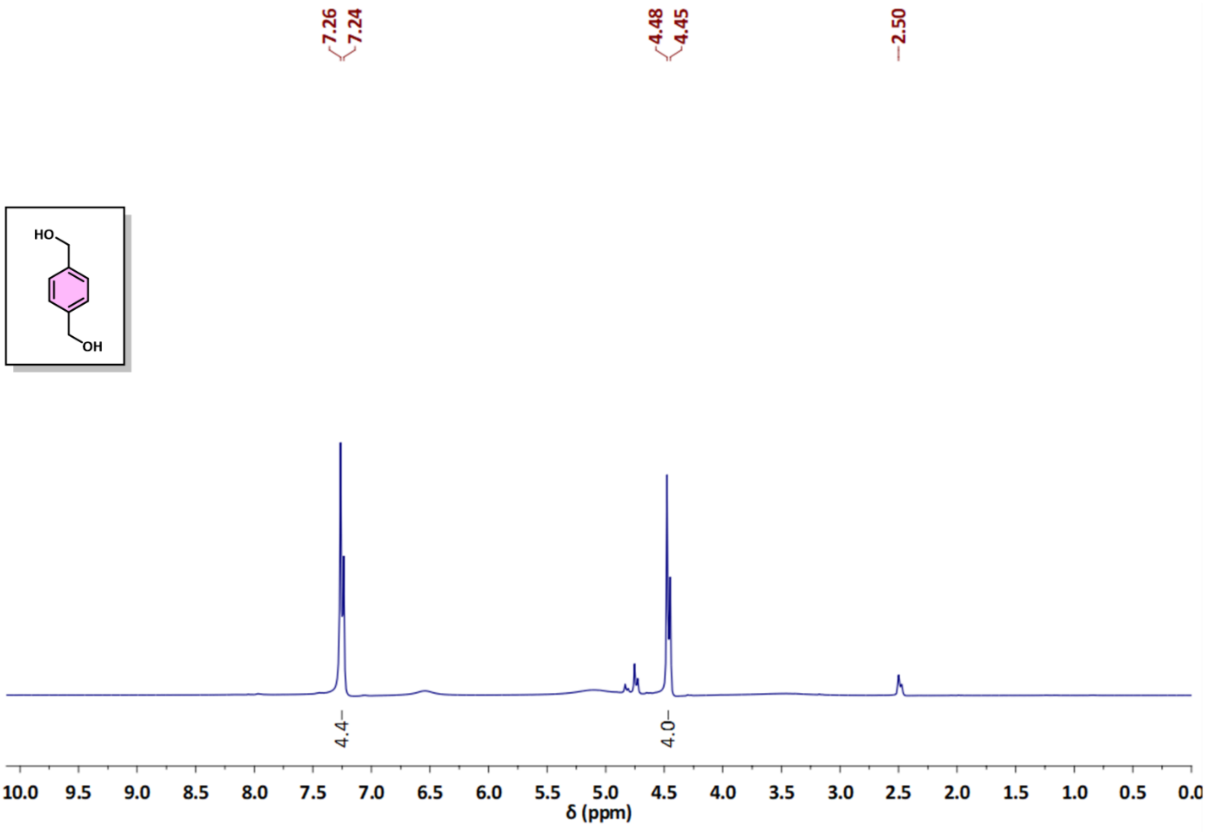

Figure S18: ^{1}H and ^{13}C NMR of compound 6c.

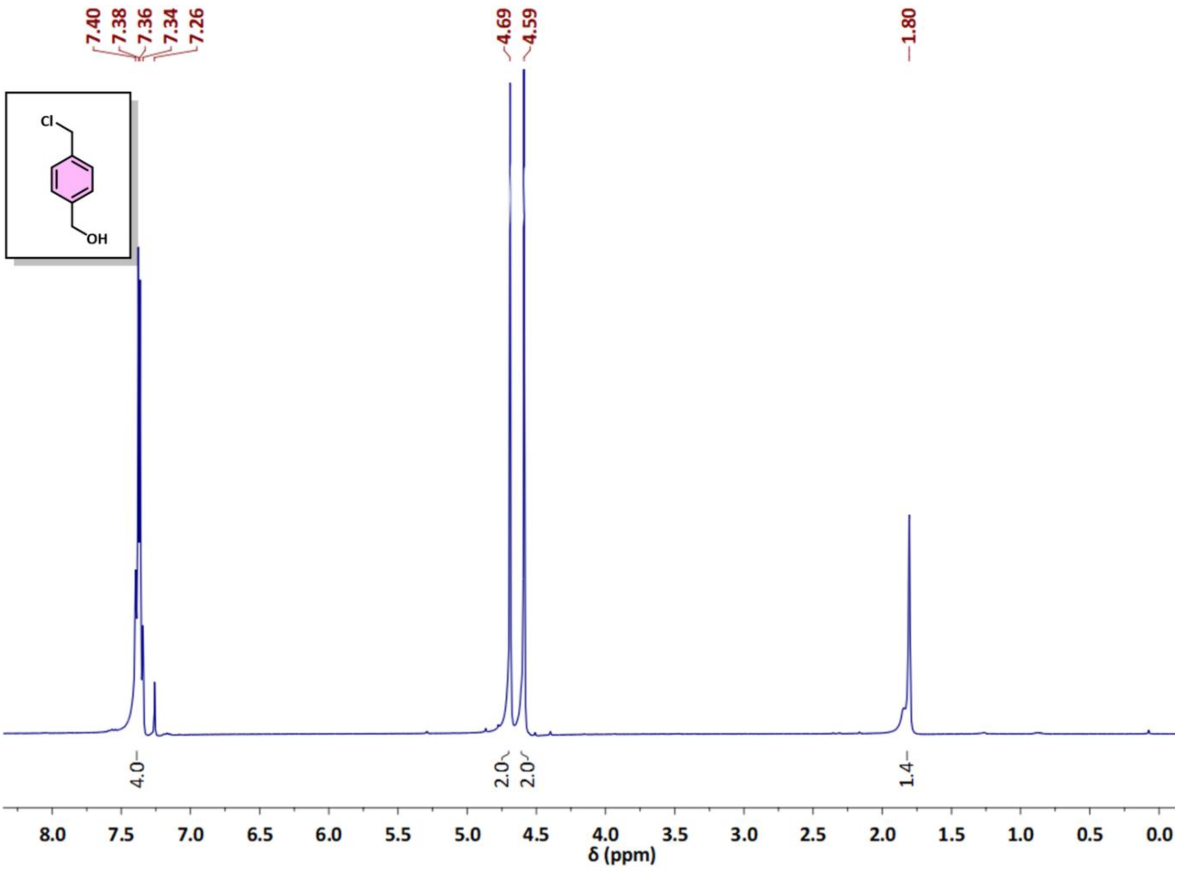

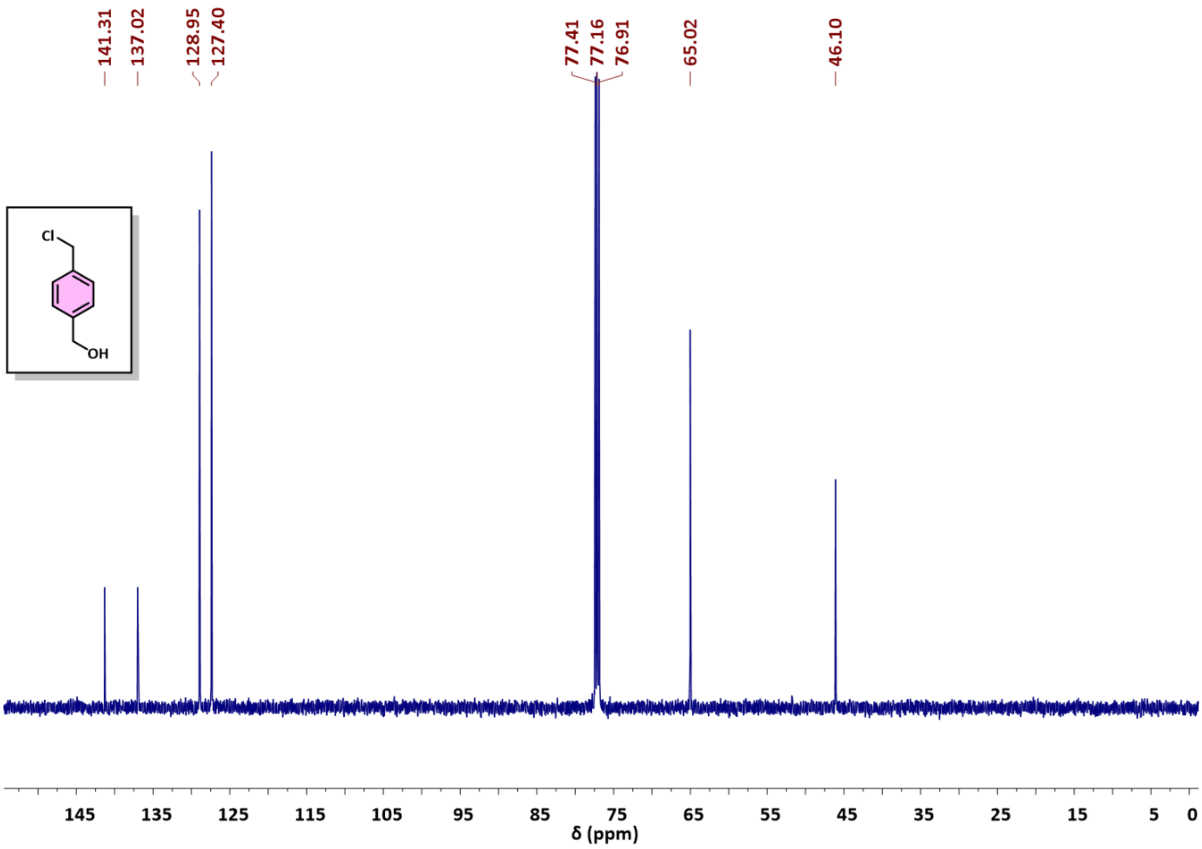

Figure S19: ^{1}H and ^{13}C NMR of compound 6d.

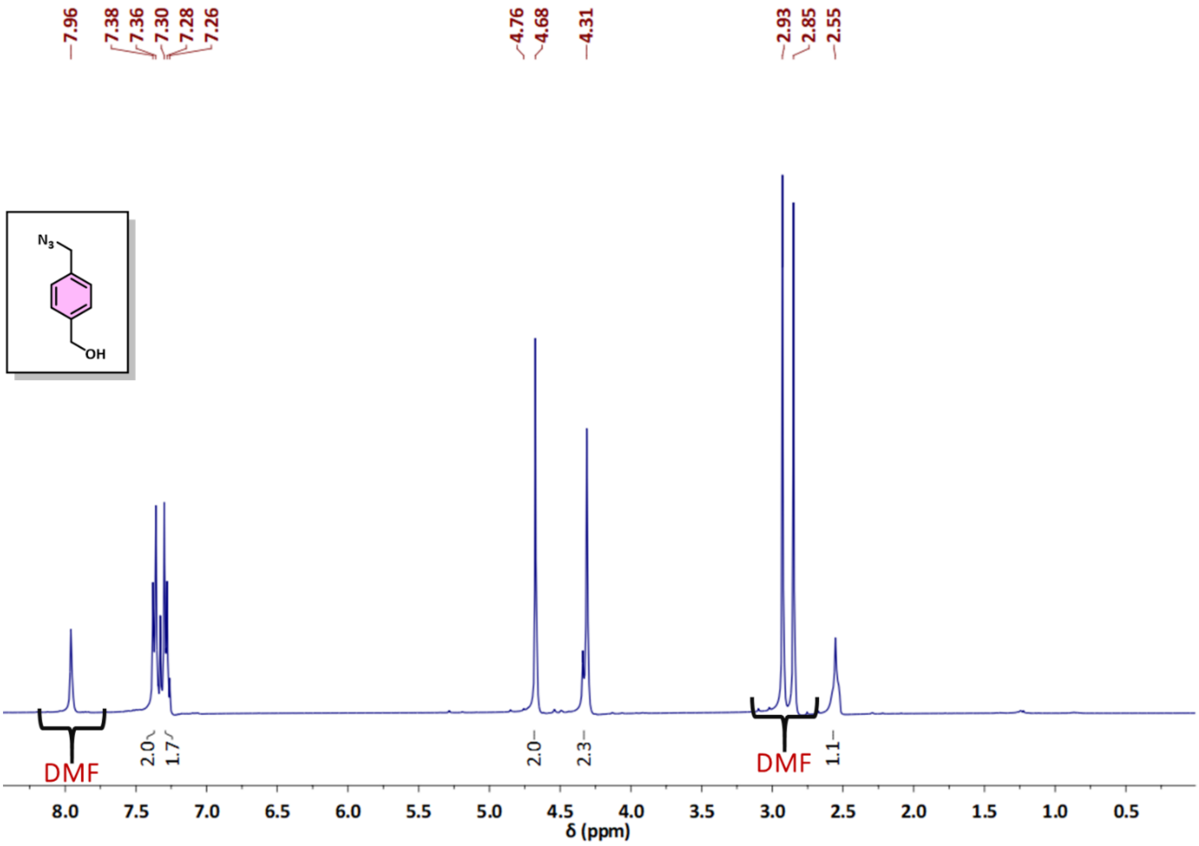

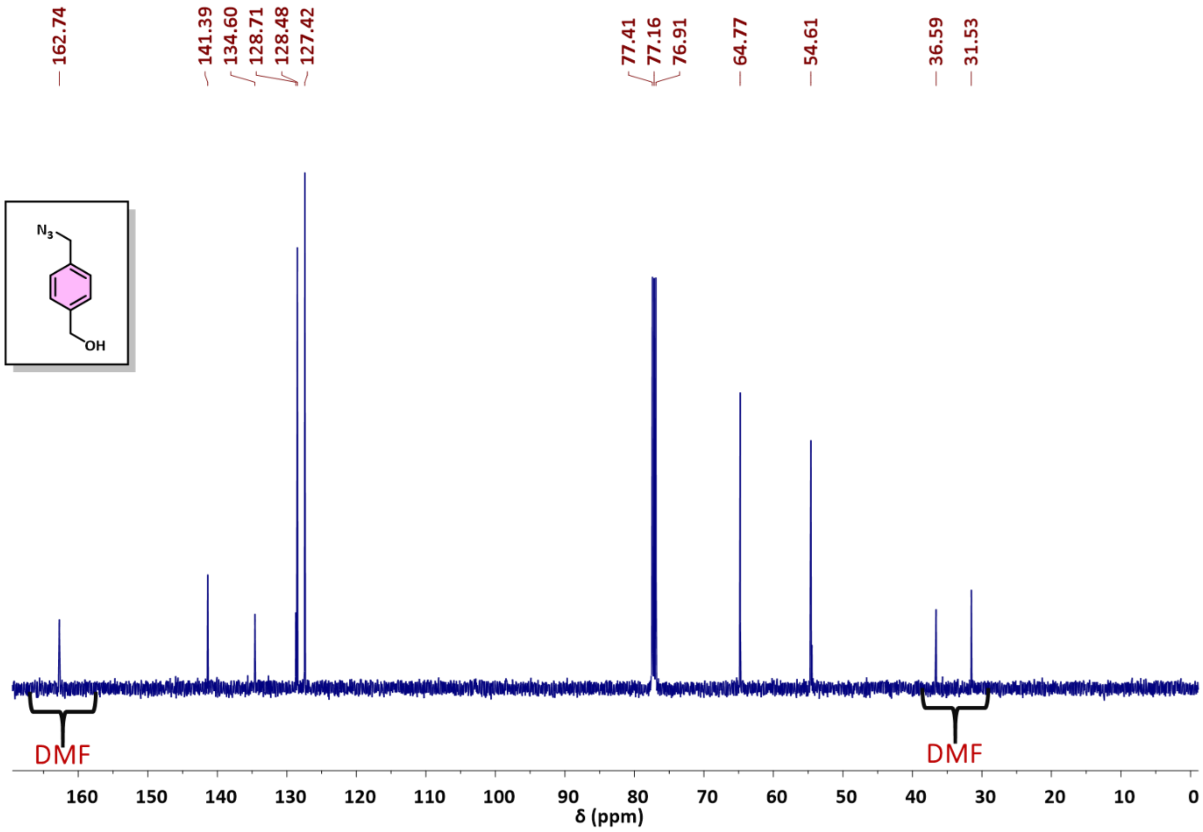

Figure S20: ^{1}H and ^{13}C NMR of compound 6f.

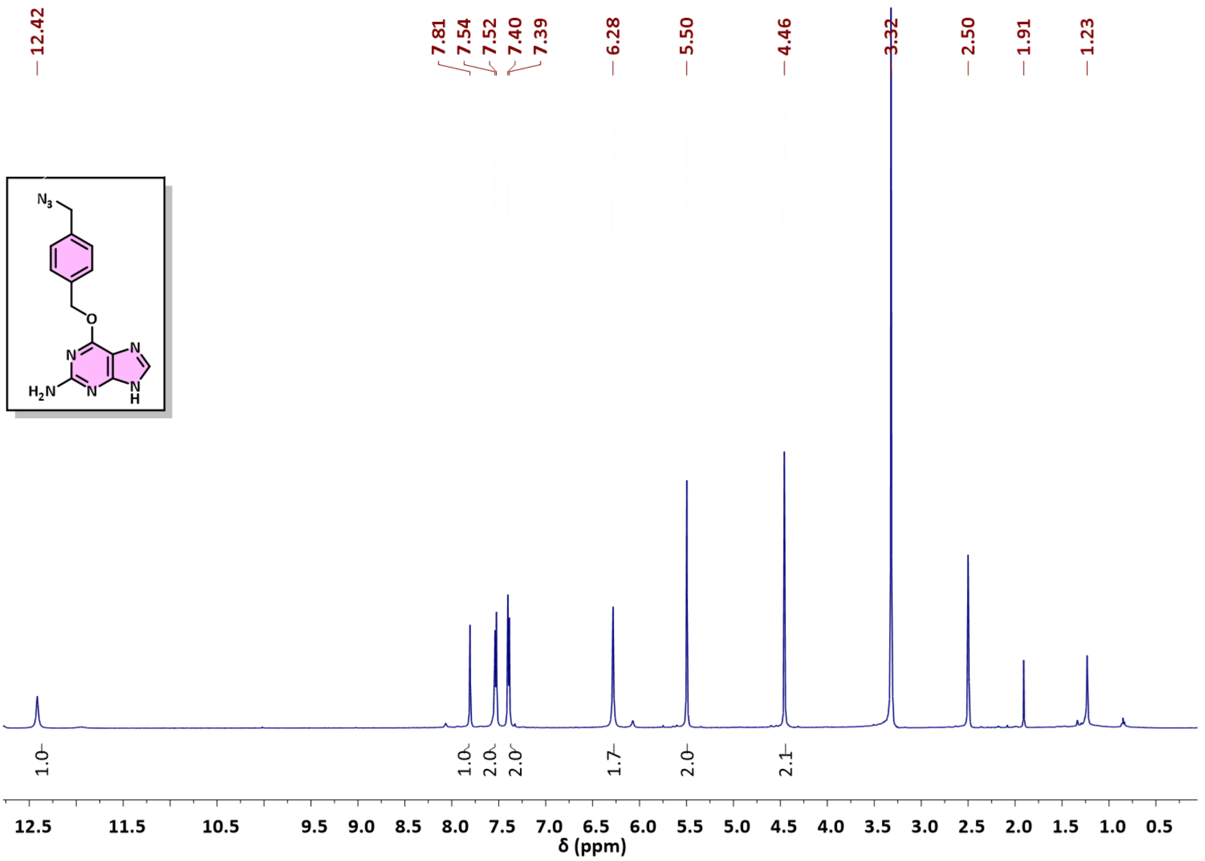

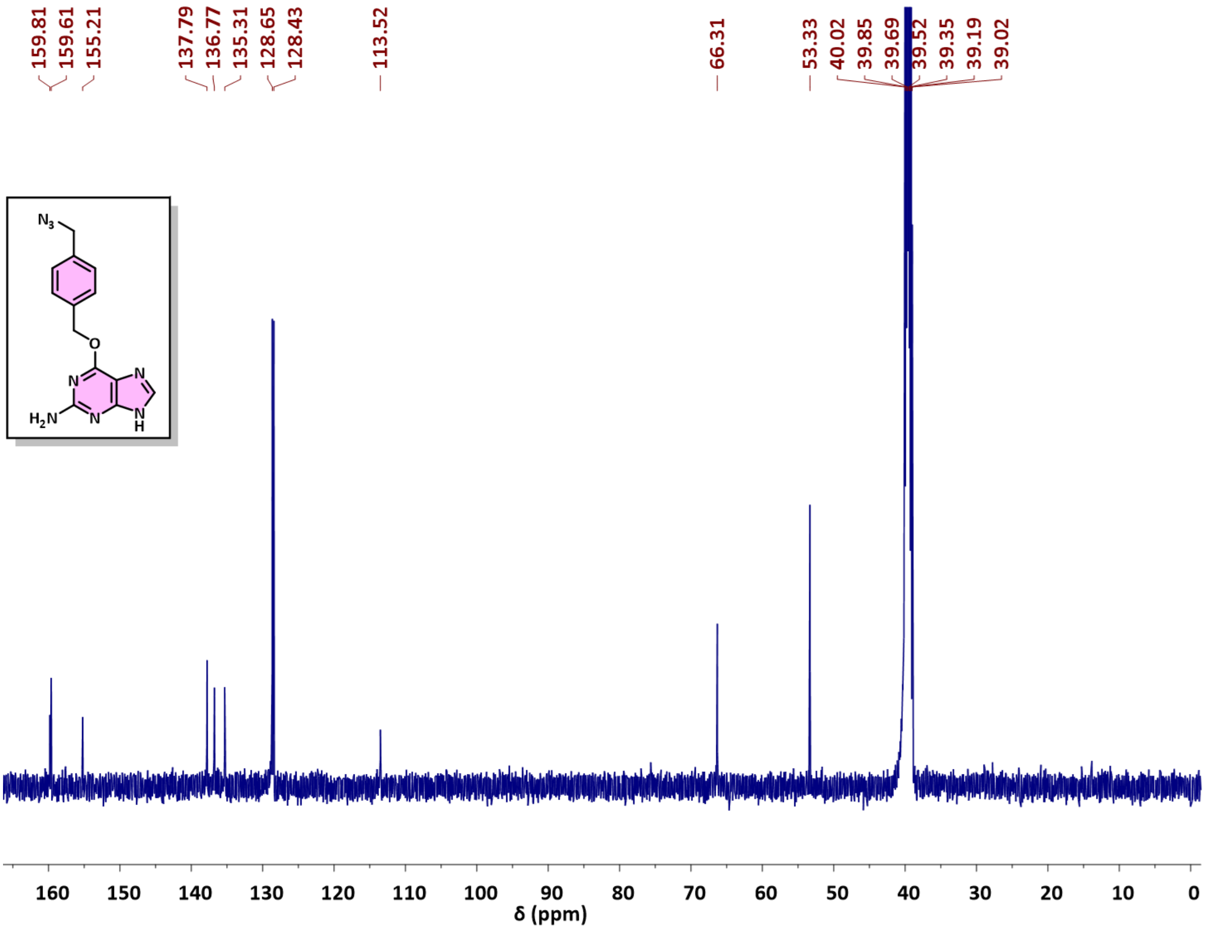

Figure S21: ^{1}H and ^{13}C NMR of phenalenone (reference singlet oxygen generator).

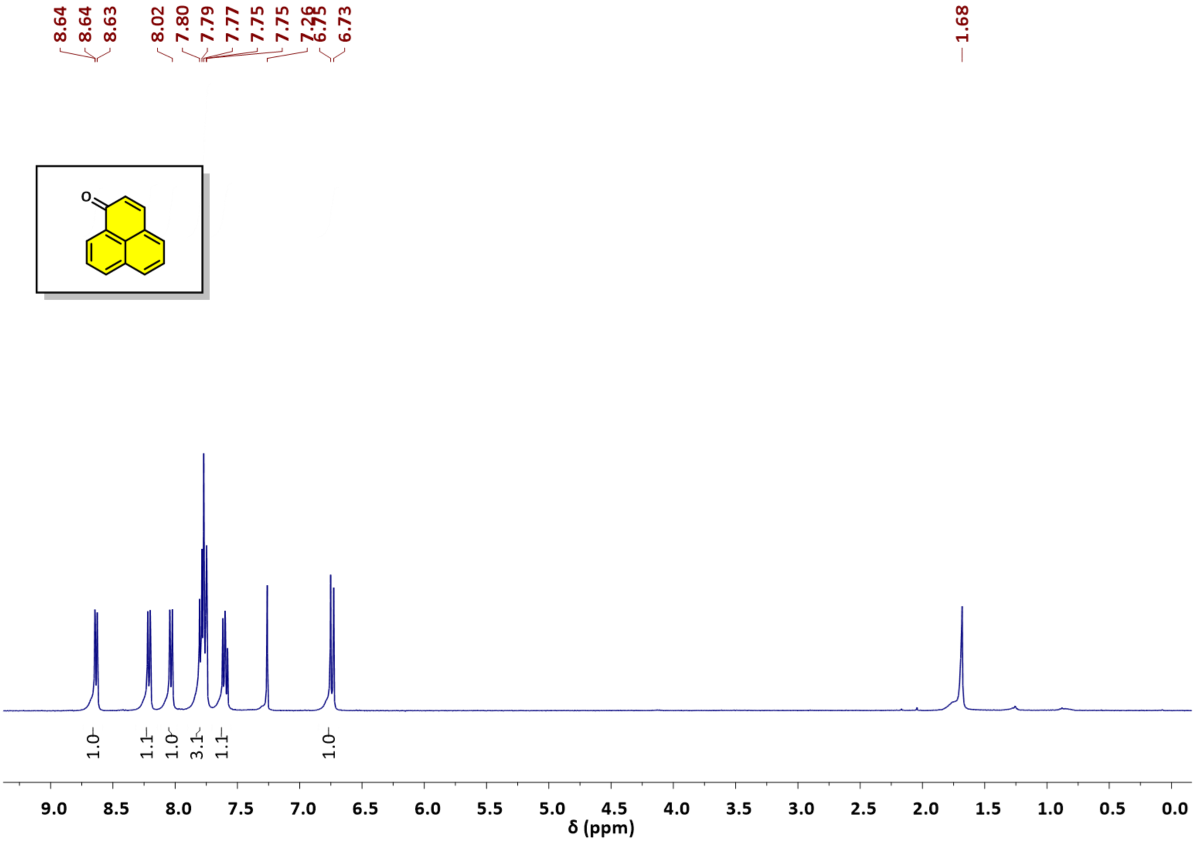

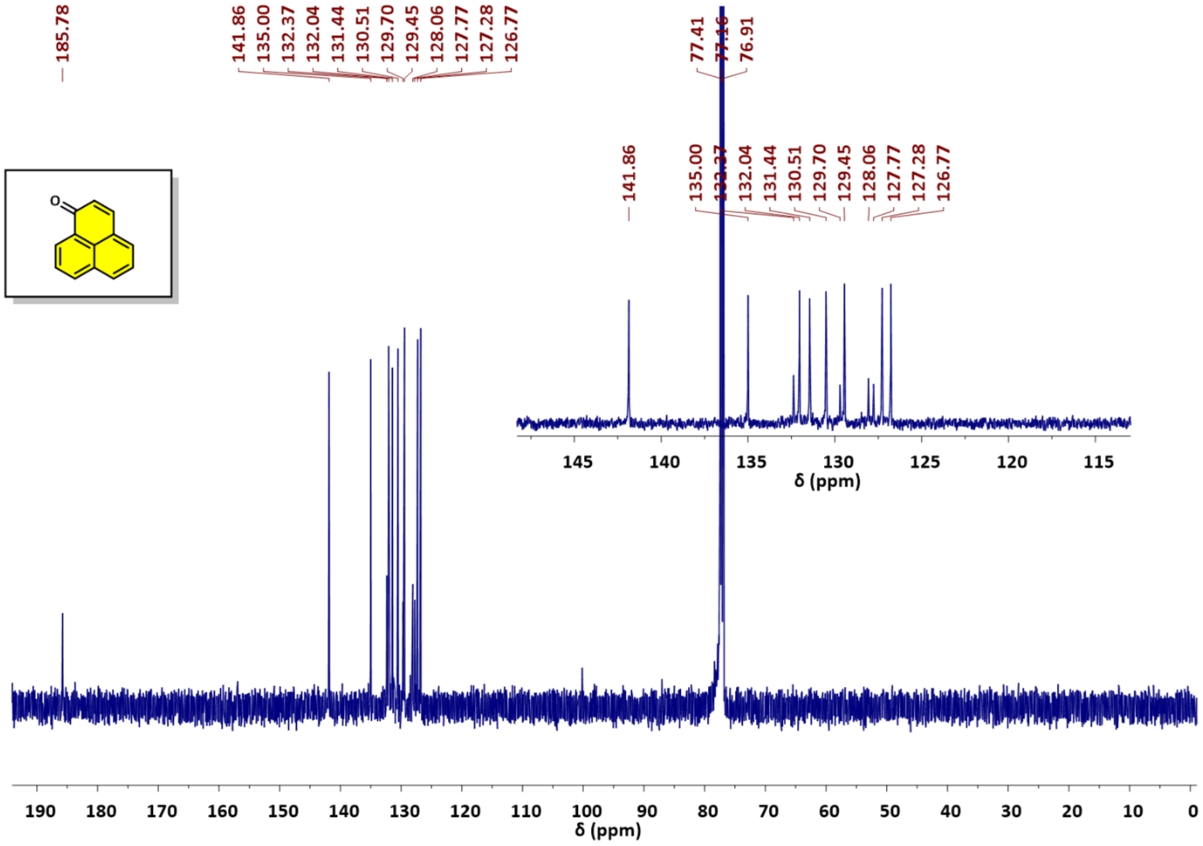

Figure S22: ^{1}H and ^{13}C NMR of Phenalenone.

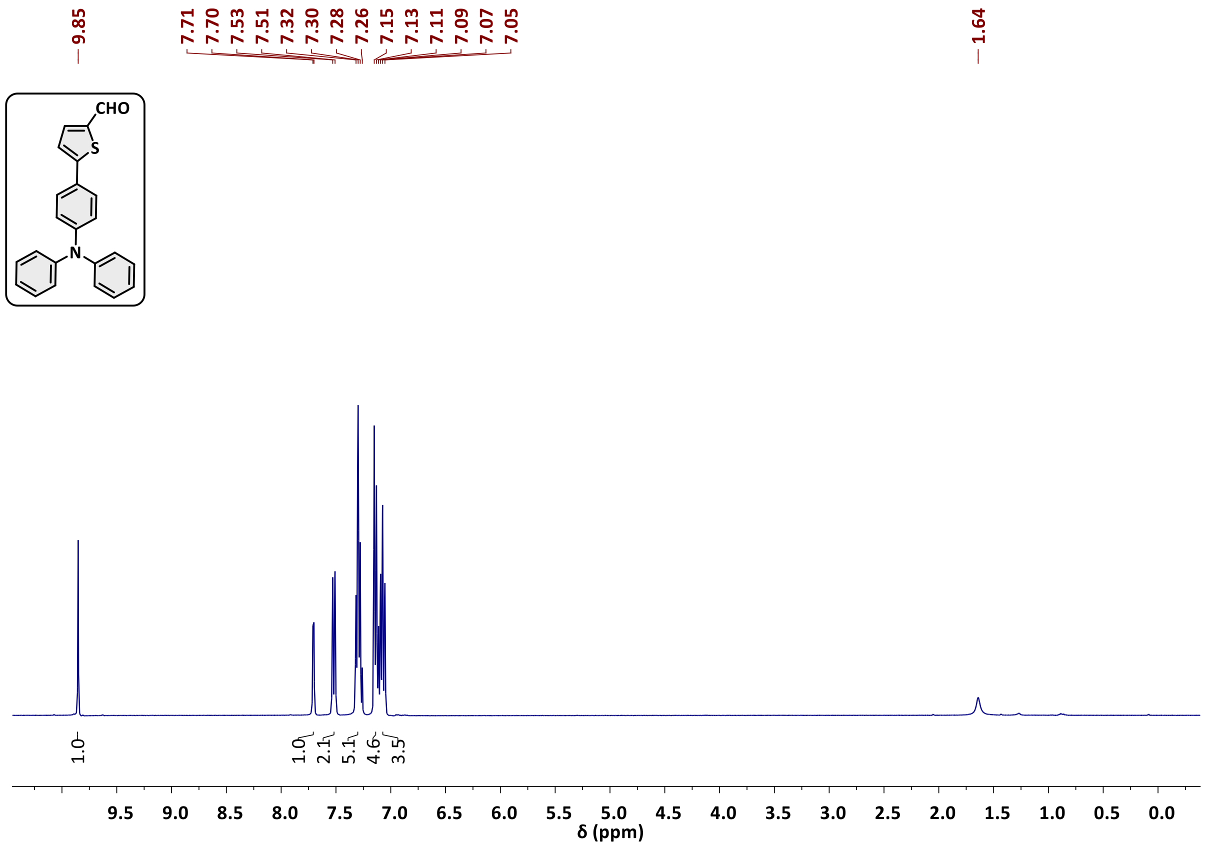

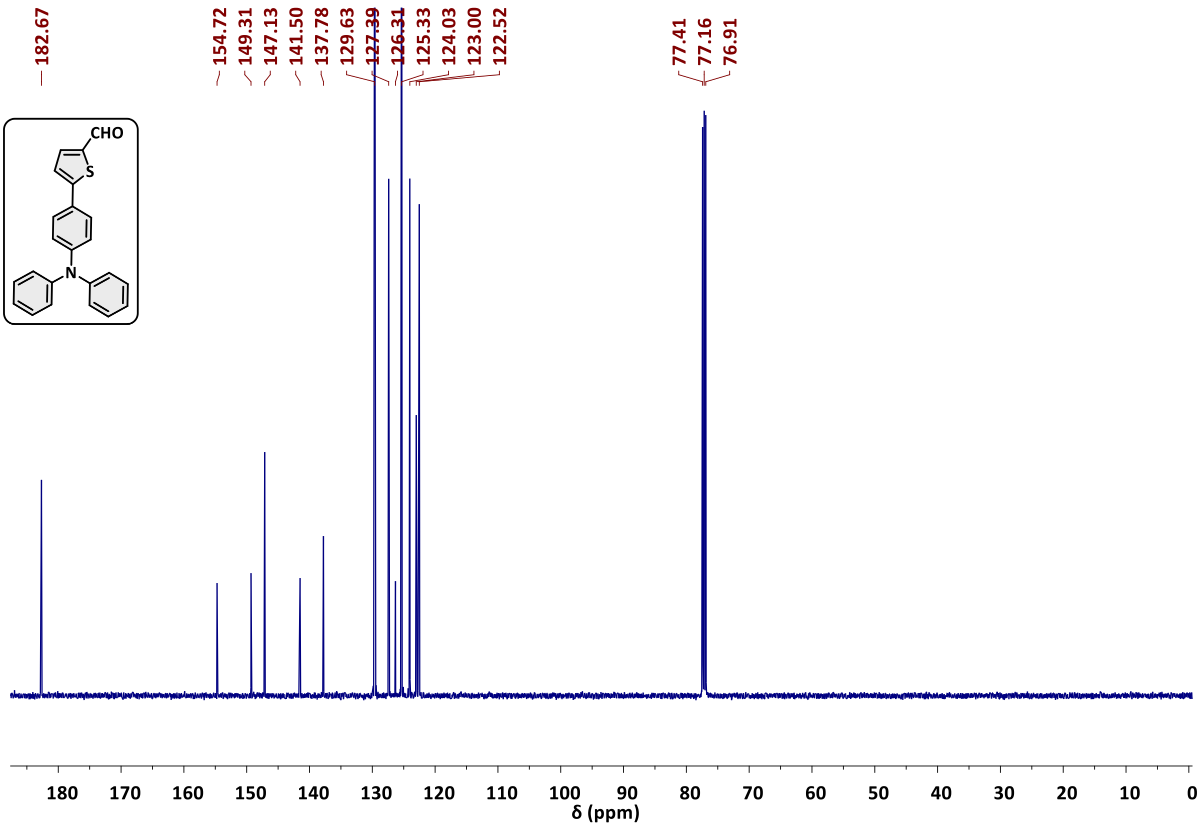

Figure S23: ^{1}H and ^{13}C NMR of III.

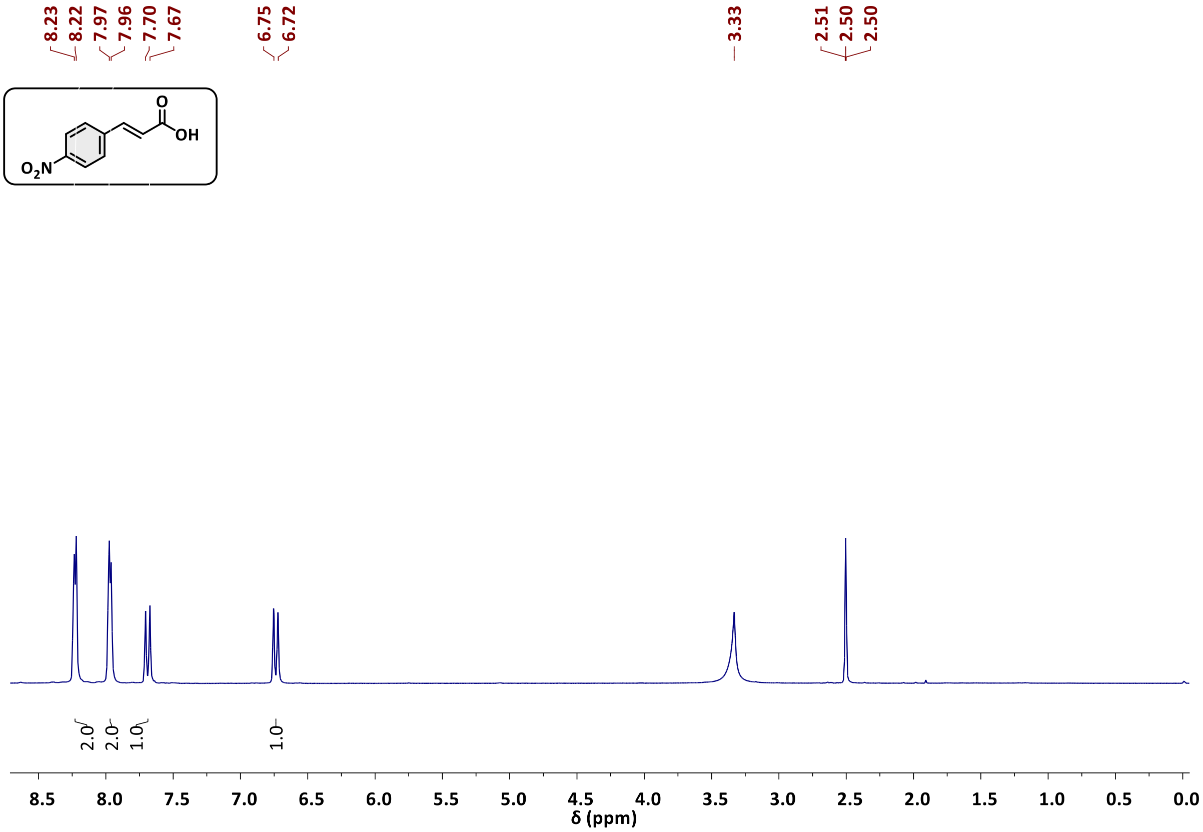

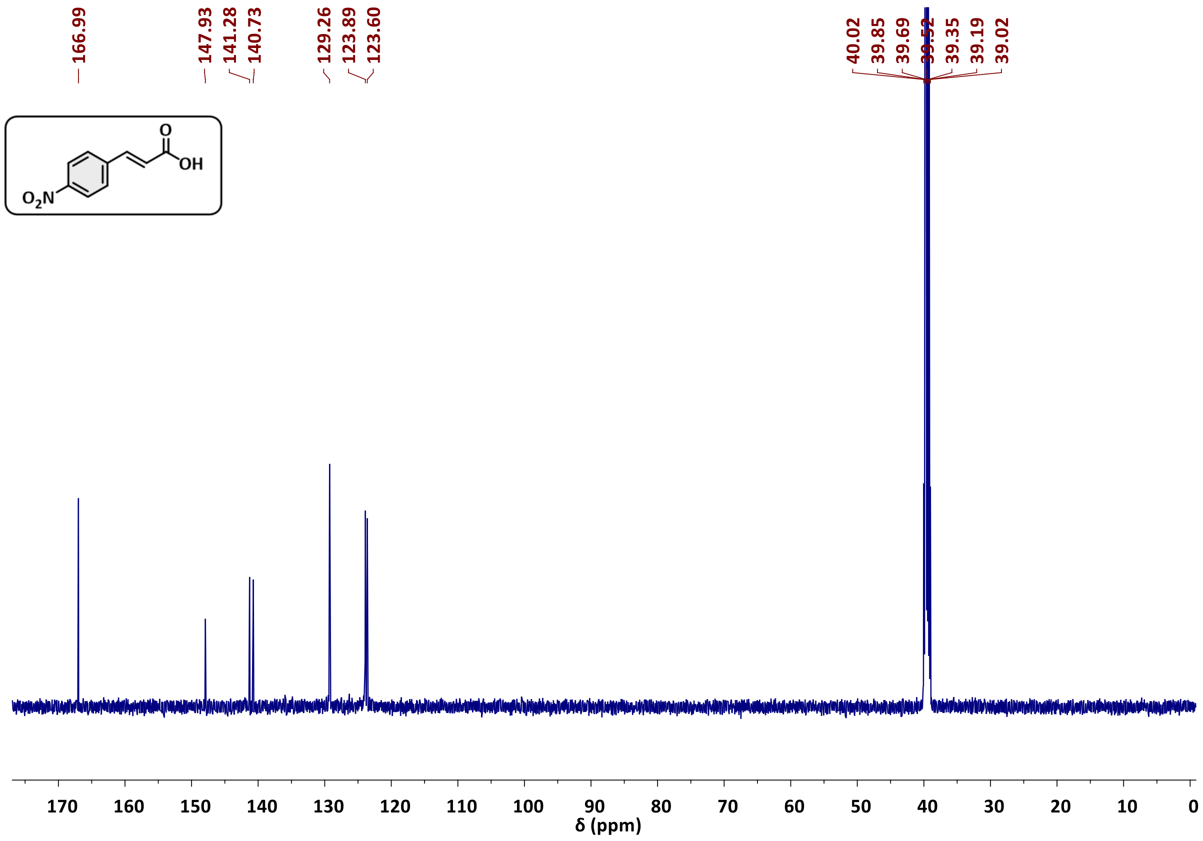

Figure S24: ^{1}H and ^{13}C NMR of 12C.

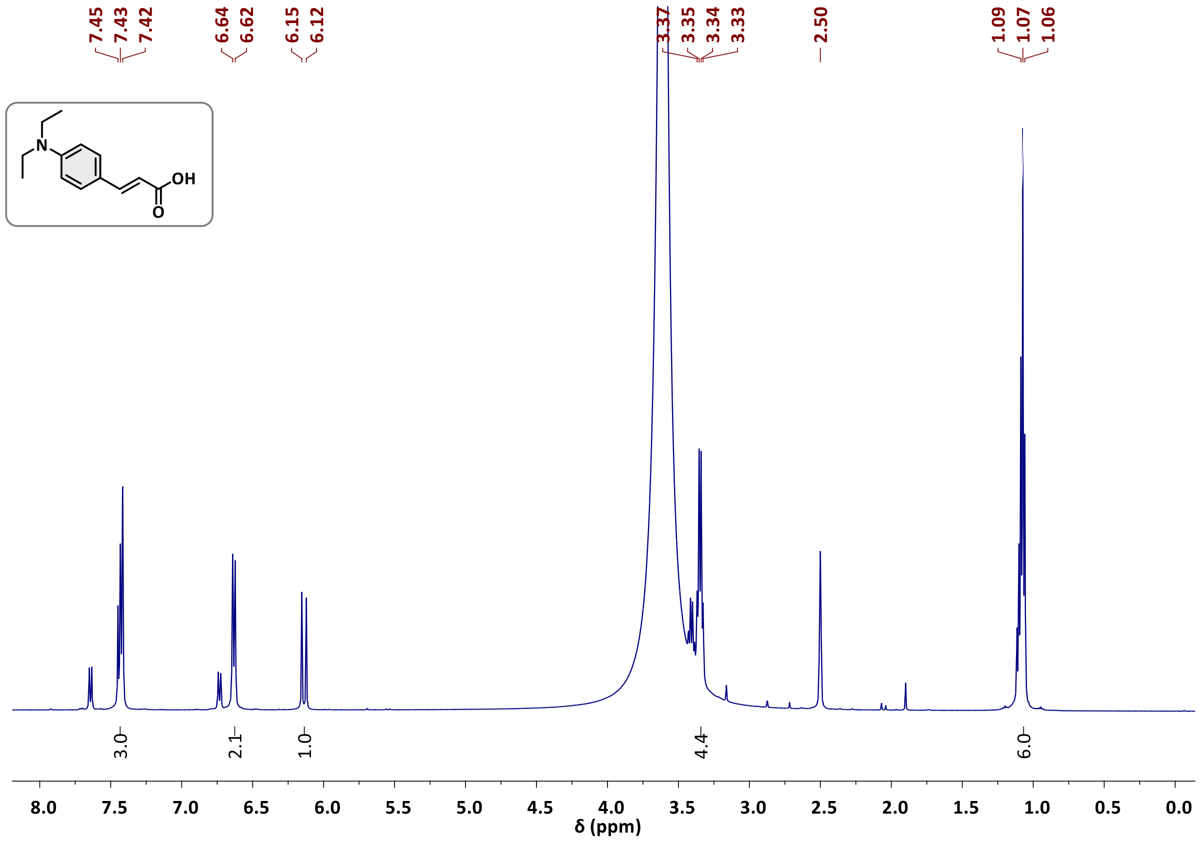

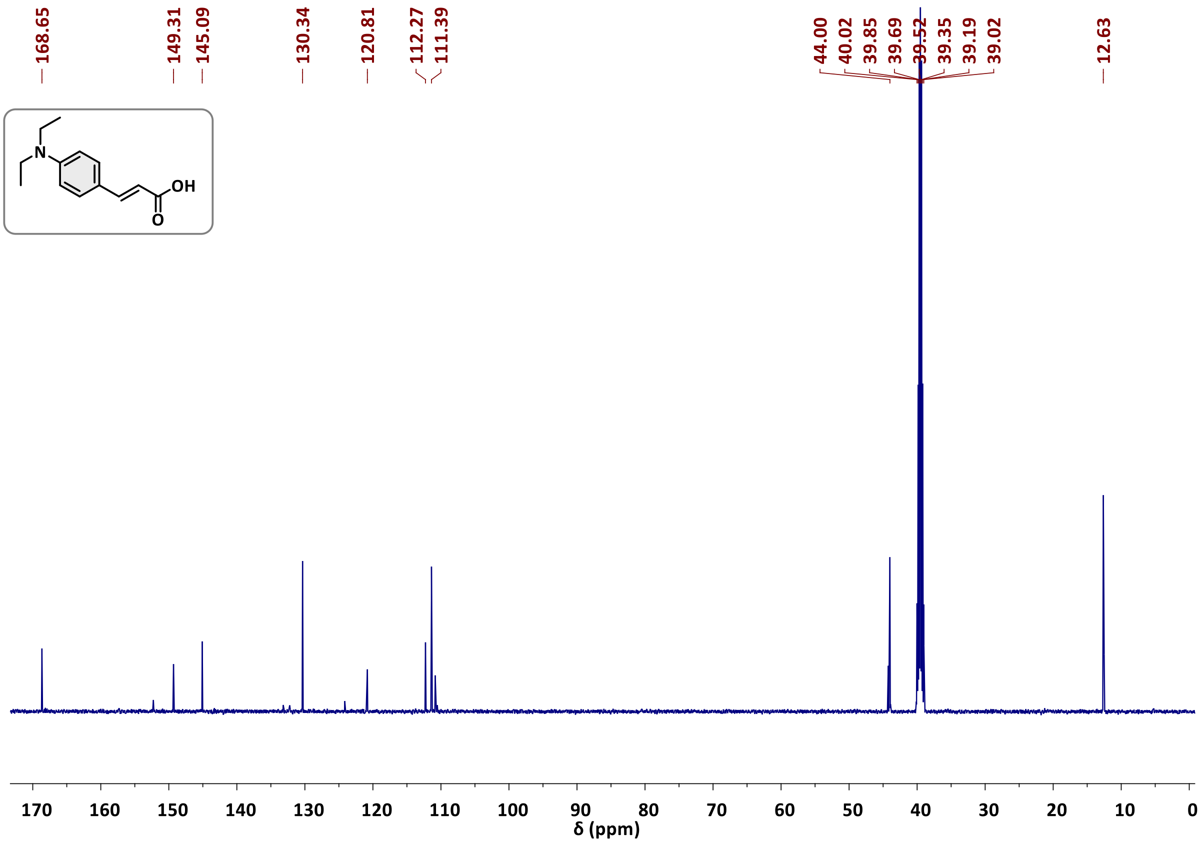

Figure S25: ^{1}H and ^{13}C NMR of 12D.

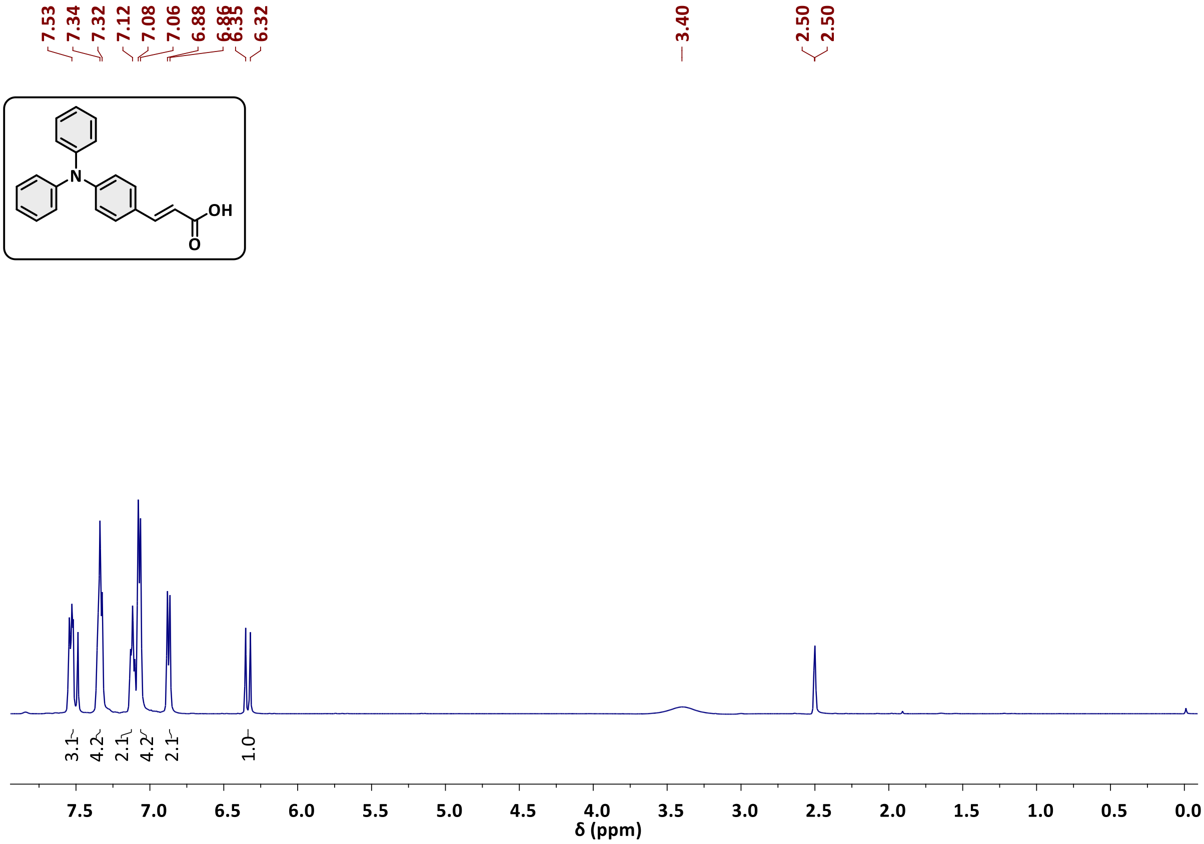

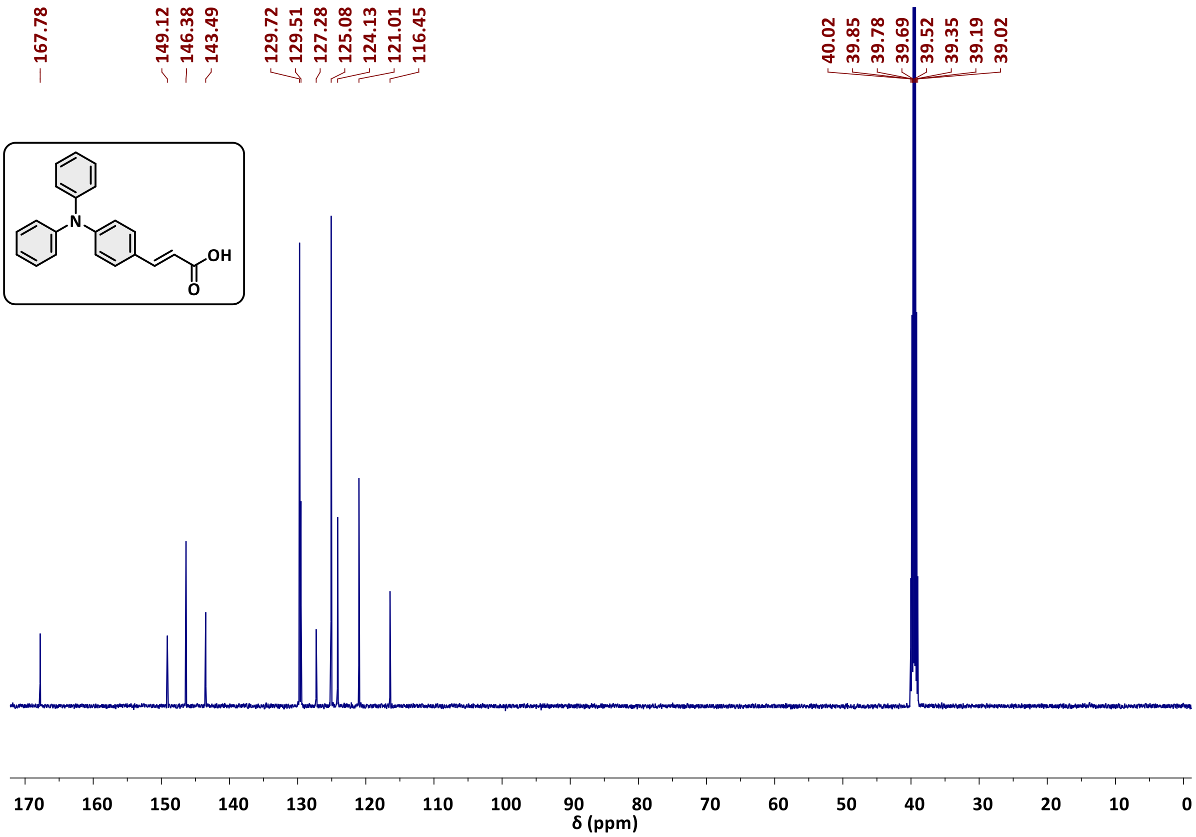

Figure S26: ^{1}H and ^{13}C NMR of 12E.

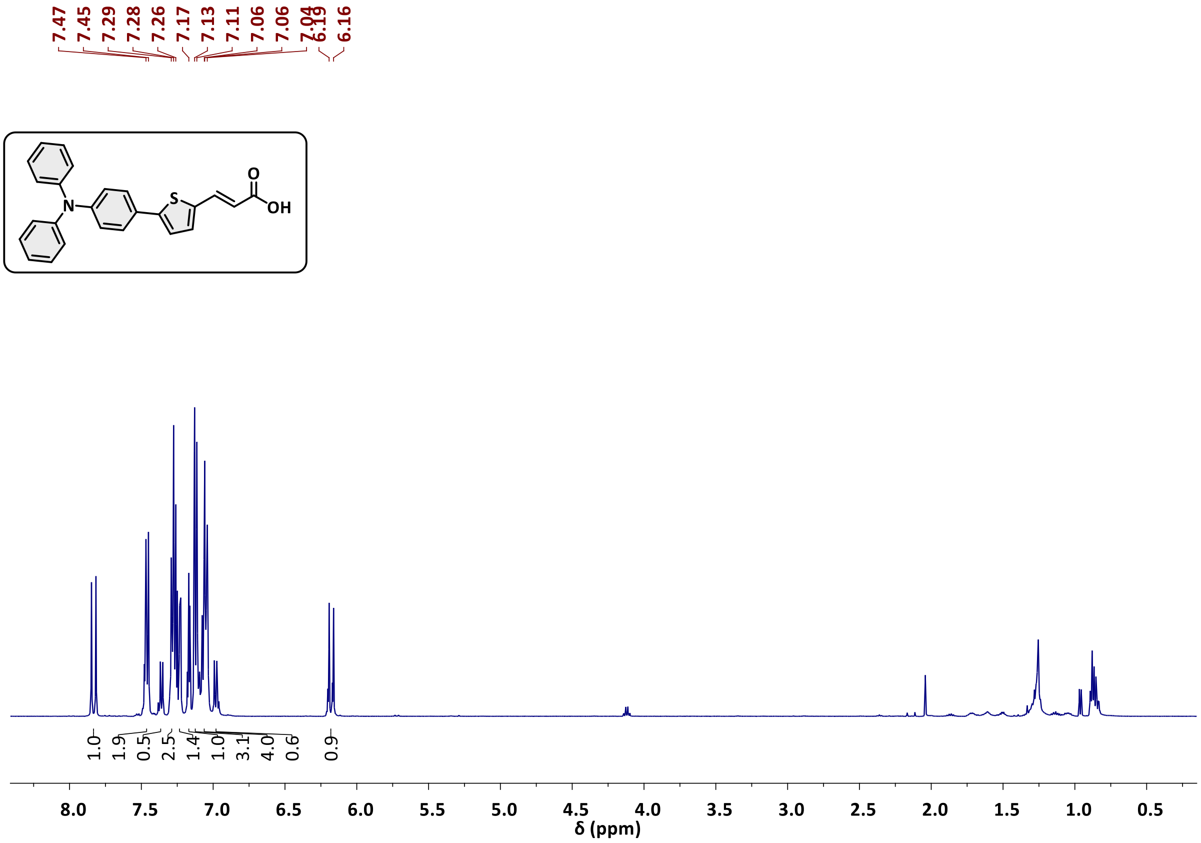

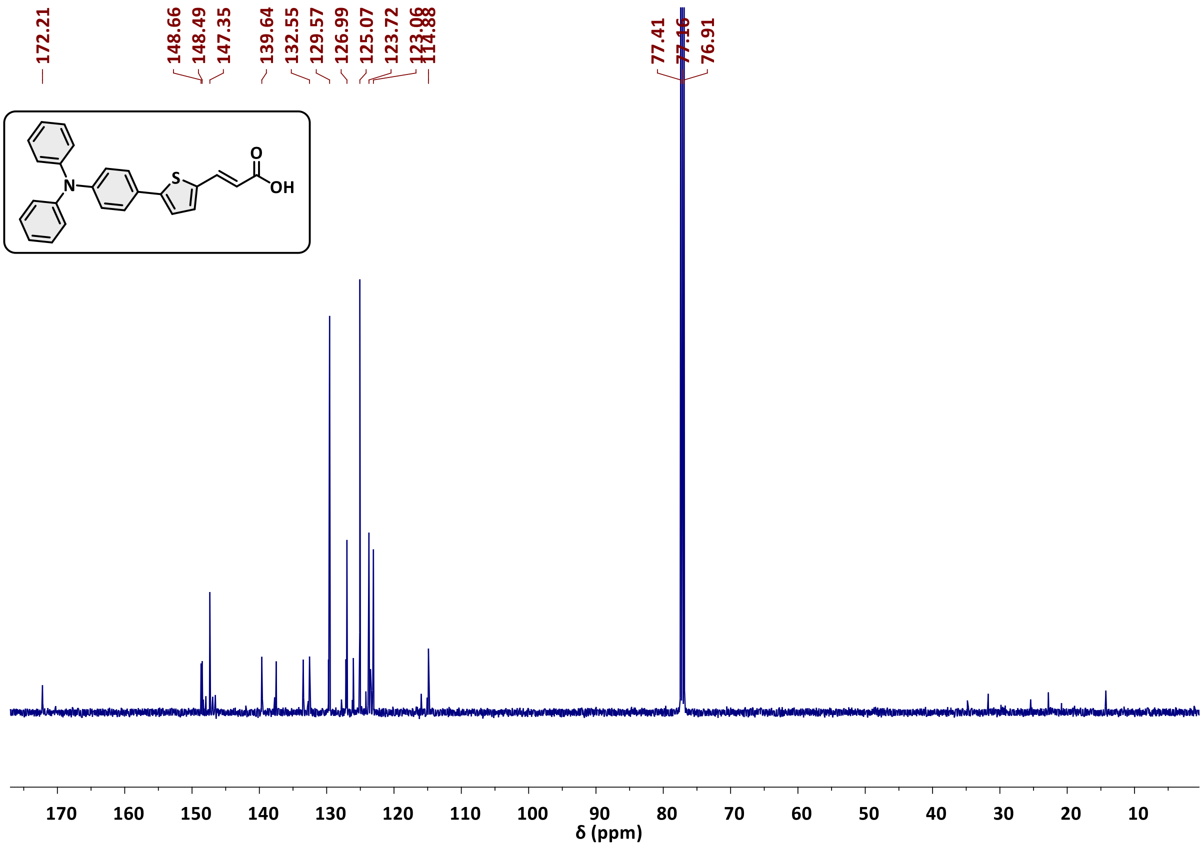

Figure S27: ^{1}H and ^{13}C NMR of 12F.

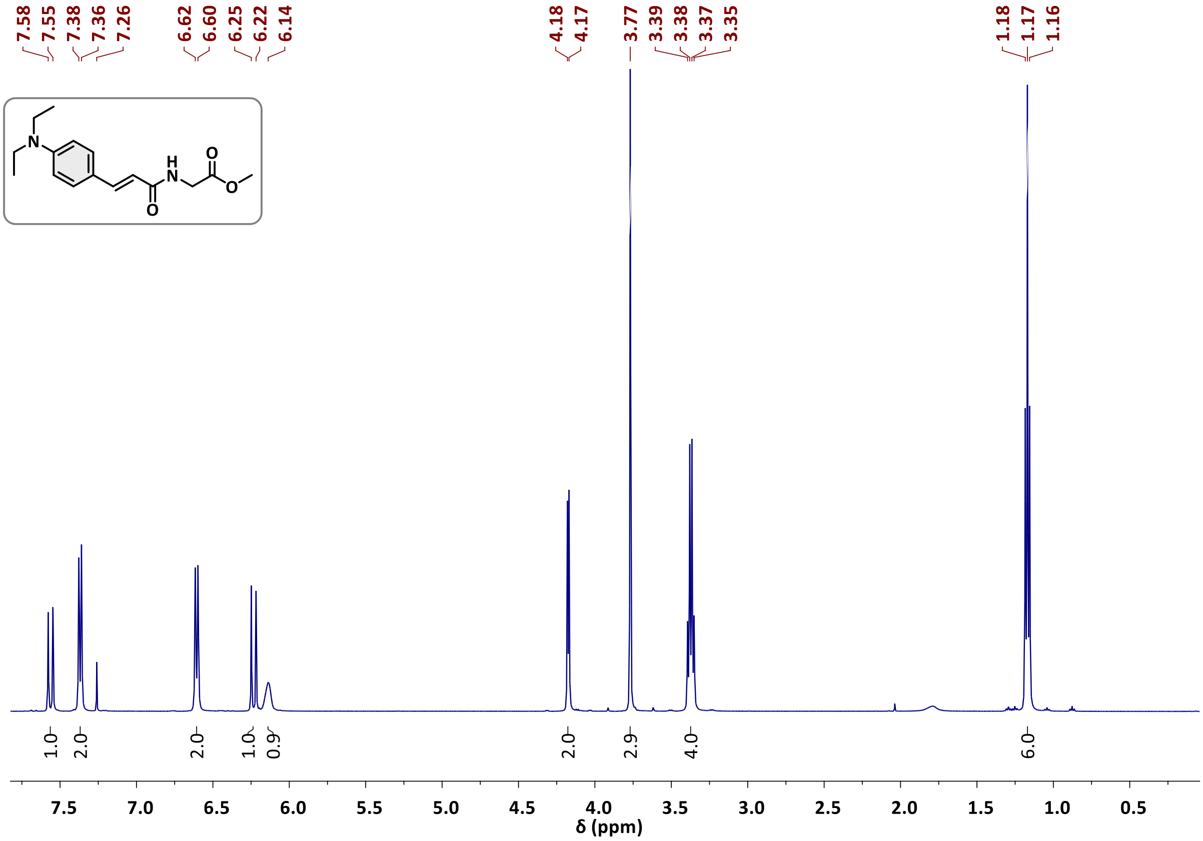

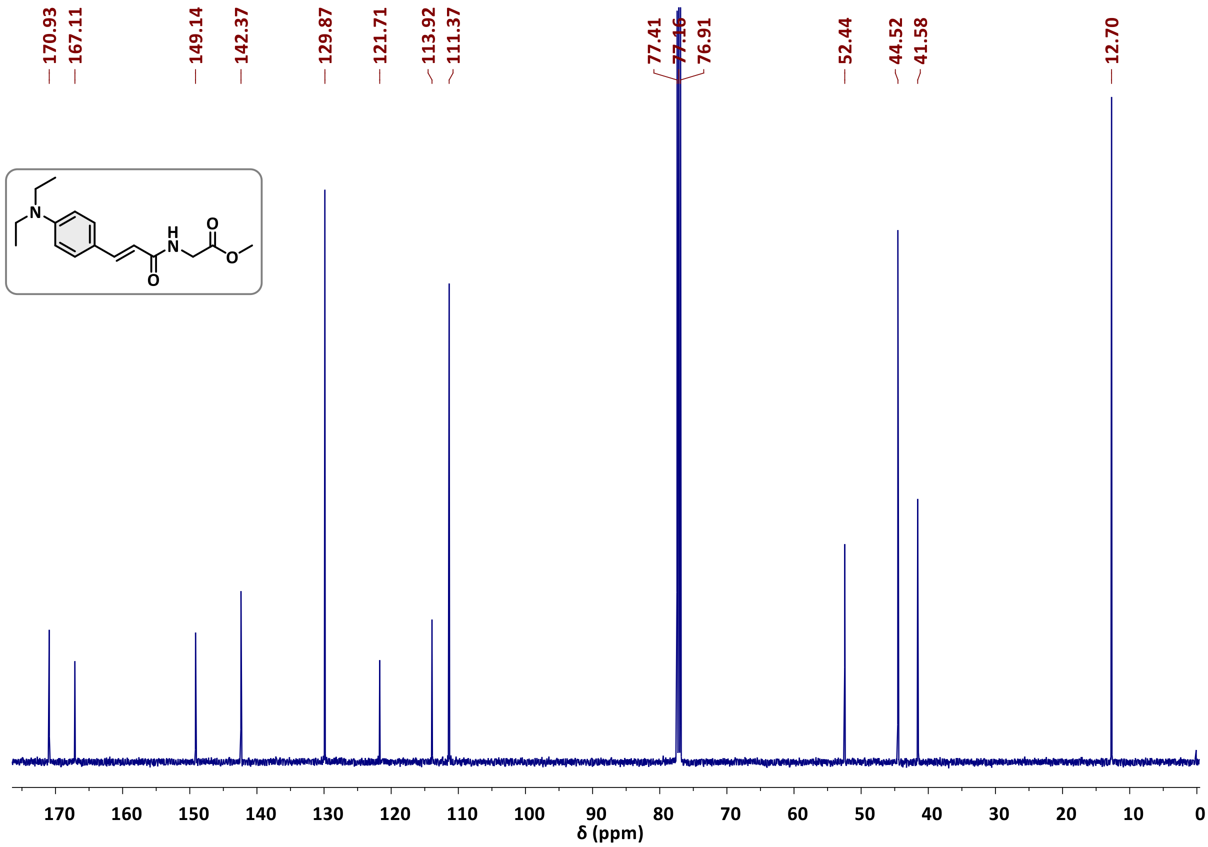

Figure S28: ^{1}H and ^{13}C NMR of 13D.

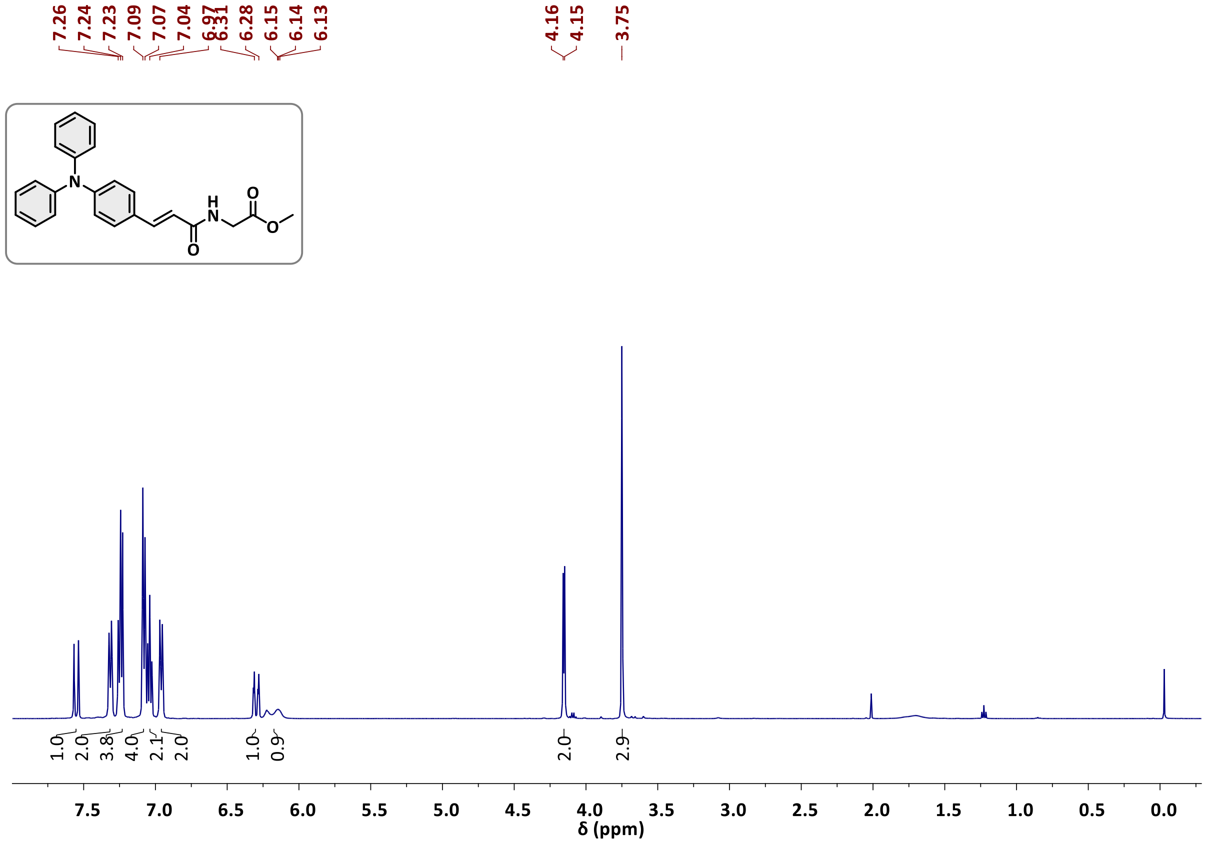

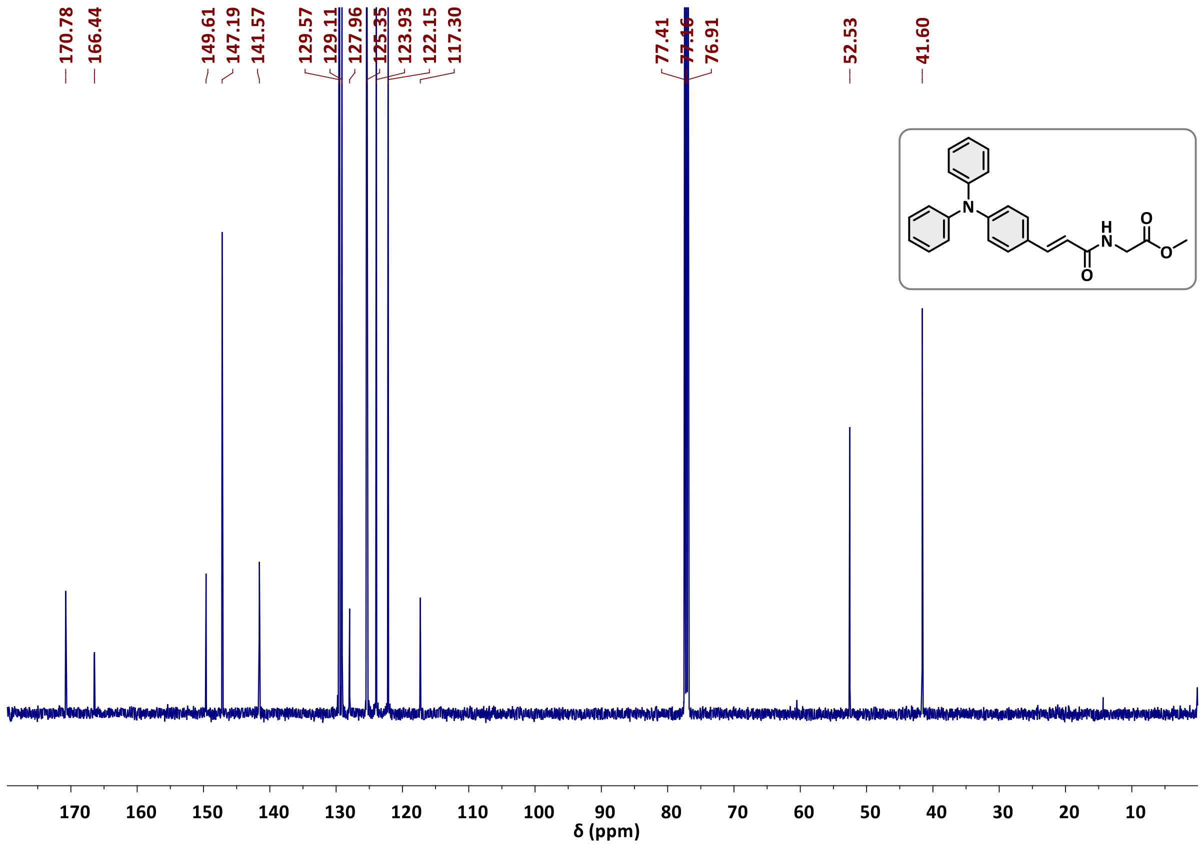

Figure S29: ^{1}H and ^{13}C NMR of 13E.

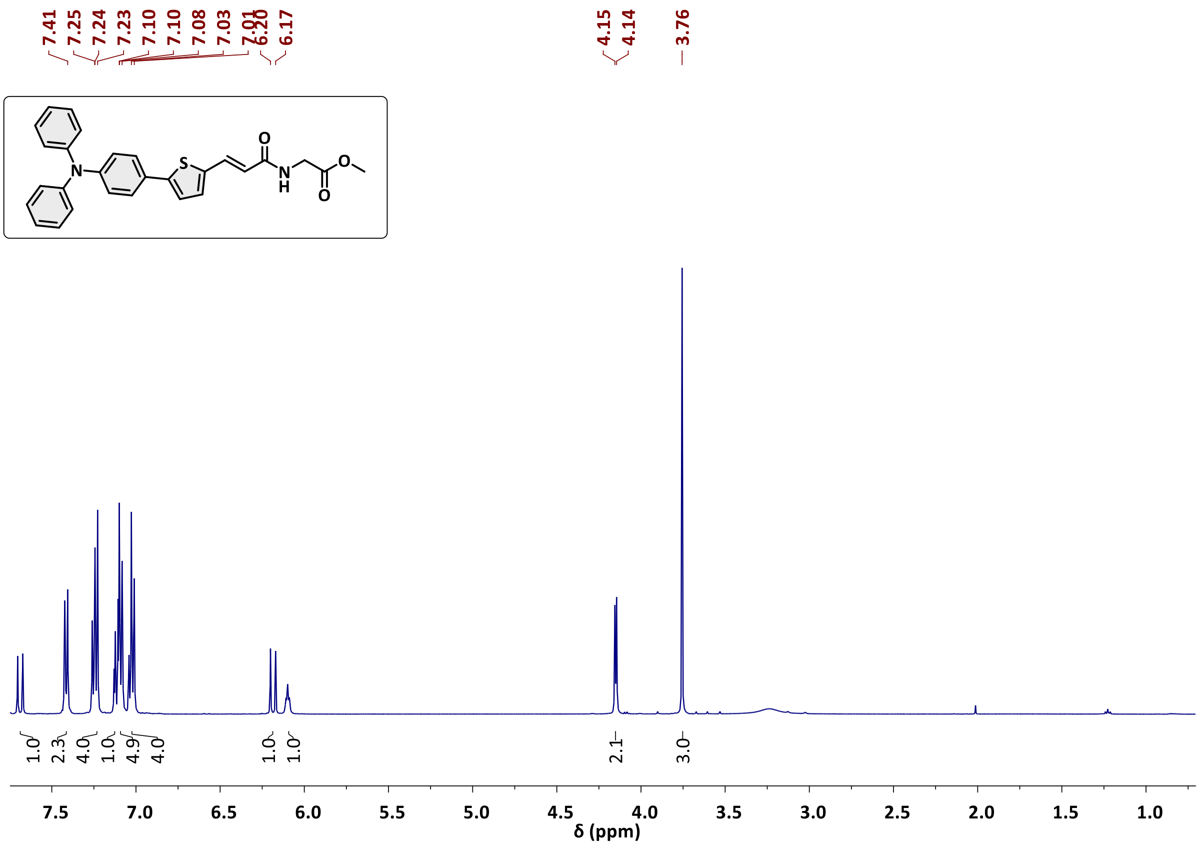

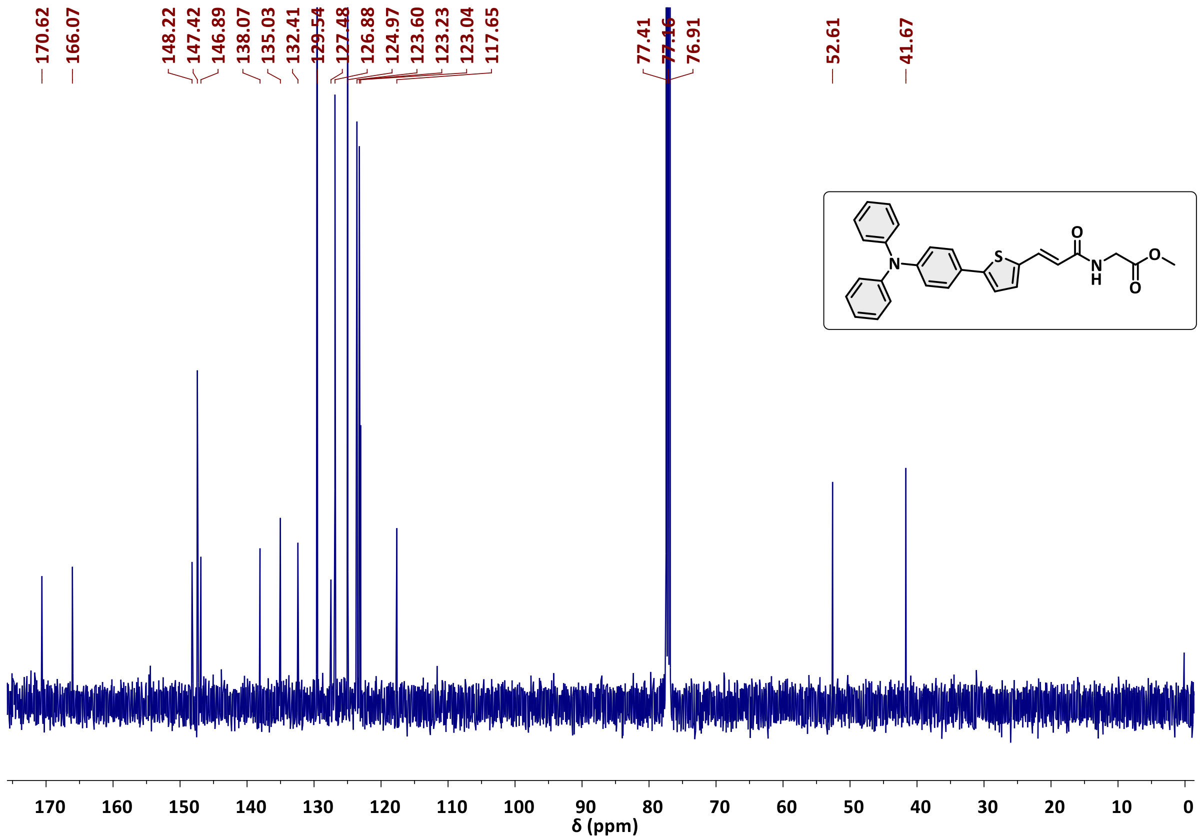

Figure S30: ^{1}H and ^{13}C NMR of 13F.

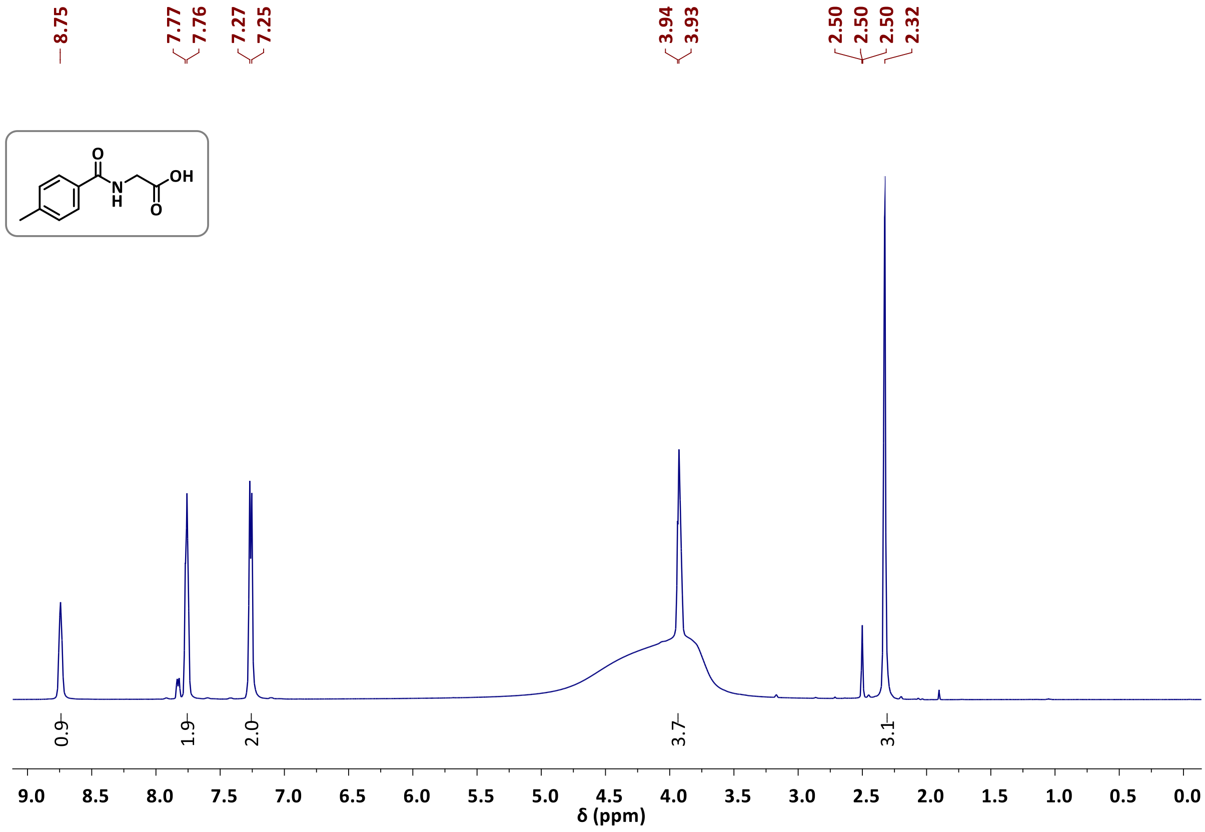

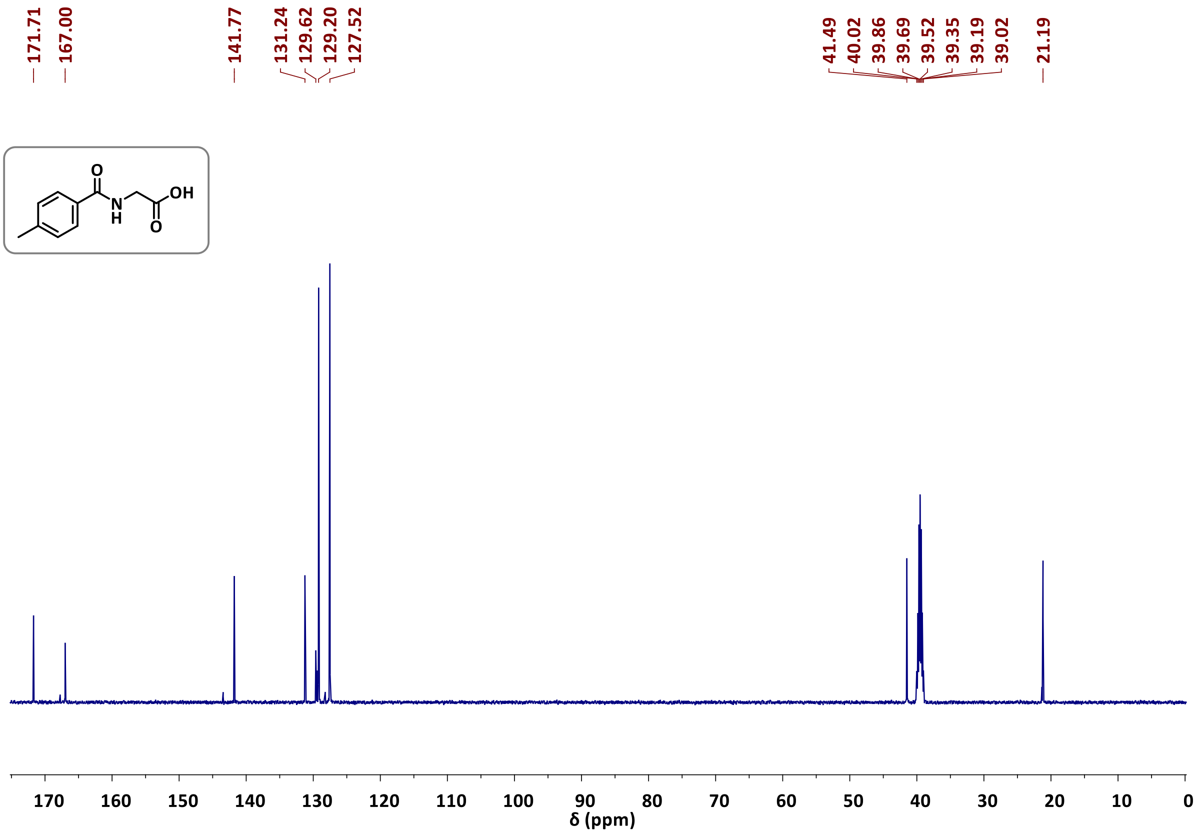

Figure S31: ^{1}H and ^{13}C NMR of 11A.

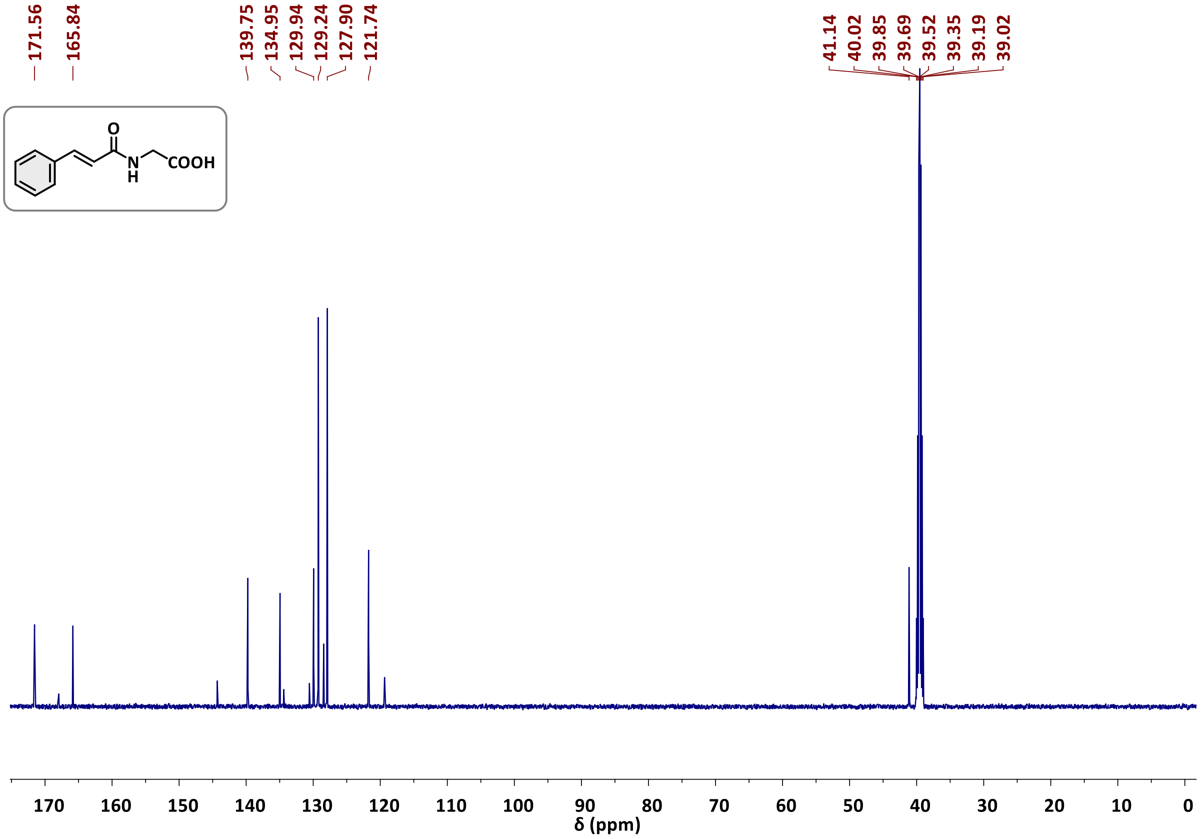

Figure S32: ^{1}H and ^{13}C NMR of 12B.

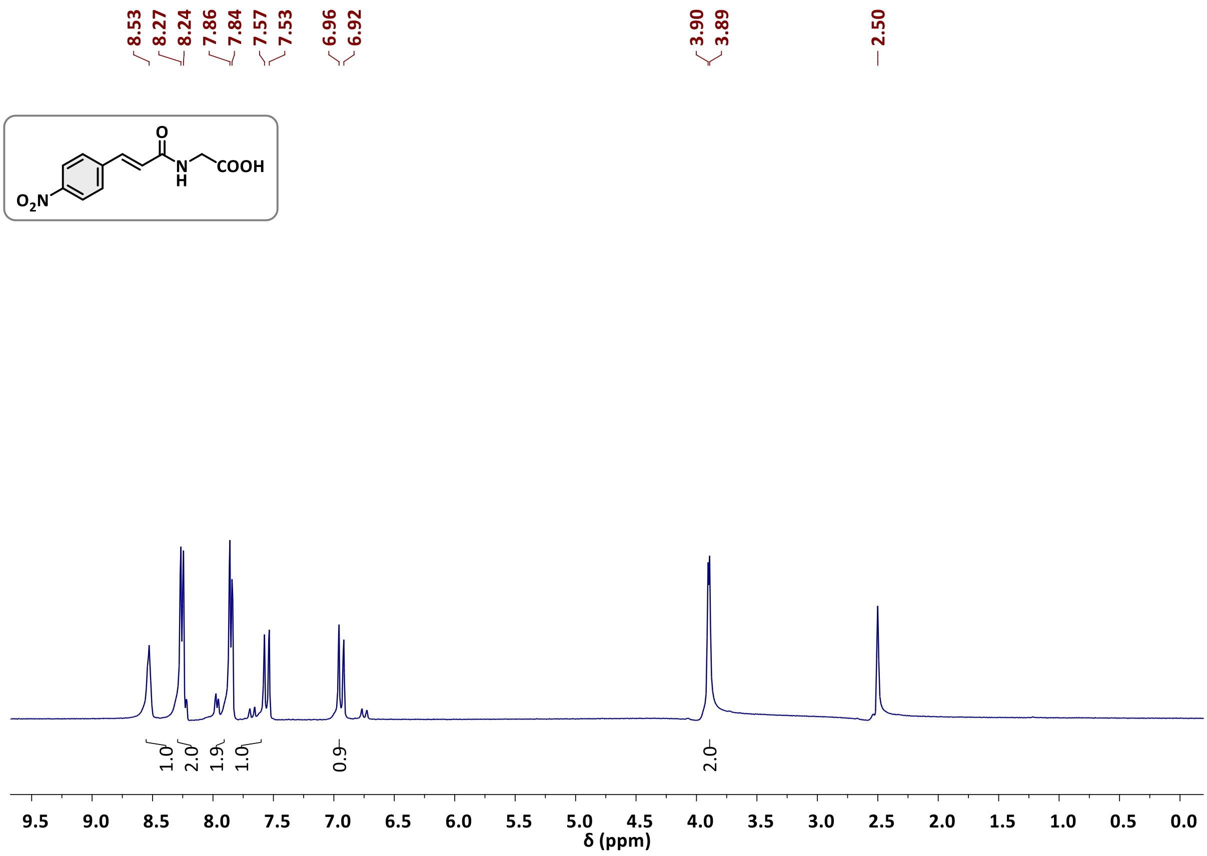

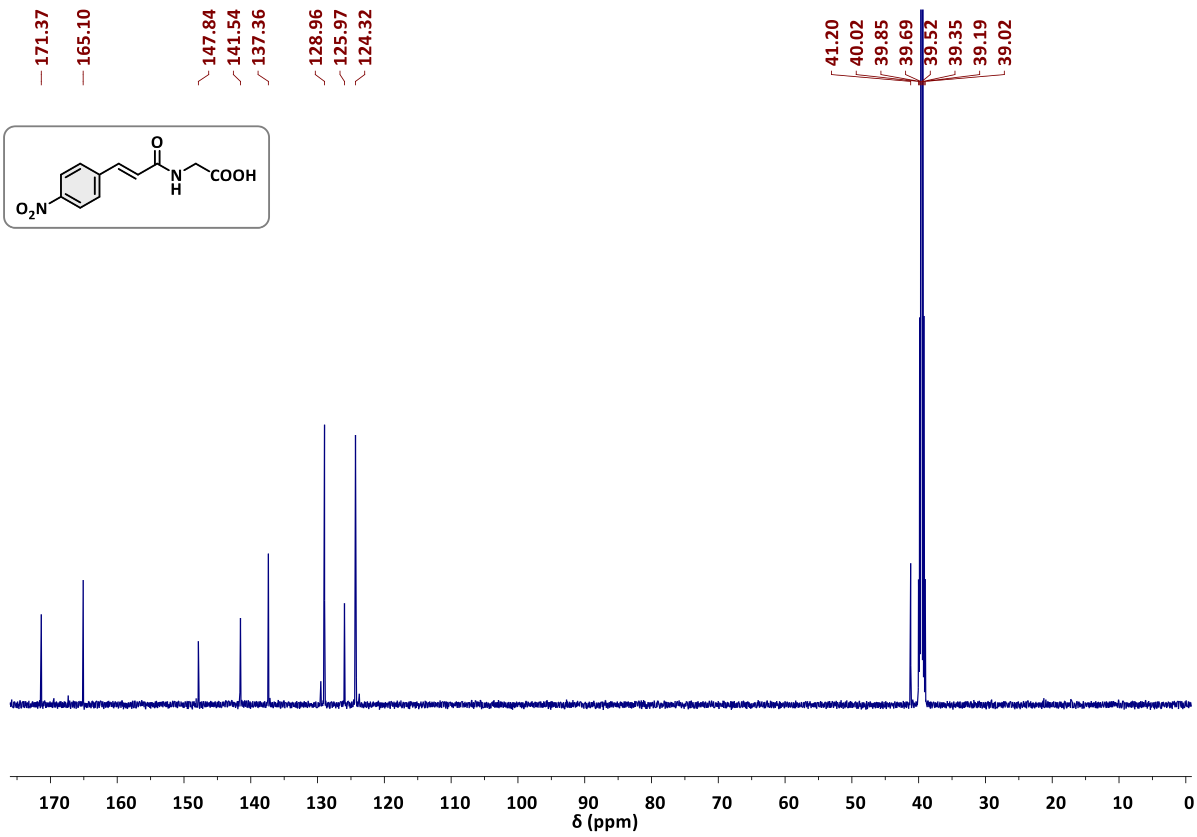

Figure S33: ^{1}H and ^{13}C NMR of 14C.

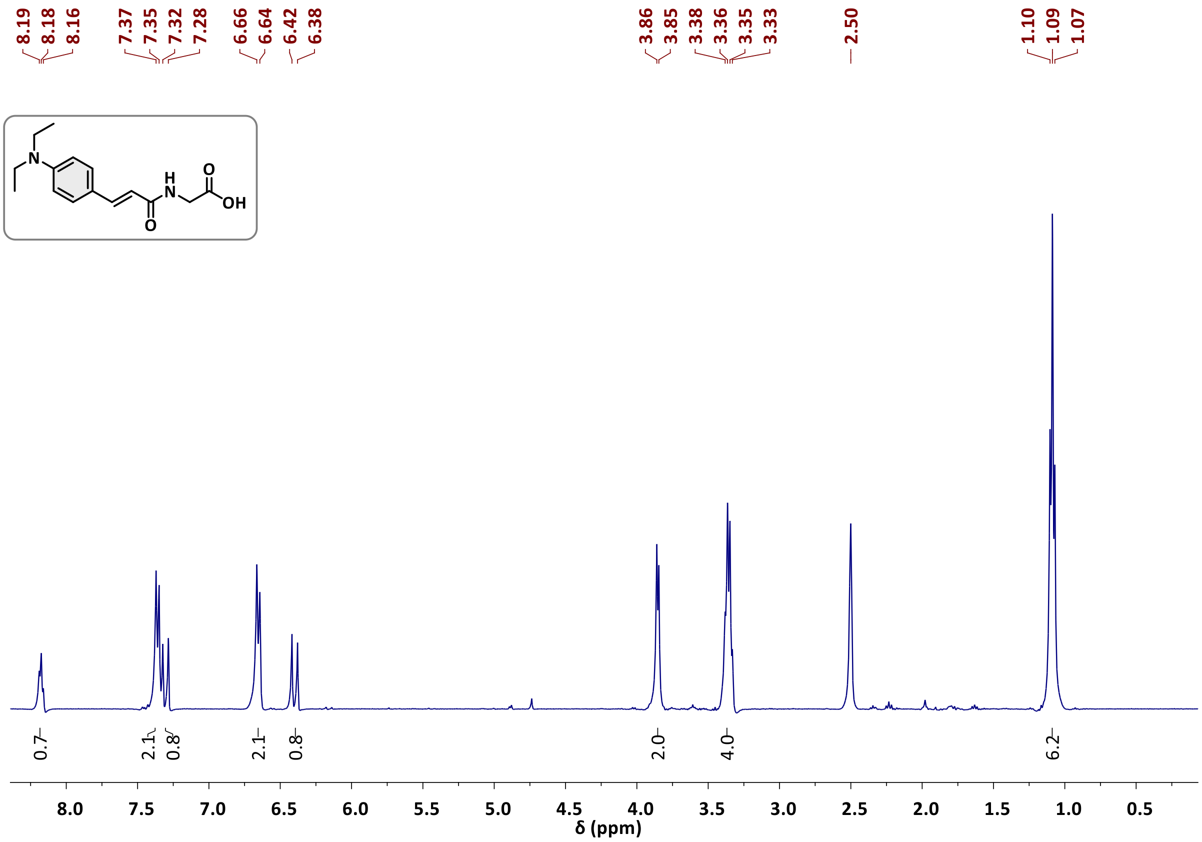

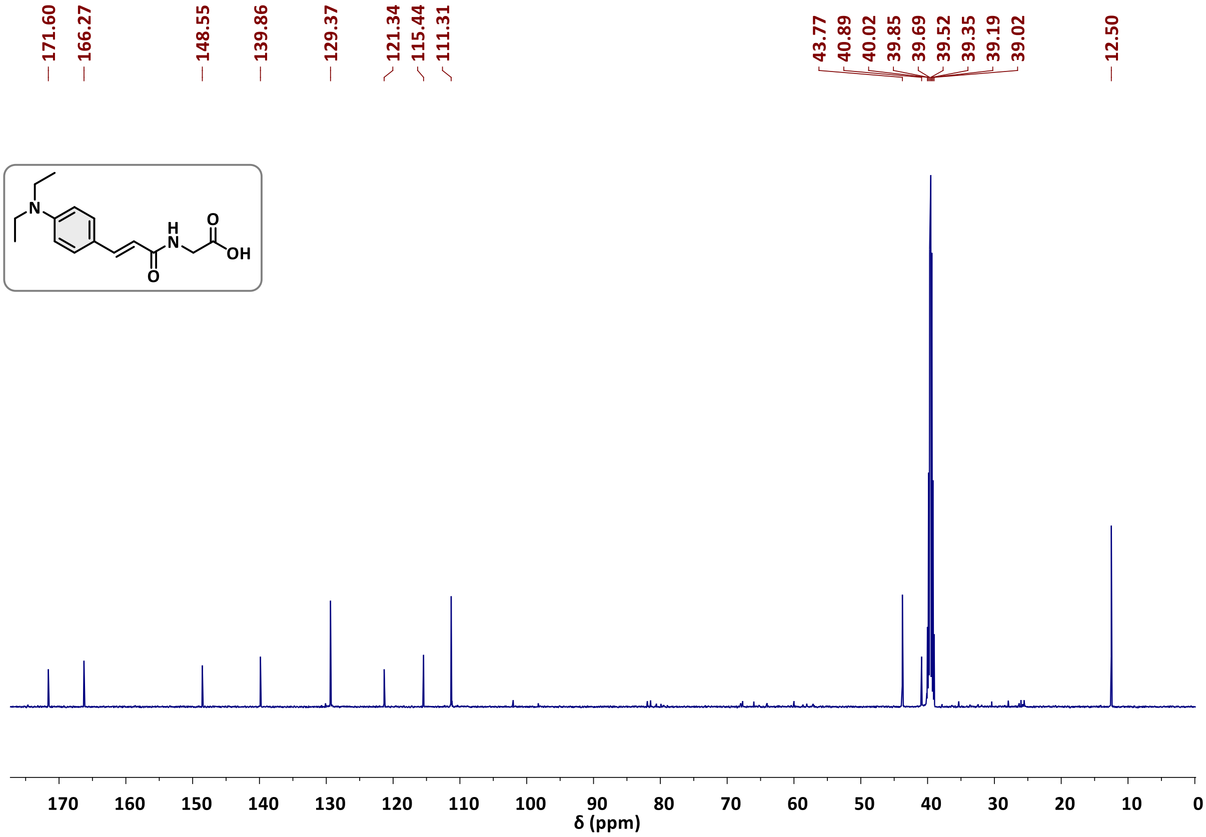

Figure S34: ^{1}H and ^{13}C NMR of 14D.

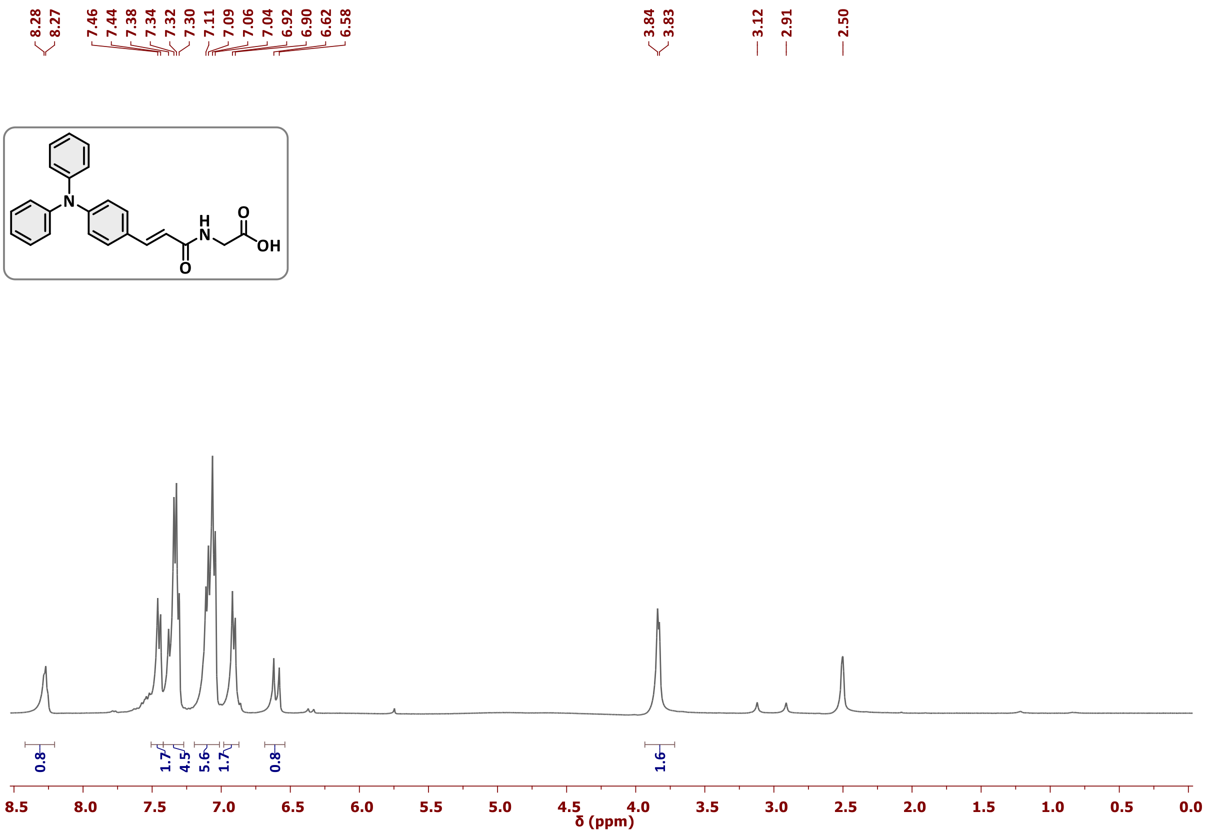

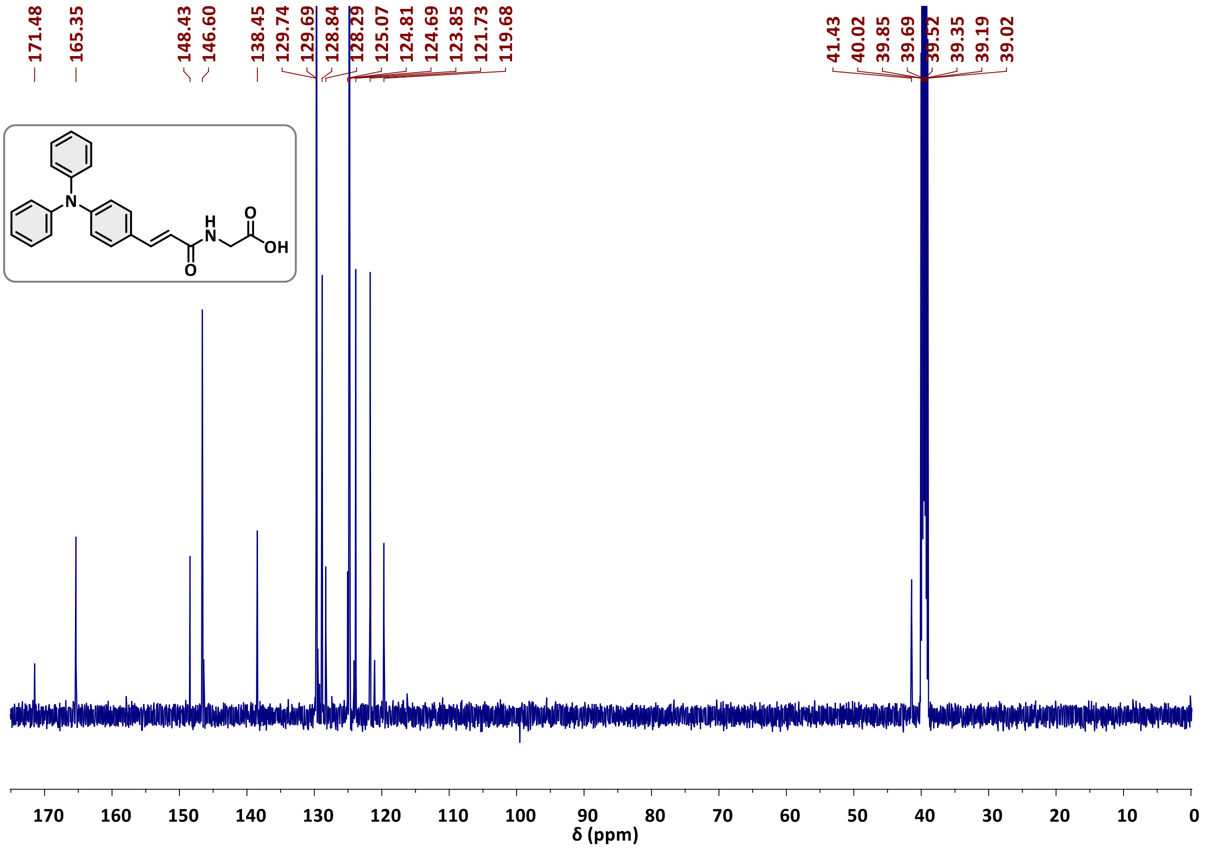

Figure S35: ^{1}H and ^{13}C NMR of 14E.

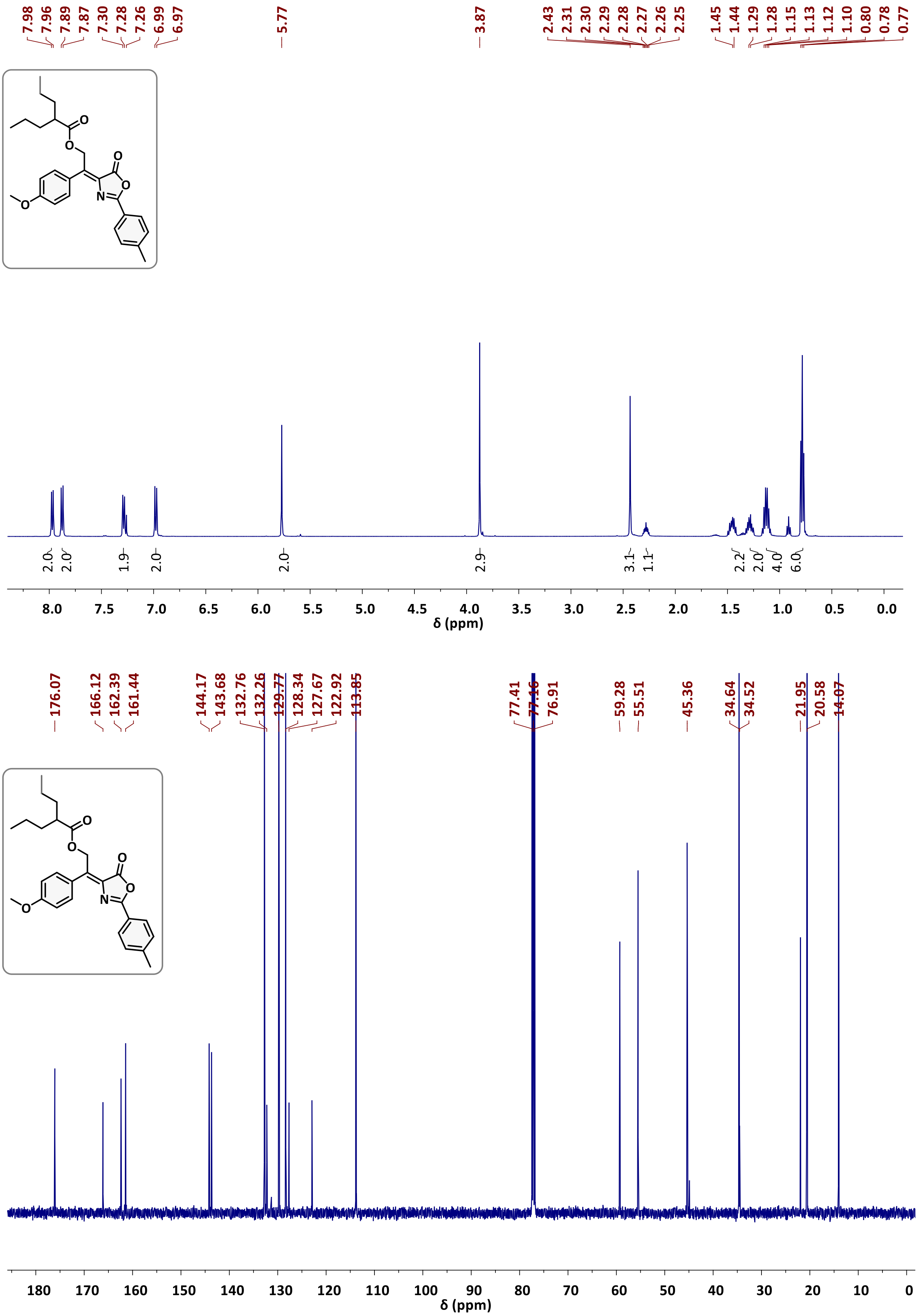

Figure S36: ^{1}H and ^{13}C NMR of 15A.

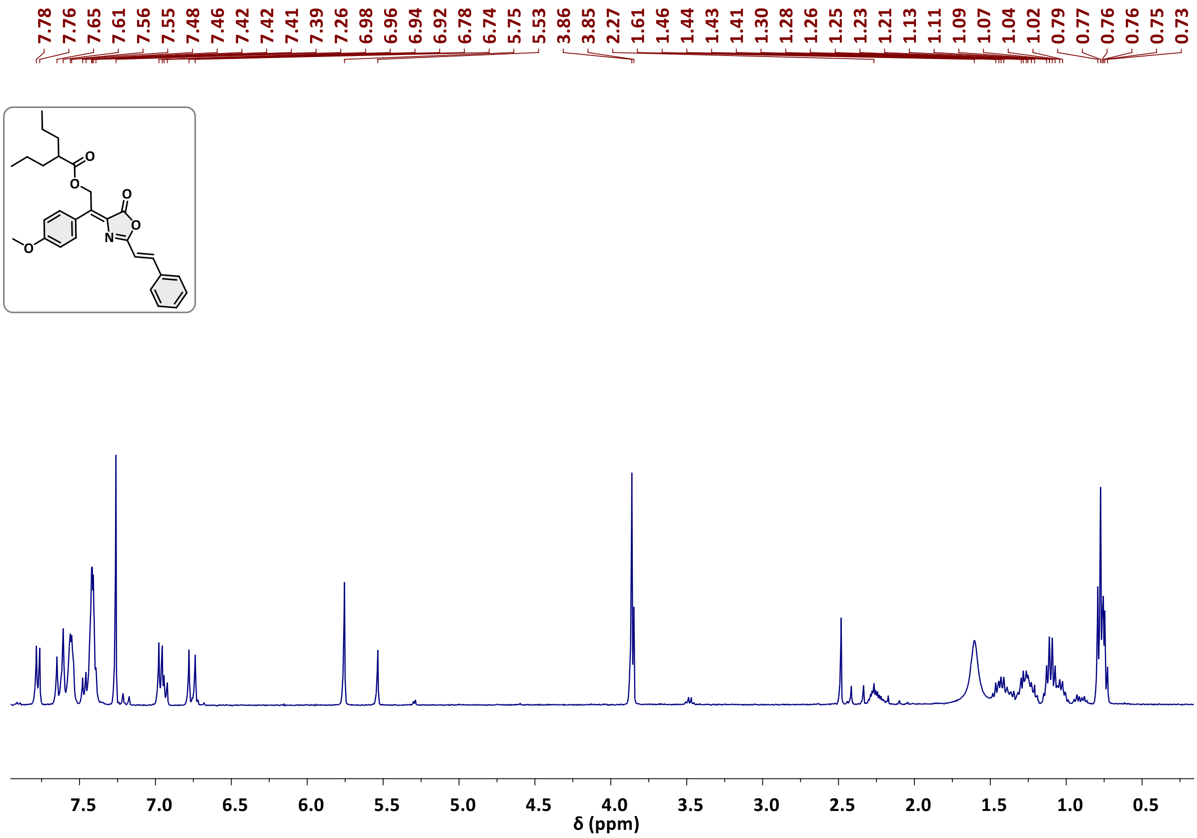

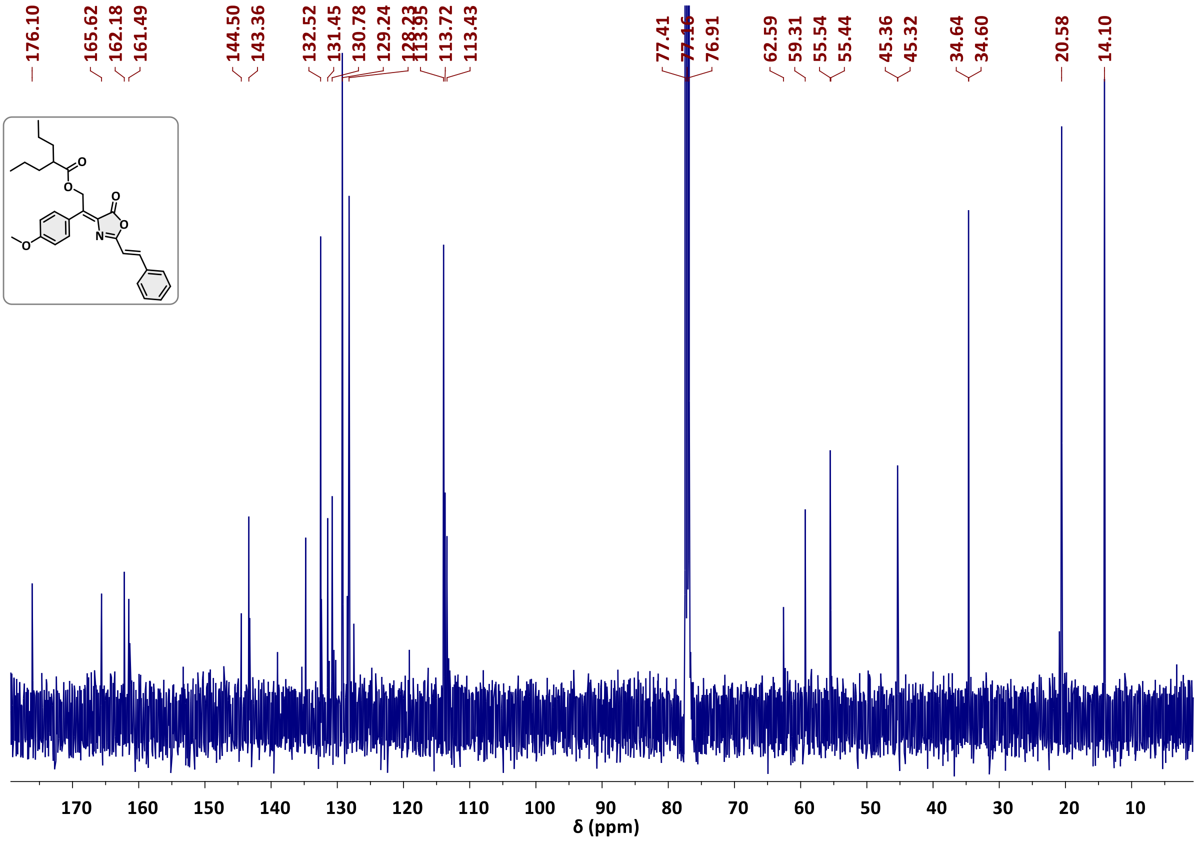

Figure S37: ^{1}H and ^{13}C NMR of 15B.

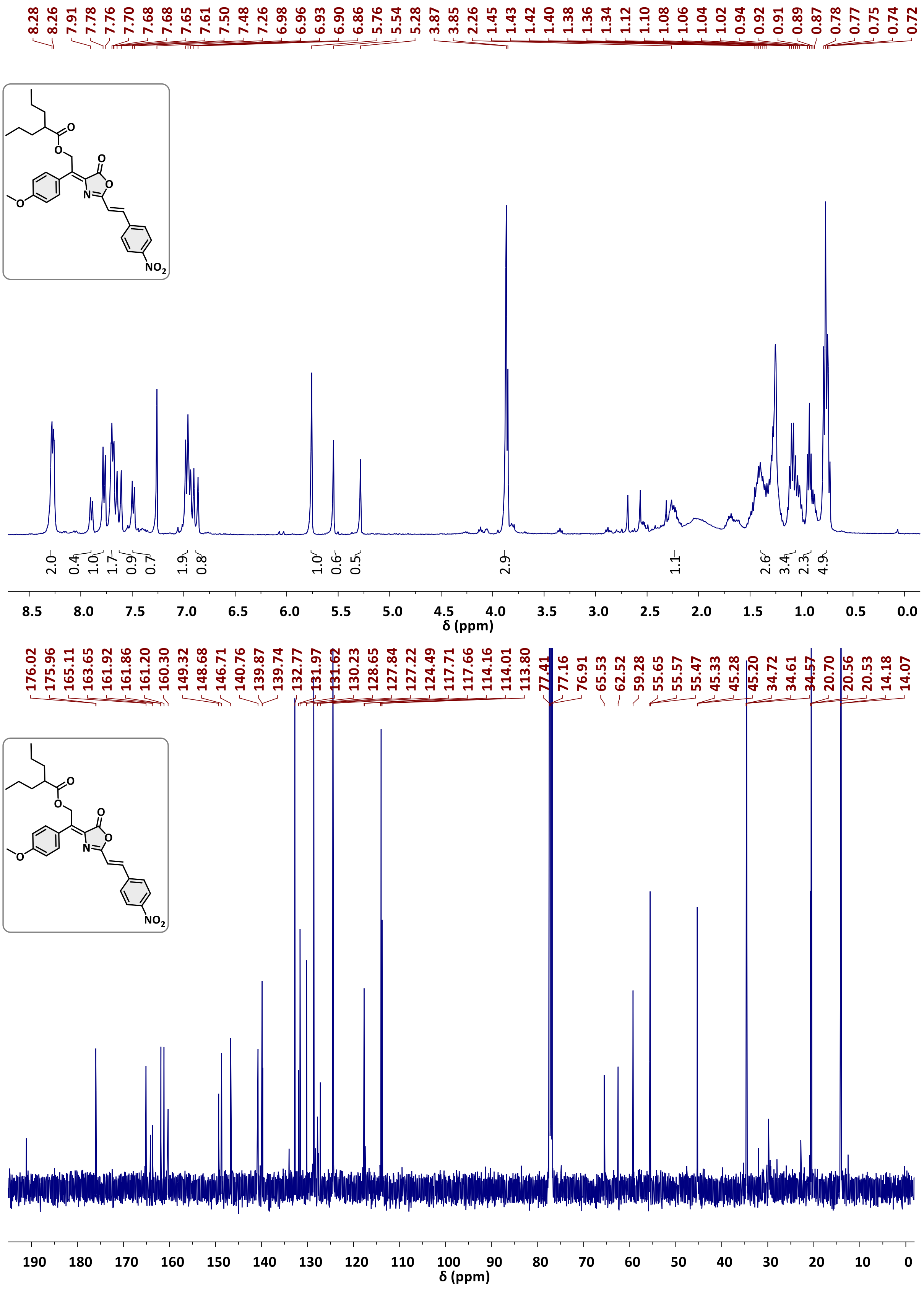

Figure S38: ^{1}H and ^{13}C NMR of 15C.

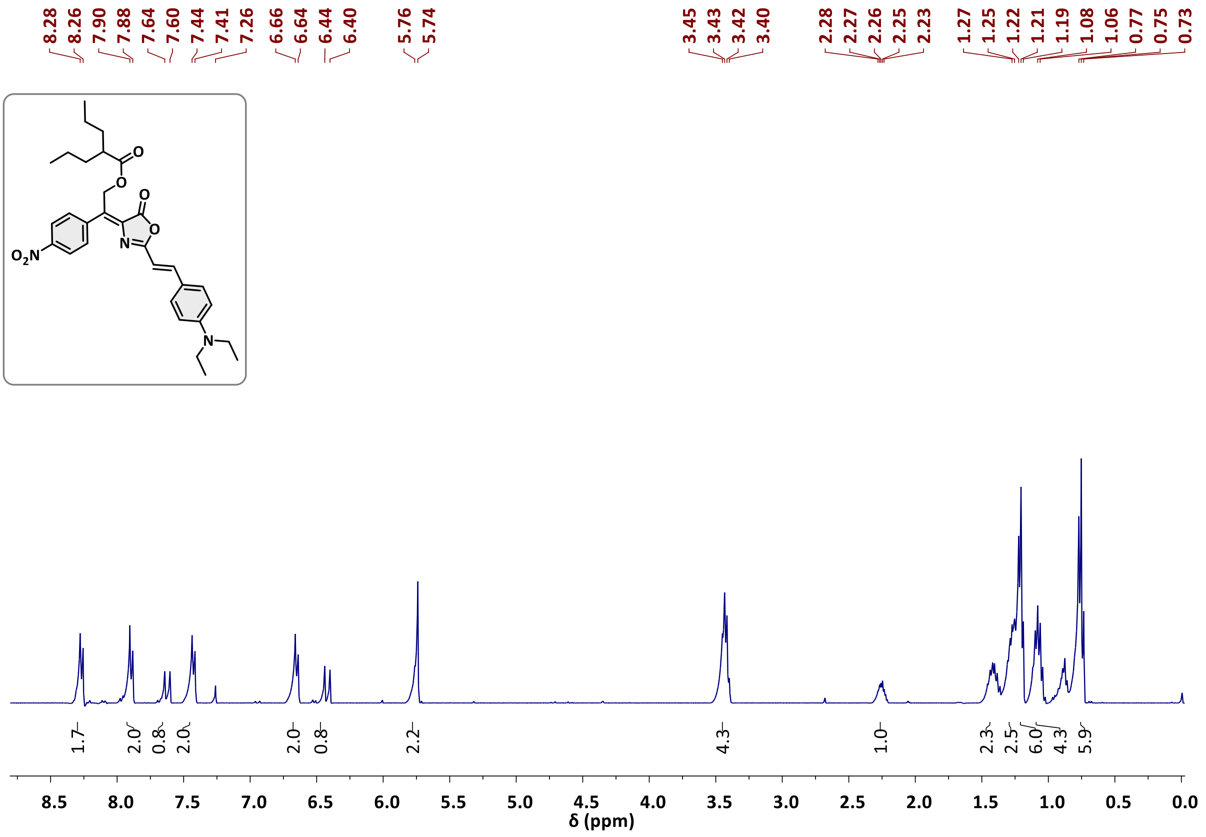

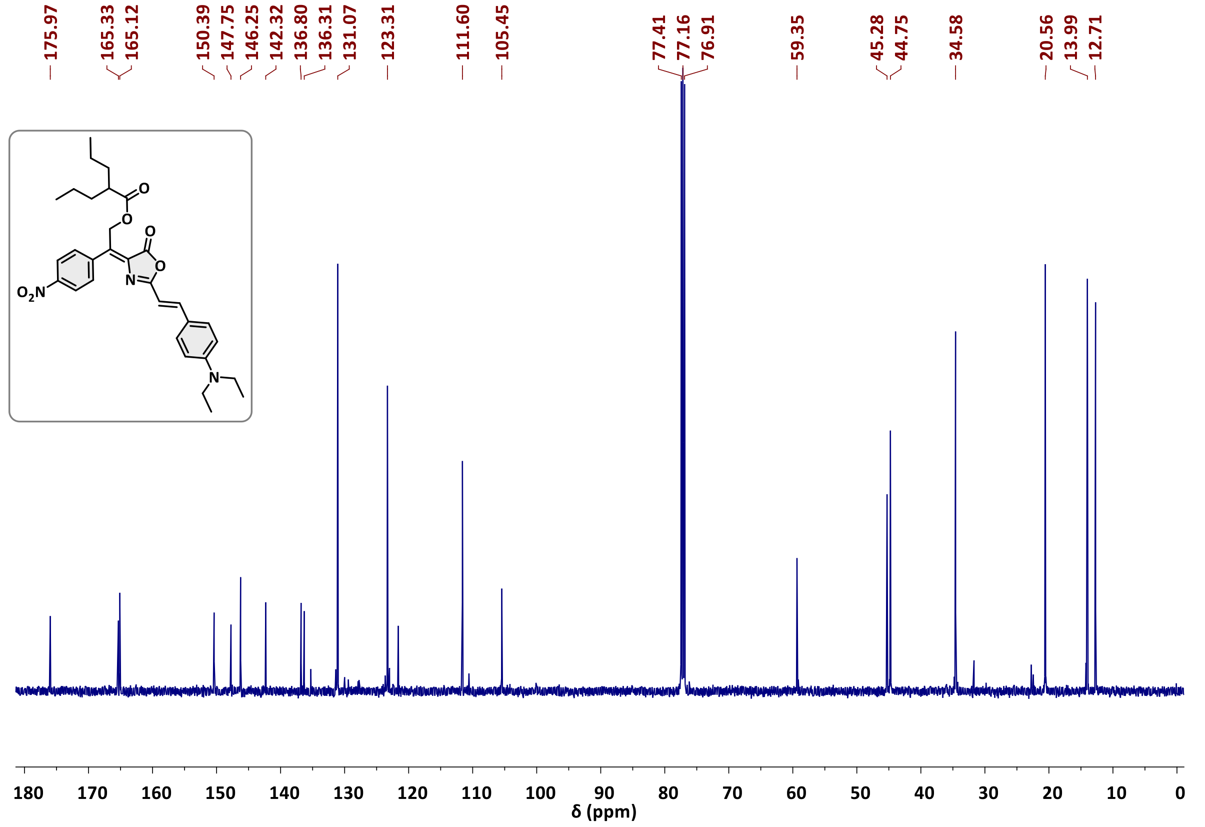

Figure S39: ^{1}H and ^{13}C NMR of 15D.

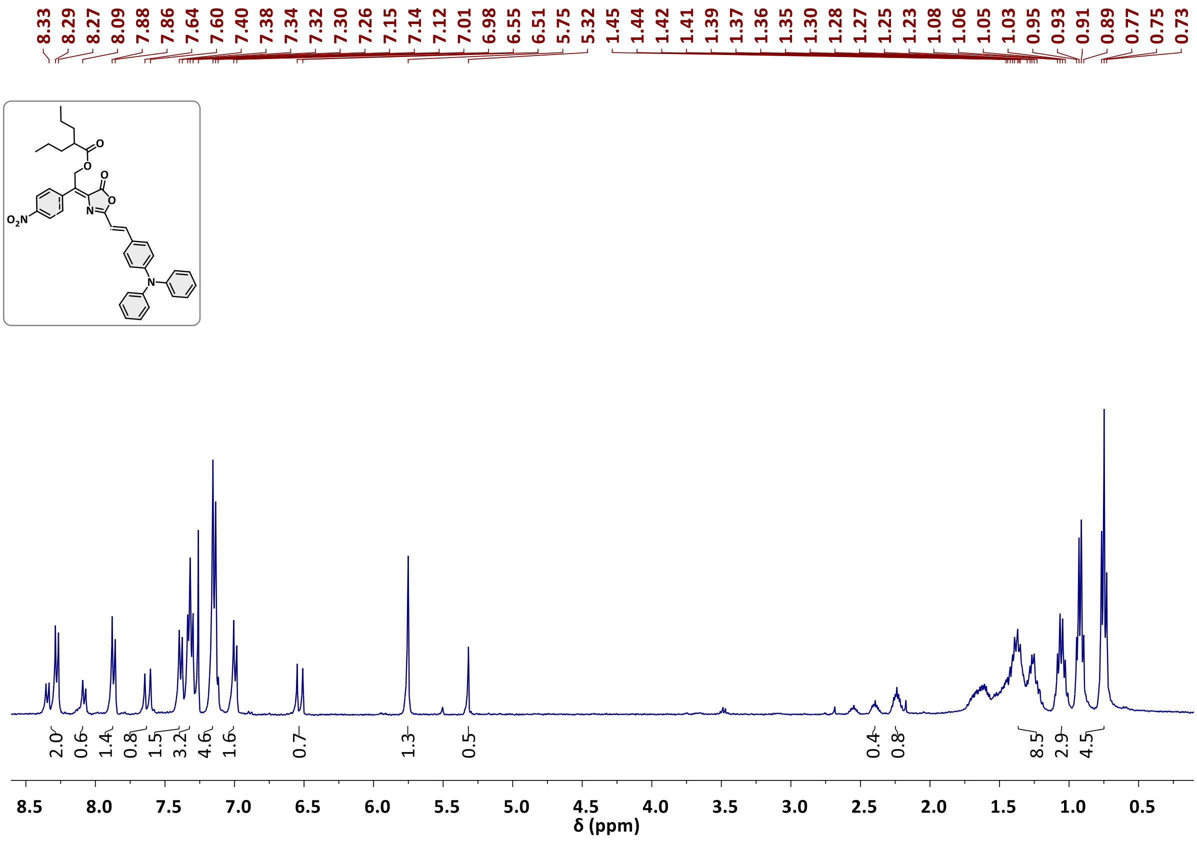

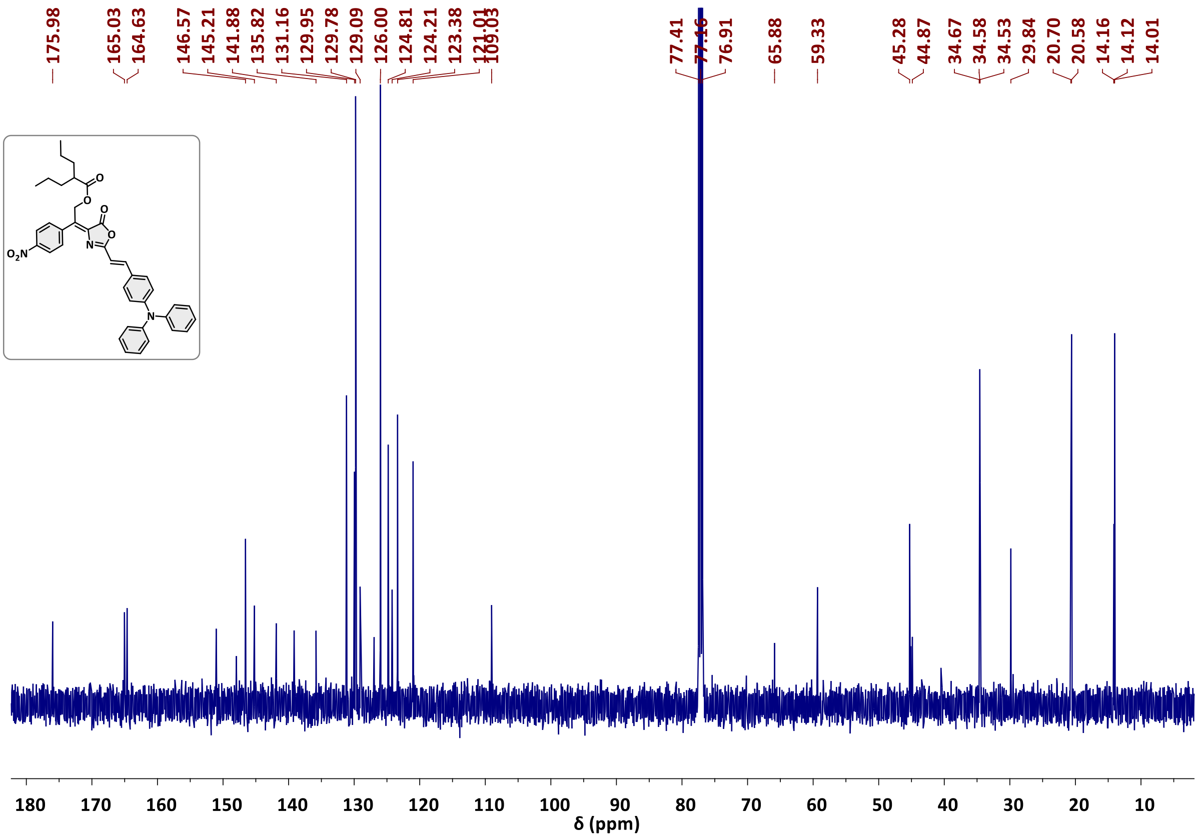

Figure S40: ^{1}H and ^{13}C NMR of 15E.

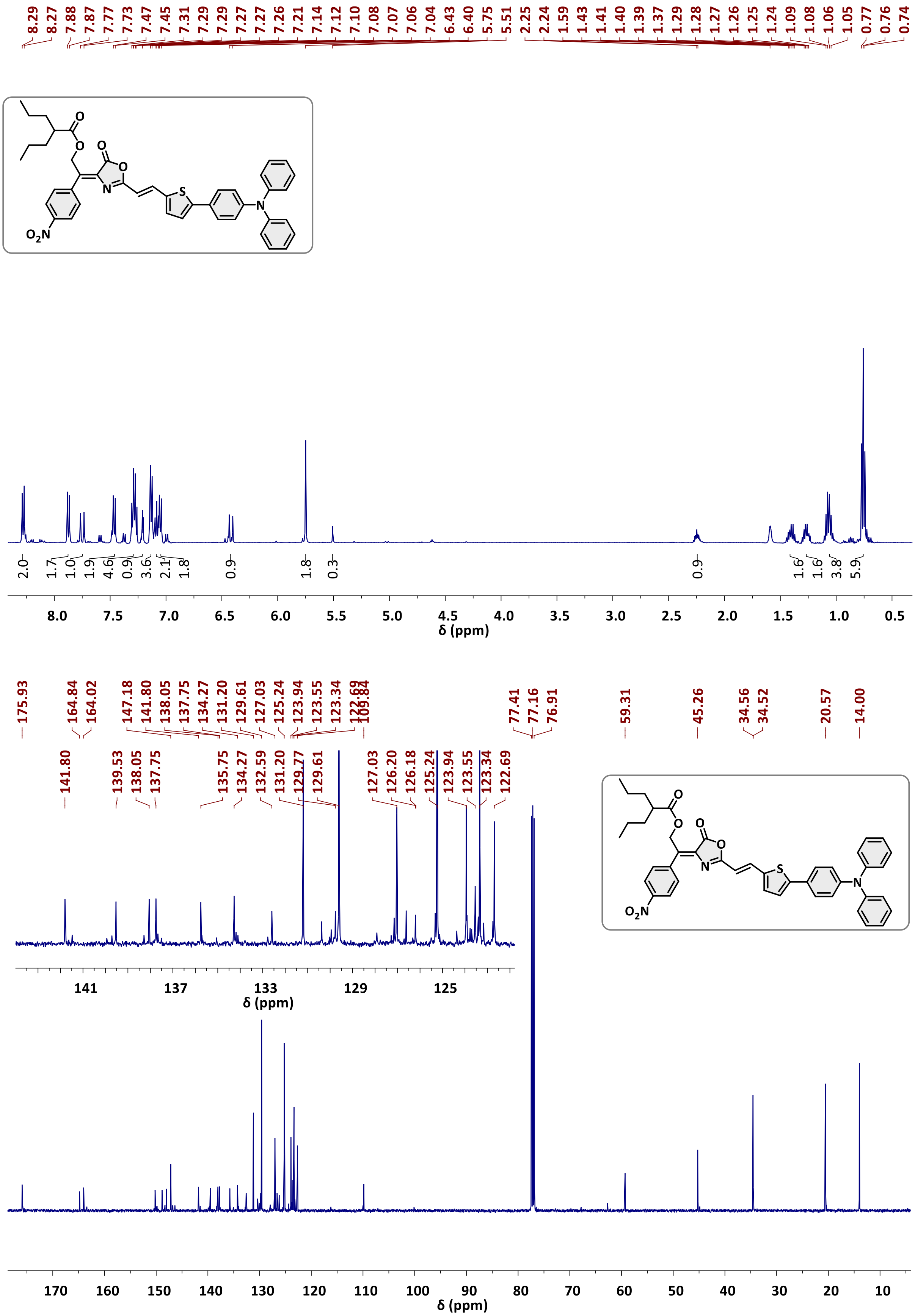

Figure S41: ^{1}H and ^{13}C NMR of 15F.

4. HRMS Analysis of all the photocages:

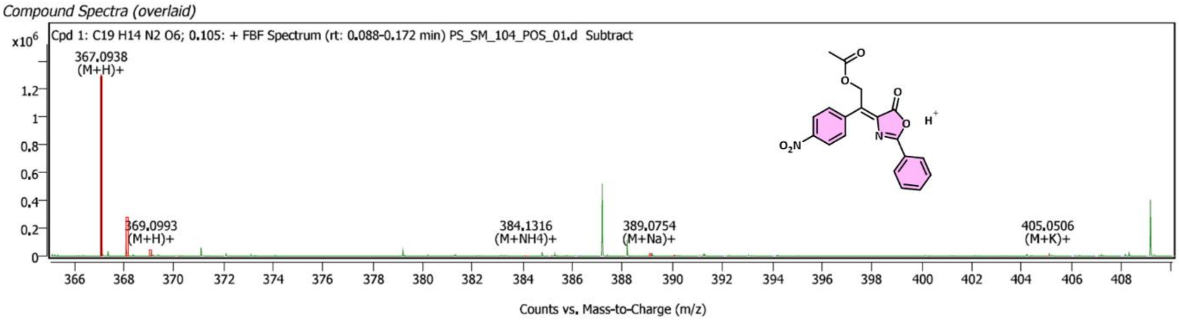

Compound ID Table

Name	Formula	Species	RT	RT Diff	Mass	CAS	ID Source	Score	Score (Lib)	Score (Tgt)
	C19 H14 N2 O6	(M+H)+ (M+NH4)+ (M+Na)+ (M+K)+	0.105		366.0865		FBF	91.72		91.72

Figure S42: HRMS of photocage 3A.

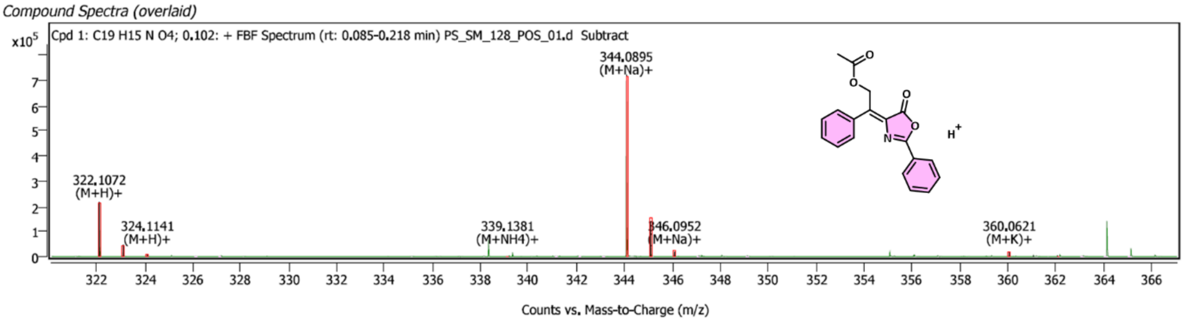

Compound ID Table

Name	Formula	Species	RT	RT Diff	Mass	CAS	ID Source	Score	Score (Lib)	Score (Tgt)
	C19 H15 N O4	(M+H)+ (M+NH4)+ (M+Na)+ (M+K)+	0.102		321.1002		FBF	99.87		99.87

Figure S43: HRMS of photocage 3B.

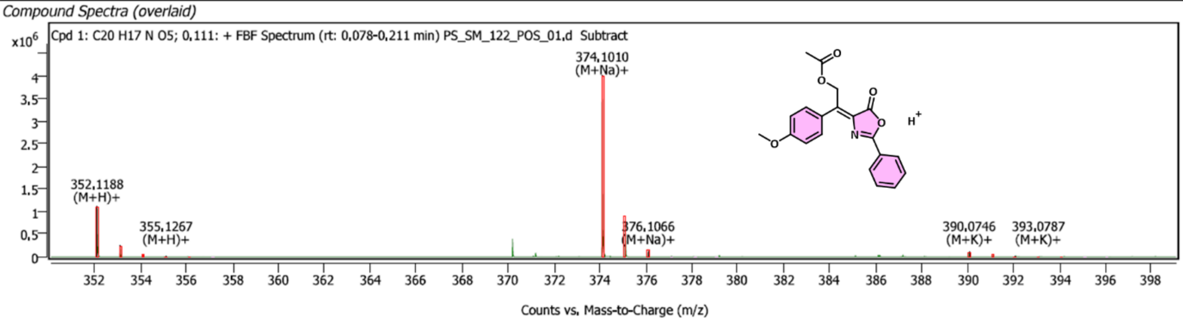

Compound ID Table

Name	Formula	Species	RT	RT Diff	Mass	CAS	ID Source	Score	Score (Lib)	Score (Tgt)
	C20 H17 N O5	(M+H)+ (M+Na)+ (M+K)+	0.111		351.1117		FBF	97.72		97.72

Figure S44: HRMS of photocage 3C.

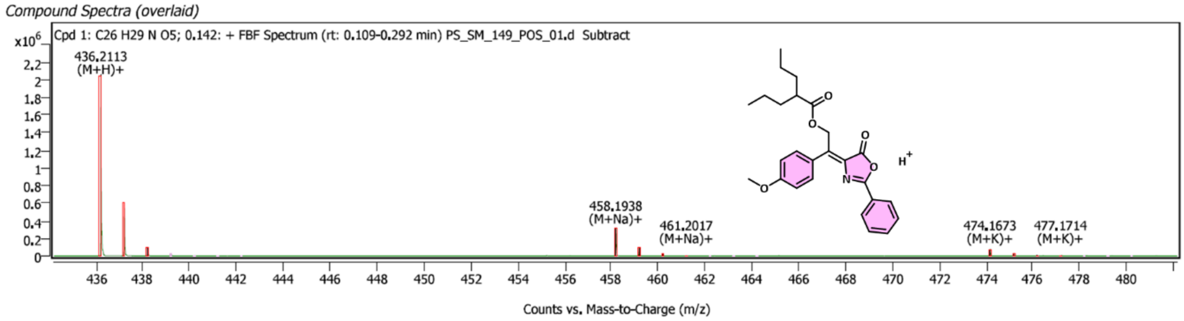

Compound ID Table

Name	Formula	Species	RT	RT Diff	Mass	CAS	ID Source	Score	Score (Lib)	Score (Tgt)
	C26 H29 N O5	(M+H)+<br>(M+Na)+<br>(M+K)+	0.142		435.2041		FBF	99.39		99.39

Figure S45: HRMS of photocage 3F.

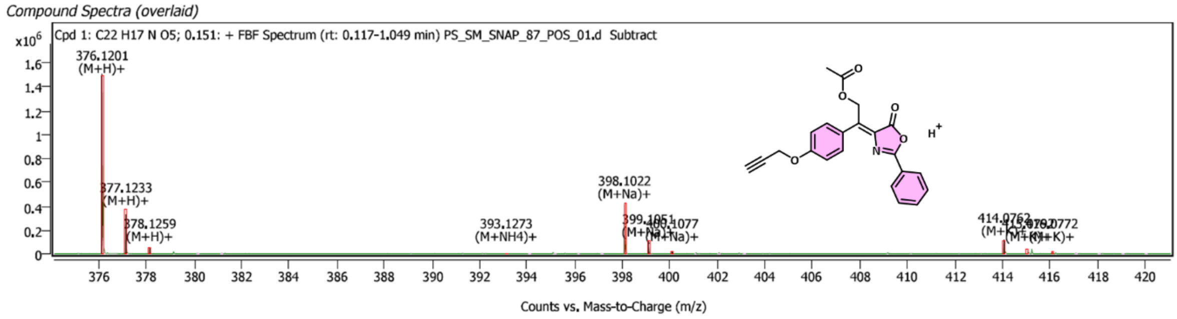

Compound ID Table

Name	Formula	Species	RT	RT Diff	Mass	CAS	ID Source	Score	Score (Lib)	Score (Tgt)
	C22 H17 N O5	(M+H)+<br>(M+NH4)+<br>(M+Na)+<br>(M+K)+	0.151		375.1128		FBF	88.23		88.23

Figure S46: HRMS of photocage 3D.

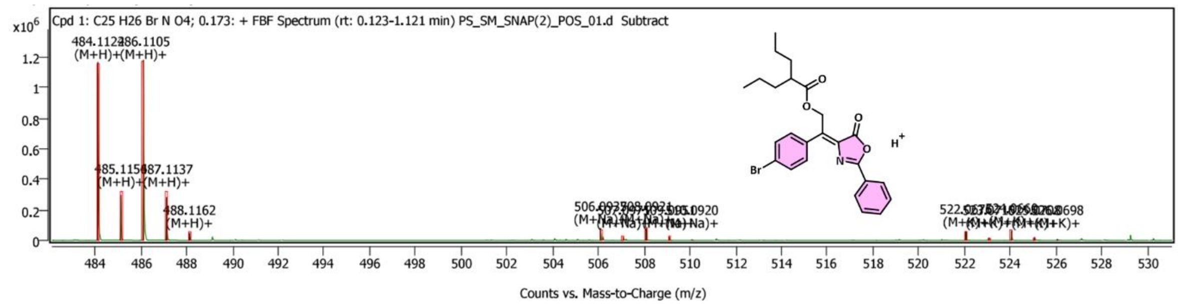

Compound ID Table

Name	Formula	Species	RT	RT Diff	Mass	CAS	ID Source	Score	Score (Lib)	Score (Tgt)
	C25 H26 Br N O4	(M+H)+<br>(M+Na)+<br>(M+K)+	0.173		483.1049		FBF	99.65		99.65

Figure S47: HRMS of photocage 3E.

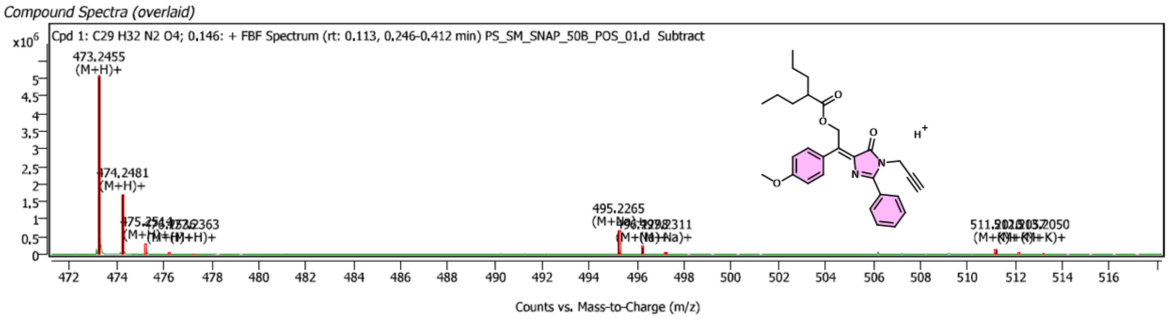

Compound ID Table

Name	Formula	Species	RT	RT Diff	Mass	CAS	ID Source	Score	Score (Lib)	Score (Tgt)
	C29 H32 N2 O4	(M+H)+ (M+Na)+ (M+K)+	0,146		472,2379		FBF	93,62		93,62

Figure S48: HRMS of photocage 6.

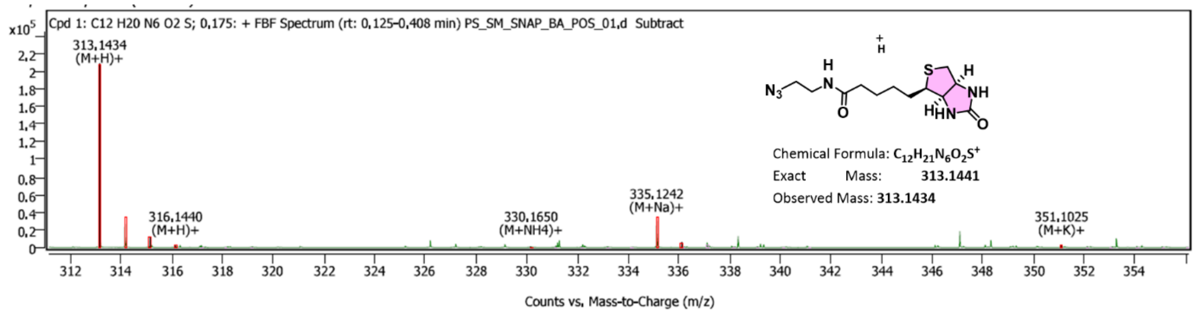

Compound ID Table

Name	Formula	Species	RT	RT Diff	Mass	CAS	ID Source	Score	Score (Lib)	Score (Tgt)
	C12 H20 N6 O2 S	(M+H)+ (M+NH4)+ (M+Na)+ (M+K)+	0,175		312,1360		FBF	98,12		98,12

Figure S49: HRMS of compound 8.

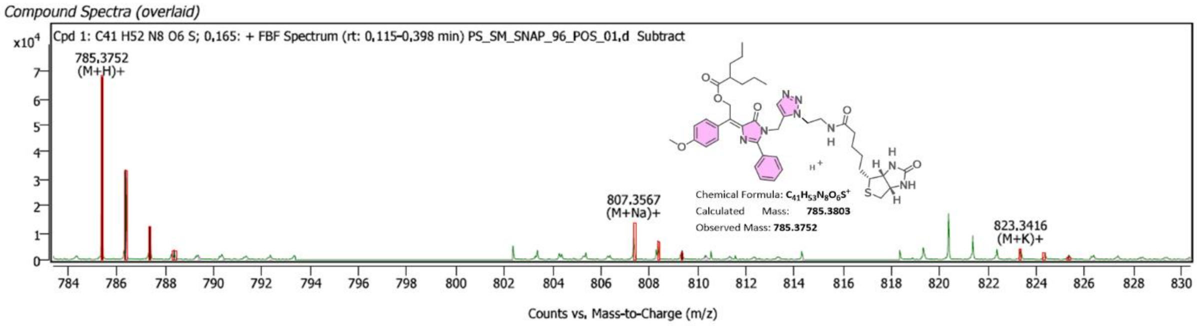

Compound ID Table

Name	Formula	Species	RT	RT Diff	Mass	CAS	ID Source	Score	Score (Lib)	Score (Tgt)
	C41 H52 N8 O6 S	(M+H)+ (M+Na)+ (M+K)+	0,165		784,3679		FBF	75,82		75,82

Figure S50: HRMS of photocage 9.

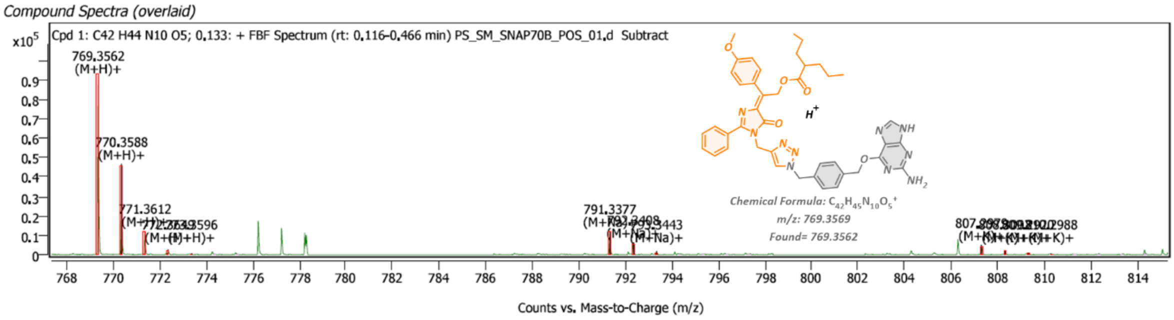

Compound ID Table

Name	Formula	Species	RT	RT Diff	Mass	CAS	ID Source	Score	Score (Lib)	Score (Tgt)
	C42 H44 N10 O5	(M+H)+ (M+Na)+ (M+K)+	0.133		768.3480		FBF	98.83		98.83

Figure S51: HRMS of photocage 7.

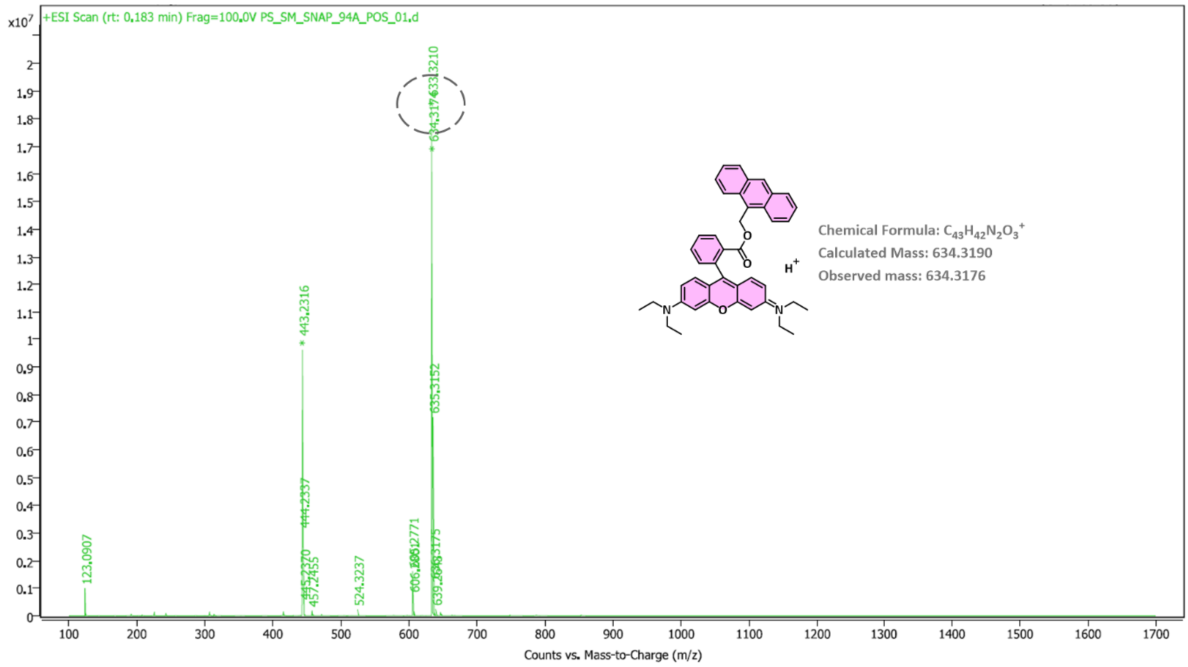

Figure S52: HRMS of compound SM-94.

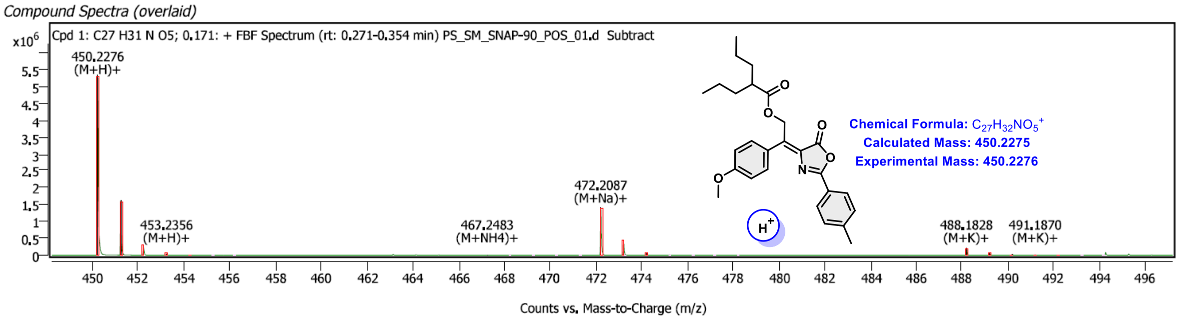

Compound ID Table

Name	Formula	Species	RT	RT Diff	Mass	CAS	ID Source	Score	Score (Lib)	Score (Tgt)
	C27 H31 N O5	(M+H)+ (M+NH4)+ (M+Na)+ (M+K)+	0.171		449.2200		FBF	99.06		99.06

Figure S53: HRMS of photocage 15A

Compound Spectra (overlaid)

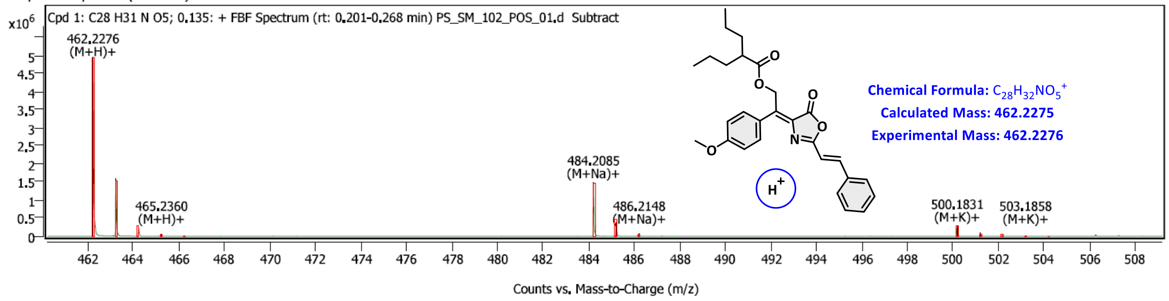

Compound ID Table

Name	Formula	Species	RT	RT Diff	Mass	CAS	ID Source	Score	Score (Lib)	Score (Tgt)
	C28 H31 N O5	(M+H)+<br>(M+Na)+<br>(M+K)+	0.135		461.2199		FBF	98.87		98.87

Figure S54: HRMS of photocage 15B

Compound Spectra (overlaid)

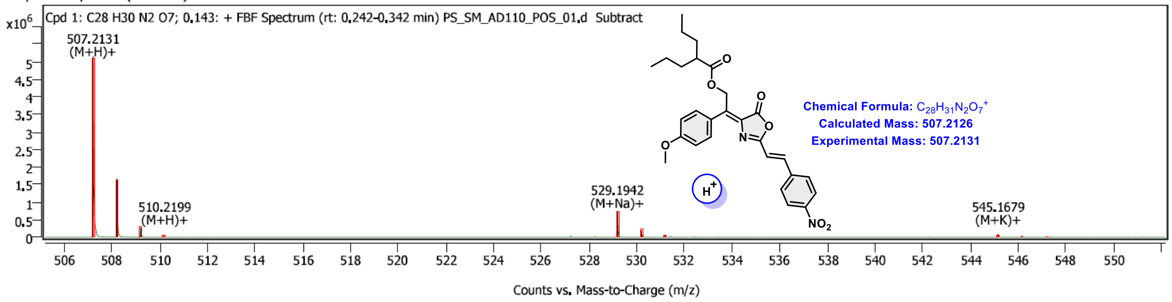

Compound ID Table

Name	Formula	Species	RT	RT Diff	Mass	CAS	ID Source	Score	Score (Lib)	Score (Tgt)
	C28 H30 N2 O7	(M+H)+<br>(M+Na)+<br>(M+K)+	0.143		506.2055		FBF	98.82		98.82

Figure S55: HRMS of photocage 15C

Compound Spectra (overlaid)

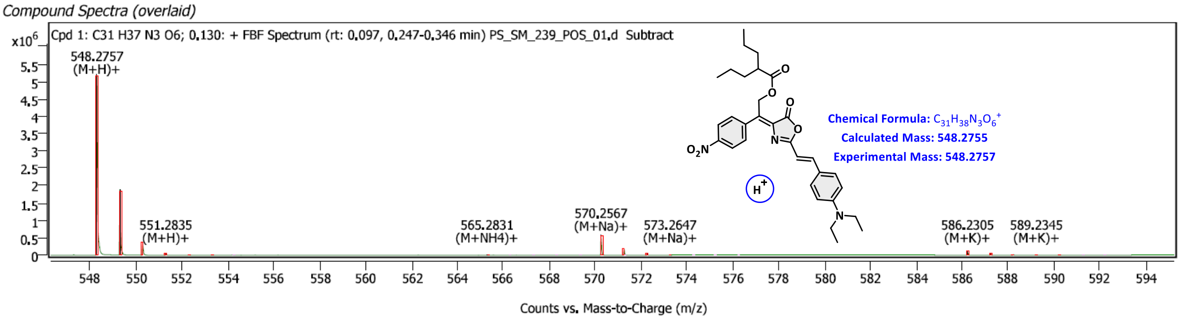

Compound ID Table

Name	Formula	Species	RT	RT Diff	Mass	CAS	ID Source	Score	Score (Lib)	Score (Tgt)
	C31 H37 N3 O6	(M+H)+<br>(M+NH4)+<br>(M+Na)+<br>(M+K)+	0.130		547.2681		FBF	98.78		98.78

Figure S56: HRMS of photocage 15D

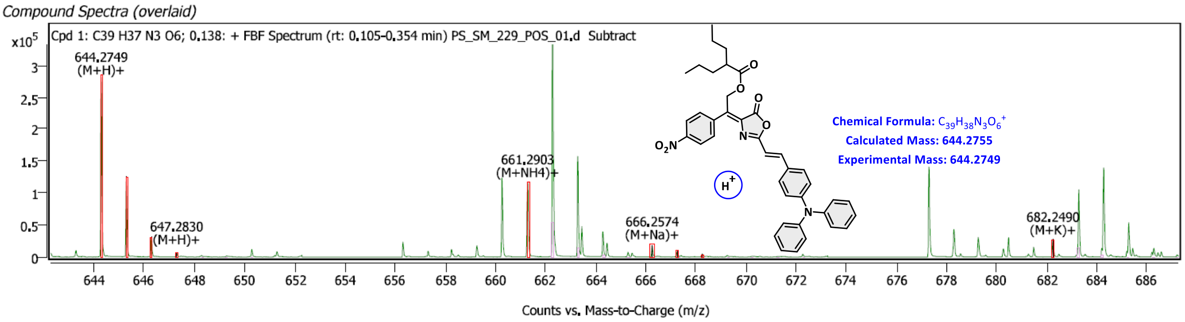

Compound ID Table

Name	Formula	Species	RT	RT Diff	Mass	CAS	ID Source	Score	Score (Lib)	Score (Tgt)
	C39 H37 N3 O6	(M+H)+ (M+NH4)+ (M+Na)+ (M+K)+	0.138		643.2661		FBF	97.97		97.97

Fig S57: HRMS of photocage 15E

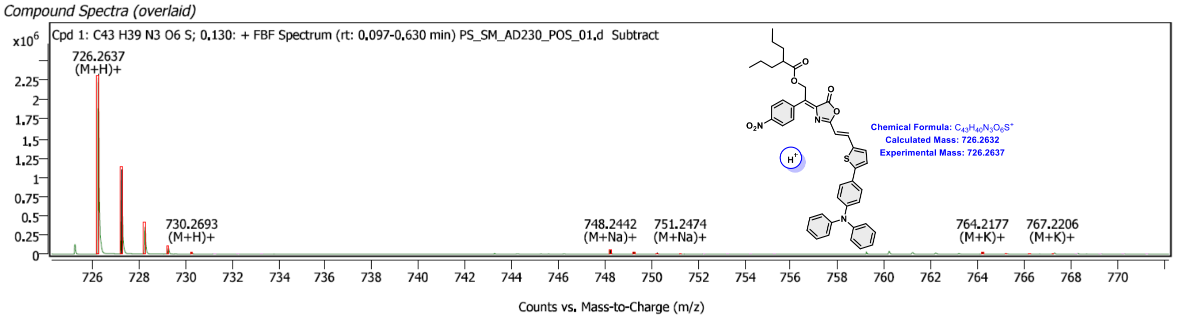

Compound ID Table

Name	Formula	Species	RT	RT Diff	Mass	CAS	ID Source	Score	Score (Lib)	Score (Tgt)
	C43 H39 N3 O6 S	(M+H)+ (M+Na)+ (M+K)+	0.130		725.2564		FBF	98.97		98.97

Figure S58: HRMS of photocage 15F

5. Photophysical Characterizations of the GFP-photocages:

The UV-vis absorption and fluorescence characterization of all the GFP-photocages have been carried out at 10^{-4}-10^{-5} M concentration in different solvents. To measure the fluorescence properties and singlet state lifetime of all the photocages in anaerobic conditions, compounds were dissolved in acetonitrile, followed by cooling to 0 °C and degassed with argon gas. All the photophysical characterizations are summarised in **Table 1**.

The fluorescent quantum yields (QY) of all the synthesized photocages **3A**, **3B**, **3D, 3E, 3F,** and **6** were determined by the reference point method. Quinine Sulphate in 1(M) H_2SO_4 (literature quantum yield: 95%) was used as the standard sample to calculate the fluorescent QYs of the photocages using **equation 1** (Where **Φ** represents quantum yield, **Abs** represents absorbance, **A** represents the area of the fluorescence curve, and **η** is the refractive index of the medium. The subscripts **S** and **R** denote the corresponding parameters for the sample and reference, respectively).

Equation 1: $$\frac{\Phi_S}{\Phi_R} = \frac{A_S}{A_R}\,\frac{(Abs)_R}{(Abs)_S}\,\frac{{\eta_S}^2}{{\eta_R}^2}$$

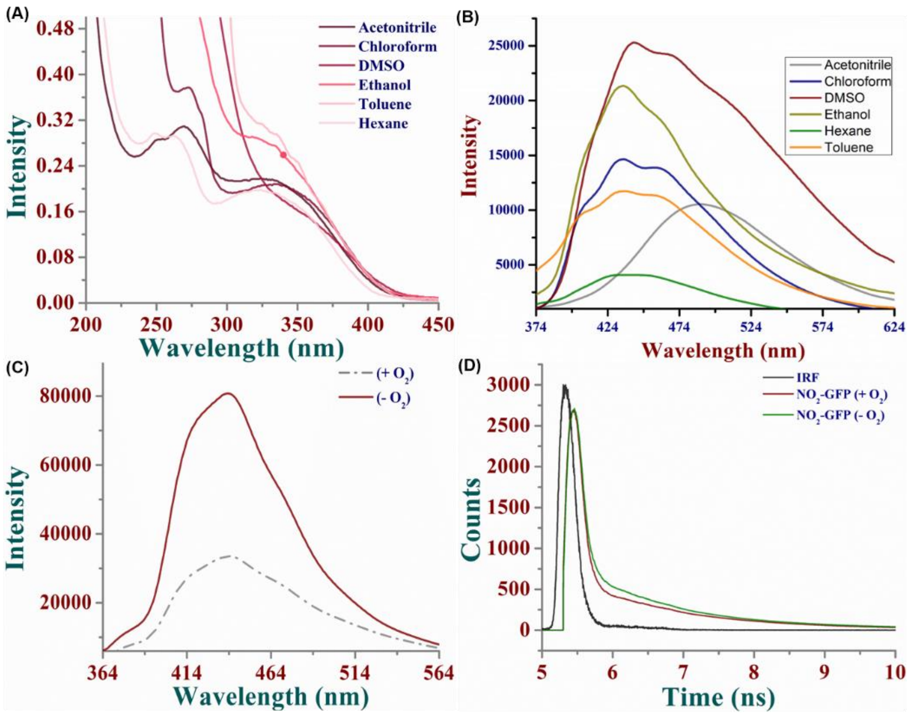

Figure S59: (A) UV-vis spectra and (B) fluorescence spectra of NO_2-GFP (Photocage 3A) $1{\times}10^{-5}$ M concentration in different solvents. (C) Oxygen-dependent fluorescence spectra of NO_2-GFP ($2{\times}10^{-5}$ M) in DMSO. (D) Oxygen-dependent time-correlated single photon counts (TCSPC) of NO_2-GFP ($2{\times}10^{-5}$ M) in DMSO.

Photophysical Characterization of Simple-GFP

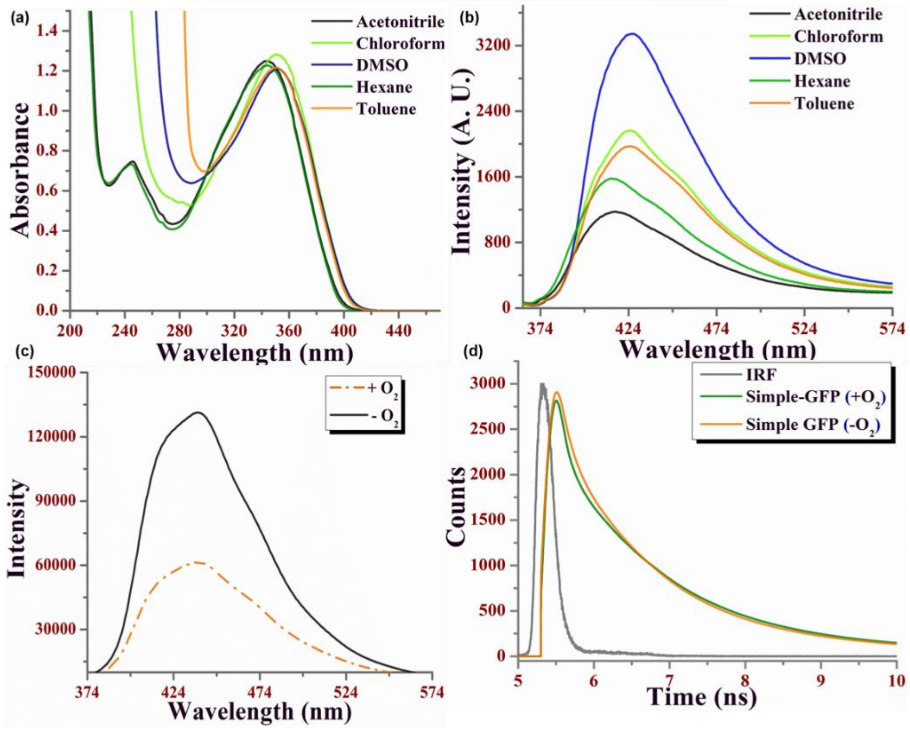

Figure S60: (A) UV-vis spectra and (B) fluorescence spectra of Simple-GFP (Photocage **3B**) $1{\times}10^{-5}$ M concentration in different solvents. (C) Oxygen-dependent fluorescence spectra of **3B** ($2{\times}10^{-5}$ M) in DMSO. (D) Oxygen-dependent time-correlated single photon counts (TCSPC) of **3B** ($2{\times}10^{-5}$ M) in DMSO.

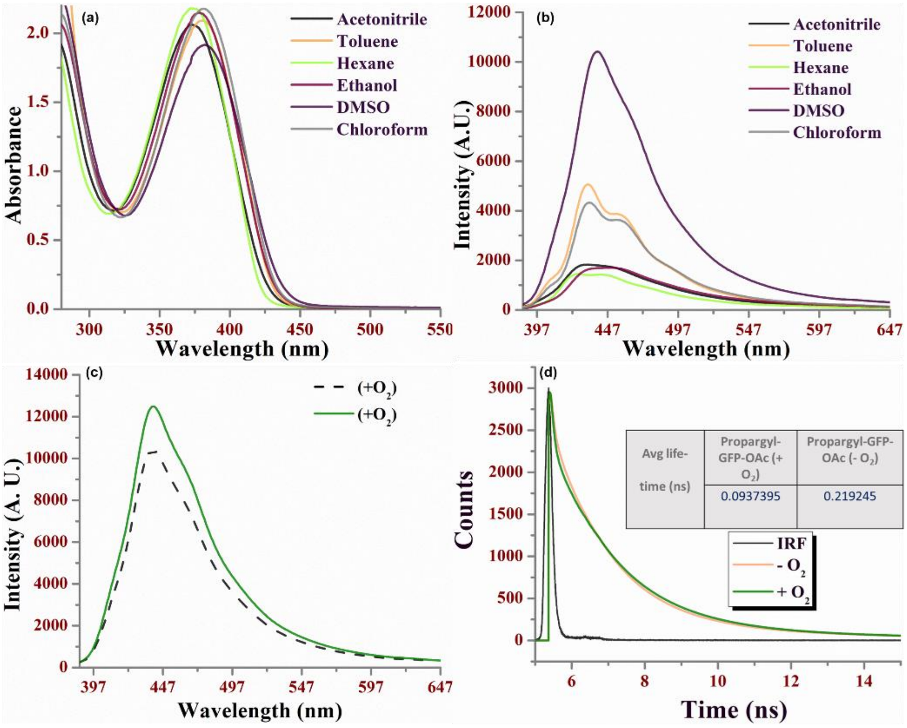

Figure S61: (A) UV-vis spectra and (B) fluorescence spectra of Simple-GFP (Photocage **3D**) 1×10^{-5} M concentration in different solvents. (C) Oxygen-dependent fluorescence spectra of **3D** (2×10^{-5} M) in DMSO. (D) Oxygen-dependent time-correlated single photon counts (TCSPC) of **3D** (2×10^{-5} M) in DMSO.

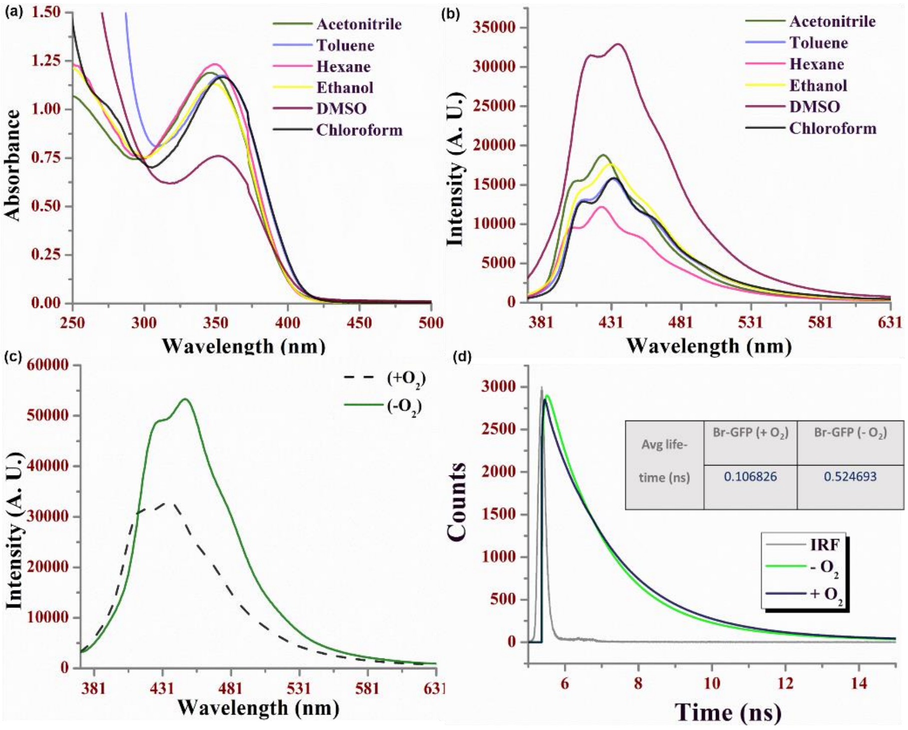

Figure S62: (A) UV-vis spectra and (B) fluorescence spectra of Simple-GFP (Photocage **3E**) 1×10^{-5} M concentration in different solvents. (C) Oxygen-dependent fluorescence spectra of **3E** (2×10^{-5} M) in DMSO (D) Oxygen-dependent time-correlated single photon counts (TCSPC) of **3E** (2×10^{-5} M) in DMSO.

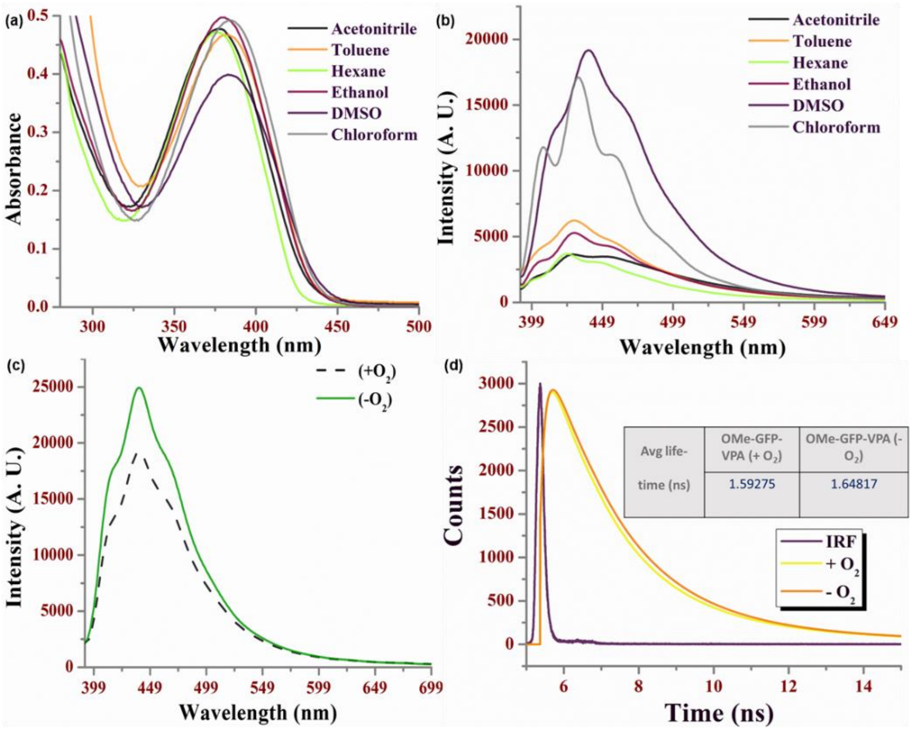

Figure S63: (A) UV-vis spectra and (B) fluorescence spectra of Simple-GFP (Photocage **3F**) 1×10^{-5} M concentration in different solvents. (C) Oxygen-dependent fluorescence spectra of **3F** (2×10^{-5} M) in DMSO (D) Oxygen-dependent time-correlated single photon counts (TCSPC) of **3F** (2×10^{-5} M) in DMSO.

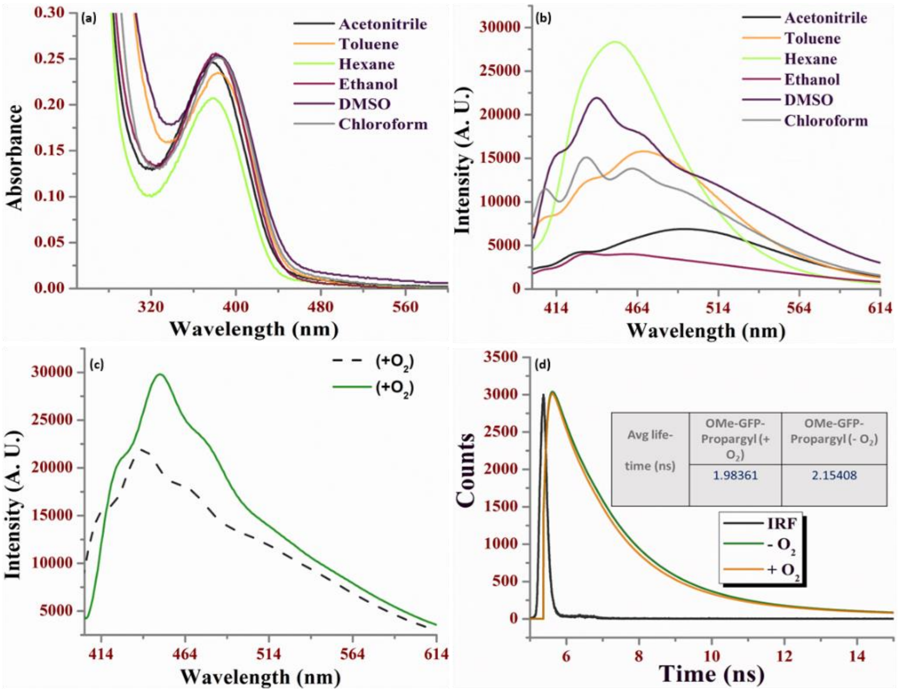

Figure S64: (A) UV-vis spectra and (B) fluorescence spectra of Simple-GFP (Photocage **6**) 1×10^{-5} M concentration in different solvents. (C) Oxygen-dependent fluorescence spectra of **6** (2×10^{-5} M) in DMSO. (D) Oxygen-dependent time-correlated single photon counts (TCSPC) of **6** (2×10^{-5} M) in DMSO.

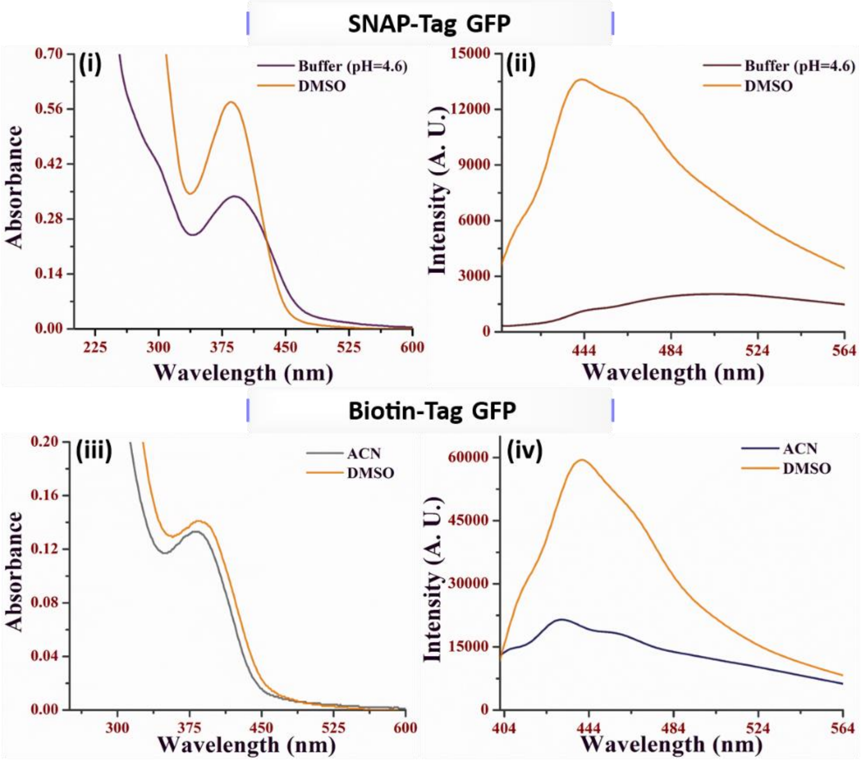

Figure S65: (A) UV-vis spectra and (B) fluorescence spectra of Simple-GFP (Photocage **6**) 1×10^{-5} M concentration in different solvents. (C) Oxygen-dependent fluorescence spectra of **6** (2×10^{-5} M) in DMSO. (D) Oxygen-dependent time-correlated single photon counts (TCSPC) of **6** (2×10^{-5} M) in DMSO.

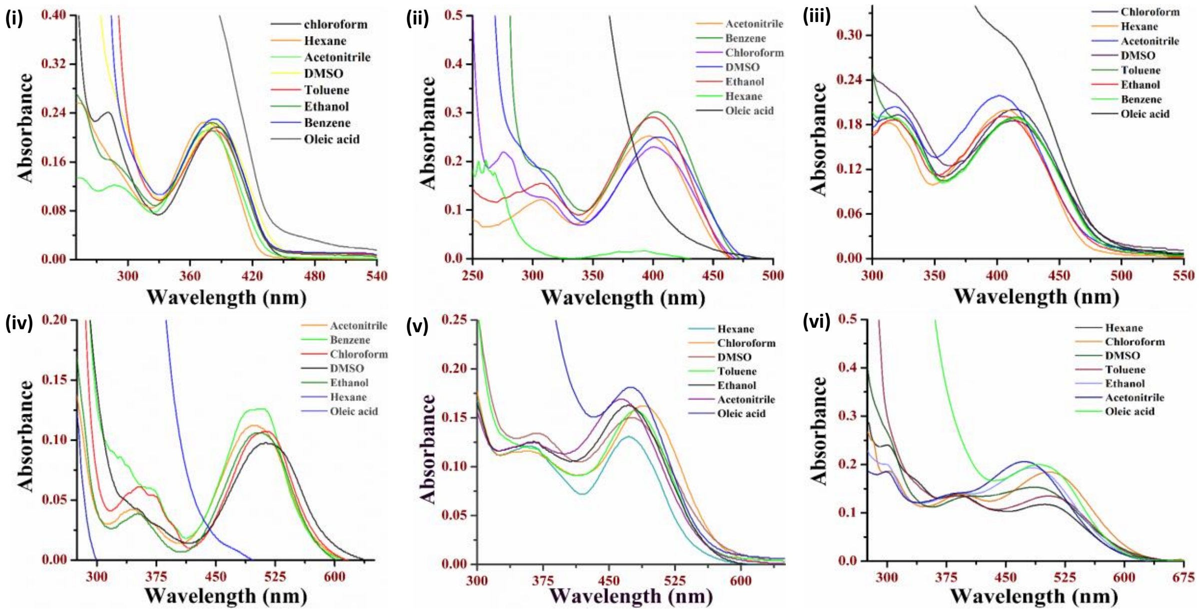

Figure S66: UV-vis spectra of Photocage (i) 15A, (ii) 15B, (iii) 15C, (iv) 15D, (v) 15E, and (vi) 15F at 1×10^{-5} M concentration in different solvents.

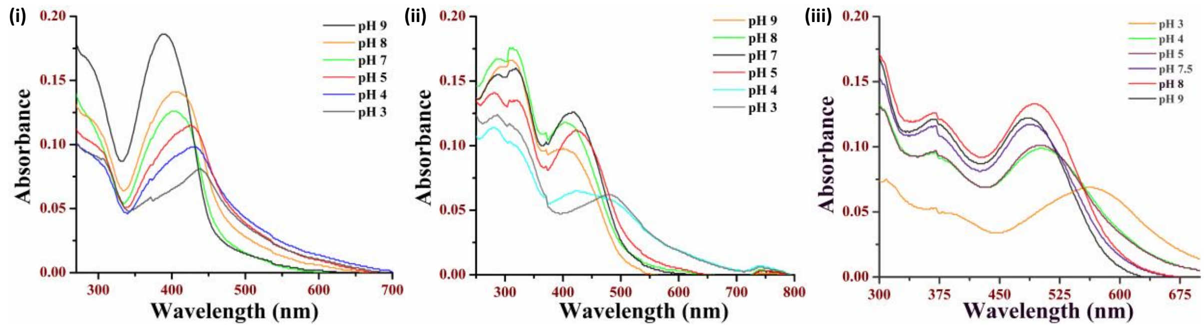

Figure S67: UV-vis spectra of Photocage (i) 15A, (ii) 15C, and (iii) 15E at 1×10^{-5} M concentration in different pH mediums.

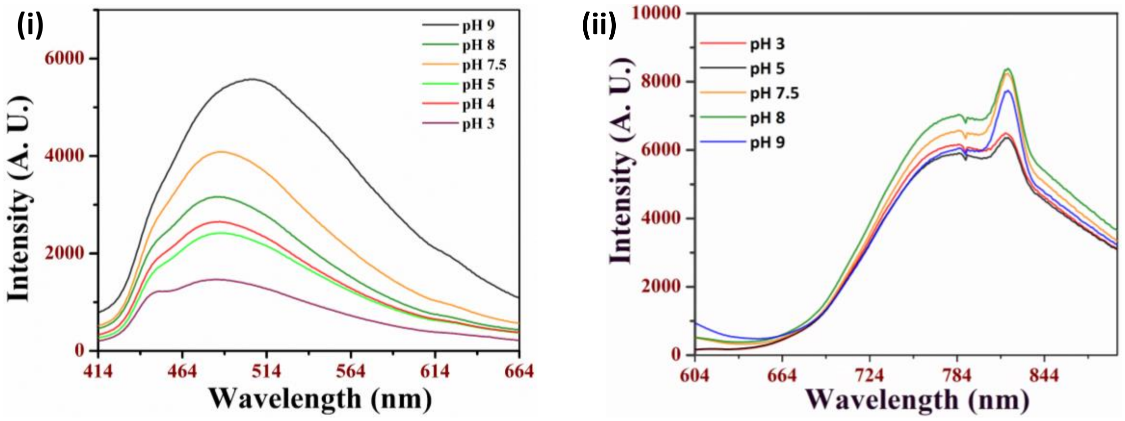

Figure S68: Emission spectra of Photocage (i) 15A and (ii) 15E at 1×10^{-5} M concentration in different pH mediums.

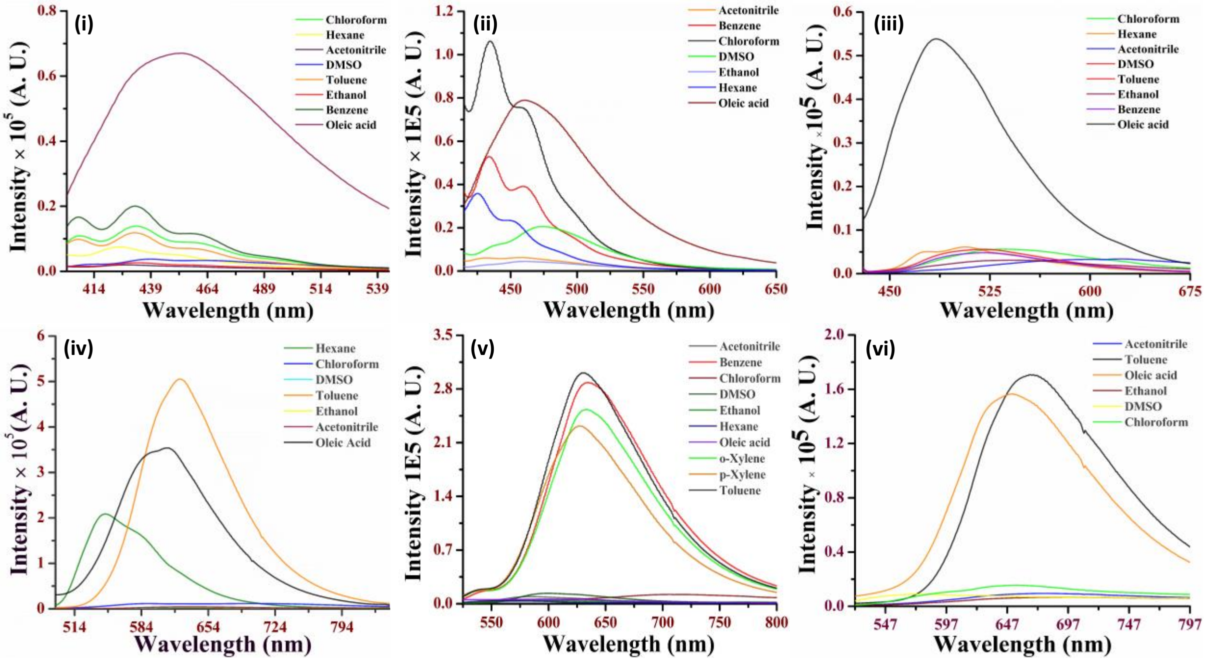

Figure S69: Emission spectra of Photocage (i) 15A, (ii) 15B, (iii) 15C, (iv) 15D, (v) 15E, and (vi) 15F at 1×10^{-5} M concentration in different solvents.

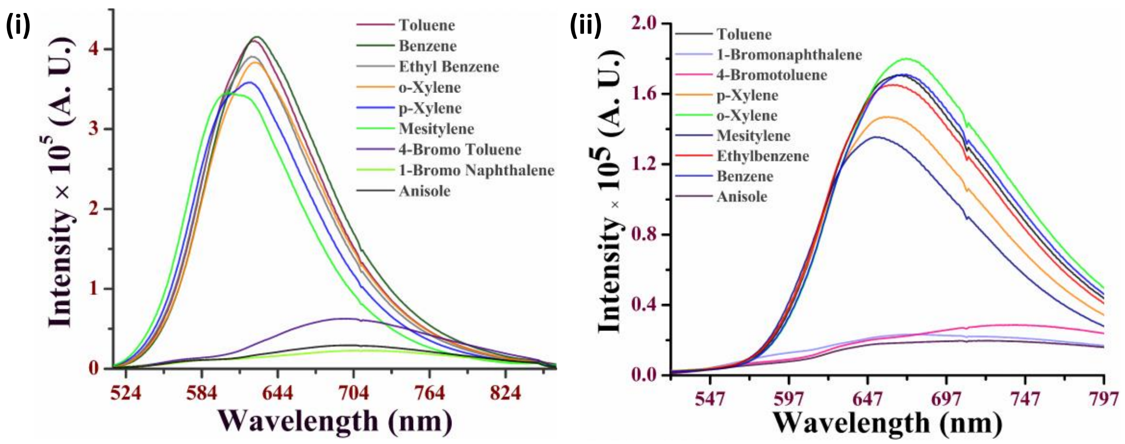

Figure S70: Emission spectra of Photocage (i) 15E and (ii) 15F at 1×10^{-5} M concentration in different aromatic solvents.

6. Determination of Photon Flux (I_0):

To determine the photon flux of the incident light source, indolylfulgide-based chemical actinometry was used. The starting indolylfulgide compound (**10**) was gifted by Prof. Tomas Slanina. A fresh solution of compound 10 (3 ml, 0.6 ×10^{-3} M) was prepared in toluene and taken in a fluorescence cuvette (equipped with a stir bar). No further light irradiation was done to convert **10C** to **10Z**, as we have seen the compound is already in the Z-isomeric form (**10Z**, checked with UV-Vis spectrophotometer). Further, change in the absorption spectra was monitored (**Figure S47b**, **S47c** for ≥ 410 nm and **Figure S47d**, **S47e** for ≥ 365 nm) with an increase in the light irradiation (≥ 365 nm, 0.1 M $CuSO_4$ filter, and ≥ 410 nm, 1 M $NaNO_2$ filter) time. The photon flux was calculated for the conversion of **10Z** to **10C**, using **equation 2,** and was used for the calculation of photochemical-quantum yield.

$$\textbf{Equation 2:}\quad I_0 = \frac{\Delta A}{t}\frac{V}{\varepsilon d \Phi}$$

Where 'I_0' is the photon flux of the incident light (Einstein s^{-1}), 'ΔA' is the growth of the closed form (**10C**) in time 't', 'V' is the sample volume in (ml), 'ε' is the molar absorption coefficient of **10C** at the wavelength used to monitor the reaction (M^{-1} cm^{-1}), 'd' is the optical pathway (cm), and 'Φ' is the quantum yield of the reaction.

Table S1: Determination of photon flux of the different light sources:

Incident light	Slope (ΔA/t)	Sample volume (cm^3)	ε (M^{-1} cm^{-1})	d (cm)	φ	Photon Flux (Einstein S^{-1})
≥ 365 nm	0.00418 ± 0.00076	3	1513	1	19.8	2.63×10^{-7}
≥ 410 nm	0.00395 ± 0.000326	3	5115	1	31.5	1.17×10^{-7}

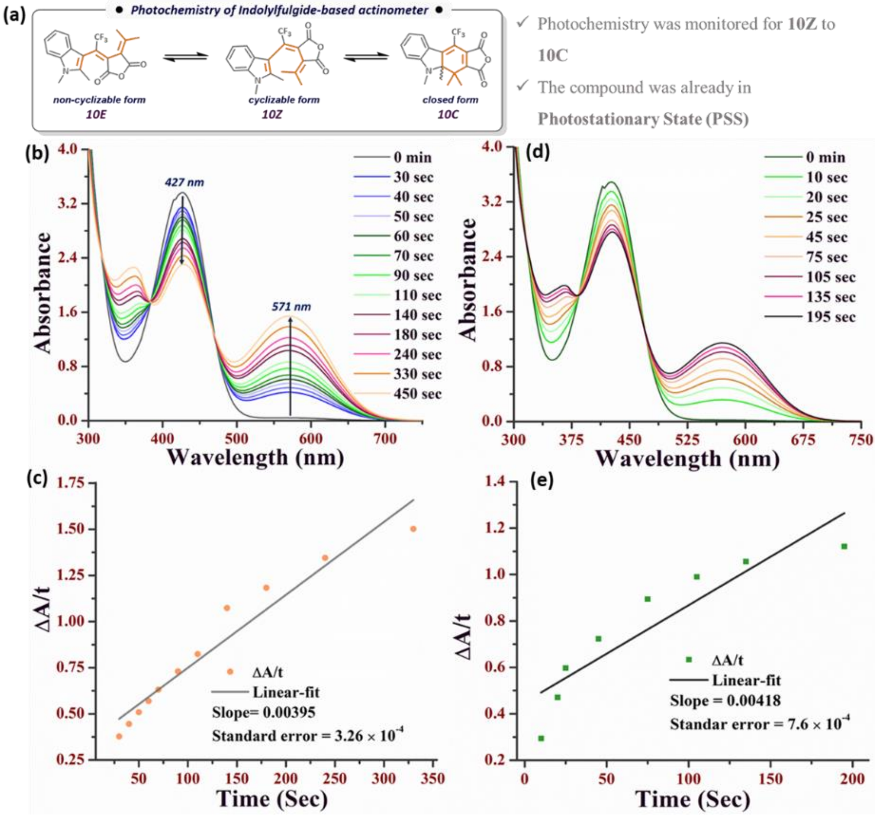

Figure S71: Change in the UV-Vis absorption spectrum of indolylfulgide under (a) ≥ 365 nm and (b) ≥ 410 nm light irradiation. Linear fitting of (ΔA/t) vs time (sec) of (c) ≥ 365 nm and (d) ≥ 410 nm light irradiation.

6a. Specification of the Light Source:

The luminous intensity of the light source was measured by a digital luminometer provided by Thors Lab. The diameter of the detector was 2 cm. The luminous intensity was 50.7 mW/cm^2 for ≥ 365 nm and 39 mW/cm^2 for ≥ 410 nm of our used light source.

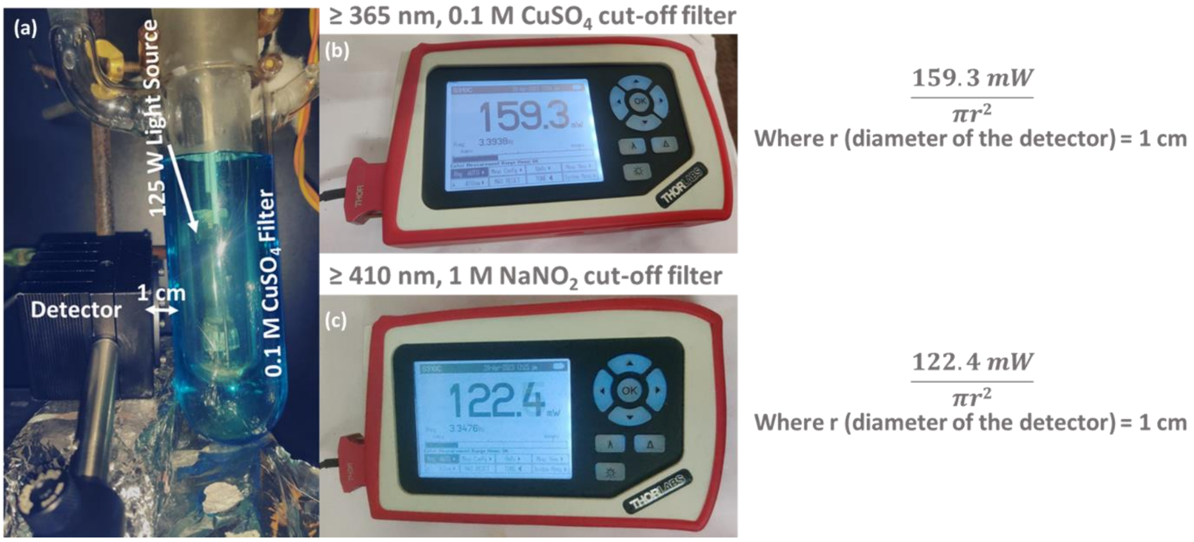

Figure S72: Photographs of the experimental set-up during the photolysis.

7. Photochemical characterizations of the GFP-photocages:

Photorelease Study of Photocage 3A:

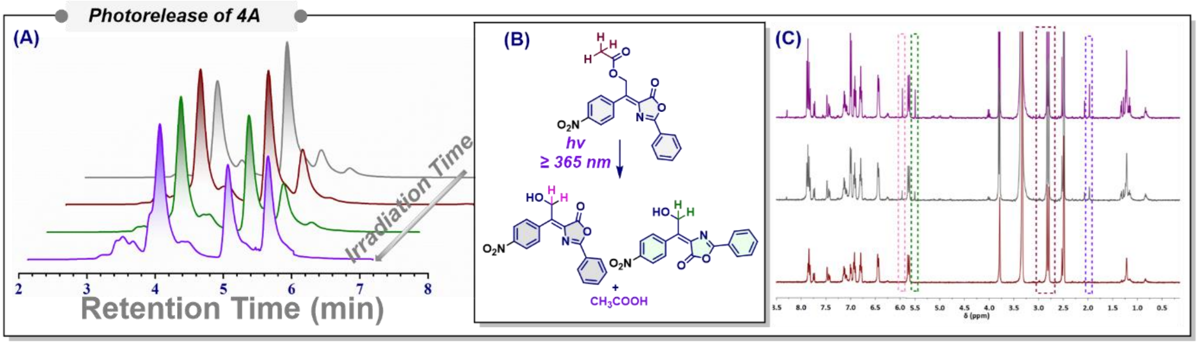

Figure S73: The course of photolysis of NO_2-GFP has been monitored by (ACN : H_2O 7:3, degassed, ≥ 365 nm Hg-lamp) (A) RP-HPLC, indicating the clean photorelease along with the generation of a photoproduct at a retention time of 4 min (C) ^{1}H NMR-study, colour codes are indicating the corresponding hydrogens of the photocage and the photoproducts.

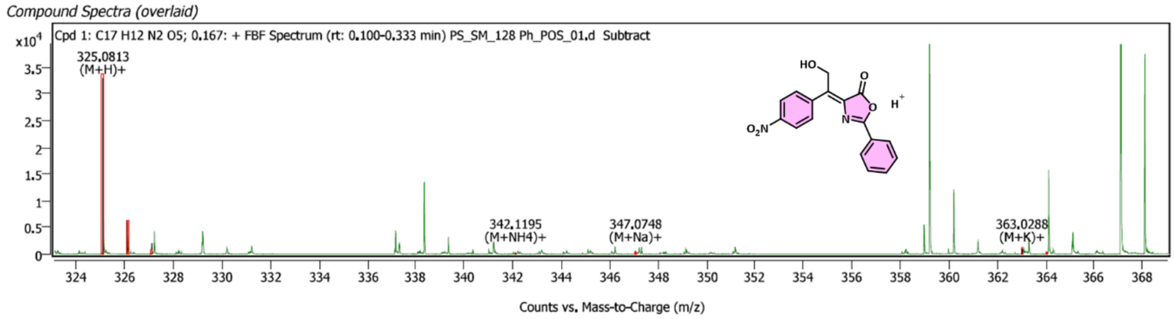

Compound ID Table

Name	Formula	Species	RT	RT Diff	Mass	CAS	ID Source	Score	Score (Lib)	Score (Tgt)
	C17 H12 N2 O5	(M+H)+ (M+NH4)+ (M+Na)+ (M+K)+	0.167		324.0744		FBF	90.68		90.68

Figure S74: The HRMS analysis of the alcohol-based photoproduct (after 1 h of light irradiation on photocage 3A).

Photorelease Study of Photocage 3B:

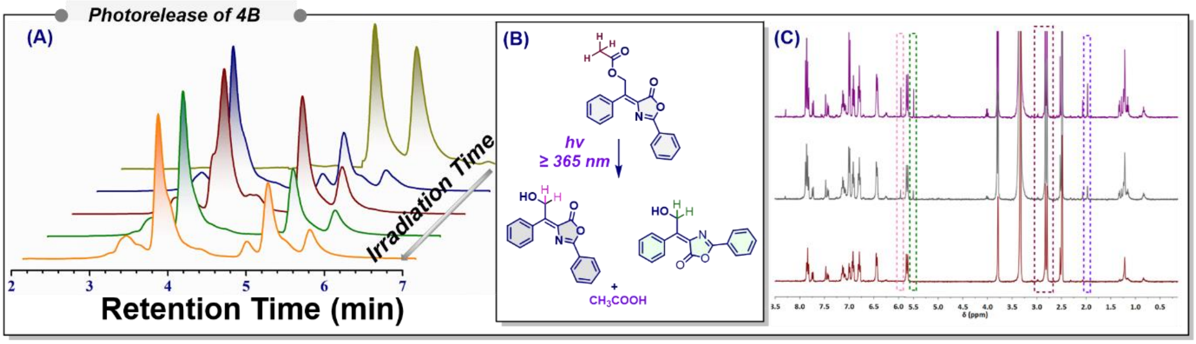

Figure S75: The course of photolysis of Simple-GFP has been monitored by (A) RP-HPLC, indicating the clean photorelease along with the generation of a photoproduct at a retention time of 4 min (ACN : H_2O 7:3, degassed, ≥ 365 nm Hg-lamp) (C) ^{1}H NMR-study, colour codes are indicating the corresponding hydrogens of the photocage and the photoproducts.

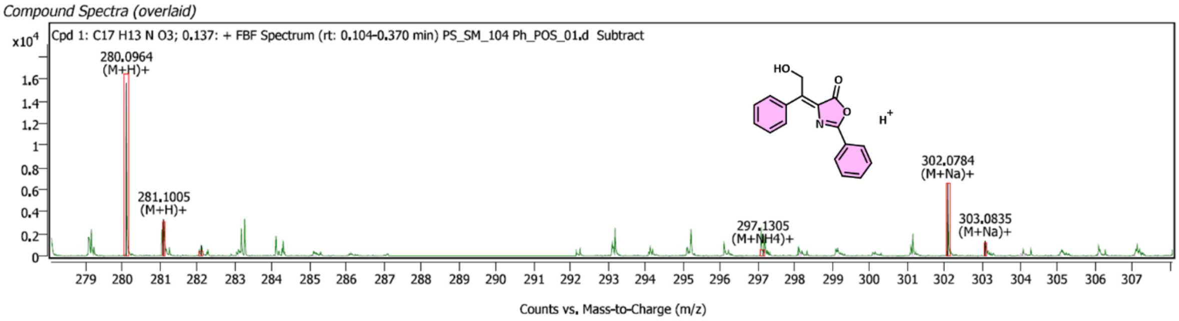

Compound ID Table

Name	Formula	Species	RT	RT Diff	Mass	CAS	ID Source	Score	Score (Lib)	Score (Tgt)
	C17 H13 N O3	(M+H)+ (M+NH4)+ (M+Na)+	0.137		279.0898		FBF	86.75		86.75

Figure S76: The HRMS analysis of the alcohol-based photoproduct (after 1 h of light irradiation on photocage 3B).

Photorelease Study of GFP-Photocages:

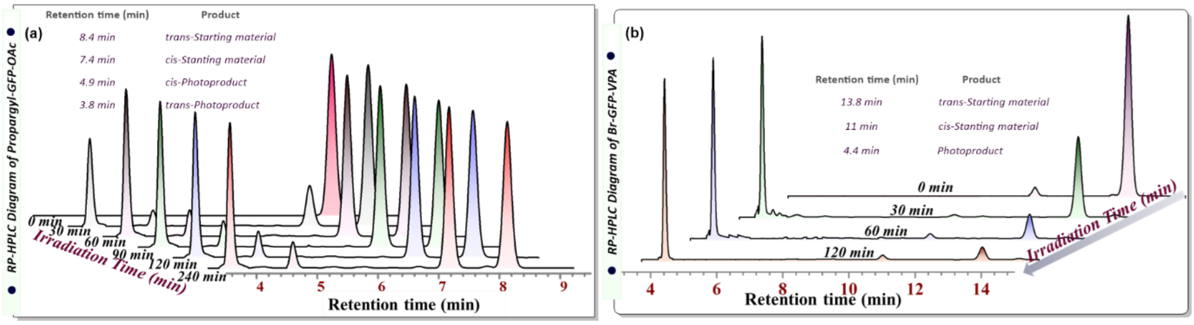

Figure S77: HPLC diagram for the photorelease of photocage (a) **3D** and (b) **3E**, with an increase in irradiation time (2 mg of photocage **3D** and **3E** dissolved in ACN : H_2O 7:3, degassed, ≥ 410 nm and ≥ 365 nm Hg-lamp respectively).

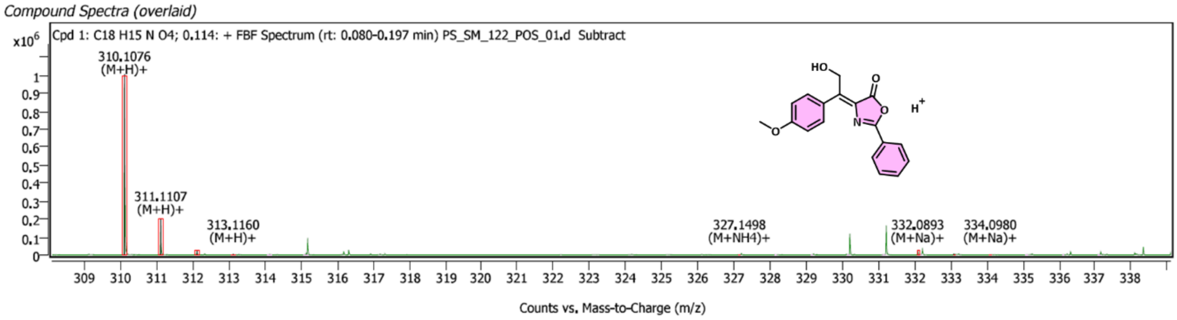

Compound ID Table

Name	Formula	Species	RT	RT Diff	Mass	CAS	ID Source	Score	Score (Lib)	Score (Tgt)
	C18 H15 N O4	(M+H)+ (M+NH4)+ (M+Na)+	0.114		309.1003		FBF	99.81		99.81

Figure S78: Photorelease of **3C** (≥ 410 nm, Hg-lamp) in ACN/water and detection of photoproduct by HRMS analysis, indicating the alcohol-based photoproduct (degassed, 3 mg photocage dissolved in 10 ml 7: 3 ACN/water).

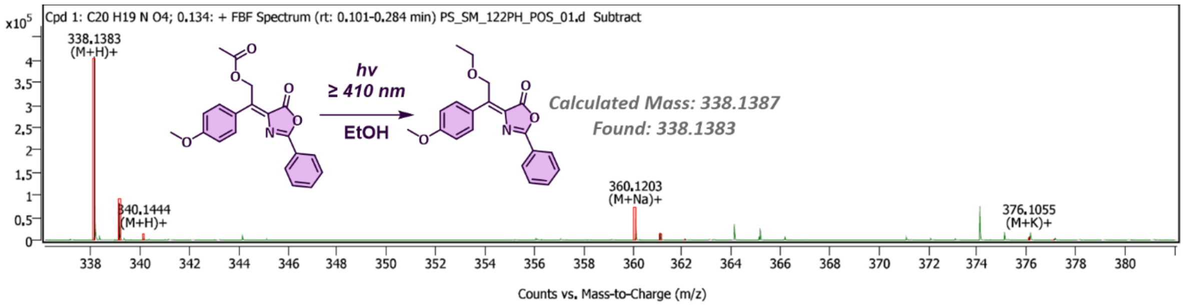

Figure S79: Photorelease of **3C** (≥ 410 nm, Hg-lamp) in ethanol and detection of photoproduct by HRMS analysis, indicating the ethoxy ether-based photoproduct (degassed, 3 mg photocage dissolved in 10 ml EtOH).

Compound Spectra (overlaid)

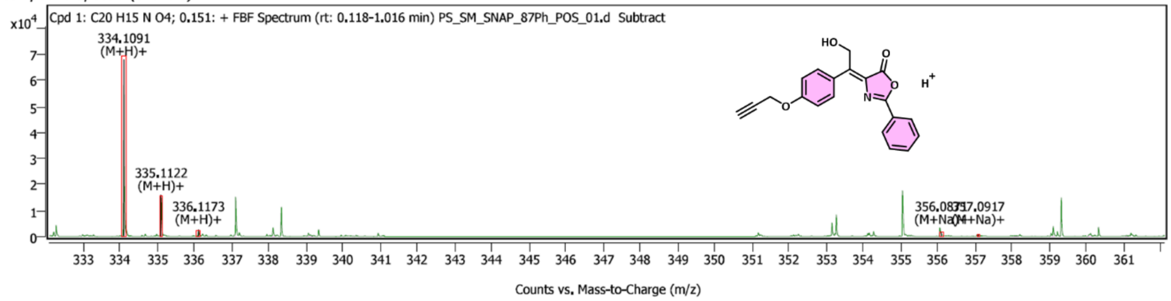

Compound ID Table

Name	Formula	Species	RT	RT Diff	Mass	CAS	ID Source	Score	Score (Lib)	Score (Tgt)
	C20 H15 N O4	(M+H)+ (M+Na)+	0.151		333.1017		FBF	90.71		90.71

Figure S80: Photorelease of **3D** (≥ 410 nm, Hg-lamp) in ACN/water and detection of photoproduct by HRMS analysis, indicating the alcohol-based photoproduct (degassed, 2 mg photocage dissolved in 10 ml 7: 3 ACN/water).

8. Quantification of Singlet oxygen:

The synthesis of the rhodamin-anthracene dye (**SM-94**) is depicted earlier (page). The dye showed a weak fluorescence due to the energy transfer from rhodamine to the anthracene moiety. After reacting with singlet oxygen, the anthracene scaffold will lead to the formation of an endoperoxide, resulting in a highly emissive moiety.

For the sensing of singlet oxygen, we have prepared a 3×10^{-3} M of stock of each GFP-photocages (in acetonitrile) and the fluorescence reporter (SM-94, in distilled water). From the stock solutions, we have taken 30 µL of each dye and the photocages. Finally, the volume was made up to 3 ml by adding distilled water. The reaction was monitored by fluorescence spectroscopy with a regular time interval with an increase in photoirradiation.

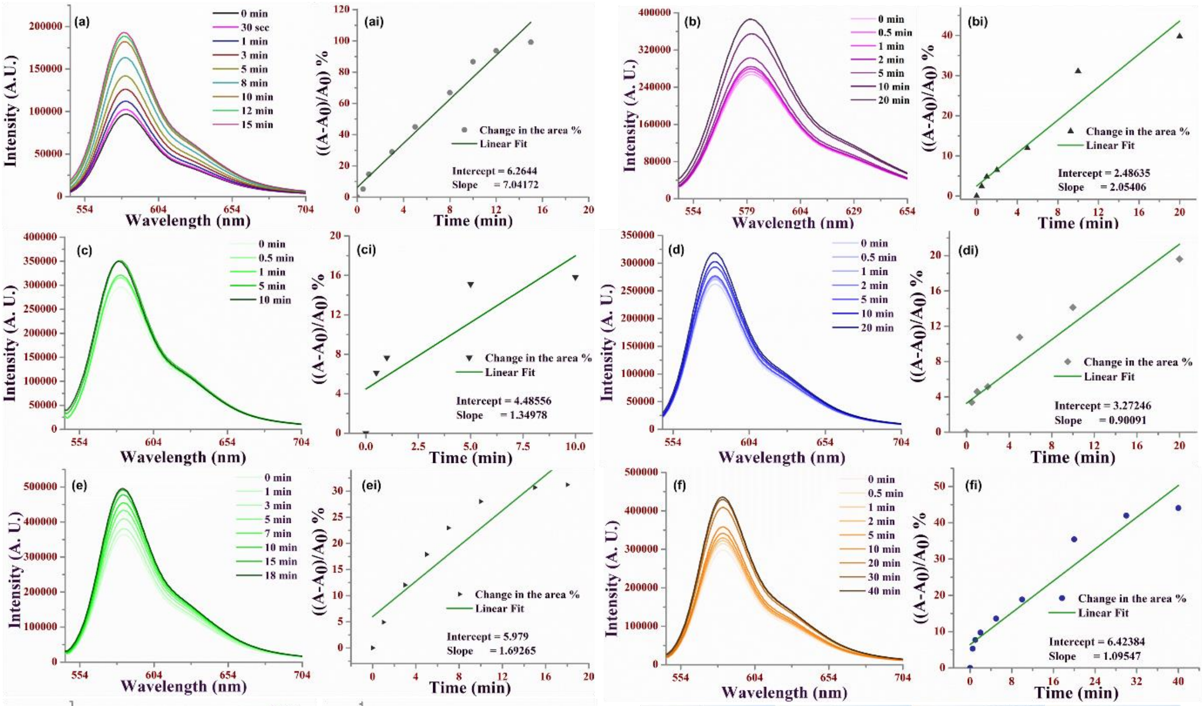

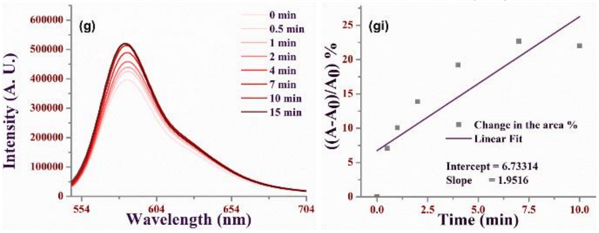

GFP-Photocages	Slope	Quantum Yield (ϕ_Δ)	% of Singlet Oxygen Generation
Phenalenone	7.04172	1 (Literature Report)	
3A	1.34978	0.19	17
3C	0.90091	0.13	18
3E	2.05406	0.3	40
3D	1.69265	0.24	30
3F	1.9516	0.28	24
6	1.09547	0.16	40

Figure S81: Change in the fluorescence intensity after sensing the photo-generated singlet oxygen using a rhodamin-anthracene conjugate dye (SM-94). Change in the fluorescence spectra of SM-94 and linear fitting curve after sensing the photo-generated singlet oxygen by (a), (ai) **phenalenone**; (b), (bi) **3A**; (c), (ci) **3C**; (d), (di) **3E**; (e), (ei) **3D**; (f), (fi) **3F**; (g), (gi) **6**.

9. TD-DFT Calculations:

Computational Details

The computational details involved in this study are as follows: The ground state geometry optimization of photocages **3c** and **3c'** was performed using the B3LYP[1-4] functional in conjunction with the def2-TZVP[5] basis set, including the D3BJ[7-8] dispersion correction and the RIJCOSX[6] approximation. For the excited state geometry optimization of **3c** and **3c'**, the CAM-B3LYP[13] functional and def2-TZVP basis set, along with the D3BJ dispersion correction and RIJCOSX approximation, were employed using the TD-DFT[10-12] method. The isomerization process from **3c** to **3c'** in the S_1 state was studied using the nudged elastic band (NEB) method, and the photodissociation in the T_1 state was investigated using the same level of theory. The analysis of natural transition orbitals (NTOs) and electron density difference plots was performed using the Multiwfn[14] software. All calculations were performed using the ORCA 5.0.3[9] software package, which provides a reliable platform for accurate and efficient computational studies.

Results and Discussion:

Figure S58 illustrates the molecular geometries of the **3c, 3c', 6c,** and **6c'** moieties.

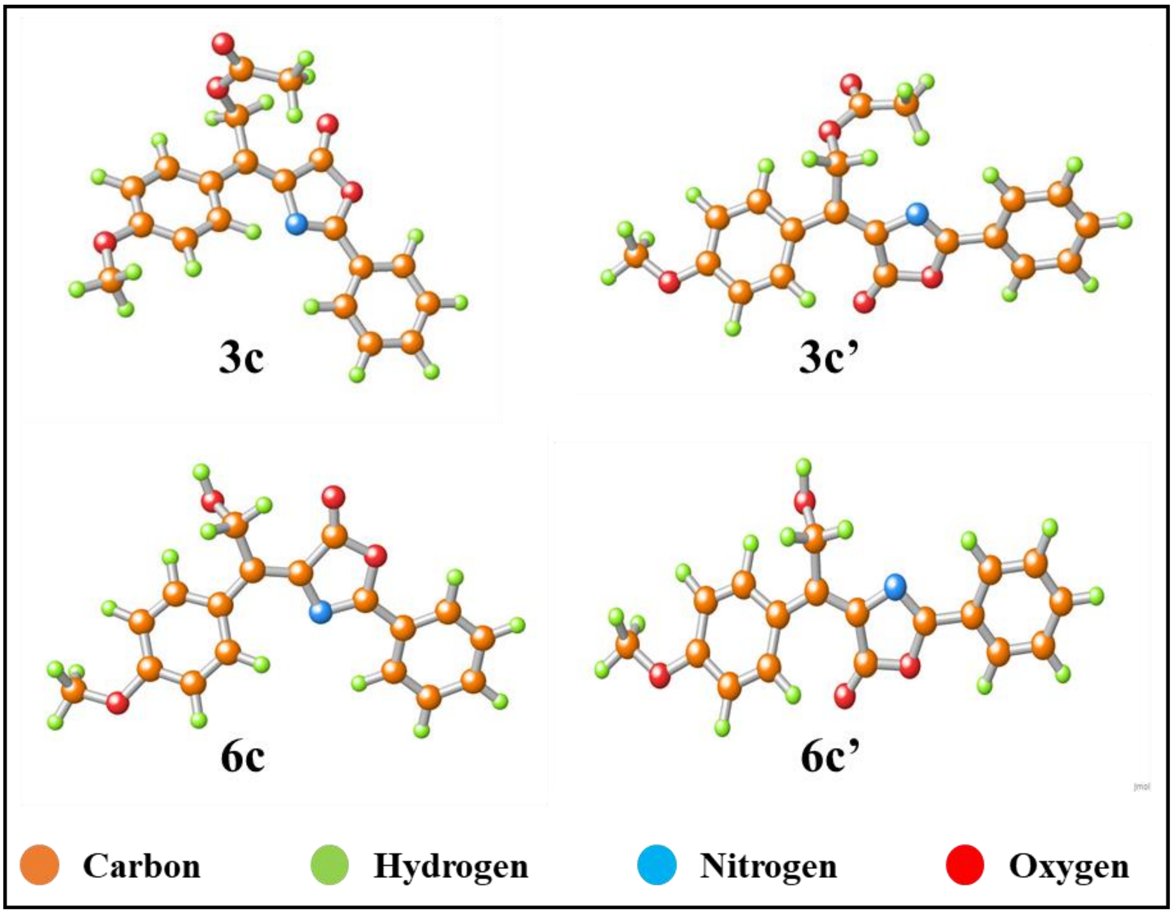

Figure S82: Molecular Geometries **of 3c, 3c', 6c,** and **6c'** Moieties.

Our calculations revealed that the isomerization barrier from **3c** to **3c'** was approximately 5 kcal/mol lower compared to the barrier from **3c'** to **3c**, as shown by the potential energy surface in Figure S83. This result strongly supports the observed fast isomerization process from the trans to cis reactant. Furthermore, we observed comparable barriers for the **6c** to **6c'** and **6c'** to **6c** isomerizations, suggesting that both products are present at equilibrium.

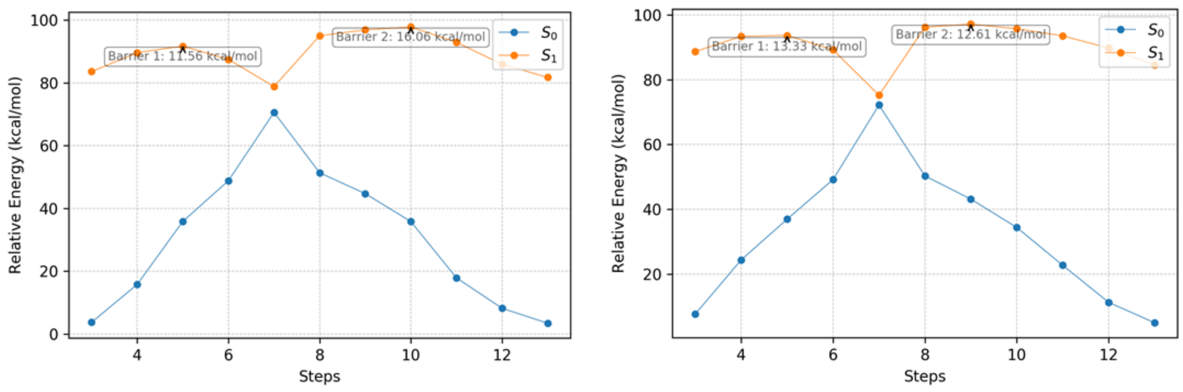

Figure S83: Potential Energy Surfaces for Isomerizations **3c** to **3c'** (left) and **6c** to **6c'** (right).

Scheme S4 provides a comprehensive depiction of the photoisomerization and photodissociation process, encompassing all the molecular transformations involved. This scheme visually represents the key steps and interactions, facilitating a clear understanding of the underlying mechanisms. Moreover, it highlights the energy barrier heights with the values mentioned in parenthesis associated with each step, offering valuable insights into the energetics of the process.

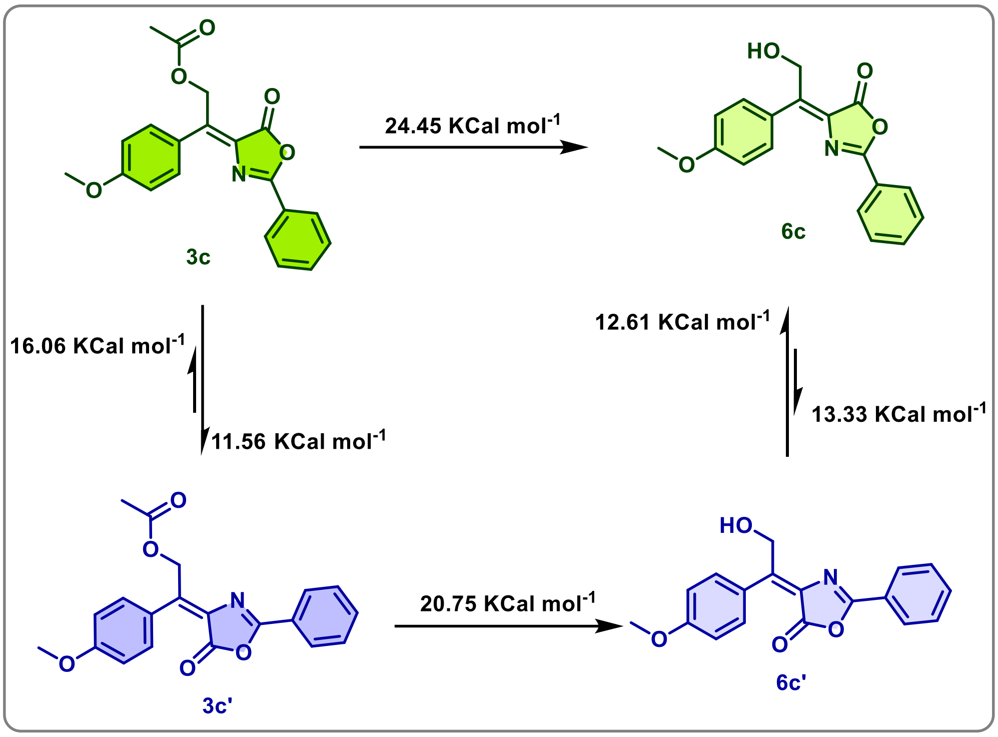

Scheme S4: Energy barriers and transitions in the photoisomerization and photodissociation pathways involving **3c-3c'**(11.56 kcal/mol), **3c'-3c**(16.06 kcal/mol), **6c-6c'**(13.33 kcal/mol), **6c'-6c** (12.61 kcal/mol), **3c-6c**(24.45 kcal/mol), and 3c'-6c'(20.75 kcal/mol). The values in parentheses represent the respective energy barriers for each transition. This figure illustrates the energetic landscape of the key transformations, providing insights into the underlying processes.

Photodissociation via the triplet state is supported by compelling experimental evidence, revealing substantial spin density and non-zero spin-orbit coupling (SOC) as key factors driving favorable intersystem crossing (ISC) dynamics. Figure S53 presents a comprehensive energy level diagram for **3c'**, demonstrating the efficient population of the triplet excited state (T_2) facilitated by a significant reduction in the energy barrier between the singlet excited state (S_1) and T_2, ΔE_{ST}.

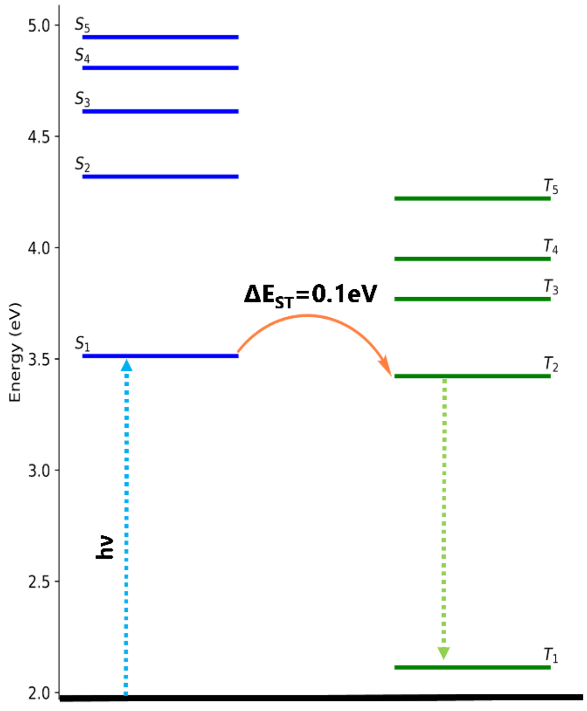

Figure S84: Energy Level Diagram for **3c'**: Facilitating Intersystem Crossing (ISC).

The investigation of spin density provides valuable insights into the role of triplet states and their influence on computational outcomes. The isosurfaces displaying diverse signs of spin density in Figure S54 indicate localized spin distribution in specific regions of the molecular structures. This observation suggests the presence of spin localization, which is indicative of spin-forbidden processes and supports the occurrence of favorable intersystem crossing (ISC) dynamics.

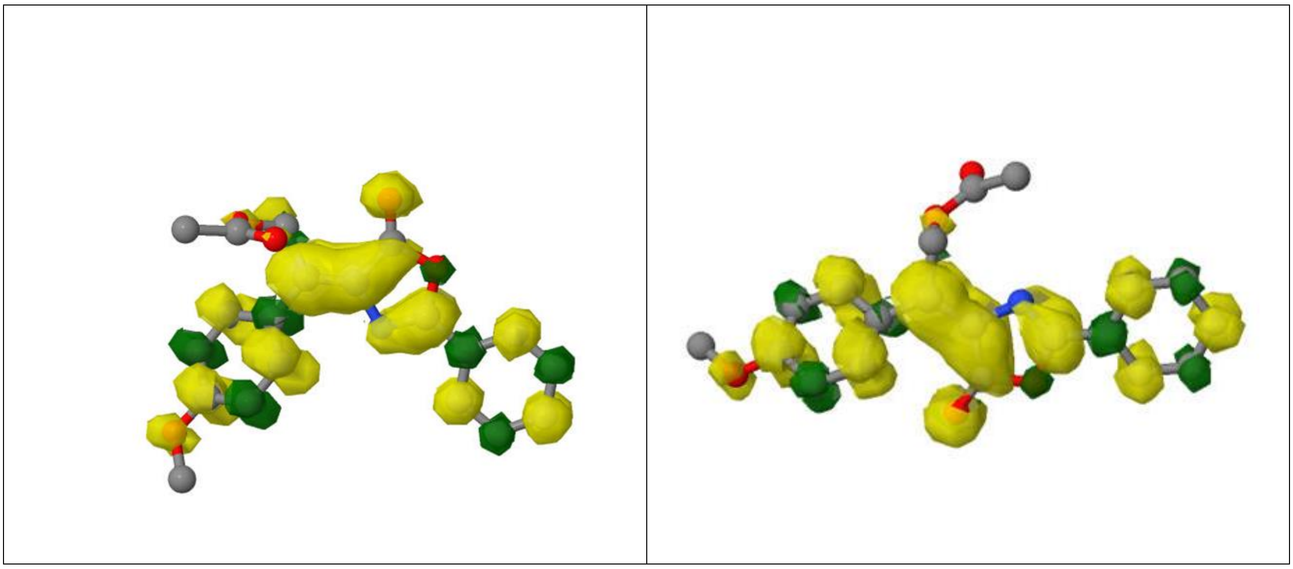

Figure S85: Spin Density Analysis in **3c** (left) and **3c'** (right)

By employing NTO analysis and CDD plots, we can further deepen our understanding of the electronic structure changes accompanying the **3c'** to **3c** isomerization process. NTOs provide insights into the orbitals involved in the electronic transitions, while CDD plots visually depict the redistribution of electron density between different electronic states.

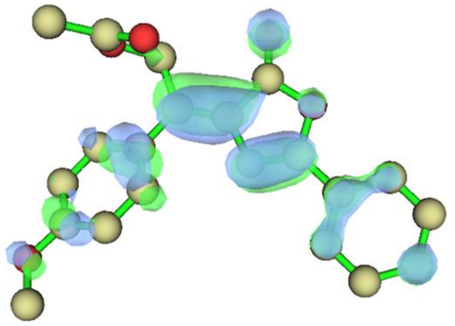

3c HONTO

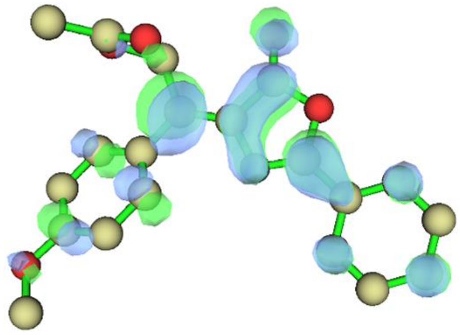

3c LUNTO

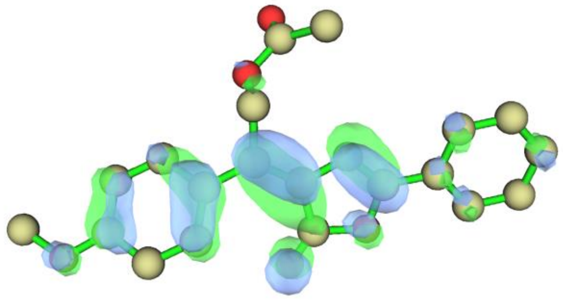

3c' HONTO

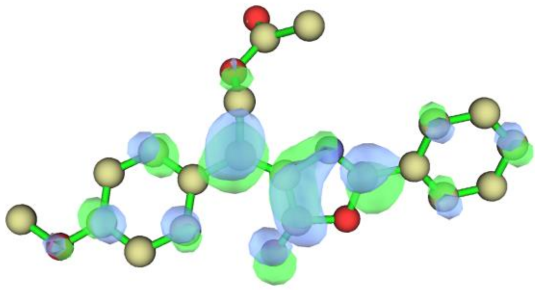

3c' LUNTO

Figure S86: The NTO (Natural Transition Orbitals) analysis conducted in this study reveals that the honto and lunto states are the primary contributors to the S_1 electronic state of the GFP protein. These states are found to exhibit significant electron density delocalization over the aromatic rings, indicating their involvement in π- π * transitions.

The charge density difference (CDD) analysis in Figure S62 reveals an increased electron density at the carbon atom adjacent to the acid-leaving group in the ester. This electron accumulation signifies a higher electron availability in that region, promoting the dissociation of the leaving group.

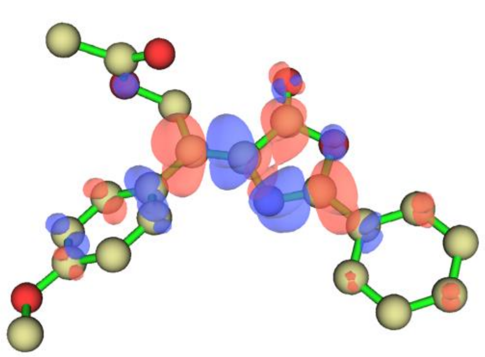

3c

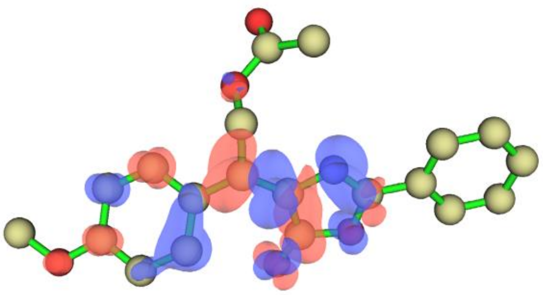

3c'

Figure S87: Charge Density Difference Plots of **3c** (Left) and **3c'** (Right) Isomers. In these plots, the colors red and blue represent an increase and decrease in excited state density compared to the ground state density, respectively.

10. Fluorescence Response in Presence of Bio-molecules:

To study the fluorescence response of GFP-photocage **6** in the presence of various bio-molecules, we have prepared stock solutions for human serum albumin (HSA), bovine serum albumin (BSA), DNA, and streptavidin in the following manner: (i) 50 µM for HAS (ii) 3.2 mg of BSA in 1 ml HEPES buffer (iii) 1 mg of DNA in 1 ml saturated NaCl solution and (iv) 1 µg of streptavidin in 1 ml PBS buffer. Further, we have explored the fluorescence response of photocage **6** for HSA, BSA, and DNA and photocage **8** (Biotin-tag) for streptavidin.

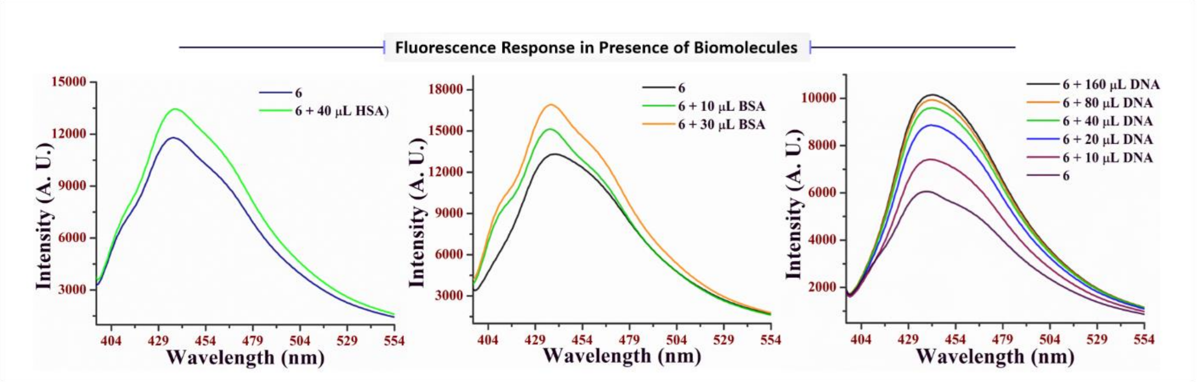

Figure S88: Fluorescence response of GFP-photocage **6**, in the presence of various bio-molecules.

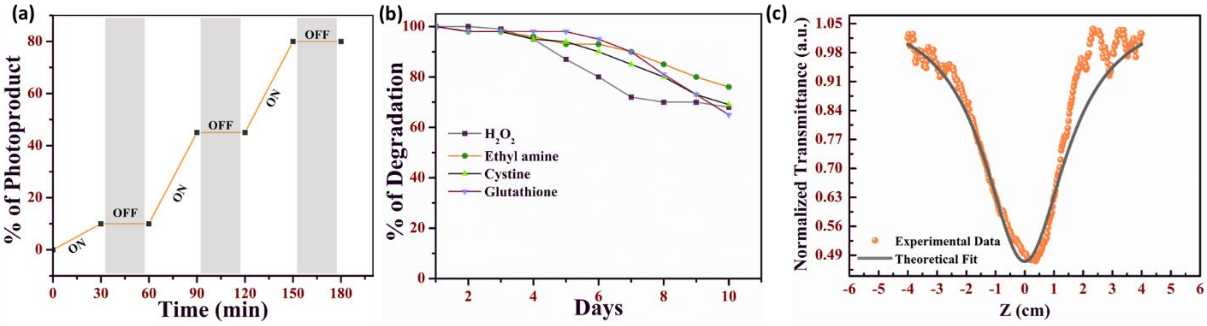

Figure S89: (a)Temporal control over the photorelease step of GFP-photocage **6** by light ON-OFF study (concentration was same as earlier) (b) Stability of the GFP-photocage in the presence of amines, biothiols, and H_2O_2 (c) Z-Scan measurement of photocage **6** in acetonitrile: H_2O 7: 3.

11. Experimental Details of Cell Experiments:

Time-Correlated Single Photon Counting (TCSPC) Assay:

The time-correlated single photon counting (TCSPC) method was utilized to achieve the fluorescence lifetimes (τ) of the synthesized GFP fluorophore along with GFP in the presence of Aβ40 peptide in PBS on the Horiba DeltaFlex lifetime device. The solutions of the GFP probe and GFP in the presence of Aβ40 peptide were distinctly excited with a 510 nm DeltaDiode laser (Horiba Scientific, model: DD510L) to obtain the τ value. Horiba EzTime decay analysis software was used for fluorescence lifetime measurements and data analysis.

Preparation of Aβ40 Monomer, Oligomer and Fibril:

Aβ40 monomers were prepared by dissolving the Aβ40 peptide in hexafluoroisopropanol (HFIP) at a concentration of 250 µM as a stock.

For the preparation of oligomers, 1.0 mg Aβ40 was dissolved in 400 µL HFIP at room temperature and stayed for 15 min. 100 µL of the resulting Aβ40 solution was added to 900 µL D.I. H_2O. After incubation at room temperature for another 15 min, the samples were centrifuged for 15 min, and the supernatant was transferred to a new tube and subjected to a gentle stream of N_2 to evaporate the HFIP. The samples were then stirred at 500 rpm using a stir bar for 24-48 h.

To prepare Aβ40 fibrils, the Aβ40 peptide (1.0 mg) was suspended in 1% ammonia hydroxyl solution (1.0 mL). One hundred microliters of the resulting solution were diluted 10-fold with PBS buffer (pH = 7.4), and kept stirring at room temperature for 5 days. ThT fluorescence experiment and TEM analysis was applied to confirm the formation of Aβ40 fibrils.

Circular Dichroism (CD) Spectroscopy:

CD spectra were acquired on a JASCO instrument (Model J-815–150S) at 25°C. Data were recorded in a 1 mm quartz cuvette at a wavelength range of 190-260 nm with a 0.5 nm step, 2 s collection times per step, and 1 nm bandwidth. The secondary structure of Aβ40 peptide at 10 µM concentration in PBS solution was monitored at the initial stage (0 hours) and after aged for 7 days at 37°C. The average of three spectra was used for each measurement.

Fourier Transform Infrared Spectroscopy (FT-IR):

FT-IR spectra were recorded using a PerkinElmer Spectrum Two FT-IR spectrometer from 4000 to 400 cm^{-1} at room temperature in ATR mode. the Aβ40 peptide fibrils were isolated from solution by centrifugation at 12000 rpm for ten minutes and the secondary structure of the peptides was monitored using FT-IR.

TEM Measurement:

Ten microliters of 5 µM of Aβ40 monomers, oligomers and fibrils in PBS solution were pipetted onto a 300 mesh carbon-coated copper grid and waited for 2 min, the liquid on the grid was carefully dried with a corner of filter paper. for negative staining 10 µL aqueous solution of uranyl acetate [2 % (w/v)] was added to the grid and allowed to float for 1 min. The excess solution was removed using a blotting paper. The sample was dried at room temperature and kept in desiccators before taking TEM analysis on JEOL (Model: JEM 2100F) instrument at 120 KV.

In Vitro Binding Assays:

To study the fluorescence response of GFP towards Aβ40 fibril, a solution of GFP (1 μM) was mixed with different concentrations (1-12 μM) of Aβ40 fibril (a final volume of 2 mL) and incubated in PBS solution at room temperature for different times (0-60 min). Emission spectra (500-800 nm) of the mixture were measured using excitation wavelengths of 490 nm for GFP.

Thioflavin T (ThT) Kinetic Assay:

A stock solution of ThT (50 μM) in PBS (pH 7.4) was made freshly with proper precaution to evade degradation from light. A solution of Aβ40 (10 μM) in 20 mmol of PBS (pH 7.4) was prepared. Aliquots of the Aβ40 peptide solution and the concentrated solution of ThT were mixed in a solution of 20 mmol PBS (pH 7.4). λ_{em} of ThT was monitored at 485 nm with λ_{ex} at 440 nm. The data were collected at 5 min intervals for 200 min. Three independent tests were executed for each ThT assay. Kinetic curves were fitted by the sigmoidal curve fitting. Error bars indicate standard deviations from the mean of three independent runs. All samples were stirred constantly by a micromagnetic stir bar. The incubation conditions were kept the same for GFP and ThT. The ex/em slit widths were retained at 5 nm.

Cell Culture:

SH-SY5Y was a three-time subcloned cell derived from the SK-N-SH human neuroblastoma cell line. The SH-SY5Y cell line was studied as a model for neurodegenerative disorders. A human neuroblastoma cell line SH-SY5Y was maintained in growth media encompassing DMEM:Ham′s F12 (1:1, pH 7.4), glutamine (2 mM), non-essential amino acids (1% NEAA), and 10% FBS. The SH-SY5Y cell line was preserved at 37 °C in the incubator with routine passage of 5% CO_2.

In Vitro Fluorescence Imaging of Aβ40 Fibrils using Synthesized GFP Dye:

The SH-SY5Y cells were seeded and cultured in a dish at 5 × 10^3 cells/dish for 24 h at 37 ℃. Next, washing through 1 × PBS, live SH-SY5Y cells were incubated with Aβ40 (10 μM) at different times at 37 °C in a 5% CO_2 atmosphere. Subsequently, the media was discarded cautiously and washed caringly using 1 × PBS. Successively, SH-SY5Y cells were incubated first with 500 nM GFP for 20 min and then stained with ThT (5 μM) for 20 min at 37 °C in a 5% CO_2 setting. Later in the treatment process, SH-SY5Y cells were finally washed twice with media before capturing the CLSM images. Confocal images of human neuroblastoma SH-SY5Y cells treated with Aβ40 (10 μM) over 0-48 h were obtained by a Leica STELLARIS 5 confocal microscope, and the images were processed through LAS X software. For ThT: laser λ_{ex} = 405 nm (blue channel, detection range of emission 450-500 nm); GFP :laser λ_{ex} = 488 nm (green channel, detection range of emission 570-670 nm).

Pearson's Correlation Coefficient (PCC) Determination:

Pearson's correlation coefficient (PCC) is one of the classical statistical analyses in pattern recognition for matching one confocal image (green channel) with another (red channel). PC graphs can explain the extent of overlapping among two patterns in a dual-color colocalization image. PC coefficients were calculated by the examination of confocal micrographs by the LAS X software with the Quantify tool. To determine PC coefficients for CLSM images, all of the pixels having the same image coordinates were paired.

PCC was calculated (image comprising red and green channels) by the following equation:

$$PCC = \frac{\sum_i (S1_i - S1_{avg}) * (S2_i - S2_{avg})}{[\sum_i (S1_i - S1_{avg})^2 * \sum_i (S2_i - S2_{avg})^2]^{(1/2)}}$$

S1 = signal intensity of pixels (pixel i) in the green channel; **S2** = signal intensity of pixels (pixel i) in the red channel; **S1avg** = mean values of pixels in the green channel; **S2avg** = mean values of pixels in the red channel.

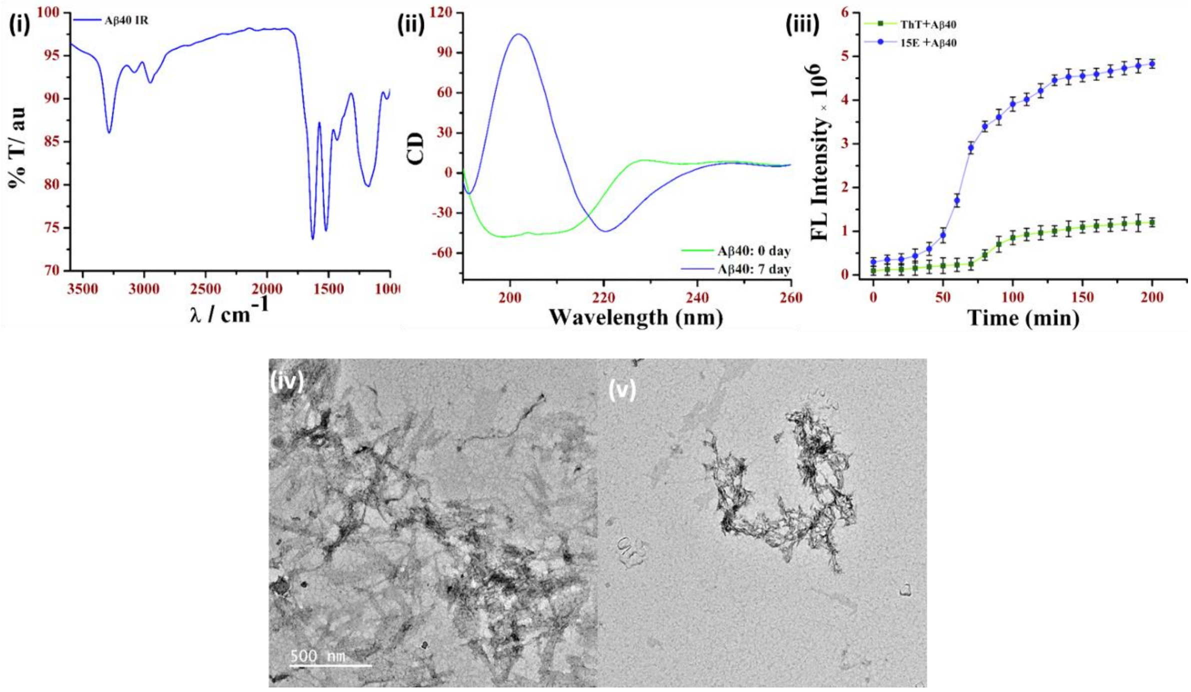

Figure S90: Characterization of Aβ40: (i)) FT-IR spectra of Aβ40 fibril; (ii)) CD spectra of Aβ40 monomers and fibrils; (iii) Monitoring of Aβ40 protein aggregation kinetics using photocage 15E (blue), and the gold standard probe ThT (green); TEM images of Aβ40 peptide (iv) oligomers, and (v) fibrils.

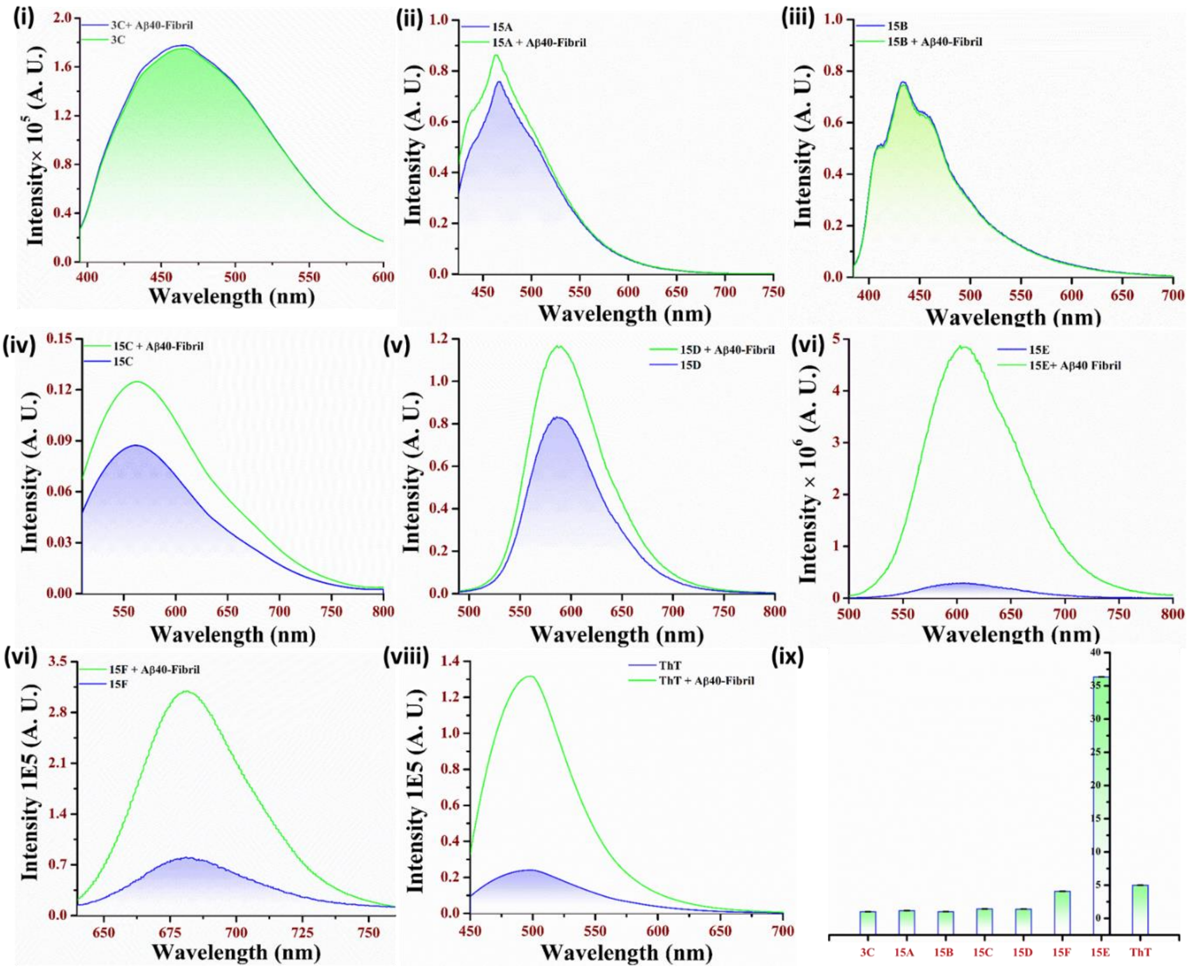

Figure S91: Fluorescence response of different photocages to the Aβ40 fibrils (i) 3C, (ii) 15A, (iii) 15B, (iv) 15C, (v) 15D, (vi) 15E, (vii) 15F; (viii) Fluorescence response of gold standard probe ThT to Aβ40 fibrils; (ix) comparative fluorescence response of the photocages.

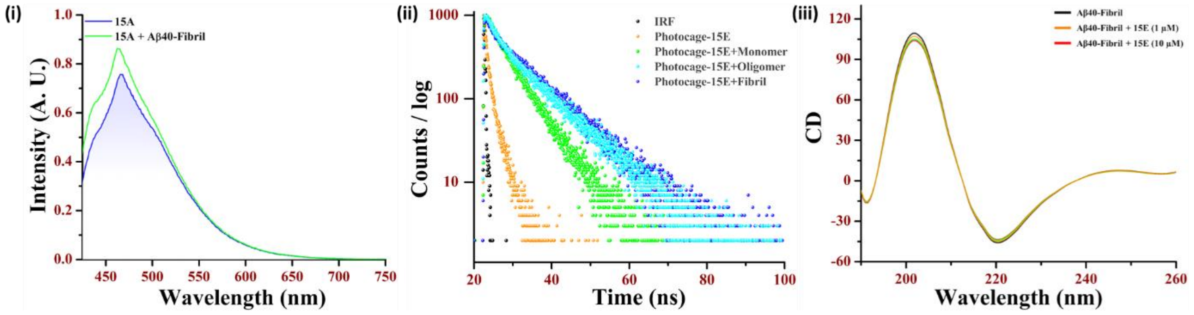

Figure S92: . (i) Change in the emission spectra of 15A in the presence of Aβ40 fibrils; (ii) Change in the singlet state lifetime of 15E in the presence of Aβ40 monomer, oligomer, and fibril; (iii) Change in the CD spectra of Aβ40 with increment in the concentration of 15E (0 μM - 10 μM).

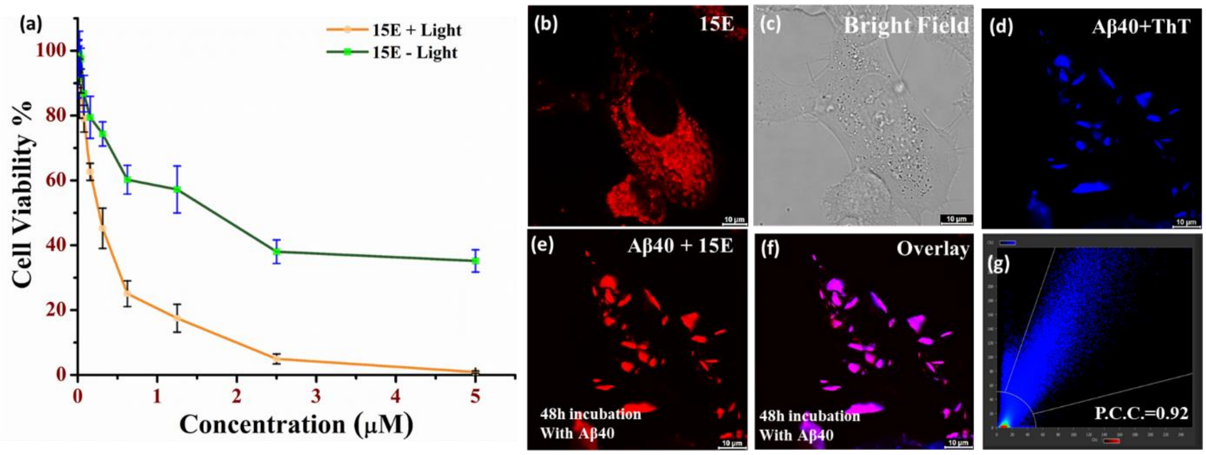

Figure S93: (a) Cell viability of SH-SY5Y cells at different doses of photocage 15E + over 48 h exhibits negligible cytotoxicity; CLSM images of SH-SY5Y cells incubated with (b), (c) 15E, Aβ40 (10 μM) for 48 h, stained with (d) Thioflavin-T (ThT) (blue), (e) 15E (red), (f) colocalized image, and (g) Pearson Correlation Coefficient (P. C. C.).

12. Co-ordinates used in DFT and TDDFT calculations:

Cartesian coordinates

Cartesian coordinates

1. **3c'**

i)S_0

NImag=0

43

Coordinates from ORCA-job opt

C	-4.64469291992999	-0.03477260121775	-0.33023567036142
C	-3.25707935674274	0.02949246337607	-0.06204026928243
C	-2.56601215354976	1.22701391906592	-0.13351475031887
C	-3.23689303853882	2.42017351099389	-0.46139586584211
C	-4.61305959047019	2.37947786445764	-0.74024744397503
C	-5.29514533105760	1.16588964681909	-0.68717195024207
H	-2.70910816203939	-0.87739948790369	0.18476323735309
H	-1.49305822391999	1.27266589997065	0.06127024370937
H	-5.16301147301798	3.28436024846382	-0.99719694164806
H	-6.36576305949027	1.15352201939119	-0.89284066083729
O	-2.48414666346454	3.53990169442589	-0.49236934667100
C	-3.09467762062749	4.77365438582399	-0.80687255298113
H	-2.30314597976714	5.53335785928252	-0.76402849020637
H	-3.53214829272269	4.76516056951022	-1.82112020662353

H	-3.88426764926343	5.03398294157633	-0.07985635678410
C	-6.51196405958349	-1.45823883183579	-1.32934093666209
H	-6.75499609077546	-2.51880043042774	-1.44733648362459
H	-6.18845917467166	-1.05186235440114	-2.29855130834199
O	-7.65795400137483	-0.68893050916934	-0.94635412598333
C	-8.80475308894877	-1.21383130300216	-0.42532550420190
O	-9.63112409956083	-0.45127202617513	-0.00583820619223
C	-8.97429334357029	-2.71602109934675	-0.45011225956669
H	-8.95980504702187	-3.09051583898759	-1.48684559764505
H	-8.16361636272667	-3.22439441620148	0.09272619847032
H	-9.94393391564467	-2.95059459141728	0.00409879073588
C	-5.39072694028787	-1.29682622452557	-0.31344251500102
C	-5.49477167720166	-4.27136742472238	1.47836945772169
C	-5.16758407188112	-2.34933303019252	0.53894406976312
N	-5.86090893475523	-3.56224360465177	0.46237153553051
O	-4.58603428899800	-3.67132615299515	2.30503647962264
C	-4.30353377513029	-2.40965966278650	1.75696007551854
O	-3.53690503968927	-1.66157027155004	2.28693341096845
C	-5.94809697033825	-5.61234330773938	1.82796324510853
C	-6.84232540203062	-6.28778490892358	0.97614685934128
C	-5.50604455896539	-6.23275462773864	3.00999158479665
C	-7.28947852562343	-7.56372122805345	1.30833808640805
H	-7.17067748370332	-5.79614762289861	0.05897782125867
C	-5.95914539296864	-7.51141977573946	3.33528772312274
H	-4.81285969573205	-5.70278050681954	3.66476453983481
C	-6.85010296467906	-8.17786756606196	2.48823015995124
H	-7.98358349717451	-8.08619015729032	0.64577363889342
H	-5.61615015777005	-7.99042527168394	4.25535319041963
H	-7.20395143458993	-9.17913270869830	2.74629037446363

ii) S_1 minimum

NImag=0

43

Coordinates from ORCA-job opt

C	-4.76382844360078	-0.10431558935817	-0.44928551380983
C	-3.41181041175511	-0.10819306688707	0.00976978599996
C	-2.66930785613803	1.04061405740775	0.04676508133027
C	-3.22379332202798	2.24803697259416	-0.36924167199975
C	-4.53919898387132	2.27807240166851	-0.86682650716327
C	-5.28073593884448	1.12804659969756	-0.91251612002487
H	-2.94574604808156	-1.04237150260356	0.27515842593824
H	-1.64536263700069	1.03367903061162	0.39276344109994
H	-4.98032717060896	3.20510970590486	-1.19927895763611
H	-6.30327853900268	1.17298041825156	-1.25748768408668
O	-2.43704701176998	3.32718819955455	-0.27817553426510
C	-2.93358421659065	4.59032499732364	-0.68123337480974
H	-2.12784138891197	5.29632491838379	-0.50536516969232
H	-3.19334298819290	4.59129187982137	-1.74169211613755
H	-3.80468998071139	4.87678339536548	-0.08898667915373
C	-6.69019363524899	-1.41513030768947	-1.44323533985965
H	-6.97925647918877	-2.45801556424230	-1.53359328614145
H	-6.39322824620886	-1.04621088752977	-2.42364550249135
O	-7.81726785092933	-0.59114760456540	-1.08180826716466
C	-8.90273873144412	-1.05298291083221	-0.42418241962433
O	-9.71393541770860	-0.25817476395180	-0.04896046919149
C	-9.05455707926922	-2.53693298411503	-0.23995260535013
H	-9.13487607285518	-3.03271243012922	-1.20831367526208
H	-8.20565962166048	-2.97605209543574	0.27999735922383
H	-9.96522134774689	-2.70710594918599	0.32437952962621
C	-5.55959039056125	-1.29076748549123	-0.46987586969325
C	-5.48738348871236	-4.24281177523806	1.41307293445842
C	-5.34798162989361	-2.33467018484502	0.43675229202572
N	-5.82301400317632	-3.60610562045245	0.32106423701732
O	-4.75960030312400	-3.49775215241568	2.27563672468499
C	-4.67854085784326	-2.22151698659656	1.72622335766952
O	-4.12936588113497	-1.31355140660212	2.29881352160010

C	-5.83761346298529	-5.58337674412781	1.77665749791862
C	-6.60450701747994	-6.35914696740300	0.89776349689769
C	-5.43757006860158	-6.12647084927965	3.00297680094605
C	-6.96227563189348	-7.64337364143677	1.24481400269979
H	-6.90296111968758	-5.93309142424583	-0.04942389633056
C	-5.80190006762016	-7.41324327040400	3.33785432041922
H	-4.84724057847253	-5.52769550486508	3.68149345971769
C	-6.56453248870611	-8.17597966533742	2.46415659482216
H	-7.55500087254771	-8.23829916126179	0.56308859738094
H	-5.49209972422930	-7.82741155914473	4.28777423139386
H	-6.84798250396065	-9.18471704091159	2.73265824701731

iii) TS in S_1

NImag=1

43

Coordinates from ORCA-job scan

C	-4.73320719152269	-0.20654226802813	-0.54099272941719
C	-3.48834191600090	-0.10279371139791	0.11113341612001
C	-2.80844213288289	1.09516598928330	0.15930692759746
C	-3.36097261856368	2.24730195328344	-0.42180940525825
C	-4.60398188018401	2.16899572888021	-1.06019922136408
C	-5.27146764783469	0.95353936337708	-1.12002644352085
H	-3.03748526932735	-0.98266702373285	0.56988631670694
H	-1.83890421416480	1.18044046738843	0.65100851008369
H	-5.06744112621577	3.05047461653033	-1.50024993120144
H	-6.25991367898647	0.90493310108292	-1.57910179835813
O	-2.63562794095103	3.37209972096518	-0.31180494455602
C	-3.12863178157302	4.56822661386588	-0.86111494805489
H	-2.37850060148486	5.34083962118970	-0.65441688085281
H	-3.26925292879129	4.48693461689804	-1.95206230237181
H	-4.08521470642930	4.86368759561950	-0.39867009948747
C	-6.26013604018166	-1.75560177137588	-1.72380360406445
H	-6.75686914682811	-2.72186608331335	-1.78939682085083

H	-6.22441928116497	-1.14028905657547	-2.61754740640078
O	-8.04734602367397	-0.60401957087254	-1.35265327563425
C	-8.86868752308226	-0.69209512507291	-0.34894016472810
O	-9.81578538784965	0.07012934052835	-0.31556361725181
C	-8.60508027413681	-1.70968702674507	0.73361545664076
H	-8.29860163309586	-2.67849927633761	0.31812595108149
H	-7.79573520550276	-1.34112417041226	1.38159558078592
H	-9.51212082496346	-1.82484418165267	1.33871418160120
C	-5.45086473089933	-1.47600712229069	-0.64988044747190
C	-5.63850273082629	-4.35512863306959	1.39875584395301
C	-5.37903368077026	-2.47829016169671	0.41316715420380
N	-5.71104591676923	-3.76386789433442	0.22161756647036
O	-5.27923113737819	-3.54096313185289	2.39814788040916
C	-5.10531269164231	-2.26118670290470	1.83633548856993
O	-4.82429417907659	-1.31354159759239	2.50719115636252
C	-5.90211127627886	-5.74870034457456	1.67637292594549
C	-6.27999220265704	-6.60022749223543	0.62661664628204
C	-5.78372649919790	-6.25131332930011	2.98064179186997
C	-6.53628731068897	-7.93851375144814	0.88391211077069
H	-6.36494932197203	-6.18918931801177	-0.37984509008774
C	-6.04132221747040	-7.59258360343831	3.22714344206424
H	-5.48980489104853	-5.57994814918201	3.78817006999657
C	-6.41742844696782	-8.43605868126089	2.18218591578954
H	-6.83093075345334	-8.60201636299320	0.06894001450192
H	-5.94929438079259	-7.98554085773242	4.24118640472324
H	-6.61969016671461	-9.49053684945640	2.38086165840263

iv) T_1 minimum

NImag=0

43

Coordinates from ORCA-job opt

C	-4.72761425185300	0.00838151993847	-0.28386861394220
C	-3.36923708076001	0.02372714268066	0.12659080011319

C	-2.59724200785127	1.14184146760515	-0.00038980218071
C	-3.13388225620606	2.32035424104063	-0.52675191804249
C	-4.46384917836936	2.34211083230121	-0.94406825056704
C	-5.23350411106851	1.20931695372441	-0.83152968612002
H	-2.92524028812799	-0.86767884842900	0.53754544533921
H	-1.56184802961680	1.14290120265114	0.31001652718091
H	-4.90631203174558	3.24238268180452	-1.34158947555699
H	-6.27290757457039	1.26310702607739	-1.12030462070204
O	-2.30014817441154	3.37410415093465	-0.58908291793194
C	-2.79175644649547	4.60307072522867	-1.08367201808761
H	-1.96219028152859	5.30166868152786	-1.03098445383303
H	-3.12135672522304	4.50867519423525	-2.12055615313751
H	-3.61680517614780	4.97273507598659	-0.47120158450340
C	-6.80744651759071	-1.24335867096678	-0.97509703915900
H	-6.98076306403042	-2.28484139897631	-1.22161839599709
H	-6.73071735719137	-0.67614578246110	-1.90031579805394
O	-7.92418507087429	-0.70005994973886	-0.24475261114364
C	-9.08105743697193	-1.36603467115910	-0.05408610624745
O	-9.96887521057642	-0.81006288988034	0.52302918907973
C	-9.19818855364354	-2.77580566425740	-0.57048226274232
H	-9.10560236061580	-2.80575896225637	-1.65635872525700
H	-8.42317478279125	-3.41579525613439	-0.15001233593921
H	-10.17702165870719	-3.14643165205343	-0.28564022792055
C	-5.54865282784120	-1.13545901301574	-0.18128349242660
C	-5.45551034965922	-4.21094776209749	1.56126080194504
C	-5.27877311889423	-2.25222426362963	0.71235479995840
N	-5.78448346956541	-3.47412163177277	0.51095489684126
O	-4.73335947617831	-3.55229754141764	2.47213315772802
C	-4.59354792719771	-2.23195856803750	1.99808719508425
O	-4.01498671053157	-1.40158936789930	2.63569322637325
C	-5.82742452286298	-5.57762585897023	1.79973506716270
C	-6.54313291380379	-6.28016913167334	0.82667146846383
C	-5.49432962358666	-6.20575452273620	3.00200957960391

C	-6.91852434682125	-7.58634919372104	1.05794035464040
H	-6.79081138440197	-5.78562656114539	-0.10149824981649
C	-5.87712354419097	-7.51272279718128	3.22379353292549
H	-4.94337449734568	-5.65801486937759	3.75296460344678
C	-6.58884300817654	-8.20535246019877	2.25562835129187
H	-7.47201462906487	-8.12839270486374	0.30338362697431
H	-5.62234174158209	-7.99531089334649	4.15741190120265
H	-6.88782979132615	-9.22936052833896	2.43449349395304

v) TS in T_1

NImag=1

43

Coordinates from ORCA-job opt

C	-4.57641428630070	-0.11319763987992	-0.49810056502255
C	-3.22756483972037	-0.03156576207935	-0.09876386924250
C	-2.56126905972752	1.17509540744997	-0.09671434295992
C	-3.22773806108715	2.35450056528384	-0.46560266389187
C	-4.57077599169256	2.29530753143609	-0.85369416826283
C	-5.22669844855425	1.07236273098429	-0.87638153073586
H	-2.68958832271546	-0.93132230134055	0.19717347279985
H	-1.51412753626498	1.24626983867746	0.19910780749415
H	-5.11913152323761	3.19538826144095	-1.12663511346817
H	-6.28572678790087	1.03228923747080	-1.13433591394646
O	-2.50474762891018	3.48515032603121	-0.41211494485696
C	-3.11302554377134	4.70838064417691	-0.74288447662103
H	-2.34713596798492	5.48334829051368	-0.61942310053793
H	-3.46797175312031	4.71699730517406	-1.78718368786789
H	-3.96205078828449	4.93291657258640	-0.07577446348851
C	-6.24025044371635	-1.60396915137828	-1.54656028568768
H	-6.73038572923381	-2.57192900538785	-1.61020522412924
H	-6.30048251114060	-0.93880501884332	-2.40257596049717
O	-7.91308541242703	-0.51154657350029	-0.74992456026766
C	-8.97870058772898	-1.06466062855104	-0.24658453814220

O	-9.88766409371238	-0.34637268556997	0.12054656405703
C	-9.04934183032474	-2.57115810607629	-0.17139019435789
H	-9.13313031592834	-2.98773927478920	-1.18707416979530
H	-8.14696821215684	-2.99844215953171	0.28604381451185
H	-9.93840681424918	-2.85581444147025	0.40396217065386
C	-5.30680632895946	-1.37920382185082	-0.55815056492439
C	-5.45920757771886	-4.27406887826302	1.46592917419065
C	-5.14864881388923	-2.41074892214373	0.46192092768472
N	-5.70497151745154	-3.62944692996859	0.34342620689334
O	-4.75341420129223	-3.57011861996394	2.35675484170941
C	-4.50835030671265	-2.30783928121945	1.77908020074884
O	-3.90152430660969	-1.46391059586063	2.36621580841257
C	-5.88930755057376	-5.61393125133654	1.79885496004562
C	-6.61280873880314	-6.36131676584829	0.85679573566974
C	-5.59635744965713	-6.16329669848488	3.05573051124924
C	-7.03747607131384	-7.64280413412534	1.17383444595374
H	-6.82954135658117	-5.91675994218125	-0.11505027503319
C	-6.02666979745704	-7.44642146067809	3.36346336278417
H	-5.03532317005163	-5.57198436111386	3.78015504651336
C	-6.74624408389428	-8.18619809096530	2.42564458721228
H	-7.60092851311112	-8.22525818452513	0.44276569131475
H	-5.80206721206160	-7.87420484319822	4.34207875493162
H	-7.08395951397033	-9.19484518110041	2.67219852890638

2. **3c**

i)S_0

NImag=0

43

Coordinates from ORCA-job opt

C	-1.46222261507823	1.93942522468015	-2.70821676122102
C	-1.73182162873980	1.25396603185520	-3.87405424715487
C	-1.88039121620213	-0.80943035021629	-2.62859315194593
C	-1.36907597002349	1.27396193788587	-1.46655339227475

H	-1.53422930109618	-0.65523642771535	-0.52408529899905
C	-1.07268207109235	2.02769586835310	-0.24241529917923
C	-1.94560149258481	-0.13084615119991	-3.84934561932172
H	-2.05520461342297	-1.88268570166967	-2.57188220983181
H	-1.79578704436386	1.77049805564548	-4.83226230869770
O	-2.21038346927774	-0.71456135941038	-5.02995132293886
C	-1.59115199124642	-0.11160341318687	-1.46338757892882
H	-1.33030783773874	3.01831756427806	-2.76649677202369
C	-2.44791172311126	-2.09820460737616	-5.07634884431894
H	-2.64066019505465	-2.34909731474352	-6.12629159269346
H	-1.57456501235858	-2.67284193105890	-4.72427141168991
H	-3.32739043722562	-2.38017939616832	-4.47321751953343
N	-0.13295705491437	0.15210729805314	1.05204734490271
C	-0.32198511824012	2.16283486477682	2.19354160343876
C	-1.33404671292543	3.51841814437180	-0.26541167575401
H	-1.21851266605506	3.95226229870775	0.73212607455088
H	-0.57819323634532	3.99202082846664	-0.90978203004315
O	-2.57720283829113	3.87154824459978	-0.84630054305582
C	-3.75923112242398	3.73386277882639	-0.19637608346470
H	-3.46634472098610	2.06914404170493	1.12899511230251
C	-3.73832445898047	3.13322694620628	1.18712734051827
H	-3.01272506103477	3.62264518423416	1.85071180808088
H	-4.74632984665312	3.22138559037701	1.60509318786850
O	-4.75803618831968	4.07999494062711	-0.75405062683011
C	-0.54519607742262	1.47793560427129	0.88943725226398
O	0.19395287734389	1.17680981837928	3.01096004522257
C	0.80884844583197	-1.15523606204596	2.90834449348399
C	0.27469151929525	0.03812758359542	2.25926934245341
O	-0.51488826202850	3.27175897202556	2.59981098707321
C	1.45808818099528	-3.47448339413909	2.76998196609425
C	1.65595207482820	-2.28573065763517	4.86398191103380
C	1.15997369327652	-1.13482847220727	4.26185802564331
C	0.96077283940718	-2.33028970309897	2.16250331236274

H	1.57760842143217	-4.39067511298930	2.18860419222736
H	2.19527025999393	-4.35636691513839	4.59680811510368
H	0.68554176086882	-2.32285016260629	1.10710579383913
C	1.80515460240113	-3.45416259826673	4.12110209548897
H	1.92735167408514	-2.27025459221782	5.92114836412186
H	1.03885363347806	-0.21388349883086	4.83293592182616

ii)S_1 minimum

Nimag=0

43

Coordinates from ORCA-job opt

C	-12.45668966156572	1.02327671566095	-0.10162991222378
C	-13.64055167788707	1.64232999882259	-0.59416264714748
C	-13.90159428097317	2.96269424645134	-0.36161771116403
C	-13.00491590580743	3.74709154529057	0.37148675820745
C	-11.83474837653434	3.16716298020835	0.88064740804613
C	-11.57748690595673	1.84102662300933	0.65312906006316
H	-14.34330783121482	1.04921425900047	-1.15460366176657
H	-14.80567935568001	3.42751226344836	-0.73033375493361
H	-11.12155232217073	3.75717753831573	1.43542547499155
H	-10.64673555839934	1.42631979521351	1.01015147364052
O	-13.34139508419696	5.03061978700899	0.53767678256959
C	-12.48833657868349	5.88402191903194	1.27922645157110
H	-12.97126859275977	6.85622304067316	1.28484446466615
H	-12.37241178464998	5.52739338569022	2.30448576731867
H	-11.50829623776003	5.96574324795819	0.80528880028209
C	-11.09768640064606	-0.99904278477639	0.52301043538937
H	-11.22850666798272	-2.07423525349021	0.50308426117567
H	-11.16926789572208	-0.64691007157367	1.55339930006006
O	-9.78945435201815	-0.63911738513274	0.02966933570785
C	-8.72888895904549	-1.47346869400707	0.10541942801104
O	-7.66775534725392	-1.07348654789051	-0.27545853127371
C	-8.94672310601401	-2.85559805760024	0.65382363433325

H	-9.34914510697237	-2.82046771095537	1.66643108944164
H	-9.65298464123340	-3.41018683914602	0.03541887339054
H	-7.98780438972851	-3.36282574707903	0.66294148866590
C	-12.13519469171958	-0.35032194385158	-0.33317028374840
C	-12.71475739173445	-1.10386967694283	-1.36346295080389
N	-13.49988945602252	-0.63147953657841	-2.36064011046024
O	-13.23110011615609	-2.81145088421875	-2.75766813128956
C	-12.51881051925060	-2.54789799589216	-1.59954606375637
O	-11.90623333564351	-3.41204125790548	-1.03374701284560
C	-13.80961708922656	-1.64628506473410	-3.13833169176865
C	-14.67734148409507	-1.62495445268984	-4.26244854354338
C	-14.94963754437435	-2.78924882172704	-4.99983218972015
C	-15.27542089050255	-0.41134580387466	-4.64825055583803
C	-15.79549503831190	-2.73383439135399	-6.08372477023264
H	-14.48922036290583	-3.72180950735762	-4.70684403695161
C	-16.12074272226936	-0.37442853731275	-5.73359194902894
H	-15.04984706500115	0.48217485235124	-4.08373579161248
C	-16.38582835678274	-1.53078046217708	-6.45681605195946
H	-16.00092232442460	-3.63287013608569	-6.64900612092086
H	-16.57642835553979	0.56188657010524	-6.02687161332738
H	-17.04806061518224	-1.49547017388696	-7.31132274121480

iii)TS in S_1

Nimag=1

43

Coordinates from ORCA-job scan

C	-12.64725456776703	0.97225005107587	0.16677046147719
C	-13.88166794478122	1.54573359043128	-0.20291637451061
C	-14.10337010022596	2.89980174538141	-0.06607373585689
C	-13.09021042349801	3.74126509394662	0.42008715258817
C	-11.85253400675458	3.19415350728157	0.77385581620021
C	-11.64487876050502	1.82637632800584	0.65347647596901

H	-14.67760770493063	0.91188295963834	-0.58888169455339
H	-15.06110326911088	3.34912634987283	-0.33168759996637
H	-11.03956975703366	3.82416063770019	1.13112381084115
H	-10.66346981167190	1.41018850844027	0.88311456222169
O	-13.39463671735805	5.04724784964637	0.50562172254637
C	-12.42772822849942	5.95092706421923	0.97788519461637
H	-12.89269314544904	6.94397402279147	0.96392146314283
H	-12.11895757654546	5.71353738282663	2.00977086678282
H	-11.53371991160944	5.96386241636686	0.33218109290797
C	-11.54984435782348	-1.08886436778192	0.97759064479221
H	-11.40998118089343	-2.16613850869179	0.93676359070322
H	-11.25546262683540	-0.56932116723462	1.88539843194678
O	-9.68025213480268	-0.53879381894817	0.13327295837743
C	-8.73716168406291	-1.37464661136121	-0.20070229336609
O	-7.69775832952077	-0.91330436767036	-0.63007604249598
C	-8.96150316287475	-2.85246916236609	-0.01134881681931
H	-9.04164881791378	-3.08024334589450	1.06242546635202
H	-9.89247051280025	-3.18842746213439	-0.48732874190023
H	-8.10682839958746	-3.39665939410317	-0.43007501563547
C	-12.38373624157809	-0.46490714645493	0.07466567223376
C	-12.91315395638442	-1.23237160187098	-1.04911315062204
N	-13.58791138066864	-0.71411204156508	-2.08440732358710
O	-13.42055071389022	-2.90506552297121	-2.46972252504999
C	-12.77124566572394	-2.67700848234665	-1.25362661729851
O	-12.24938017807118	-3.56607233377117	-0.63889757045561
C	-13.87067823025645	-1.71463226896854	-2.89574168735958
C	-14.58756128114494	-1.63069158040373	-4.14608532790251
C	-14.84027484783939	-2.78291341237813	-4.90563836123144
C	-15.02796500999325	-0.37791669520601	-4.60153720187501
C	-15.52855282235052	-2.67836073821310	-6.10618020489012
H	-14.49121925765122	-3.75041400634988	-4.54306184798340
C	-15.71432022230321	-0.28496818213598	-5.80276473315566
H	-14.81525280257872	0.50539306321515	-3.99822429626844

C	-15.96586079589084	-1.43272841468237	-6.55558138270754
H	-15.72595814852846	-3.57356451540706	-6.69856104233011
H	-16.05570544416339	0.68839015785200	-6.15963239053395
H	-16.50609424812149	-1.35523454978086	-7.50131594534310

iv) T_1 minimum

Nimag=0

43

Coordinates from ORCA-job opt

C	-1.67097138817358	1.92652765401699	-2.65416244950391
C	-1.94966776573318	1.22322201900996	-3.79862620254756
C	-1.87786283653477	-0.85498188105703	-2.55873940510510
C	-1.45725806926027	1.26991000506111	-1.40088653423969
H	-1.44083447749250	-0.69506758685312	-0.48105669036212
C	-1.13531536370029	2.01734149061741	-0.23261084647512
C	-2.05407765809782	-0.18036296362743	-3.77369202718865
H	-1.96610992062192	-1.93899142597533	-2.50292928902756
H	-2.09999201574185	1.73270347361950	-4.75127029155313
O	-2.32390496018700	-0.77739231750614	-4.94452497529067
C	-1.58557738792711	-0.14664836699298	-1.40703414228644
H	-1.62408219279086	3.01178862016517	-2.70908588806921
C	-2.44016121876720	-2.17667322118717	-4.99645797537958
H	-2.65251080655773	-2.43573209151588	-6.04050676516149
H	-1.50539713486650	-2.67274976011808	-4.68576005391169
H	-3.26560600789613	-2.53913606073985	-4.36129337074527
N	-0.09744454009637	0.18119855990334	1.09408796416420
C	-0.65647603402629	2.00446272513644	2.33882395837066

C	-1.16665112045910	3.52550412157474	-0.25316595845688
H	-1.04508585955892	3.92468092947219	0.76017207775305
H	-0.32451316816117	3.90478496881000	-0.85439138216145
O	-2.30825387563077	4.10287395332529	-0.87705077632899
C	-3.55690425252251	4.05994561088679	-0.35381544268960
H	-3.59674551927944	2.25543703983859	0.80606849873661
C	-3.75596836594325	3.33419728401979	0.95093257521287
H	-3.05559834839597	3.66538743689961	1.72870793091490
H	-4.78862326382365	3.50406224910508	1.27313895865099
O	-4.44176911015178	4.59124190445304	-0.95893513467891
C	-0.65677793866194	1.39237230061772	1.00077689569774
O	-0.06624904385073	1.04017950357313	3.15220984348821
C	0.82487527391706	-1.18539690262144	2.93980959420219
C	0.22930647696633	-0.00633521258995	2.36873762063113
O	-1.02418435666246	3.06138976097569	2.78306973054432
C	1.72214459745741	-3.41039267207616	2.64985594286676
C	1.65174841533648	-2.41074837817665	4.85123213742649
C	1.08119579881823	-1.26375451513190	4.31921002728732
C	1.15196180879065	-2.26954434342061	2.10656087762187
H	1.97686699547028	-4.25114372238534	2.00180123053068
H	2.42194536203145	-4.38571046970105	4.44459323990428
H	0.95242781924039	-2.18970660781272	1.03734108685860
C	1.97276589213871	-3.48514605423641	4.02097763739609
H	1.84879373663520	-2.47080681303130	5.92324083499429
H	0.82524182477095	-0.41829024432504	4.95884693790973

v) TS in T_1

Nimag=1

43

Coordinates from ORCA-job opt

C	-2.37487978463391	1.61965630250233	-2.56977822495005
C	-2.71516015314986	0.83611735511756	-3.65210258256670
C	-0.90762431235428	-0.68505135261974	-3.12630745820185

C	-1.29434363358726	1.27233681461104	-1.72998843781998
H	0.25322022170201	-0.19828283120435	-1.39931909624571
C	-0.93884194428274	2.14467882686427	-0.60975105171636
C	-1.98193070163674	-0.32316299487921	-3.94743655573625
H	-0.31882671614974	-1.57743318091862	-3.33281253272548
H	-3.56309352660782	1.08738941258207	-4.29034543762143
O	-2.38180370500318	-1.02179034324487	-5.02310297597765
C	-0.58240867226118	0.10093413402558	-2.02912703447520
H	-2.98071297950071	2.49169204501232	-2.31989903880254
C	-1.70721442372844	-2.20427684442237	-5.36872497459523
H	-2.20600274567165	-2.60138098883238	-6.26090573055632
H	-0.64687000867762	-2.01353964578000	-5.60577707123343
H	-1.76351554882072	-2.95485976666392	-4.56226295789245
N	-0.34469589302094	0.27889555515833	0.92618344989682
C	0.22828235554652	2.34714202195522	1.70983551933752
C	-1.18354420932156	3.50262207489009	-0.66673014420504
H	-0.85914034092373	4.15518951117132	0.14030846327441
H	-1.43046911506112	3.96594646136878	-1.61656166296912
O	-3.26622323382129	3.67719044391655	-0.30763300397383
C	-3.87914364544186	3.52250684056989	0.83199330485050
H	-2.74641517199347	2.08929630208289	1.95157671702202
C	-3.07994939138026	3.13317848041799	2.05141394544104
H	-2.19061993161988	3.76537426171138	2.17528388822759
H	-3.72371816309793	3.21621288094204	2.93496148824717
O	-5.08242243054125	3.68877802596882	0.86192148818375
C	-0.37493824569527	1.58252638881091	0.61274682564418
O	0.59176672579155	1.36427394059128	2.63683497962653
C	0.46448676726569	-1.02978137500324	2.85634415877288
C	0.22436284935902	0.18478752426629	2.11291251281953
O	0.41400217527443	3.51264918613060	1.91670768019299
C	0.28019038199621	-3.43092586235036	3.02103623424461
C	1.30226180404852	-2.16718320458862	4.81357513135487
C	1.08696369539277	-0.98861746119736	4.11309482452588

C	0.06008808149120	-2.25946842258996	2.31264254994122
H	-0.03525172115424	-4.38764178808552	2.60094049381897
H	1.07027568109632	-4.31173048542299	4.82585542274675
H	-0.42798922764685	-2.26800815831154	1.33740961584992
C	0.90035932508222	-3.38713923308505	4.27044860579764
H	1.78585658882797	-2.13688557595706	5.79151276336103
H	1.39433292391111	-0.02771427551040	4.52722490908682

3. **6c’**

i) S_0 cis

38

Coordinates from ORCA-job neb_reactant

C	0.06210213643688	0.03673803036339	0.03933897675421
C	1.44840501779870	0.09231357511664	-0.00444915732322
C	-0.84955234208265	1.18081560262502	0.03526636773854
C	-0.55361543617555	-1.35011851069813	0.13003705770864
C	2.35085054905842	1.27495188356623	0.15957770634391
N	2.24718983941399	-1.05208759653576	-0.07527982680520
O	2.19488125780934	2.46667997894941	0.27957740109416
O	3.65121338929902	0.70580893585005	0.19958918333508
C	3.48335447560336	-0.65150858987810	0.03701317341901
H	0.19203402257186	-2.08454472772773	-0.23937294232552
O	-0.90301239574975	-1.60509677276158	1.49606557206467
C	4.68183979665618	-1.48258205402139	-0.00113050959124
C	5.96742190803175	-0.90893657362789	0.13252972384053
C	7.10466208215188	-1.72714206843920	0.09414988539862
C	6.97154703439300	-3.11606254569994	-0.07608560449670
C	5.69245352381017	-3.68977031576726	-0.20900521628425
C	4.55164919165829	-2.88138310080907	-0.17228671386151
C	-0.56868434600772	2.39985468351514	-0.64286664673386
C	-2.11025945538275	1.06663223948632	0.68007603023080
H	-1.45252621311529	-1.38365609630268	-0.53187785351174
C	-1.48833180204172	3.44330882629830	-0.66119881361238

H	0.37944407929196	2.52043965363548	-1.17885702383870
C	-2.72738158816275	3.32030768914944	0.01082270485924
H	-1.27583247010639	4.37999219846237	-1.19624171716560
C	-3.03351699542549	2.11665377049055	0.68489514678844
O	-3.55133770780235	4.39763965062362	-0.05405782339467
H	-3.98524953010024	1.99093966928142	1.21800592088010
H	-2.33807903358506	0.13903232866336	1.22530901169208
C	-4.80720564963364	4.33278398928148	0.60466116210031
H	-5.30099465013004	5.30612799798789	0.42911364866057
H	-5.44704572750426	3.52099607998076	0.19264498080768
H	-4.68960795300877	4.17696582638089	1.69999832833289
H	3.54250690095397	-3.30597659843174	-0.27356966403803
H	6.05853620768856	0.17819261537435	0.26619266383685
H	5.58833910940351	-4.77740603792408	-0.34193360887492
H	8.10388826301066	-1.27785273139763	0.19855278644796
H	7.86709048213731	-3.75550580871182	-0.10498829409541
H	-1.23818597116445	-2.51994509634812	1.53497398361865

ii) **6c**

38

Coordinates from ORCA-job neb_product

C	-0.00390501337405	-0.06404284568719	0.04558140568631
C	1.38484550168839	-0.05123005791112	-0.00669990090033
C	-0.84601499991897	1.12971841592620	0.09305525042382
C	-0.71141484258595	-1.41106407966593	0.08218160607810
C	2.27080724266595	-1.25450846383809	-0.05500669750752
N	2.20070332845720	1.08110494892437	-0.01490620240644
O	2.09965139755153	-2.45392998734890	-0.05883066484234
O	3.58237165257686	-0.71346100431153	-0.10375080683675
C	3.43322087831909	0.65381345943341	-0.06938543730702
H	-0.01387426284528	-2.20099491441075	-0.25075184734841
O	-1.17790459746462	-1.66590917915044	1.41470619745471
C	4.64254288619812	1.46958343633869	-0.08849271288455

C	5.91935333486130	0.86981132600818	-0.18967640380379
C	7.06817338207811	1.67211410395999	-0.21056230341970
C	6.95558539654951	3.07088058695258	-0.12976173711586
C	5.68579436243971	3.67048928118408	-0.02600225190722
C	4.53330736639411	2.87818938536250	-0.00509337210599
C	-0.38657263804765	2.43547442167537	-0.25432858821555
C	-2.20663127488710	1.00599561650160	0.48779837560530
H	-1.56646140548206	-1.37762801339464	-0.63416687041479
C	-1.23694658442513	3.53393667144045	-0.21953642478052
H	0.66207131119977	2.55527266292610	-0.55199757660820
C	-2.58715209900339	3.38751932405841	0.18093281862859
H	-0.88515133531787	4.53675133815007	-0.50258910799336
C	-3.06583988626817	2.10836444821084	0.54320881946096
O	-3.33511742342343	4.51952544585095	0.18581882999769
H	-4.10282054727007	1.96447580296120	0.87416221045686
H	-2.58834988244217	0.02716526372748	0.81006259070850
C	-4.69966659840691	4.43258872592152	0.56975551409110
H	-5.11071779802021	5.45560244781221	0.49115034213601
H	-5.27304063343577	3.75694211923729	-0.10272201517651
H	-4.81022288409251	4.07615297930408	1.61779111165056
H	3.53229533422401	3.32471236924413	0.08056706183200
H	5.99452631430730	-0.22496053691126	-0.25140792702855
H	5.59814390593584	4.76552957178963	0.04058528094818
H	8.06030970034612	1.20266863312498	-0.29046730219282
H	7.86023424676722	3.69775164149353	-0.14600548048713
H	-1.05714283584888	-2.61804534489004	1.57779421612467

12a. References:

1. Becke, A. D. Becke’s three parameter hybrid method using the LYP correlation functional. *J. Chem. Phys.* **1993**, 98, 5648−5652.

2. Lee, C.; Yang, W.; Parr, R. G. Development of the ColleSalvetti Correlation-Energy Formula into a Functional of the Electron Density. *Phys. Rev. B: Condens. Matter Mater. Phys.* **1988**, 37 (2), 785−789.

3. Vosko, S. H.; Wilk, L.; Nusair, M. Accurate Spin-Dependent Electron Liquid Correlation Energies for Local Spin Density Calculations: a Critical Analysis. *Can. J. Phys*. **1980**, 58, 1200−1211.

4. Stephens, P. J.; Devlin, F. J.; Chabalowski, C. F.; Frisch, M. J. Ab Initio Calculation of Vibrational Absorption and Circular Dichroism Spectra Using Density Functional Force Fields. *J. Phys. Chem*. **1994**, 98 (45), 11623−11627.

5. Weigend, F.; Ahlrichs, R. Balanced Basis Sets of Split Valence, Triple Zeta Valence and Quadruple Zeta Valence Quality for H to Rn: Design and Assessment of Accuracy. *Phys. Chem. Chem. Phys.* **2005**, *7* (18), 3297-3305.

6. Izsák, R.; Neese, F. Speeding up Spin-Component-Scaled Third-Order Pertubation Theory with the Chain of Spheres Approximation: The COSX-SCS-MP3 Method. *Mol. Phys.* **2013**, 111 (9−11), 1190−1195.

7. Grimme, S.; Antony, J.; Ehrlich, S.; Krieg, H. A Consistent and Accurate Ab Initio Parametrization of Density Functional Dispersion Correction (DFT-D) for the 94 Elements H-Pu. *J. Chem. Phys.* **2010**, 132 (15), 154104.

8. Grimme, S.; Ehrlich, S.; Goerigk, L. Effect of the Damping Function in Dispersion Corrected Density Functional Theory. *J. Comput. Chem.* **2011**, 32 (7), 1456−1465.

9. Neese, F. The ORCA Program System. *Wiley Interdiscip. Rev.: Comput. Mol. Sci.* **2012**, 2 (1), 73−78.

10. Casida, M. E.; Huix-Rotllant, M. Progress in Time-Dependent Density-Functional Theory. *Annu. Rev. Phys. Chem*. **2012**, 63, 287−323.

11. Marques, M. A. L.; Gross, E. K. U. Time-dependent density functional theory. *Annu. Rev. Phys. Chem*. **2004**, 55, 427−455.

12. Dreuw, A.; Head-Gordon, M. Single-reference ab initio methods for the calculation of excited states of large molecules. *Chem. Rev.* **2005**, 105, 4009−4037.

13. Yanai, T.; Tew, D. P.; Handy, N. C. A New Hybrid Exchange-Correlation Functional Using the Coulomb-Attenuating Method (CAM-B3LYP). *Chem. Phys. Lett.* **2004**, 393 (1-3), 51–57.

14. Lu, T.; Chen, F. Multiwfn: A Multifunctional Wavefunction Analyzer. *J. Comput. Chem.* **2012**, 33 (5), 580–592.
